# Palladium- and Brønsted acid-catalyzed enantio-, site- and *E*/*Z*-selective addition of alkylidenecyclopropanes with imines†

**DOI:** 10.1039/d2sc05674g

**Published:** 2023-02-04

**Authors:** Xin-Lian Liu, Han-Ze Lin, Lu-Qi Tan, Jin-Bao Peng

**Affiliations:** a School of Biotechnology and Health Sciences, Wuyi University Jiangmen Guangdong 529020 People's Republic of China pengjb_05@126.com

## Abstract

Transition-metal catalyzed functionalization of ACPs has been widely investigated in cycloaddition and 1,3-difunctionalization reactions. However, the transition metal catalyzed nucleophilic reactions of ACPs have rarely been reported. In this article, an enantio-, site- and *E*/*Z*-selective addition of ACPs with imines for the synthesis of dienyl substituted amines has been developed *via* palladium- and Brønsted acid co-catalysis. A range of synthetically valuable dienyl substituted amines were effectively prepared with good to excellent yields and excellent enantio- and *E*/*Z*-selectivities.

## Abstract 2

An enantio-, site- and *E*/*Z*-selective addition of ACPs with imines for the synthesis of dienyl substituted amines has been developed *via* palladium- and Brønsted acid co-catalysis.

## Graphical abstract

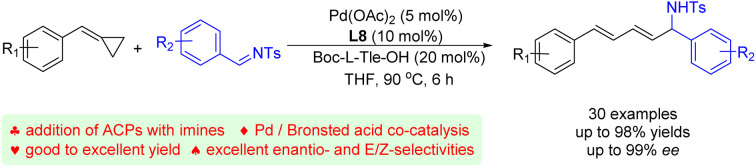

## Introduction

Three-membered carbocycles have found many applications not only as versatile building blocks for organic chemistry^1^ but also as valuable targets of synthesis.^2^ Among them, alkylidenecyclopropanes (ACPs) and methylenecyclopropanes (MCPs), containing an *exo*-C=C double bond, exhibit a higher strain energy and are thus more reactive than simple cyclopropanes.^3^ Nevertheless, these highly strained molecules are surprisingly stable and are readily accessible from simple materials. In addition to their high strain energy, the presence of the double bond, which allows coordination to the transition-metal, leads to an additional variety of activation processes. Their unique structural and electronic properties have therefore attracted considerable interest both in synthetic and mechanistic studies. A series of very interesting and characteristic transformations have been developed in the past decades.^4^ Among them, the ring-opening and ring-expansion reactions of ACPs are usually favorable owing to the concomitant release of cyclopropane ring strain.

**Scheme 1:**
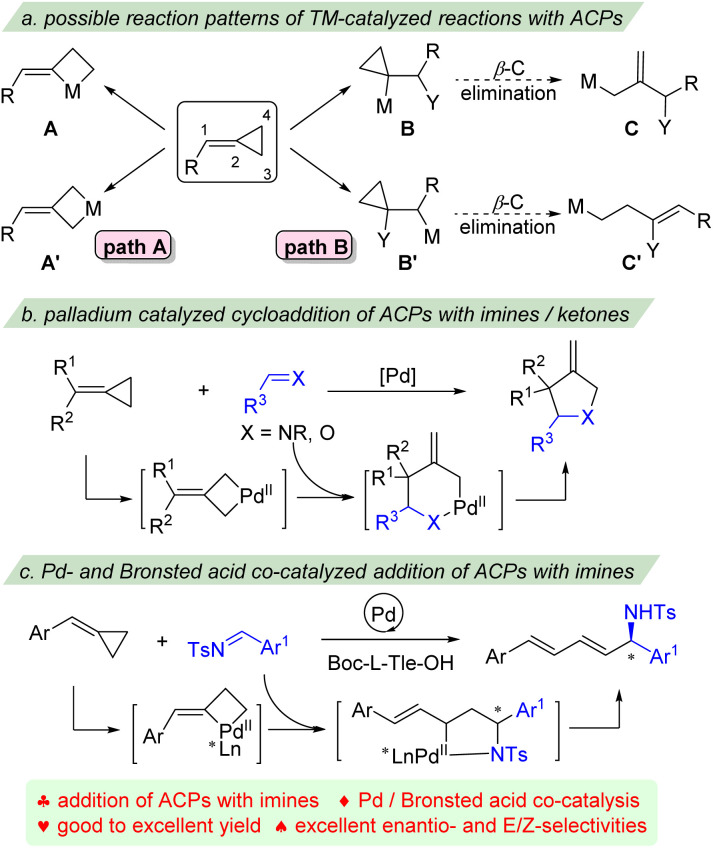
Transition metal-catalyzed reaction of ACPs. (a) Possible reaction patterns of TM-catalyzed reactions with ACPs. (b) Palladium catalyzed cycloaddition of ACPs with imines/ketones. (c) Pd- and Brønsted acid co-catalyzed addition of ACPs with imines.

Recently, impressive progress has been made in transition-metal catalyzed functionalization of ACPs.^5^ Generally, the reactions of ACPs with transition-metal catalysis can occur *via* two different reaction patterns (Scheme 1). The first is the direct oxidative addition of low valence transition-metal to the cyclopropane of ACPs, either addition into the proximal bond to give metallacyclobutane A, or addition into the distal bond to provide intermediate A′. These cyclic metal complexes have been shown versatile for cycloaddition reactions as three carbon (3C) components to afford different types of carbocyclic products (Scheme 1a, path A).^6^ For example, the intra- and intermolecular [3 + 2], [3 + 2 + 1], [3 + 2 + 2] and [4 + 3] cycloaddition reactions of ACPs with different unsaturated partners have been well established by the groups of López, Evans, Saito, Shi, and others. The second reaction pattern is based on the carbometalation of the *exo*-methylene part with organometallic species to form two regioisomeric intermediates B and B′, which can further undergo β-carbon elimination to give allyl-metal species C and homoallyl-metal intermediate C′, respectively (Scheme 1a, path B).^7^ The palladium catalyzed heterocycloaddition of ACPs with ketones and imines to give highly substituted tetrahydrofuran and pyrrolidine derivatives has also been reported (Scheme 1b).^8^ These reactions usually proceed *via* distal C–C bond insertion, followed by isomerization/migratory insertion or metallo-ene process to afford the cyclic products.

These strategies produce 1,3-functionalized products of ACPs. However, the transition metal catalyzed nucleophilic reactions of ACPs have rarely been reported.^9^ Recently, we developed a palladium-catalyzed ligand-controlled selective 1,4-addition and cycloaddition reaction of ACPs with β,γ-unsaturated α-ketoesters, however, attempts for the enantioselective reaction failed.^10^ We envisioned that the interaction of nitrogen of imines with the catalyst would help the control of the enantioselectivity. Herein, we report an enantio-, site- and *E*/*Z*-selective addition of ACPs with imines *via* palladium- and Brønsted acid co-catalysis (Scheme 1c). A range of dienyl substituted amines were effectively prepared with good to excellent yields and excellent enantio- and *E*/*Z*-selectivities.

## Results and discussion

Initially, naphthyl substituted ACP 1a and imine 2a were selected as model substrates to evaluate the feasibility of the ring opening addition reaction. To our delight, using Pd(OAc)_2_ as the catalyst and PPh_3_ as the ligand, when a solution of 1a and 2a in toluene was stirred at 100 °C for 6 h, the 1,4-addition product *rac*-3aa was obtained in 60% yield (see details in the ESI†). After preliminary screening of the reaction conditions including catalyst, ligand, temperature and solvent, we found that *rac*-3aa could be obtained in 96% yield using the Pd(OAc)_2_/BuPAd_2_ catalyst system in THF (see details in the ESI†). Then, we focused on developing an enantioselective addition of ACPs with imines. We found that replacement of BuPAd_2_ with a chiral ligand (*S,S*)-Ph-BPE (L1) gave the desired product in 6% yield and 2% ee (Table 1, entry 1). Biaryl bisphosphine ligand SEGPHOS (L2) provided 3aa in 57% yield with a moderate ee of 47% (Table 1, entry 2). However, increasing the steric bulk of the phosphine substitute decreased the enantioselectivity dramatically (Table 1, entry 3). Phosphoramidite ligands were found to be effective for this reaction (Table 1, entries 4–8). The desired product 3aa was produced in good yields and moderate ee when phosphoramidite ligands L4–L7 were used. When TADDOL-derived phosphoramidites L8 was used as the ligand, 3aa was obtained in 70% yield and 65% ee. Notably, the concentration of the reactants affected both the yield and the enantioselectivity of this reaction significantly. Higher yield and enantioselectivity were obtained when the reaction was carried out with higher concentration (Table 1, entries 8–10). Recently, dual catalysis by transition metal and a Brønsted acid has been proven to be a powerful strategy for redox-neutral coupling of alkenes and carbonyl compounds.^11^ Thus, a series of Brønsted acids were tested in this reaction. Pleasingly, both the reactivity and the selectivity were improved with the addition of *N*-Boc-L-*tert*-Leucine (Boc-L-Tle-OH). 3aa was obtained in high yield and excellent ee (Table 1, entry 11). Other Brønsted acids such as Ac-Phe-OH and 1-naphthoic acid gave similar enantioselectivity but lower yields (Table 1, entries 13 and 14). It should be mentioned that the used of Boc-D-Tle-OH greatly reduced the yield and stereoselectivity due to the mis-matched effect (Table 1, entry 12).

**Table 1:** Optimization of the reaction conditions^a^

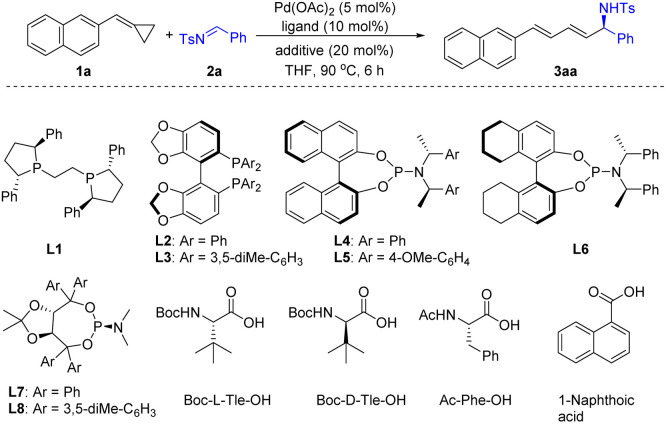				
Entry	Ligand	Additive	Yield^b^ [%]	ee^c^ [%]
1	L1	—	6	2
2	L2	—	56	47
3	L3	—	64	—
4	L4	—	87	41
5	L5	—	74	53
6	L6	—	85	20
7	L7	—	53	47
8	L8	—	70	65
9^d^	L8	—	72	78
10^e^	L8	—	75	91
11^e^	L8	Boc-L-Tle-OH	88	97
12^e^	L8	Boc-D-Tle-OH	63	54
13^e^	L8	Ac-Phe-OH	67	97
14^e^	L8	1-Naphthoic acid	73	94

a Reaction conditions: 1a (0.10 mmol), 2a (0.12 mmol), Pd(OAc)_2_ (5 mol%), L (10 mol%), additive (20 mol%), THF (1 mL), 6 h.

b Isolated yields.

c Determined by HPLC analysis on a chiral stationary phase.

d THF (0.5 mL).

e THF (0.3 mL).

**Scheme 2:**
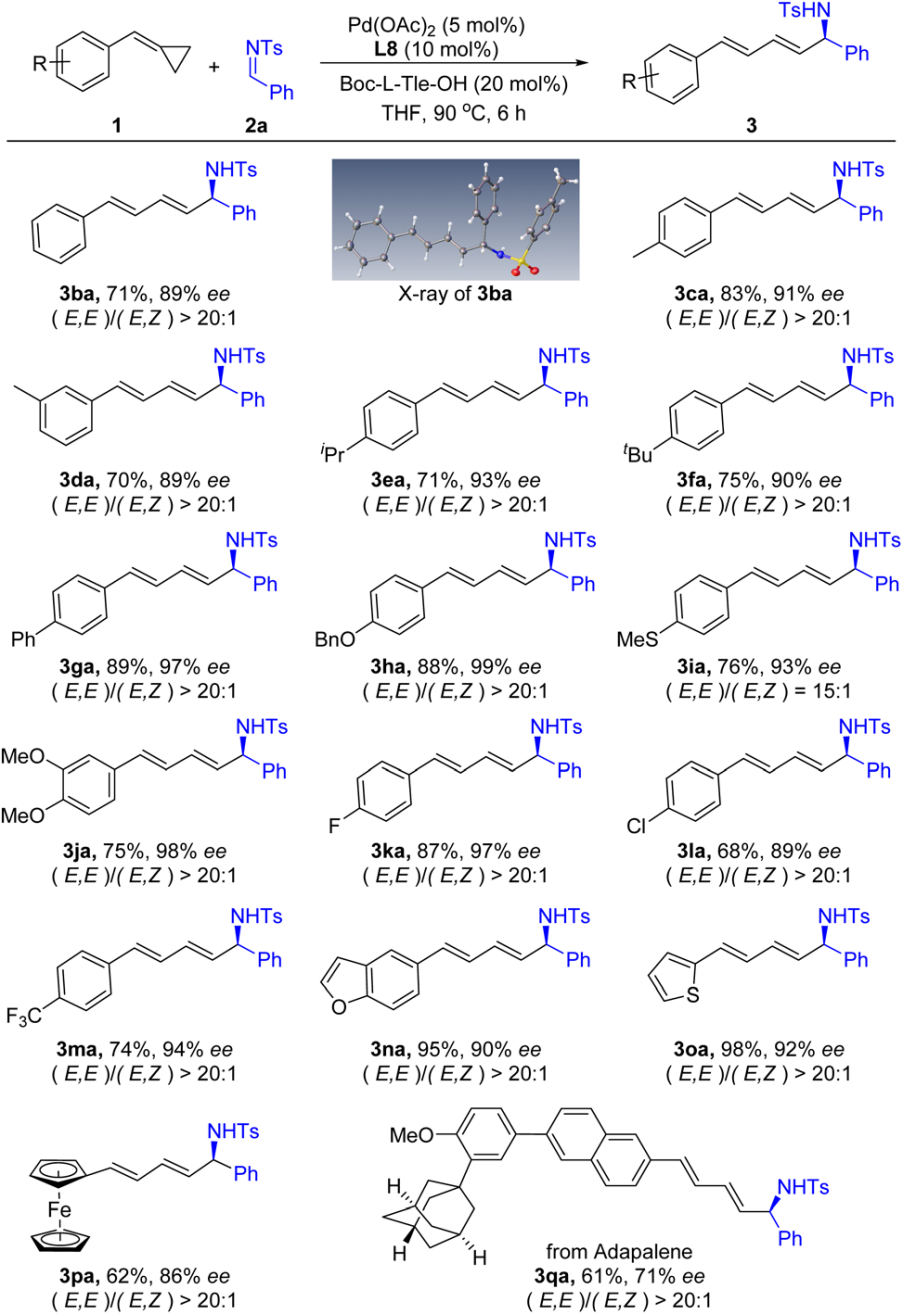
Substrate scope of ACPs. Reaction conditions: 1a (0.10 mmol), 2a (0.12 mmol), Pd(OAc)_2_ (5 mol%), L8 (10 mol%), Boc-L-Tle-OH (20 mol%), THF (0.3 mL), 6 h. Isolated yields, ee determined by HPLC analysis on a chiral stationary phase.

With the optimized reaction conditions in hand, we turned our attention to explore the substrate scope of this asymmetric 1,4-addition reaction. First, a range of substituted ACPs 1 was applied to react with *N*-Ts imine 2a under the optimized reaction conditions. As summarized in Scheme 2, various aryl substituted ACPs were well tolerated and produced the corresponding products in good yields with excellent enantioselectivity. Both electron-donating (3ca–3ja) and electron-withdrawing (3ka–3ma) substituents were compatible on the benzene ring of ACPs. Functional groups such as thioether- (3ia), fluoro- (3ka), chloro- (3la) and trifluoromethyl-groups (3ma) were compatible in this reaction. Gratifyingly, heteroaryl-substituted ACPs were tolerated as well. For example, 5-benzofuranyl and 3-thienyl substituted ACPs reacted with imine 2a smoothly and afforded the corresponding products 3na and 3oa in 95% and 98% yields, respectively. In addition, ACP with a ferrocene group was also tolerated in this reaction and provided the desired product 3pa in 62% yield and 86% ee. Moreover, when ACP 1q derived from adapalene was treated with 2a under standard conditions, the target product 3qa was obtained in moderate yield and enantioselectivity. Notably, the reaction proceeds in an excellent stereoselective manner. The products were obtained as *E*,*E*-isomers, and only trace amounts of *E*,*Z*-isomers were observed in some cases. The structure of the products was assigned based on X-ray crystallography analysis of 3ba as a representative example.^12^

**Scheme 3:**
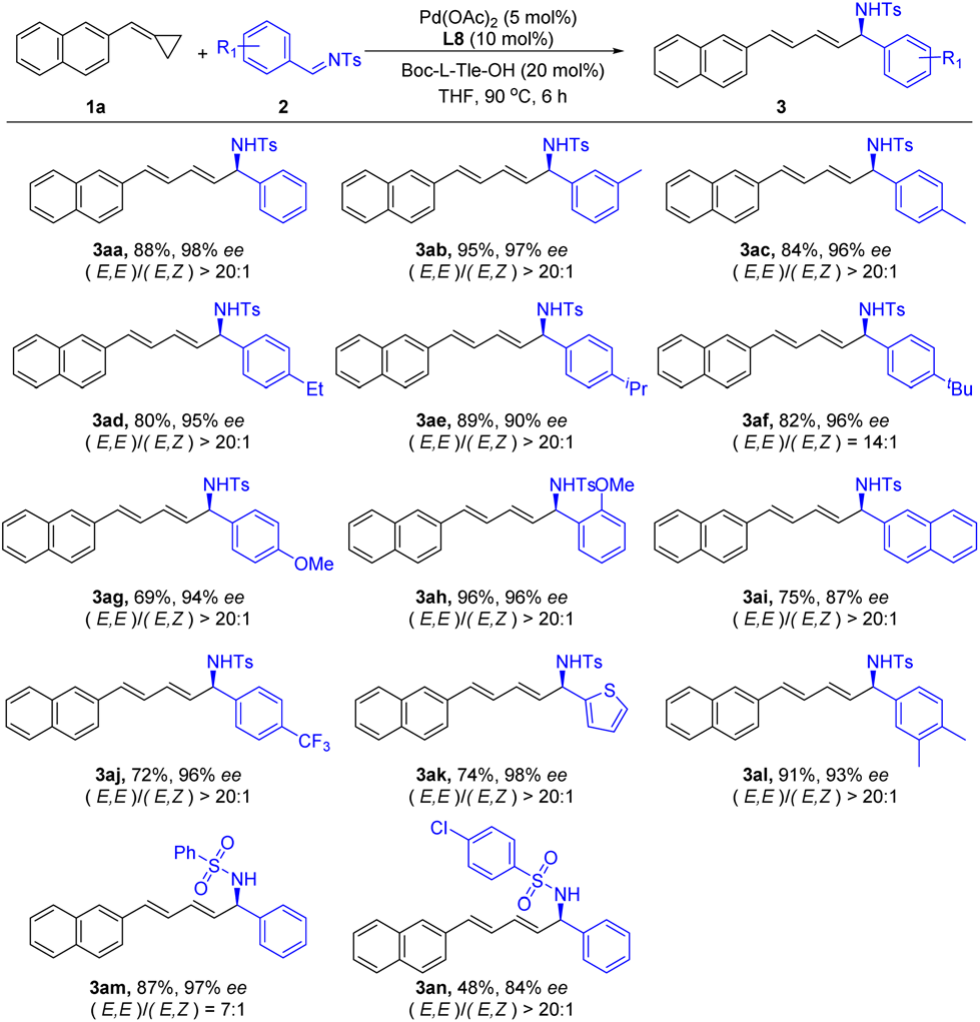
Substrate scope of imine. reaction conditions: 1a (0.10 mmol), 2a (0.12 mmol), Pd(OAc)_2_ (5 mol%), L8 (10 mol%), Boc-L-Tle-OH (20 mol%), THF (0.3 mL), 6 h. Isolated yields, ee determined by HPLC analysis on a chiral stationary phase.

Then, we further examined the scope of the imine 2 to demonstrate the generality of this reaction (Scheme 3). A group of *N*-Ts imines 2 possessing different substitutions at different positions of the phenyl ring were successfully applied in this reaction and produced the corresponding products in good to excellent yields with excellent enantioselectivities. In addition, a 2-naphthyl-based imine 2l also reacted smoothly and gave the desired product 3al in a 75% yield and 87% ee. Heteroaryl groups such as 3-thiophenyl were tolerated as well, delivering the corresponding product 3ak in high yields with excellent enantioselectivity. *N*-Sulfonyl substituted imines 2m and 2n were also suitable substrates in this reaction and produced the corresponding products 3am and 3an, respectively. However, no desired reaction was observed when imines with *N*-phenyl and *N*-butyl groups were used in this reaction.

**Scheme 4:**
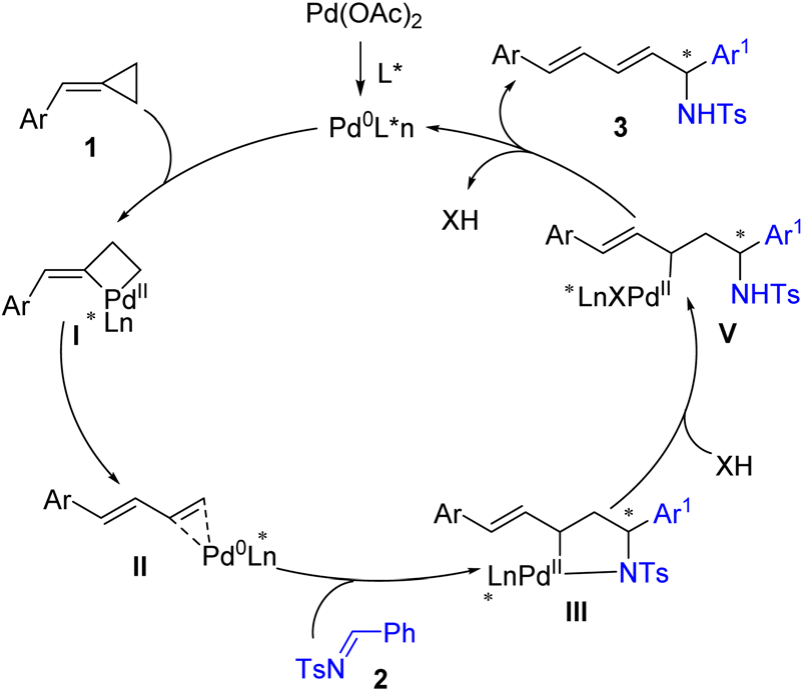
Proposed catalytic cycle.

On the basis of the experimental results and the previous literature,^10,11^ a plausible catalytic cycle is proposed in Scheme 4. First, the oxidative addition of the *in situ* generated Pd(0) to the proximal C–C bond of ACPs 1 generates a cyclic Pd(ii) complex I. The cyclic Pd(ii) complex I undergoes β-H elimination and reductive elimination to give an *η*^2^-coordinated 1,3-diene intermediate II. Then, intermediate II undergoes enantioselective cyclopalladation with imines 2 to give a cyclic palladium intermediate III. Intermediate III was then protonated by Brønsted acid to give complex IV, which underwent reductive elimination to release the addition product 3 and regenerated Pd(0) for the next catalytic cycle. The use of Brønsted acid activated the imines and provided an extra means to tune the enantioselectivity of the products.

## Conclusions

In summary, we have developed an enantio-, site- and *E*/*Z*-selective addition of ACPs with imines *via* palladium- and Brønsted acid co-catalysis. A range of synthetically valuable dienyl substituted amines were effectively prepared with good to excellent yields and excellent enantio- and *E*/*Z*-selectivities.

## Data availability

All experimental data and detailed procedures are available in the ESI.†

## Author contributions

J.-B. P. conceived and directed the project. X.-L. L. performed the experiments. H.-Z. L. and L.-Q. T. participated in substrate synthesis and discussions. X.-L. L. and J.-B. P. wrote the manuscript and ESI†.

## Conflicts of interest

There are no conflicts to declare.

## Supplementary Material

SC-014-D2SC05674G-s001

SC-014-D2SC05674G-s002
