# Supplementary material for: Palladium- and Brønsted acid-catalyzed enantio-, site- and E/Z-selective addition of alkylidenecyclopropanes with imines
Source: Chem Sci. 2023 Feb 4;14(9):2348–52. doi: 10.1039/d2sc05674g (PMC9977444; doi:10.1039/d2sc05674g)
Supplement: SC-014-D2SC05674G-s001 [file SC-014-D2SC05674G-s001.pdf]

Electronic Supplementary Material (ESI) for Chemical Science.
This journal is © The Royal Society of Chemistry 2023

Supporting Information

Palladium- and Brønsted Acid-Catalyzed Enantio-, Site- and E/Z-Selective Addition of Alkylidenecyclopropanes with Imines

Xin-Lian Liu,[†] Han-Ze Lin,[†] Lu-Qi Tan,[†] and Jin-Bao Peng*,[†]

[†]School of Biotechnology and Health Sciences, Wuyi University, Jiangmen, Guangdong 529020, People's Republic of China; orcid.org/0000-0002-0568-7740; E-mail: pengjb_05@126.com

Table of Contents

Supporting Information 1

1. General Information 1

2. Substrate Synthesis 1

    2.1 Synthesis of Alkylidenecyclopropanes (ACPs) 1

    2.2 N-Ts imines 2

3. Optimization of Reaction Conditions 3

4. General Procedure 7

5. Exploration of Products 7

    5.1. Spectroscopic Data of Products 7

    5.2. Unsuccessful Substrates Attempts 20

6 X-ray Crystal Structure Determination of the Products 21

7. References 22

8. Copies of NMR Spectra for Compounds 24

1. General Information

Reagents, solvents and analytical methods:

Unless otherwise noted, all reactions were carried out under a nitrogen atmosphere. All reagents were from commercial sources and used as received without further purification. All solvents were dried by standard techniques and distilled prior to use. Column chromatography was performed on silica gel (200-300 meshes) using petroleum ether (bp. 60~90 °C) and ethyl acetate as eluent. ^{1}NMR spectra were recorded on a Bruker Avance operating at for ^{1}H NMR at 500 MHz, ^{13}C NMR at 126 MHz and ^{19}F NMR at 471 MHz and spectral data were reported in ppm relative to tetramethylsilane (TMS) as internal standard and $CDCl_3$ (^{1}H NMR δ 7.27, ^{13}C NMR δ 77.0) as solvent. High-resolution mass spectra (HRMS) is produced by Thermo Fisher Scientific. Its main body is composed of two parts: Thermo Scientific's UltiMate 3000 Series liquid system and Thermo Scientific Q-Exactive combined quadrupole Orbitrap mass spectrometer. All coupling constants (*J*) are reported in Hz. The following abbreviations were used to describe peak splitting patterns when appropriate: s = singlet, d = doublet, dd = double doublet, ddd = double doublet of doublets, t = triplet, dt = double triplet, q = quatriplet, m = multiplet, br = broad. All chemicals were used without purification as commercially available unless otherwise noted. **1a-1q** were prepared according to the previous literature.[1-4] **2a-2o** were prepared according to the previous literature.[5]

2. Substrate Synthesis

2.1 Synthesis of Alkylidenecyclopropanes (ACPs)

Compounds **1a-1q** were prepared according to the previous literature.[1] **1a-1c**[1], **1d-1g**[4], **1h**[2], **1i**[3], **1j-1l**[2] are known compounds.

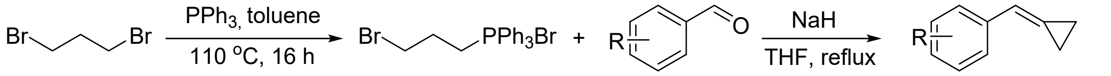

An oven-dried 250 mL two-neck round-bottom flask equipped with a Teflon magnetic stirringbar, a reflux condenser and an oil bubbler were connected to nitrogen. The system was charged with PPh_3 (25.0 g, 95.2 mmol, 1.0 equiv) and toluene (100 mL). The vessel was sealed with a rubber septum and vigorously stirred at room temperature before adding 1,3-dibromopropane (10.2 mL, 20.2 g, 100 mmol, 1.05 equiv). The system was placed on a heating block and the temperature was increased to 115 °C with vigorously stirring. After 16 h, the reaction was cooled down to room temperature. The white precipitate was filtered off in vacuo, washed with toluene (3×15 mL) and dried under reduced pressure to afford phosphonium salts as a white solid.

A solution of (4-bromobutyl)triphenylphosphonium bromide (6.01 g, 13 mmol) and NaH (1.04 g, 26 mmol) in THF (25 mL) was stirred at 70 °C under N_2 for 12 h. Afterwards compound aldehyde (10 mmol) in THF (5 mL) was added and the reaction solution was stirred at 70 °C until compound aldehyde was consumed completely. The reaction mixture was cooled to room temperature, and the mixture was filtered through a celite. The filtrate was concentrated under

reduced pressure and the residue was purified by silica gel flash chromatography (eluent: petroleum ether) to afford the product BCPs in moderate yield.

1-(5-(6-(cyclopropylidenemethyl)naphthalen-2-yl)-2-methoxyphenyl)adamantane **(1q)**

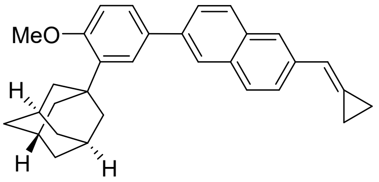

^{1}H NMR (500 MHz, CDCl$_3$) δ 7.95 (s, 1H), 7.89 – 7.82 (m, 3H), 7.72 (dd, J = 8.5, 1.8 Hz, 1H), 7.60 (d, J = 2.3 Hz, 1H), 7.54 (dd, J = 8.4, 2.3 Hz, 1H), 7.00 (d, J = 8.4 Hz, 1H), 6.97 – 6.92 (m, 1H), 3.91 (s, 3H), 2.17 (t, J = 22.5 Hz, 9H), 1.82 (s, 6H), 1.57 – 1.51 (m, 4H).

^{13}C NMR (126 MHz, CDCl$_3$) δ 158.6, 139.0, 138.7, 135.7, 133.3, 133.0, 132.6, 128.4, 128.3, 126.0, 126.0, 125.7, 125.0, 124.9, 124.9, 118.7, 112.2, 55.3, 40.8, 37.3, 37.3, 29.3, 4.6, 1.5.

HRMS (ESI): *m/z* calculated for $C_{31}H_{32}O$ $[M+H]^+$: 421.2526, found: 421.2521.

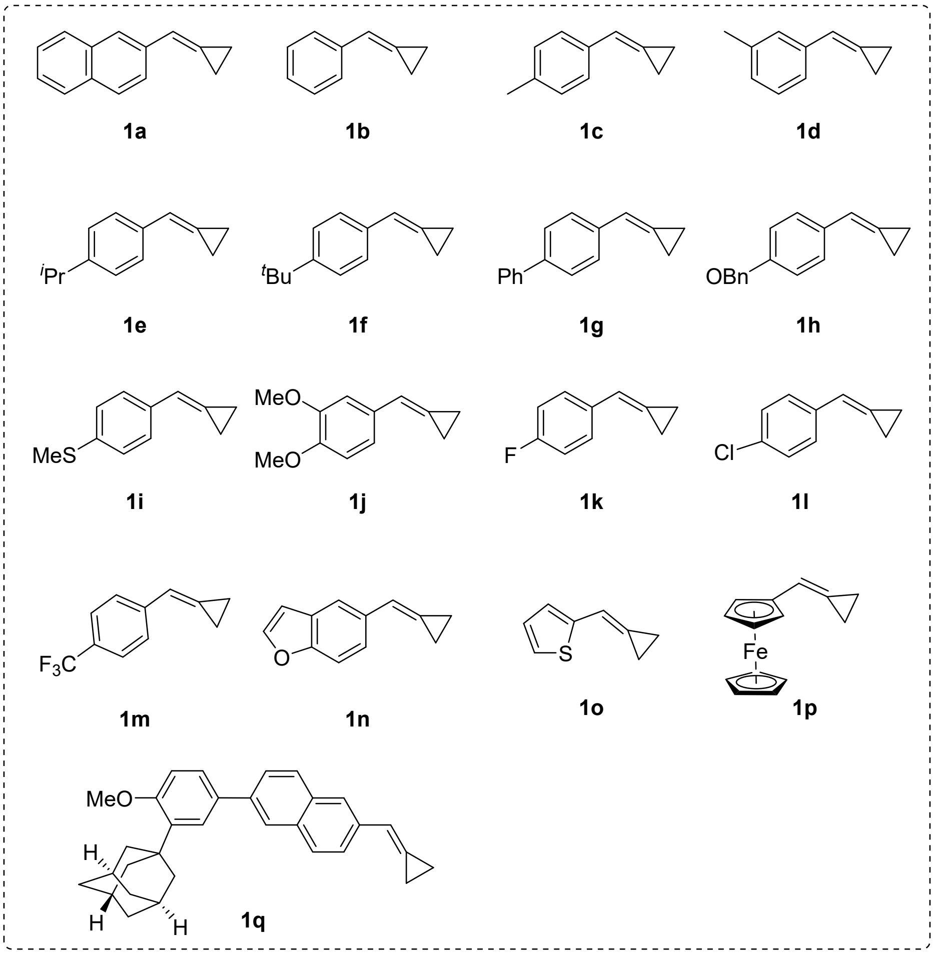

Figure S1 Substrates of Alkylidenecyclopropanes

2.2 N-Ts imines

Compounds **2a** – **2o** were synthesized according to the known method.[5]

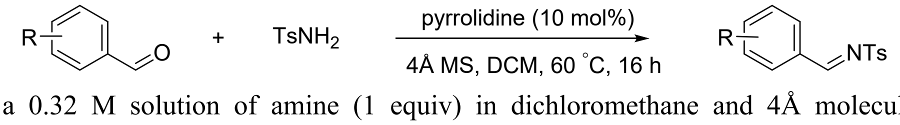

To a 0.32 M solution of amine (1 equiv) in dichloromethane and 4Å molecular sieves (1g/mmol) the corresponding aldehyde (equivalents indicated in each case) and 10 mol% of pyrrolidine (stock solution in dichloromethane) were added. The mixture was stirred in a sealed

vial at 60° C. After 16 h, the reaction was cooled down to room temperature. Then, the reaction was filteredb through either a short pad of Celite. Water (20 mL) was added to the crude product, which was then extracted with dichloromethane (20 mL ×2). The combined organic phase was washed with a saturated solution of NaCl (15 mL). The combined organic fractions were dried with anhydrous Na_2SO_4, and distilled of at reduced pressure. The crude product was recrystallized to obtain the imine product, or the residue was purified by silica gel flash chromatography (petroleum ether / ethyl acetate = 5:1) to afford the product imine in moderate yield.

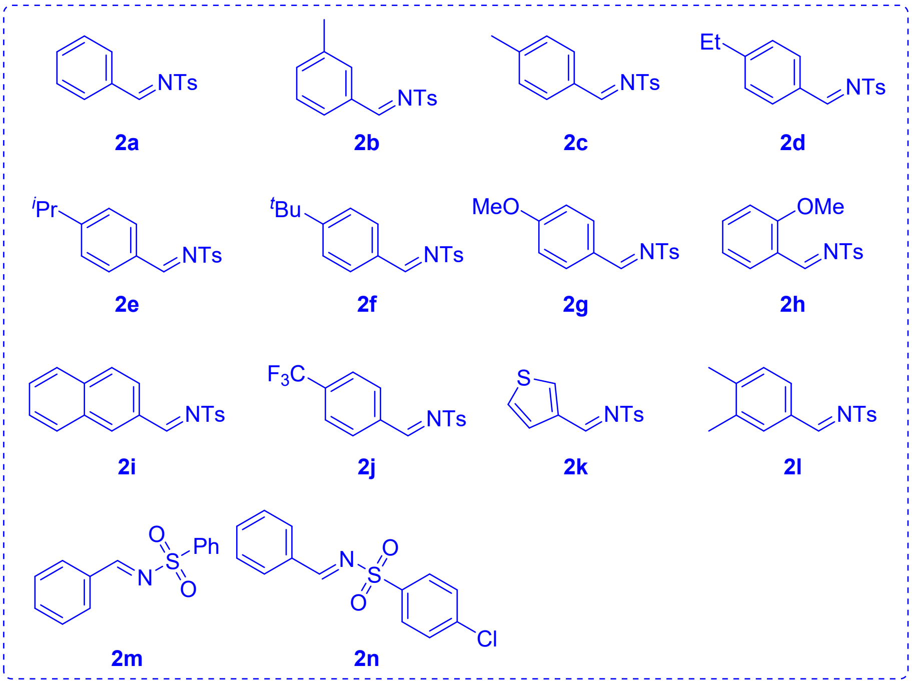

Figure S2 Substrates of *N-Ts imine*

3. Optimization of Reaction Conditions

Table S1. Optimization of the catalyst.[a]

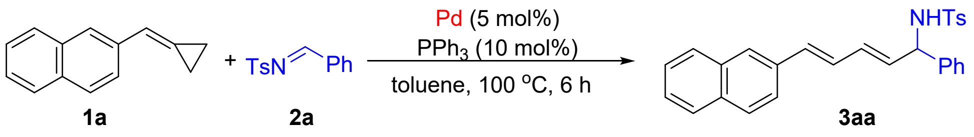

Entry	Catalyst	Yield (%)[b]
1	$Pd_2(dba)_3$	56
2	$Pd(OAc)_2$	60
3	$Pd(TFA)_2$	trace
4	$Pd(acac)_2$	48
5	$PdCl_2$	trace
6	$Pd(pph_3)_4$	40
7	No Pd	trace

[a]Reaction conditions: **1a** (0.10 mmol), **2a** (0.12 mmol), Pd (5 mol%), PPh_3 (10 mol%) in toluene

(0.5 mL) at 100 ºC for 6 h. [b]Isolated yield.

Table S2. Optimization of ligand.[a]

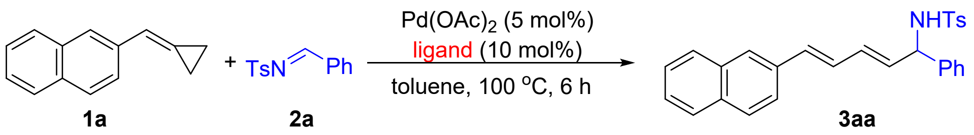

Entry	Ligand	Yield (%)[b]
1	pph$_3$	60
2	BuPAd$_2$	71
3	Pcy$_3$	46
4	DPPP	71
5	BINAP	-

[a]Reaction conditions: **1a** (0.10 mmol), **2a** (0.12 mmol), Pd(OAc)$_2$ (5 mol%), ligand (10 mol%) in toluene (0.5 mL) at 100 ºC for 6 h. [b]Isolated yield.

Table S3. Optimization of the solvent.[a]

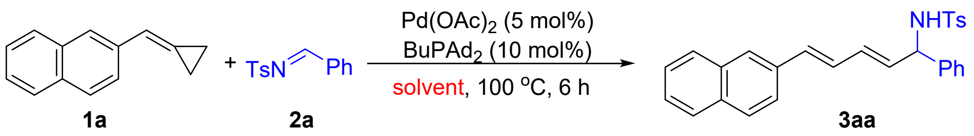

Entry	Solvent	Yield (%)[b]
1	toluene	71
2	chlorobenzene	trace
3	MeCN	32
4	DCE	trace
5	THF	82
6	DMSO	trace
7	fluorobenzene	59
8	dioxane	71

[a]Reaction conditions: **1a** (0.10 mmol), **2a** (0.12 mmol), Pd(OAc)$_2$ (5 mol%), BuPAd$_2$ (10 mol%) in solvent (0.5 mL) at 100 ºC for 6 h. [b]Isolated yield.

Table S4. Optimization of temperature.[a]

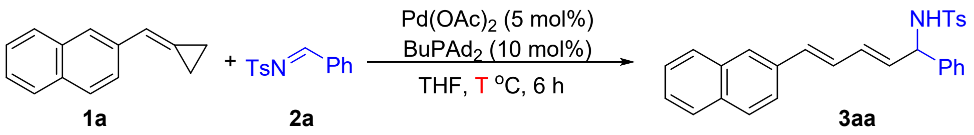

Entry	Temp. (°C)	Yield (%)[b]
1	70	45
2	80	49
3	90	89
4	100	82

5	110	77

[a]Reaction conditions: **1a** (0.10 mmol), **2a** (0.12 mmol), $Pd(OAc)_2$ (5 mol%), $BuPAd_2$ (10 mol%) in THF (0.5 mL) at T °C for 6 h. [b]Isolated yield.

Table S5. Optimization of the equivalent of solvent.[a]

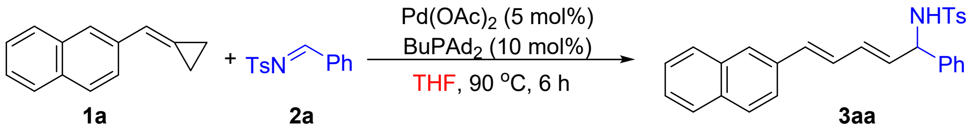

Entry	Solvent (x mL)	Yield (%)[b]
1	0.3	96
2	0.5	89
3	1	60

[a]Reaction conditions: **1a** (0.10 mmol), **2a** (0.12 mmol), $Pd(OAc)_2$ (5 mol%), $BuPAd_2$ (10 mol%) in THF (x mL) at 90 °C for 6 h. [b] Isolated yield.

Table S6. Optimization of the ligand.[a]

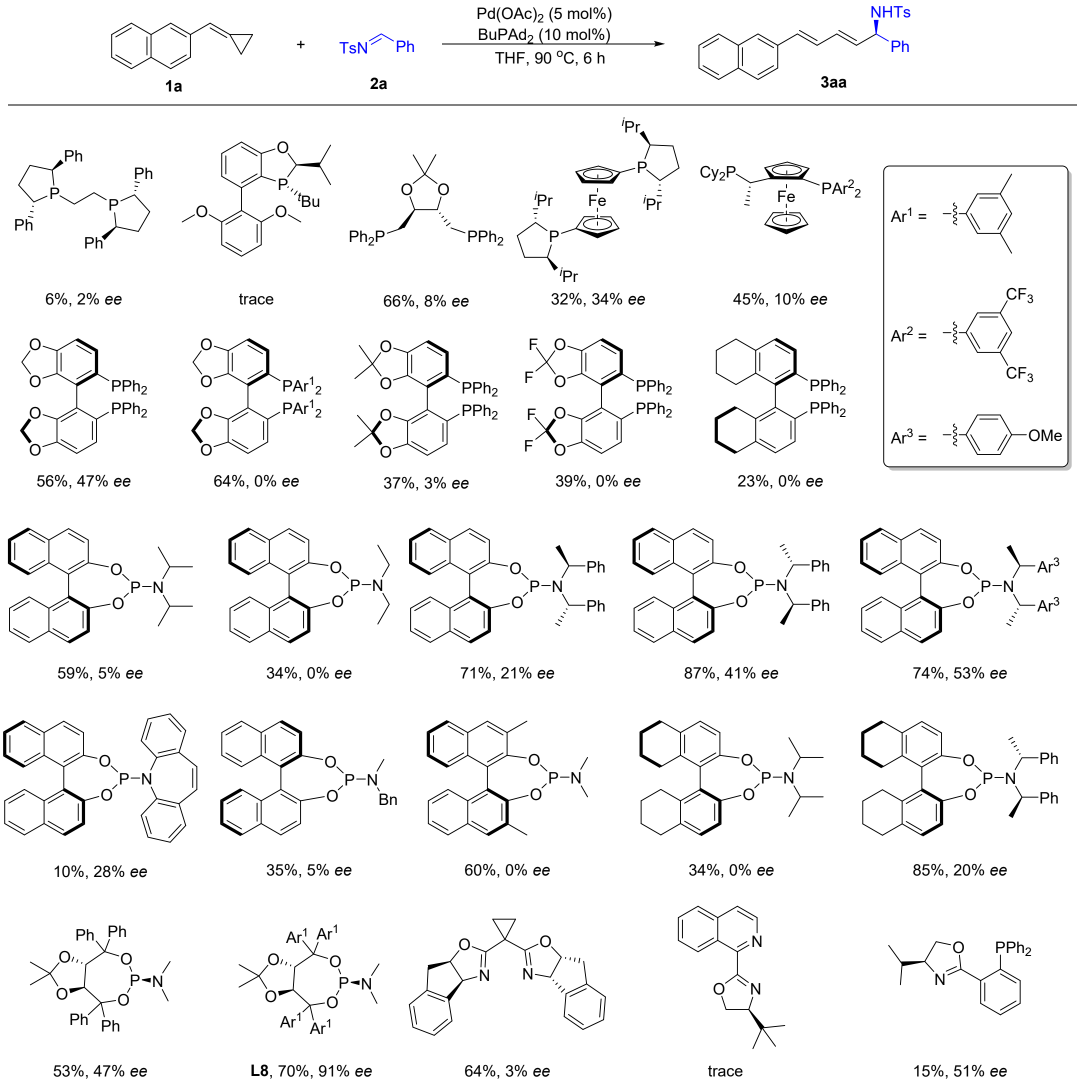

Reaction conditions: **1a** (0.10 mmol), **2a** (0.12 mmol), $Pd(OAc)_2$ (5 mol%), **L** (10 mol%), at THF (0.3 mL) for 6 h.

Table S7. Optimization of the equivalent of solvent.[a]

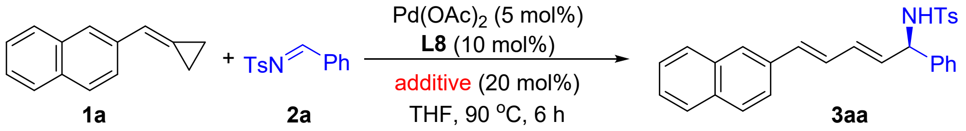

Entry	Additive	Yield (%)[b]	*ee* (%)[c]
1	none	70	65
2[d]	none	72	78
3[e]	none	75	91
4[e]	Triethylalumine	-	-
5[e]	(S)-(+)-BNDHP	NR	-
6[e]	1-Naphthoic acid	73	94
7[e]	Boc-L-Tle-OH	88	97
8[e]	Boc-L-Val-OH	91	94

9[e]	Ac-Phe-OH	67	97
10[e]	Boc-D-Tle-OH	63	54

[a]Reaction conditions: **1a** (0.10 mmol), **2a** (0.12 mmol), $Pd(OAc)_2$ (5 mol%), **L8** (10 mol%), additive (20 mol%) in THF (1 mL) at 90 °C for 6 h. [b]Isolated yield. [c]Determined by HPLC analysis on a chiral stationary phase.[d]THF (0.5 mL). [e]THF (0.3 mL).

4. General Procedure

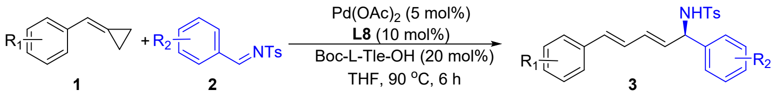

In a 15 mL Schlenk tube equipped with magnetic stir bar was charged with **1** (0.1 mmol, 1.0 eq.), **2** (0.12 mmol, 1.2 eq.), $Pd(OAc)_2$ (0.1 mg, 0.005 mmol, 0.05 eq.), **L8** (6.5 mg, 0.01 mmol, 0.1 eq.), Boc-L-Tle-OH (4.6 mg, 0.02 mmol, 0.2 eq.), The tube was connected to an nitrogen-vacuum line, evacuated and backfilled with N_2 (×3). THF (0.3 mL) was added to the reaction tube. and the Schlenk tube was sealed quickly. The reaction mixture was stirred at 90 °C for 6 hours. The mixture was concentrated under reduced pressure and the residue was purified by flash chromatography on silica gel eluting with petroleum ether/EtOAc (v/v = 10:1 to 5:1) to afford the products **3**.

5. Exploration of Products

5.1. Spectroscopic Data of Products

4-methyl-*N-((S,2E,4E)*-5-(naphthalen-2-yl)-1-phenylpenta-2,4-dien-1-yl)benzenesulfonamide **(3aa)** (Known compound).[6]

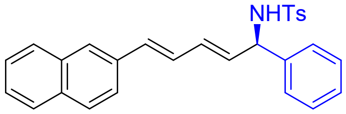

The reaction performed according to the standard procedure A afforded 38.6 mg (88% yield, *(E,E)/(E,Z)* > 20:1). Colorless semisolid.

^{1}H NMR (500 MHz, $CDCl_3$) δ 7.78 (dd, J = 11.7, 8.9 Hz, 3H), 7.69 (d, J = 8.3 Hz, 3H), 7.55 (d, J = 9.9 Hz, 1H), 7.50 – 7.42 (m, 2H), 7.25 (t, J = 6.6 Hz, 3H), 7.20 (t, J = 8.8 Hz, 4H), 6.74 (dd, J = 15.6, 10.4 Hz, 1H), 6.59 (d, J = 15.6 Hz, 1H), 6.22 (dd, J = 15.1, 10.5 Hz, 1H), 5.79 (dd, J = 15.1, 6.7 Hz, 1H), 5.20 (d, J = 7.3 Hz, 1H), 5.07 (t, J = 6.9 Hz, 1H), 2.36 (s, 3H).

^{13}C NMR (126 MHz, $CDCl_3$) δ 143.4, 139.8, 137.8, 134.5, 133.8, 133.71, 133.2, 132.7, 132.3, 129.6, 128.8, 128.4, 128.1, 128.0, 128.0, 127.8, 127.5, 127.2, 126.7, 126.5, 126.1, 123.4, 59.7, 21.6.

IR (thin film): 3842, 3741, 3612, 3272, 3031, 2357, 1597, 1499, 1431, 1323, 1156, 1089, 1032, 987, 920, 813, 750, 669, 557, 474 cm^{-1}.

$[\alpha]_D^{23}$ -40.0 (c 1.0, $CHCl_3$, 98% *ee* sample).

Enantiomeric excess was determined to be 98% *ee* by chiral HPLC analysis (CHIRALPAK IA (n-hexane/^{i}PrOH = 4/1, flow rate 1.0 mL/min, detection at 290.0 nm, t_R = 13.9 min (minor), and 16.3 min (major).

N-((R,2E,4E)-1,5-diphenylpenta-2,4-dien-1-yl)-4-methylbenzenesulfonamide **(3ba)** (Known compound).[6]

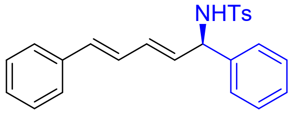

The reaction performed according to the standard procedure A afforded 27.6 mg (71% yield, *(E,E)/(E,Z)* > 20:1). Colorless semisolid.

^{1}H NMR (500 MHz, $CDCl_3$) δ 7.67 (d, *J* = 8.3 Hz, 2H), 7.38 – 7.28 (m, 5H), 7.25 (d, *J* = 7.1 Hz, 3H), 7.22 – 7.15 (m, 4H), 6.62 (dd, *J* = 15.6, 10.5 Hz, 1H), 6.42 (d, *J* = 15.7 Hz, 1H), 6.15 (dd, *J* = 15.2, 10.5 Hz, 1H), 5.74 (dd, *J* = 15.1, 6.7 Hz, 1H), 5.19 (d, *J* = 7.3 Hz, 1H), 5.04 (t, *J* = 6.9 Hz, 1H), 2.36 (s, 3H).

^{13}C NMR (126 MHz, $CDCl_3$) δ 143.4, 139.7, 137.7, 137.0, 133.7, 132.6, 132.2, 129.6, 128.8, 128.7, 127.9, 127.9, 127.6, 127.4, 127.2, 126.5, 59.7, 21.6.

Enantiomeric excess was determined to be 89% *ee* by chiral HPLC analysis (CHIRALPAK IA (*n*-hexane/^{i}PrOH = 19/1, flow rate 1.0 mL/min, detection at 290.0 nm, t_R = 35.8 min (minor), and 38.2 min (major).

4-methyl-*N-((S,2E,4E)*-1-phenyl-5-(p-tolyl)penta-2,4-dien-1-yl)benzenesulfonamide **(3ca)**

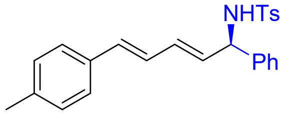

The reaction performed according to the standard procedure A afforded 33.4 mg (83% yield, *(E,E)/(E,Z)* > 20:1). Colorless semisolid.

^{1}H NMR (500 MHz, $CDCl_3$) δ 7.66 (d, *J* = 8.2 Hz, 2H), 7.27 – 7.22 (m, 5H), 7.20 (d, *J* = 8.2 Hz, 2H), 7.17 (dd, *J* = 7.5, 1.8 Hz, 2H), 7.12 (d, *J* = 8.0 Hz, 2H), 6.57 (dd, *J* = 15.6, 10.5 Hz, 1H), 6.40 (d, *J* = 15.6 Hz, 1H), 6.13 (dd, *J* = 15.1, 10.5 Hz, 1H), 5.71 (dd, *J* = 15.1, 5.9 Hz, 1H), 5.08 – 4.99 (m, 2H), 2.37 (s, 3H), 2.34 (s, 3H).

^{13}C NMR (126 MHz, $CDCl_3$) δ 143.4, 139.8, 137.9, 137.8, 134.2, 133.7, 132.8, 131.5, 129.6, 129.5, 128.8, 127.9, 127.4, 127.2, 126.7, 126.4, 59.7, 21.6, 21.4.

IR (thin film): 3271, 3031, 2920, 2357, 1168, 1601, 1503, 1447, 1325, 1157, 1090, 811, 756, 667, 553, 406 cm^{-1}.

HRMS (ESI): *m/z* calculated for $C_{25}H_{25}NO_2S$ $[M-H]^-$: 402.1533, found: 402.1527.

$[\alpha]_D^{23}$ -23.3 (*c* 1.0, $CHCl_3$, 91% *ee* sample).

Enantiomeric excess was determined to be 91% *ee* by chiral HPLC analysis (CHIRALPAK IA (*n*-hexane/^{i}PrOH = 4/1, flow rate 1.0 mL/min, detection at 272.8 nm, t_R = 10.6 min (minor), and 12.3 min (major).

4-methyl-*N-((R,2E,4E)*-1-phenyl-5-(m-tolyl)penta-2,4-dien-1-yl)benzenesulfonamide **(3da)**

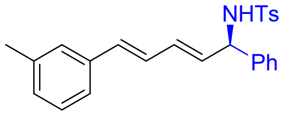

The reaction performed according to the standard procedure A afforded 28.2 mg (70% yield, *(E,E)/(E,Z)* > 20:1). Colourless liquid.

^{1}H NMR (500 MHz, $CDCl_3$) δ 7.68 (d, *J* = 8.2 Hz, 2H), 7.24 (t, *J* = 6.4 Hz, 3H), 7.18 (dt, *J* = 21.4, 6.1 Hz, 7H), 7.06 (d, *J* = 7.3 Hz, 1H), 6.61 (dd, *J* = 15.6, 10.5 Hz, 1H), 6.39 (d, *J* = 15.6 Hz, 1H), 6.14 (dd, *J* = 15.1, 10.5 Hz, 1H), 5.73 (dd, *J* = 15.1, 6.8 Hz, 1H), 5.33 (t, *J* = 14.2 Hz, 1H), 5.05 (t, *J* = 7.1 Hz, 1H), 2.36 (s, 3H), 2.35 (s, 3H).

^{13}C NMR (126 MHz, $CDCl_3$) δ 143.3, 139.8, 138.2, 137.8, 137.0, 133.7, 132.6, 132.0, 129.5, 128.7, 128.7, 128.6, 127.8, 127.5, 127.4, 127.2, 127.1, 123.7, 59.7, 21.6, 21.5.

HRMS (ESI): *m/z* calculated for $C_{25}H_{25}NO_2S$ $[M-H]^-$: 402.1533, found: 402.1537.

$[\alpha]_D^{23}$ -31.6 (*c* 1.0, $CHCl_3$, 89% *ee* sample).

Enantiomeric excess was determined to be 89% *ee* by chiral HPLC analysis (CHIRALPAK IA (*n*-hexane/^{i}PrOH = 19/1, flow rate 1.0 mL/min, detection at 254.0 nm, t_R = 39.1 min (minor), and 41.6 min (major).

N-((S,2E,4E)-5-(4-isopropylphenyl)-1-phenylpenta-2,4-dien-1-yl)-4-methylbenzenesulfonamide **(3ea)**

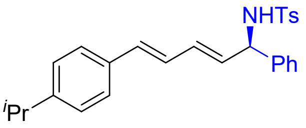

The reaction performed according to the standard procedure A afforded 30.6 mg (71% yield, *(E,E)/(E,Z)* > 20:1). Yellow liquid.

^{1}H NMR (500 MHz, CDCl$_3$) δ 7.67 (d, J = 8.2 Hz, 2H), 7.28 (d, J = 8.2 Hz, 2H), 7.24 (t, J = 6.6 Hz, 3H), 7.19 (dd, J = 7.9, 6.2 Hz, 6H), 6.58 (dd, J = 15.6, 10.5 Hz, 1H), 6.40 (d, J = 15.6 Hz, 1H), 6.13 (dd, J = 15.1, 10.5 Hz, 1H), 5.72 (dd, J = 15.1, 6.7 Hz, 1H), 5.24 (d, J = 7.3 Hz, 1H), 5.05 (t, J = 7.0 Hz, 1H), 2.91 (dt, J = 13.8, 6.9 Hz, 1H), 2.37 (s, 3H), 1.26 (d, J = 6.9 Hz, 6H).

^{13}C NMR (126 MHz, CDCl$_3$) δ 148.8, 143.3, 139.8, 137.8, 134.7, 133.7, 132.8, 131.6, 129.5, 128.8, 127.8, 127.4, 127.2, 126.8, 126.5, 59.7, 34.0, 24.0, 21.6.

IR (thin film): 3273, 3031, 2961, 2358, 1687, 1604, 1501, 1452, 1328, 1157, 1091, 1054, 983, 922, 817, 755, 699, 668, 556 cm^{-1}.

HRMS (ESI): *m/z* calculated for $C_{27}H_{29}NO_2S$ [M-H]$^-$: 430.1846, found: 430.1840.

$[\alpha]_D^{23}$ 60 (*c* 1.0, CHCl$_3$, 93% *ee* sample).

Enantiomeric excess was determined to be 93% *ee* by chiral HPLC analysis (CHIRALPAK IA (*n*-hexane/^{i}PrOH = 4/1, flow rate 1.0 mL/min, detection at 254.0 nm, t_R = 9.9 min (minor), and 12.4 min (major).

N-((R,2E,4E)-5-(4-(tert-butyl)phenyl)-1-phenylpenta-2,4-dien-1-yl)-4-methylbenzenesulfonamide **(3fa)**

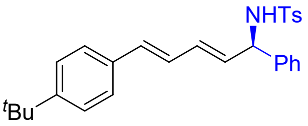

The reaction performed according to the standard procedure A afforded 33.4 mg (75% yield, *(E,E)/(E,Z)* > 20:1). Colourless liquid.

^{1}H NMR (500 MHz, CDCl$_3$) δ 7.66 (d, *J* = 8.2 Hz, 2H), 7.34 (d, *J* = 8.4 Hz, 2H), 7.29 (d, *J* = 8.4 Hz, 2H), 7.25 (d, *J* = 7.3 Hz, 3H), 7.22 – 7.16 (m, 4H), 6.59 (dd, *J* = 15.6, 10.5 Hz, 1H), 6.41 (d, *J* = 15.6 Hz, 1H), 6.13 (dd, *J* = 15.1, 10.5 Hz, 1H), 5.72 (dd, *J* = 15.1, 6.6 Hz, 1H), 5.09 (d, *J* = 7.1 Hz, 1H), 5.04 (t, *J* = 6.8 Hz, 1H), 2.38 (s, 3H), 1.33 (s, 9H).

^{13}C NMR (126 MHz, CDCl$_3$) δ 151.0, 143.6, 139.1, 137.6, 134.1, 133.5, 132.8, 131.4, 129.7, 129.4, 128.7, 127.8, 127.3, 127.0, 126.7, 126.4, 126.1, 125.6, 59.6, 34.6, 31.2, 21.5.

HRMS (ESI): *m/z* calculated for $C_{28}H_{31}NO_2S$ [M-H]$^-$: 444.2003, found: 444.2007.

$[\alpha]_D^{23}$ -21.0 (*c* 1.0, CHCl$_3$, 90% *ee* sample).

Enantiomeric excess was determined to be 90% *ee* by chiral HPLC analysis (CHIRALPAK IA (*n*-hexane/^{i}PrOH = 4/1, flow rate 1.0 mL/min, detection at 321.1 nm, t_R = 10.1 min (minor), and 11.9 min (major).

N-((S,2E,4E)-5-([1,1'-biphenyl]-4-yl)-1-phenylpenta-2,4-dien-1-yl)-4-methylbenzenesulfonamide **(3ga)** (Known compound).[6]

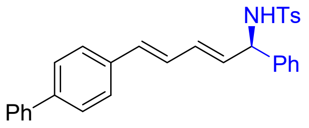

The reaction performed according to the standard procedure A afforded 41.4 mg (89% yield, *(E,E)/(E,Z)* > 20:1). Colorless semisolid.

^{1}H NMR (500 MHz, CDCl$_3$) δ 7.68 (d, *J* = 8.2 Hz, 2H), 7.61 (d, *J* = 7.3 Hz, 2H), 7.56 (d, *J* = 8.2 Hz, 2H), 7.44 (dd, *J* = 17.4, 7.9 Hz, 4H), 7.36 (t, *J* = 7.3 Hz, 1H), 7.25 (t, *J* = 6.3 Hz, 3H), 7.22 (d, *J* = 8.1 Hz, 2H), 7.20 – 7.16 (m, 2H), 6.67 (dd, *J* = 15.6, 10.5 Hz, 1H), 6.47 (d, *J* = 15.6 Hz, 1H), 6.19 (dd, *J* = 15.1, 10.5 Hz, 1H), 5.77 (dd, *J* = 15.1, 6.1 Hz, 1H), 5.07 (q, *J* = 7.0 Hz, 2H), 2.38 (s, 3H).

^{13}C NMR (126 MHz, CDCl$_3$) δ 143.4, 140.7, 140.6, 139.7, 137.8, 136.1, 133.2, 132.7, 132.3, 129.6, 128.9, 128.8, 128.0, 127.7, 127.5, 127.5, 127.4, 127.2, 127.0, 127.0, 59.7, 21.6.

IR (thin film): 3272, 3029, 2924, 2857, 2357, 1910, 1661, 1599, 1486, 1446, 1325, 1158, 1090, 1034, 989, 923, 819, 706, 695, 558 cm^{-1}.

HRMS (ESI): *m/z* calculated for $C_{30}H_{27}NO_2S$ [M-H]$^-$: 464.1690, found: 464.1682.

$[\alpha]_D^{23}$ +40 (*c* 1.0, CHCl$_3$, 97% *ee* sample).

Enantiomeric excess was determined to be 97% *ee* by chiral HPLC analysis (CHIRALPAK IA (*n*-hexane/^{i}PrOH = 4/1, flow rate 1.0 mL/min, detection at 278.5 nm, t_R = 16.8 min (minor), and 18.6 min (major).

N-((S,2E,4E)-5-(4-(benzyloxy)phenyl)-1-phenylpenta-2,4-dien-1-yl)-4-methylbenzenesulfonamide **(3ha)**

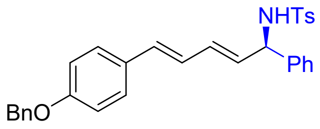

The reaction performed according to the standard procedure A afforded 43.6 mg (88% yield, *(E,E)/(E,Z)* > 20:1). White solid (m.p = 115–117 °C).

^{1}H NMR (500 MHz, CDCl$_3$) δ 7.66 (d, *J* = 8.2 Hz, 2H), 7.47 – 7.33 (m, 6H), 7.29 (d, *J* = 8.7 Hz, 2H), 7.25 (d, *J* = 7.2 Hz, 2H), 7.20 (d, *J* = 8.1 Hz, 2H), 7.18 – 7.14 (m, 2H), 6.93 (d, *J* = 8.7 Hz, 2H), 6.50 (dd, *J* = 15.6, 10.3 Hz, 1H), 6.38 (d, *J* = 15.6 Hz, 1H), 6.12 (dd, *J* = 15.1, 10.4 Hz, 1H), 5.68 (dd, *J* = 15.1, 6.7 Hz, 1H), 5.07 (s, 2H), 5.03 (t, *J* = 6.8 Hz, 1H), 4.96 (d, *J* = 7.1 Hz, 1H), 2.37 (s, 3H).Hz, 1H), 4.07 (q, *J* = 7.3 Hz, 1H), 3.83 (s, 3H), 3.34 (ddd, *J* = 24.1, 17.3, 7.3 Hz, 2H), 2.32 (s, 3H).

^{13}C NMR (126 MHz, CDCl$_3$) δ 158.7, 143.4, 139.9, 137.8, 136.9, 133.3, 132.9, 131.0, 130.1, 129.6, 128.8, 128.7, 128.2, 127.9, 127.8, 127.6, 127.4, 127.2, 125.7, 115.1, 70.2, 59.7, 21.6.

IR (thin film): 3852, 3740, 3358, 3262, 3032, 2922, 2357, 1652, 1600, 1508, 1452, 1302, 1243, 1158, 1094, 1024, 905, 814, 744, 693, 545, 420 cm^{-1}.

HRMS (ESI): *m/z* calculated for $C_{31}H_{29}NO_3S$ [M-H]$^-$: 494.1795, found: 494.1788.

$[\alpha]_D^{23}$ -16.7 (*c* 1.0, CHCl$_3$, 99% *ee* sample).

Enantiomeric excess was determined to be 99% *ee* by chiral HPLC analysis (CHIRALPAK IA (*n*-hexane/^{i}PrOH = 4/1, flow rate 1.0 mL/min, detection at 306.5 nm, t_R = 11.4 min (minor), and 15.4 min (major).

4-methyl-*N-((S,2E,4E)*-5-(4-(methylthio)phenyl)-1-phenylpenta-2,4-dien-1-yl)benzenesulfonamide **(3ia)**

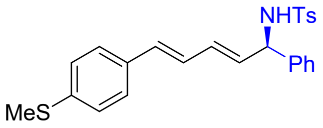

The reaction performed according to the standard procedure A afforded 33.1 mg (76% yield, *(E,E)/(E,Z)* = 15:1). Yellow solid (m.p = 130–132 °C).

^{1}H NMR (500 MHz, CDCl$_3$) δ 7.66 (d, *J* = 8.2 Hz, 2H), 7.27 – 7.22 (m, 5H), 7.19 (t, *J* = 7.4 Hz, 4H), 7.15 (dd, *J* = 7.4, 1.8 Hz, 2H), 6.58 (dd, *J* = 15.6, 10.5 Hz, 1H), 6.37 (d, *J* = 15.6 Hz, 1H), 6.14 (dd, *J* = 15.1, 10.5 Hz, 1H), 5.72 (dd, *J* = 15.1, 6.1 Hz, 1H), 5.04 (q, *J* = 7.1 Hz, 1H), 2.49 (s, 3H), 2.37 (s, 3H).

^{13}C NMR (126 MHz, CDCl$_3$) δ 143.4, 139.7, 138.2, 137.8, 133.9, 133.1, 132.6, 132.0, 129.6, 128.8, 127.9, 127.4, 127.2, 127.0, 126.9, 126.6, 59.7, 21.6, 15.8.

IR (thin film): 3852, 3740, 3612, 3271, 3026, 2921, 2357, 152, 1595, 1493, 1435, 1324, 1157, 1091, 1033, 988, 925, 814, 755, 670, 558 cm^{-1}.

HRMS (ESI): *m/z* calculated for $C_{25}H_{25}NO_2S_2$ [M-H]$^-$: 434.1254, found: 434.1247.

Enantiomeric excess was determined to be 93% *ee* by chiral HPLC analysis (CHIRALPAK IA (*n*-hexane/^{i}PrOH = 4/1, flow rate 1.0 mL/min, detection at 307.7 nm, t_R = 16.2 min (minor), and 19.7 min (major).

N-((S,2E,4E)-5-(3,4-dimethoxyphenyl)-1-phenylpenta-2,4-dien-1-yl)-4-methylbenzenesulfonamide **(3ja)**

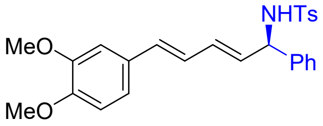

The reaction performed according to the standard procedure A afforded 33.7 mg (75% yield, *(E,E)/(E,Z)* > 20:1). Brown semisolid.

^{1}H NMR (500 MHz, CDCl$_3$) δ 7.65 (d, *J* = 8.2 Hz, 2H), 7.23 (d, *J* = 7.0 Hz, 3H), 7.19 (d, *J* = 8.1 Hz, 2H), 7.17 – 7.13 (m, 2H), 6.88 (d, *J* = 7.6 Hz, 2H), 6.81 (d, *J* = 8.2 Hz, 1H), 6.50 (dd, *J* = 15.5, 10.4 Hz, 1H), 6.36 (d, *J* = 15.6 Hz, 1H), 6.12 (dd, *J* = 15.1, 10.4 Hz, 1H), 5.71 (dd, *J* = 15.1, 6.7 Hz, 1H), 5.23 (d, *J* = 7.2 Hz, 1H), 5.02 (t, *J* = 7.0 Hz, 1H), 3.88 (d, *J* = 5.0 Hz, 6H), 2.36 (s, 3H).

^{13}C NMR (126 MHz, CDCl$_3$) δ 149.1, 143.3, 139.8, 137.7, 133.4, 132.7, 131.2, 130.1, 129.5, 128.7, 127.8, 127.4, 127.1, 125.9, 120.0, 111.2, 108.6, 59.7, 56.0, 55.9, 21.6.

IR (thin film): 3265, 2931, 2841, 2739, 2593, 2312, 1914, 1677, 1595, 1508, 1454, 1332, 1262, 1154, 1092, 1027, 909, 811, 669, 551 cm^{-1}.

HRMS (ESI): *m/z* calculated for $C_{26}H_{27}NO_4S$ [M-H]$^-$: 448.1588, found: 448.1581.

$[\alpha]_D^{23}$ +26.7 (*c* 1.0, $CHCl_3$, 98% *ee* sample).

Enantiomeric excess was determined to be 98% *ee* by chiral HPLC analysis (CHIRALPAK IA (*n*-hexane/^{i}PrOH = 4/1, flow rate 1.0 mL/min, detection at 306.5 nm, t_R = 22.8 min (minor), and 31.0 min (major).

N-((S,2E,4E)-5-(4-fluorophenyl)-1-phenylpenta-2,4-dien-1-yl)-4-methylbenzenesulfonamide **(3ka)**

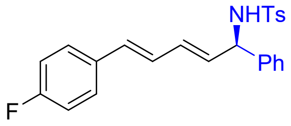

The reaction performed according to the standard procedure A afforded 35.4 mg (87% yield, *(E,E)/(E,Z)* > 20:1). Yellow oily liquid.

^{1}H NMR (500 MHz, CDCl$_3$) δ 7.67 (d, *J* = 8.1 Hz, 2H), 7.30 (dd, *J* = 8.4, 5.6 Hz, 2H), 7.23 (d, *J* = 6.6 Hz, 3H), 7.21 – 7.14 (m, 4H), 7.00 (t, *J* = 8.6 Hz, 2H), 6.53 (dd, *J* = 15.6, 10.4 Hz, 1H), 6.37 (d, *J* = 15.6 Hz, 1H), 6.14 (dd, *J* = 15.1, 10.4 Hz, 1H), 5.75 (dd, *J* = 15.1, 6.7 Hz, 1H), 5.37 (d, *J* = 7.4 Hz, 1H), 5.03 (t, *J* = 7.0 Hz, 1H), 2.35 (s, 3H).

^{13}C NMR (126 MHz, CDCl$_3$) δ 162.4, 143.3, 139.7, 137.7, 133.2, 132.3, 129.5, 128.8, 128.0, 127.9, 127.9, 127.4, 127.4, 127.1, 115.8, 115.6, 59.6, 21.6.
^{19}F NMR (471 MHz, CDCl$_3$) δ -113.82.
IR (thin film): 3489, 3036, 2919, 2739, 2362, 1902, 1691, 1598, 1503, 1445, 1330, 1228, 1156, 1090, 984, 923, 822, 745, 669, 555 cm^{-1}.
HRMS (ESI): *m/z* calculated for $C_{24}H_{22}FNO_2S$ [M-H]$^-$: 406.1283, found: 406.1277.

$[\alpha]_D^{23}$ -76.7 (*c* 1.0, $CHCl_3$, 97% *ee* sample).

Enantiomeric excess was determined to be 97% *ee* by chiral HPLC analysis (CHIRALPAK IA (*n*-hexane/^{i}PrOH = 9/1, flow rate 1.0 mL/min, detection at 283.0 nm, t_R = 25.6 min (minor), and 27.0 min (major).

N-((S,2E,4E)-5-(4-chlorophenyl)-1-phenylpenta-2,4-dien-1-yl)-4-methylbenzenesulfonamide **(3la)**

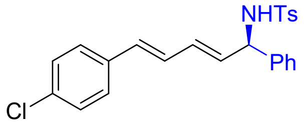

The reaction performed according to the standard procedure A afforded 28.8 mg (68% yield, *(E,E)/(E,Z)* > 20:1). Yellow liquid.
^{1}H NMR (500 MHz, CDCl$_3$) δ 7.66 (d, *J* = 8.2 Hz, 2H), 7.27 – 7.22 (m, 7H), 7.20 (d, *J* = 8.1 Hz, 2H), 7.14 (dd, *J* = 7.3, 2.0 Hz, 2H), 6.60 (dd, *J* = 15.6, 10.5 Hz, 1H), 6.38 (d, *J* = 15.6 Hz, 1H), 6.21 – 6.10 (m, 1H), 5.77 (dd, *J* = 15.1, 6.5 Hz, 1H), 5.07 – 4.99 (m, 1H), 4.97 (d, *J* = 7.0 Hz, 1H), 2.37 (s, 3H).
^{13}C NMR (126 MHz, CDCl$_3$) δ 143.5, 139.6, 137.7, 135.5, 133.5, 132.9, 132.3, 129.6, 129.0, 128.9, 128.2, 128.0, 127.7, 127.4, 127.2, 59.6, 21.6.
IR (thin film): 3272, 3032, 2919, 2358, 1908, 1684, 1595, 1490, 1412, 1327, 1157, 1091, 984, 926, 817, 755, 697, 670, 556 cm^{-1}.
HRMS (ESI): *m/z* calculated for $C_{24}H_{22}ClNO_2S$ [M-H]$^-$: 422.0987, found: 422.0982.

$[\alpha]_D^{23}$ -46.7 (*c* 1.0, $CHCl_3$, 85% *ee* sample).

Enantiomeric excess was determined to be 89% *ee* by chiral HPLC analysis (CHIRALPAK IA (*n*-hexane/^{i}PrOH = 4/1, flow rate 1.0 mL/min, detection at 278.0 nm, t_R = 13.7 min (minor), and 15.0 min (major).

4-methyl-*N-((S,2E,4E)*-1-phenyl-5-(4-(trifluoromethyl)phenyl)penta-2,4-dien-1-yl)benzenesulfonamide **(3ma)**

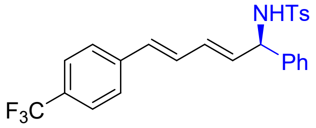

The reaction performed according to the standard procedure A afforded 33.8 mg (74% yield, *(E,E)/(E,Z)* > 20:1). Yellow solid (m.p = 110–112 °C).
^{1}H NMR (500 MHz, CDCl$_3$) δ 7.67 (d, *J* = 8.2 Hz, 2H), 7.55 (d, *J* = 8.2 Hz, 2H), 7.42 (d, *J* = 8.2 Hz, 2H), 7.26 – 7.22 (m, 3H), 7.20 (d, *J* = 8.0 Hz, 2H), 7.14 (dd, *J* = 7.1, 2.0 Hz, 2H), 6.71 (dd, *J* = 15.6, 10.5 Hz, 1H), 6.45 (d, *J* = 15.7 Hz, 1H), 6.21 (dd, *J* = 15.2, 10.6 Hz, 1H), 5.85 (dd, *J* = 15.2, 6.5 Hz, 1H), 5.19 (d, *J* = 7.2 Hz, 1H), 5.05 (t, *J* = 6.8 Hz, 1H), 2.37 (s, 3H).
^{13}C NMR (126 MHz, CDCl$_3$) δ 143.5, 139.5, 137.7, 134.1, 131.9, 131.9, 130.1, 129.6, 128.9, 128.1, 127.4, 127.2, 126.6, 125.7, 125.7, 59.6, 21.6.
^{19}F NMR (471 MHz, CDCl$_3$) δ -62.5.
IR (thin film): 3271, 3033, 2924, 2357, 1610, 1495, 1418, 1325, 1160, 1116, 991, 926, 823, 756,

673, 558 cm^{-1}.

HRMS (ESI): *m/z* calculated for $C_{25}H_{22}F_3NO_2S$ [M-H]$^-$: 456.1251, found: 456.1245.

$[\alpha]_D^{23}$ -43.3 (*c* 1.0, $CHCl_3$, 94% *ee* sample).

Enantiomeric excess was determined to be 94% *ee* by chiral HPLC analysis (CHIRALPAK IA (*n*-hexane/^{i}PrOH = 4/1, flow rate 1.0 mL/min, detection at 295.5 nm, t_R = 12.8 min (minor), and 13.6 min (major).

N-((S,2E,4E)-5-(benzofuran-5-yl)-1-phenylpenta-2,4-dien-1-yl)-4-methylbenzenesulfonamide **(3na)**

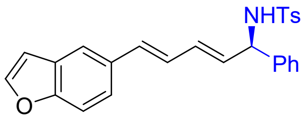

The reaction performed according to the standard procedure A afforded 40.8 mg (95% yield, *(E,E)/(E,Z)* > 20:1). Yellow solid (m.p = 123–125 °C).

^{1}H NMR (500 MHz, $CDCl_3$) δ 7.68 (d, *J* = 8.1 Hz, 2H), 7.62 – 7.52 (m, 2H), 7.43 (d, *J* = 8.6 Hz, 1H), 7.32 (d, *J* = 8.6 Hz, 1H), 7.24 (q, *J* = 6.2 Hz, 3H), 7.19 (t, *J* = 6.7 Hz, 4H), 6.74 (d, *J* = 1.1 Hz, 1H), 6.60 (dd, *J* = 15.6, 10.2 Hz, 1H), 6.51 (d, *J* = 15.6 Hz, 1H), 6.16 (dd, *J* = 15.1, 10.2 Hz, 1H), 5.73 (dd, *J* = 15.1, 6.7 Hz, 1H), 5.30 (d, *J* = 7.3 Hz, 1H), 5.05 (t, *J* = 7.0 Hz, 1H), 2.35 (s, 3H).

^{13}C NMR (126 MHz, $CDCl_3$) δ 154.8, 145.7, 143.4, 139.8, 137.7, 133.9, 132.7, 132.2, 131.5, 129.5, 128.8, 127.9, 127.9, 127.4, 127.2, 126.7, 122.9, 119.3, 111.6, 106.8, 59.7, 21.6.

IR (thin film): 3852, 3740, 3272, 3027, 2921, 2357, 1599, 1452, 1324, 1202, 1157, 1094 ,1031, 988, 927, 812, 757, 670, 556, 428 cm^{-1}.

HRMS (ESI): *m/z* calculated for $C_{26}H_{23}NO_3S$ [M-H]$^-$: 428.1326, found: 428.1318.

$[\alpha]_D^{23}$ -43.3 (*c* 1.0, $CHCl_3$, 90% *ee* sample).

Enantiomeric excess was determined to be 90% *ee* by chiral HPLC analysis (CHIRALPAK IA (*n*-hexane/^{i}PrOH = 4/1, flow rate 1.0 mL/min, detection at 329.1 nm, t_R = 17.2 min (minor), and 19.2 min (major).

4-methyl-*N-((S,2E,4E)*-1-phenyl-5-(thiophen-2-yl)penta-2,4-dien-1-yl)benzenesulfonamide **(3oa)**

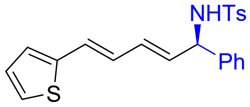

The reaction performed according to the standard procedure A afforded 38.7 mg (98% yield, *(E,E)/(E,Z)* > 20:1). Brown solid (m.p = 108–110 °C).

^{1}H NMR (500 MHz, $CDCl_3$) δ 7.66 (d, *J* = 8.3 Hz, 2H), 7.26 (dd, *J* = 4.4, 2.3 Hz, 1H), 7.23 (d, *J* = 7.1 Hz, 2H), 7.20 – 7.14 (m, 5H), 7.13 (dd, *J* = 2.9, 1.1 Hz, 1H), 6.44 (d, *J* = 8.2 Hz, 1H), 6.09 (dd, *J* = 15.3, 8.0 Hz, 1H), 5.70 (dd, *J* = 15.1, 6.7 Hz, 1H), 5.30 (d, *J* = 7.4 Hz, 1H), 5.02 (t, *J* = 7.0 Hz, 1H), 2.36 (s, 3H).

^{13}C NMR (126 MHz, $CDCl_3$) δ 143.3, 139.8, 139.8, 137.7, 132.5, 131.7, 129.5, 128.7, 127.8, 127.7, 127.7, 127.4, 127.1, 126.3, 124.9, 122.6, 59.6, 21.6.

IR (thin film): 3272, 3029, 2921, 2357, 1646, 1598, 1494, 1438, 1326, 1158, 1090, 1034, 987, 924, 810, 764, 673, 621, 555 cm^{-1}.

HRMS (ESI): *m/z* calculated for $C_{22}H_{21}NO_2S_2$ [M-H]$^-$: 394.0941, found: 394.0934.

$[\alpha]_D^{23}$ -93.3 (*c* 1.0, $CHCl_3$, 92% *ee* sample).

Enantiomeric excess was determined to be 92% *ee* by chiral HPLC analysis (CHIRALPAK IA (*n*-hexane/^{i}PrOH = 4/1, flow rate 1.0 mL/min, detection at 254.0 nm, t_R = 13.8 min (minor), and 15.6 min (major).

4-methyl-*N-((S,2E,4E)*-1-phenyl-5-(Ferroceneyl)penta-2,4-dien-1-yl)benzenesulfonamide **(3pa)**

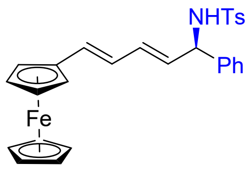

The reaction performed according to the standard procedure A afforded 30.8 mg (62% yield, *(E,E)/(E,Z)* > 20:1). Brownish yellow liquid.

^{1}H NMR (500 MHz, CDCl$_3$) δ 7.68 (d, *J* = 7.2 Hz, 2H), 7.33 – 7.11 (m, 7H), 6.23 (q, *J* = 15.5 Hz, 2H), 6.12 – 5.97 (m, 1H), 5.62 (dd, *J* = 14.7, 6.1 Hz, 1H), 5.32 (d, *J* = 6.1 Hz, 1H), 5.02 (s, 1H), 4.30 (d, *J* = 35.1 Hz, 4H), 4.12 (s, 6H), 2.40 (s, 3H).

^{13}C NMR (126 MHz, CDCl$_3$) δ 143.2, 139.9, 137.8, 132.9, 132.2, 129.5, 129.3, 128.7, 127.7, 127.3, 127.1, 125.4, 82.7, 69.3, 69.2, 69.2, 67.1, 66.8, 59.8, 21.6.

IR (thin film): 3273, 3089, 3028, 2921, 2357, 1601, 1446, 1325, 1157, 1092, 1034, 988, 927, 815, 755, 669, 557, 489, 406 cm^{-1}.

HRMS (ESI): *m/z* calculated for $C_{28}H_{27}FeNO_2S$ [M-H]$^-$: 496.1039, found: 496.1030.

$[\alpha]_D^{23}$ -10.0 (*c* 1.0, $CHCl_3$, 86% *ee* sample).

Enantiomeric excess was determined to be 86% *ee* by chiral HPLC analysis (CHIRALPAK IA (*n*-hexane/^{i}PrOH = 4/1, flow rate 1.0 mL/min, detection at 290.0 nm, t_R = 12.1 min (minor), and 16.6 min (major).

N-((R,2E,4Z)-5-(6-(3-(adamantan-1-yl)-4-methoxyphenyl)naphthalen-2-yl)-1-phenylpenta-2,4-dien-1-yl)-4-methylbenzenesulfonamide **(3qa)**

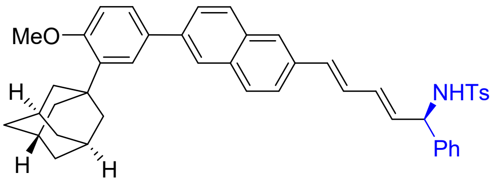

The reaction performed according to the standard procedure A afforded 41.4 mg (61% yield, *(E,E)/(E,Z)* > 20:1). Yellow solid (m.p = 159–161 °C).

^{1}H NMR (500 MHz, CDCl$_3$) δ 7.94 (s, 1H), 7.86 – 7.79 (m, 3H), 7.74 – 7.66 (m, 4H), 7.62 – 7.51 (m, 3H), 7.32 (d, J = 8.1 Hz, 1H), 7.26 – 7.17 (m, 5H), 7.00 (d, J = 8.5 Hz, 1H), 6.75 (dd, J = 15.5, 10.4 Hz, 1H), 6.60 (d, J = 15.6 Hz, 1H), 6.22 (dd, J = 15.0, 10.5 Hz, 1H), 5.78 (dd, J = 15.1, 6.6 Hz, 1H), 5.07 (t, J = 6.7 Hz, 1H), 5.02 (d, J = 7.1 Hz, 1H), 3.91 (s, 3H), 2.38 (s, 3H), 2.19 (s, 9H), 1.81 (s, 6H).

^{13}C NMR (126 MHz, CDCl$_3$) δ 158.7, 143.7, 143.5, 139.8, 139.3, 139.2, 139.0, 137.7, 134.2, 133.9, 133.6, 133.1, 132.8, 132.5, 132.1, 129.9, 129.6, 128.9, 128.6, 128.5, 128.0, 127.7, 127.5, 127.2, 126.6, 126.5, 126.3, 126.0, 125.7, 124.9, 123.7, 112.2, 59.7, 55.3, 40.7, 37.3, 37.3, 29.2, 21.7.

HRMS (ESI): *m/z* calculated for $C_{45}H_{45}NO_3S$ [M-H]$^-$: 678.3047, found: 678.3051.

$[\alpha]_D^{23}$ -10.5 (*c* 1.0, $CHCl_3$, 71% *ee* sample).

Enantiomeric excess was determined to be 71% *ee* by chiral HPLC analysis (CHIRALPAK IA (*n*-hexane/^{i}PrOH = 4/1, flow rate 1.0 mL/min, detection at 321.1 nm, t_R = 20.1 min (minor), and 23.5 min (major).

4-methyl-*N-((S,2E,4E)*-5-(naphthalen-2-yl)-1-(m-tolyl)penta-2,4-dien-1-yl)benzenesulfonamide **(3ab)**

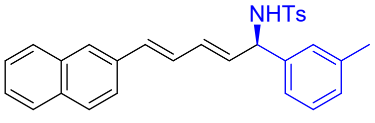

The reaction performed according to the standard procedure A afforded 43.0 mg (95% yield, *(E,E)/(E,Z)* > 20:1). Light yellow solid (m.p = 153–155 °C).

^{1}H NMR (500 MHz, CDCl$_3$) δ 7.78 (dd, *J* = 11.4, 8.7 Hz, 3H), 7.72 – 7.65 (m, 3H), 7.56 (dd, *J* = 8.6, 1.6 Hz, 1H), 7.50 – 7.42 (m, 2H), 7.21 (d, *J* = 8.0 Hz, 2H), 7.15 (t, *J* = 7.6 Hz, 1H), 7.04 (d, *J* = 7.5 Hz, 1H), 6.97 (d, *J* = 7.7 Hz, 1H), 6.91 (s, 1H), 6.76 (dd, *J* = 15.5, 10.5 Hz, 1H), 6.61 (d, *J* = 15.6 Hz, 1H), 6.24 (dd, *J* = 15.3, 10.2 Hz, 1H), 5.79 (dd, *J* = 15.1, 6.6 Hz, 1H), 5.03 (t, *J* = 6.7 Hz, 1H), 4.97 (d, *J* = 7.1 Hz, 1H), 2.37 (s, 3H), 2.26 (s, 3H).

^{13}C NMR (126 MHz, CDCl$_3$) δ 143.4, 139.6, 138.5, 137.8, 134.6, 133.7, 133.2, 132.5, 132.5, 129.5, 128.8, 128.7, 128.4, 128.1, 128.1, 127.9, 127.8, 127.5, 126.7, 126.5, 126.1, 124.2, 123.4, 59.7, 21.6, 21.4.

IR (thin film): 3842, 3740, 3612, 3273, 3030, 2916, 2357, 1601, 1501, 1433, 1324, 1156, 1090, 1037, 987, 906, 810, 749, 668, 559, 475 cm^{-1}.

HRMS (ESI): *m/z* calculated for $C_{29}H_{27}NO_2S$ [M-H]$^-$: 452.1690, found: 452.1684.

Enantiomeric excess was determined to be 97% *ee* by chiral HPLC analysis (CHIRALPAK IA (*n*-hexane/^{i}PrOH = 4/1, flow rate 1.0 mL/min, detection at 306.3 nm, t_R = 11.9 min (minor), and 13.1 min (major).

4-methyl-*N-((R,2E,4E)*-5-(naphthalen-2-yl)-1-(p-tolyl)penta-2,4-dien-1-yl)benzenesulfonamide **(3ac)**

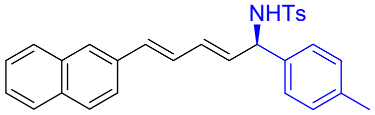

The reaction performed according to the standard procedure A afforded 38.1 mg (84% yield, *(E,E)/(E,Z)* > 20:1). Colourless liquid.

^{1}H NMR (500 MHz, CDCl$_3$) δ 7.78 (dd, *J* = 11.9, 8.7 Hz, 3H), 7.69 (d, *J* = 8.1 Hz, 3H), 7.55 (d, *J* = 8.6 Hz, 1H), 7.46 (td, *J* = 12.8, 6.3 Hz, 2H), 7.22 (d, *J* = 8.0 Hz, 2H), 7.07 (s, 4H), 6.75 (dd, *J* = 15.6, 10.4 Hz, 1H), 6.59 (d, *J* = 15.6 Hz, 1H), 6.22 (dd, *J* = 15.1, 10.5 Hz, 1H), 5.78 (dd, *J* = 15.1, 6.5 Hz, 1H), 5.08 (d, *J* = 7.1 Hz, 1H), 5.02 (t, *J* = 6.8 Hz, 1H), 2.37 (s, 3H), 2.32 (s, 3H).

^{13}C NMR (126 MHz, CDCl$_3$) δ 143.4, 137.8, 137.8, 136.8, 134.6, 133.7, 133.6, 133.1, 132.6, 132.5, 129.6, 129.5, 128.4, 128.1, 127.8, 127.5, 127.1, 126.7, 126.5, 126.1, 123.4, 59.5, 21.6, 21.2.

HRMS (ESI): *m/z* calculated for $C_{29}H_{27}NO_2S$ [M-H]$^-$: 452.1690, found: 452.1694.

$[\alpha]_D^{23}$ -10.5 (*c* 1.0, $CHCl_3$, 96% *ee* sample).

Enantiomeric excess was determined to be 96% *ee* by chiral HPLC analysis (CHIRALPAK IA (*n*-hexane/^{i}PrOH = 4/1, flow rate 1.0 mL/min, detection at 321.1 nm, t_R = 15.6 min (minor), and 18.2 min (major).

N-((S,2E,4E)-1-(4-ethylphenyl)-5-(naphthalen-2-yl)penta-2,4-dien-1-yl)-4-methylbenzenesulfonamide **(3ad)**

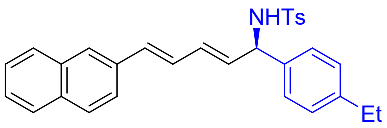

The reaction performed according to the standard procedure A afforded 37.4 mg (80% yield, *(E,E)/(E,Z)* > 20:1). Yellow solid (m.p = 157–159 °C).

^{1}H NMR (500 MHz, CDCl$_3$) δ 7.79 (dd, *J* = 13.0, 8.9 Hz, 3H), 7.69 (dd, *J* = 5.4, 2.7 Hz, 3H), 7.56 (dd, *J* = 8.6, 1.5 Hz, 1H), 7.51 – 7.44 (m, 2H), 7.19 (d, *J* = 8.2 Hz, 2H), 7.09 (q, *J* = 8.5 Hz, 4H), 6.75 (dd, *J* = 15.6, 10.4 Hz, 1H), 6.58 (d, *J* = 15.6 Hz, 1H), 6.24 (dd, *J* = 15.1, 10.5 Hz, 1H), 5.80 (dd, *J* = 15.1, 6.7 Hz, 1H), 5.43 (dd, *J* = 47.4, 7.9 Hz, 1H), 5.06 (t, *J* = 7.0 Hz, 1H), 2.61 (q, *J* = 7.6 Hz, 2H), 2.35 (s, 3H), 1.22 (t, *J* = 7.6 Hz, 3H).
^{13}C NMR (126 MHz, CDCl$_3$) δ 144.0, 143.2, 137.8, 136.9, 134.6, 133.7, 133.5, 133.1, 132.6, 132.3, 129.5, 128.3, 128.2, 128.1, 128.1, 127.8, 127.4, 127.1, 126.6, 126.4, 126.1, 123.4, 59.5, 28.6, 21.5, 15.7.
IR (thin film): 3273, 3039, 2964, 2357, 1913, 1599, 1507, 1433, 1326, 1158, 1091, 1380, 988, 915, 816, 748, 668, 557, 477 cm^{-1}.
HRMS (ESI): *m/z* calculated for $C_{30}H_{29}NO_2S$ [M-H]$^-$: 466.1846, found: 466.1838.

$[\alpha]_D^{23}$ +13.3 (*c* 1.0, CHCl$_3$, 95% *ee* sample).

Enantiomeric excess was determined to be 95% *ee* by chiral HPLC analysis (CHIRALPAK IA (*n*-hexane/^{i}PrOH = 4/1, flow rate 1.0 mL/min, detection at 319.0 nm, t_R = 14.2 min (minor), and 17.2 min (major).

N-((S,2E,4E)-1-(4-isopropylphenyl)-5-(naphthalen-2-yl)penta-2,4-dien-1-yl)-4-methylbenzenesulfonamide **(3ae)**

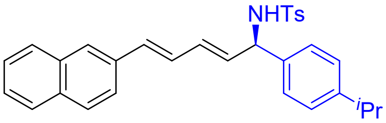

The reaction performed according to the standard procedure A afforded 42.8 mg (89% yield, *(E,E)/(E,Z)* > 20:1). Yellow solid (m.p = 149–153 °C).
^{1}H NMR (500 MHz, CDCl$_3$) δ 7.79 (dd, *J* = 12.3, 8.8 Hz, 3H), 7.72 – 7.64 (m, 3H), 7.56 (d, *J* = 9.9 Hz, 1H), 7.46 (dq, *J* = 13.8, 6.8 Hz, 2H), 7.19 (d, *J* = 8.0 Hz, 2H), 7.10 (s, 4H), 6.75 (dd, *J* = 15.6, 10.4 Hz, 1H), 6.59 (d, *J* = 15.6 Hz, 1H), 6.25 (dd, *J* = 15.1, 10.5 Hz, 1H), 5.81 (dd, *J* = 15.1, 6.7 Hz, 1H), 5.24 (d, *J* = 7.2 Hz, 1H), 5.06 (t, *J* = 6.9 Hz, 1H), 2.87 (dt, *J* = 13.8, 6.9 Hz, 1H), 2.35 (s, 3H), 1.23 (d, *J* = 6.9 Hz, 6H).
^{13}C NMR (126 MHz, CDCl$_3$) δ 148.6, 143.2, 137.8, 137.0, 134.6, 133.7, 133.5, 133.1, 132.6, 132.3, 129.5, 128.4, 128.1, 128.1, 127.8, 127.4, 127.1, 126.8, 126.6, 126.5, 126.1, 123.4, 59.5, 33.9, 24.0, 21.6.
IR (thin film): 3851, 3740, 3271, 3030, 2959, 2357, 1599, 1507, 1427, 1323, 1156, 1090, 1038, 987, 919, 814, 747, 667, 559, 474 cm^{-1}.
HRMS (ESI): *m/z* calculated for $C_{31}H_{31}NO_2S$ [M-H]$^-$: 480.2003, found: 480.1996.
Enantiomeric excess was determined to be 90% *ee* by chiral HPLC analysis (CHIRALPAK IA (*n*-hexane/^{i}PrOH = 4/1, flow rate 1.0 mL/min, detection at 254.0 nm, t_R = 11.9 min (minor), and 15.1 min (major).

N-((S,2E,4E)-1-(4-(tert-butyl)phenyl)-5-(naphthalen-2-yl)penta-2,4-dien-1-yl)-4-methylbenzenesulfonamide **(3af)**

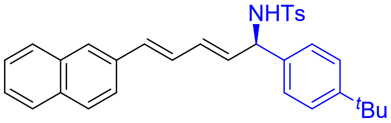

The reaction performed according to the standard procedure A afforded 40.6 mg (82% yield, *(E,E)/(E,Z)* = 14:1). Brown semisolid.
^{1}H NMR (500 MHz, CDCl$_3$) δ 7.81 – 7.76 (m, 3H), 7.72 – 7.63 (m, 3H), 7.56 (d, *J* = 8.0 Hz, 1H), 7.49 – 7.42 (m, 2H), 7.26 (d, *J* = 8.4 Hz, 2H), 7.19 (d, *J* =

8.0 Hz, 2H), 7.09 (d, J = 8.3 Hz, 2H), 6.76 (dd, J = 15.6, 10.4 Hz, 1H), 6.61 (d, J = 15.6 Hz, 1H), 6.25 (dd, J = 15.1, 10.5 Hz, 1H), 5.81 (dd, J = 15.1, 6.6 Hz, 1H), 5.06 (t, J = 6.8 Hz, 1H), 4.99 (d, J = 7.0 Hz, 1H), 2.36 (s, 3H), 1.29 (s, 9H).

^{13}C NMR (126 MHz, CDCl$_3$) δ 151.0, 143.2, 137.8, 136.6, 134.6, 133.7, 133.7, 133.2, 132.6, 132.5, 129.5, 128.4, 128.1, 127.8, 127.5, 126.9, 126.7, 126.5, 126.1, 125.7, 123.4, 59.5, 34.6, 31.4, 21.6.

IR (thin film): 3272, 3052, 2959, 2358, 1687, 1601, 1506, 1430, 1328, 1157, 1092, 1029, 986, 917, 817, 749, 667, 571, 476 cm^{-1}.

HRMS (ESI): *m/z* calculated for $C_{32}H_{33}NO_2S$ [M-H]$^-$: 494.2159, found: 494.2151.

$[\alpha]_D^{23}$ -20.0 (*c* 1.0, CHCl$_3$, 96% *ee* sample).

Enantiomeric excess was determined to be 96% *ee* by chiral HPLC analysis (CHIRALPAK IA (*n*-hexane/^{i}PrOH = 4/1, flow rate 1.0 mL/min, detection at 265.0 nm, t_R = 10.7 min (minor), and 13.4 min (major).

N-((S,2E,4E)-1-(4-methoxyphenyl)-5-(naphthalen-2-yl)penta-2,4-dien-1-yl)-4-methylbenzenesulfonamide **(3ag)**

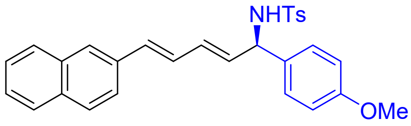

The reaction performed according to the standard procedure A afforded 32.4 mg (69% yield, *(E,E)/(E,Z)* > 20:1). White solid (m.p = 135–137 °C).

^{1}H NMR (500 MHz, CDCl$_3$) δ 7.83 – 7.74 (m, 3H), 7.69 (d, J = 7.7 Hz, 3H), 7.55 (d, J = 8.5 Hz, 1H), 7.50 – 7.42 (m, 2H), 7.21 (d, J = 7.9 Hz, 2H), 7.09 (d, J = 8.4 Hz, 2H), 6.78 (d, J = 8.3 Hz, 2H), 6.76 – 6.70 (m, 1H), 6.58 (d, J = 15.6 Hz, 1H), 6.21 (dd, J = 14.9, 10.6 Hz, 1H), 5.78 (dd, J = 15.1, 6.5 Hz, 1H), 5.20 (d, J = 7.1 Hz, 1H), 5.02 (t, J = 6.8 Hz, 1H), 3.77 (s, 3H), 2.36 (s, 3H).

^{13}C NMR (126 MHz, CDCl$_3$) δ 159.3, 143.3, 137.8, 134.6, 133.7, 133.6, 133.1, 132.6, 132.3, 131.9, 129.5, 128.4, 128.4, 128.1, 128.1, 127.8, 127.4, 126.6, 126.5, 126.1, 123.4, 114.1, 59.1, 55.4, 21.6.

IR (thin film): 3853, 3739, 3272, 3034, 2925, 2357, 1601, 1509, 1438, 1324, 1249, 1159, 1091, 1031, 989, 910, 815, 750, 668, 552, 475 cm^{-1}.

HRMS (ESI): *m/z* calculated for $C_{29}H_{27}NO_3S$ [M-H]$^-$: 468.1639, found: 468.1630.

$[\alpha]_D^{23}$ -10.0 (*c* 1.0, CHCl$_3$, 94% *ee* sample).

Enantiomeric excess was determined to be 94% *ee* by chiral HPLC analysis (CHIRALPAK IA (*n*-hexane/^{i}PrOH = 4/1, flow rate 1.0 mL/min, detection at 273.6 nm, t_R = 18.5 min (minor), and 22.3 min (major).

N-((R,2E,4E)-1-(2-methoxyphenyl)-5-(naphthalen-2-yl)penta-2,4-dien-1-yl)-4-methylbenzenesulfonamide **(3ah)**

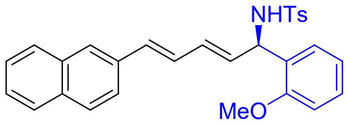

The reaction performed according to the standard procedure A afforded 45.0 mg (96% yield, *(E,E)/(E,Z)* > 20:1). Yellow solid (m.p = 159–161 °C).

^{1}H NMR (500 MHz, CDCl$_3$) δ 7.81 – 7.76 (m, 3H), 7.72 – 7.65 (m, 3H), 7.56 (dd, J = 8.6, 1.4 Hz, 1H), 7.50 – 7.42 (m, 2H), 7.22 (d, J = 8.1 Hz, 2H), 7.09 (d, J =

8.7 Hz, 2H), 6.79 (d, J = 8.7 Hz, 2H), 6.75 (dd, J = 13.5, 8.3 Hz, 1H), 6.60 (d, J = 15.6 Hz, 1H), 6.22 (dd, J = 15.1, 10.5 Hz, 1H), 5.78 (dd, J = 15.1, 6.6 Hz, 1H), 5.02 (t, J = 6.7 Hz, 1H), 4.92 (d, J = 6.9 Hz, 1H), 3.78 (s, 3H), 2.38 (s, 3H).

^{13}C NMR (126 MHz, CDCl$_3$) δ 159.34, 143.39, 137.83, 134.58, 133.72, 133.68, 133.17, 132.58, 132.47, 131.85, 129.58, 128.44, 128.40, 128.11, 128.07, 127.80, 127.48, 126.68, 126.50, 126.13, 123.41, 114.19, 59.17, 55.43, 21.63.

IR (thin film): 3851, 3739, 3617, 3279, 3029, 2923, 2357, 1661, 1595, 1493, 1451, 1327, 1245, 1157, 1090, 1029, 984, 812, 751, 669, 556, 474 cm^{-1}.

HRMS (ESI): *m/z* calculated for $C_{29}H_{27}NO_3S$ [M-H]$^-$: 468.1639, found: 468.1631.

$[\alpha]_D^{23}$ -116.7 (*c* 1.0, CHCl$_3$, 96% *ee* sample).

Enantiomeric excess was determined to be 96% *ee* by chiral HPLC analysis (CHIRALPAK IA (*n*-hexane/^{i}PrOH = 9/1, flow rate 1.0 mL/min, detection at 305.0 nm, t_R = 15.7 min (major), and 17.3 min (minor).

N-((S,2E,4E)-1,5-di(naphthalen-2-yl)penta-2,4-dien-1-yl)-4-methylbenzenesulfonamide **(3ai)**

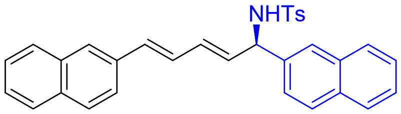

The reaction performed according to the standard procedure A afforded 36.7 mg (75% yield, *(E,E)/(E,Z)* > 20:1). White solid (m.p = 146–148 °C).

^{1}H NMR (500 MHz, CDCl$_3$) δ 7.79 (d, *J* = 8.3 Hz, 4H), 7.71 (d, *J* = 10.5 Hz, 3H), 7.64 (d, *J* = 8.2 Hz, 2H), 7.56 (d, *J* = 8.2 Hz, 2H), 7.51 – 7.45 (m, 4H), 7.26 (d, *J* = 1.7 Hz, 1H), 7.10 (d, *J* = 8.0 Hz, 2H), 6.79 (dd, *J* = 15.6, 10.5 Hz, 1H), 6.61 (d, *J* = 15.6 Hz, 1H), 6.27 (dd, *J* = 15.0, 10.6 Hz, 1H), 5.89 (dd, *J* = 15.2, 6.5 Hz, 1H), 5.24 (t, *J* = 6.8 Hz, 1H), 5.02 (d, *J* = 7.2 Hz, 1H), 2.27 (s, 3H).

^{13}C NMR (126 MHz, CDCl$_3$) δ 143.5, 137.7, 136.8, 134.5, 134.0, 133.7, 133.3, 133.2, 133.1, 133.0, 132.2, 129.5, 128.8, 128.4, 128.1, 128.1, 128.0, 127.8, 127.7, 127.5, 126.8, 126.5, 126.5, 126.4, 126.3, 126.2, 125.0, 123.4, 59.8, 21.5.

IR (thin film): 3852, 3740, 3272, 3050, 2922, 2357, 1917, 1597, 1504, 1426, 1325, 1157, 1089, 1037, 988, 858, 904, 814, 749, 668, 554, 476 cm^{-1}.

HRMS (ESI): *m/z* calculated for $C_{32}H_{27}NO_2S$ [M-H]$^-$: 488.1690, found: 488.1683.

$[\alpha]_D^{23}$ -23.3 (*c* 1.0, CHCl$_3$, 87% *ee* sample).

Enantiomeric excess was determined to be 87% *ee* by chiral HPLC analysis (CHIRALPAK IA (*n*-hexane/^{i}PrOH = 9/1, flow rate 1.0 mL/min, detection at 304.0 nm, t_R = 41.1 min (minor), and 44.9 min (major).

4-methyl-*N-((R,2E,4E)*-5-(naphthalen-2-yl)-1-(4-(trifluoromethyl)phenyl)penta-2,4-dien-1-yl)benzenesulfonamide **(3aj)**

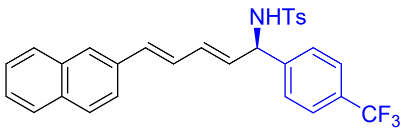

The reaction performed according to the standard procedure A afforded 36.5 mg (72% yield, *(E,E)/(E,Z)* > 20:1). Yellow solid (m.p = 128–130 °C).

^{1}H NMR (500 MHz, CDCl$_3$) δ 7.78 (dd, *J* = 10.8, 7.7 Hz, 3H), 7.69 (s, 1H), 7.62 (d, *J* = 8.2 Hz, 2H), 7.54 (d, *J* = 9.6 Hz, 1H), 7.46 (d, *J* = 8.0 Hz, 4H), 7.29 (d, *J* = 8.1 Hz, 2H), 7.15 (d, *J* = 8.1 Hz, 2H), 6.73 (dd, *J* = 15.6, 10.3 Hz, 1H), 6.59 (d, *J* = 15.6 Hz,

1H), 6.18 (dd, J = 15.2, 10.5 Hz, 1H), 5.76 (dd, J = 15.1, 6.7 Hz, 1H), 5.55 (d, J = 7.3 Hz, 1H), 5.14 (t, J = 7.1 Hz, 1H), 2.34 (s, 3H).

^{13}C NMR (126 MHz, CDCl$_3$) δ 143.7, 137.4, 134.5 134.3, 133.7, 133.5, 133.2, 131.1, 129.6, 128.5, 128.1, 127.8, 127.7, 127.6, 127.3, 126.9, 126.6, 126.3, 125.6 (d, J = 3.6 Hz), 123.3, 59.4, 21.5.

HRMS (ESI): *m/z* calculated for $C_{29}H_{24}F_3NO_2S$ [M-H]$^-$: 506.1407, found: 506.1409.

$[\alpha]_D^{23}$ -31.6 (*c* 1.0, CHCl$_3$, 96% *ee* sample).

Enantiomeric excess was determined to be 96% *ee* by chiral HPLC analysis (CHIRALPAK IA (*n*-hexane/^{i}PrOH = 4/1, flow rate 1.0 mL/min, detection at 321.1 nm, t_R = 13.8 min (minor), and 21.1 min (major).

4-methyl-*N-((S,2E,4E)*-5-(naphthalen-2-yl)-1-(thiophen-2-yl)penta-2,4-dien-1-yl)benzenesulfonamide **(3ak)**

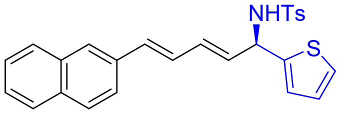

The reaction performed according to the standard procedure A afforded 32.9 mg (74% yield, *(E,E)/(E,Z)* > 20:1). Yellow solid (m.p = 159–163 °C).

^{1}H NMR (500 MHz, CDCl$_3$) δ 7.79 (t, J = 8.8 Hz, 3H), 7.74 – 7.68 (m, 3H), 7.57 (dd, J = 8.6, 1.6 Hz, 1H), 7.50 – 7.42 (m, 2H), 7.26 – 7.22 (m, 3H), 7.05 (d, J = 2.9 Hz, 1H), 6.87 (dd, J = 5.0, 1.2 Hz, 1H), 6.75 (dd, J = 15.6, 10.4 Hz, 1H), 6.62 (d, J = 15.6 Hz, 1H), 6.24 (dd, J = 15.1, 10.4 Hz, 1H), 5.78 (dd, J = 15.1, 6.8 Hz, 1H), 5.16 (t, J = 7.1 Hz, 1H), 4.85 – 4.76 (m, 1H), 2.38 (s, 3H).

^{13}C NMR (126 MHz, CDCl$_3$) δ 143.5, 140.9, 137.8, 134.5, 134.0, 133.7, 133.2, 132.8, 131.7, 129.7, 128.4, 128.1, 127.9, 127.8, 127.4, 126.8, 126.7, 126.5, 126.4, 126.2, 123.4, 122.5, 55.6, 21.7.

IR (thin film): 3843, 3739, 3612, 3269, 3046, 2922, 2357, 1647, 1511, 1423, 1324, 1156, 1089, 1036, 988, 915, 856, 809, 750, 667, 552 cm^{-1}.

HRMS (ESI): *m/z* calculated for $C_{26}H_{23}NO_2S_2$ [M-H]$^-$: 444.1097, found: 444.1092.

$[\alpha]_D^{23}$ +20.0 (*c* 1.0, CHCl$_3$, 98% *ee* sample).

Enantiomeric excess was determined to be 98% *ee* by chiral HPLC analysis (CHIRALPAK IA (*n*-hexane/^{i}PrOH = 4/1, flow rate 1.0 mL/min, detection at 318.0 nm, t_R = 16.3 min (minor), and 16.9 min (major).

N-((R,2E,4E)-1-(3,4-dimethylphenyl)-5-(naphthalen-2-yl)penta-2,4-dien-1-yl)-4-methylbenzenesulfonamide **(3al)**

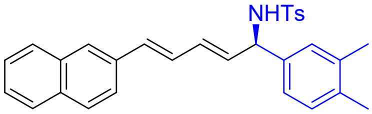

The reaction performed according to the standard procedure A afforded 42.5 mg (91% yield, *(E,E)/(E,Z)* > 20:1). Yellow solid (m.p = 147–149 °C).

^{1}H NMR (500 MHz, CDCl$_3$) δ 7.82 – 7.74 (m, 4H), 7.74 – 7.65 (m, 3H), 7.56 (d, J = 8.6 Hz, 1H), 7.47 (dd, J = 14.4, 7.2 Hz, 2H), 7.22 (d, J = 8.1 Hz, 2H), 7.03 (d, J = 7.7 Hz, 1H), 6.93 – 6.84 (m, 2H), 6.76 (dd, J = 15.6, 10.4 Hz, 1H), 6.60 (d, J = 15.6 Hz, 1H), 6.25 (dd, J = 15.1, 10.5 Hz, 1H), 5.79 (dd, J = 15.1, 6.3 Hz, 1H), 4.98 (dt, J = 13.8, 6.8 Hz, 2H), 2.38 (s, 3H), 2.22 (s, 3H), 2.17 (s, 3H).

^{13}C NMR (126 MHz, CDCl$_3$) δ 143.3, 137.8, 137.1, 137.1, 136.4, 134.6, 133.7, 133.6, 133.1,

132.7, 132.3, 130.0, 129.5, 128.4, 128.4, 128.2, 128.1, 127.8, 127.5, 126.6, 126.5, 126.1, 124.5, 123.4, 59.5, 21.6, 19.8, 19.5.
HRMS (ESI): *m/z* calculated for $C_{30}H_{29}NO_2S$ $[M-H]^-$: 466.1846, found: 466.1849.
Enantiomeric excess was determined to be 93% *ee* by chiral HPLC analysis (CHIRALPAK IA (*n*-hexane/^{i}PrOH = 9/1, flow rate 1.0 mL/min, detection at 280.0 nm, t_R = 26.5 min (minor), and 28.7 min (major).

N-((R,2E,4E)-5-(naphthalen-2-yl)-1-phenylpenta-2,4-dien-1-yl)benzenesulfonamide **(3am)**

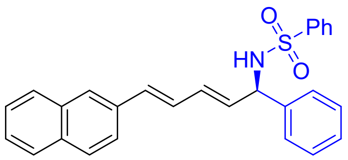

The reaction performed according to the standard procedure A afforded 37.0 mg (87% yield, *(E,E)/(E,Z)* = 7:1). White solid (m.p = 144–146 °C).
^{1}H NMR (500 MHz, CDCl3) δ 7.76 (dd, J = 32.1, 20.0 Hz, 6H), 7.57 – 7.39 (m, 7H), 7.25 (d, J = 6.9 Hz, 2H), 7.17 (dd, J = 7.4, 1.9 Hz, 2H), 6.75 (dd, J = 15.6, 10.4 Hz, 1H), 6.60 (d, J = 15.6 Hz, 1H), 6.25 (dd, J = 15.1, 10.5 Hz, 1H), 5.80 (dd, J = 15.1, 6.6 Hz, 1H), 5.24 (d, J = 7.2 Hz, 1H), 5.11 (t, J = 6.9 Hz, 1H).
^{13}C NMR (126 MHz, $CDCl_3$) δ 140.7, 139.6, 134.5, 133.9, 133.7, 133.2, 132.8, 132.6, 132.2, 129.0, 128.9, 128.4, 128.1, 128.0, 127.9, 127.8, 127.4, 127.2, 126.8, 126.5, 126.2, 123.4, 59.7.
HRMS (ESI): *m/z* calculated for $C_{27}H_{23}NO_2S$ $[M-H]^-$: 424.1377, found: 424.1382.

$[\alpha]_D^{23}$ -31.5 (*c* 1.0, $CHCl_3$, 97% *ee* sample).

Enantiomeric excess was determined to be 97% *ee* by chiral HPLC analysis (CHIRALPAK IA (*n*-hexane/^{i}PrOH = 4/1, flow rate 1.0 mL/min, detection at 321.1 nm, t_R = 13.7 min (minor), and 17.5 min (major).

4-chloro-*N-((R,2E,4E)*-5-(naphthalen-2-yl)-1-phenylpenta-2,4-dien-1-yl)benzenesulfonamide **(3an)**

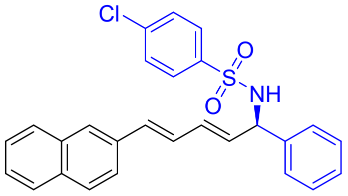

The reaction performed according to the standard procedure A afforded 22.0 mg (48% yield, *(E,E)/(E,Z)* > 20:1). Colourless liquid.
^{1}H NMR (500 MHz, $CDCl_3$) δ 7.87 (d, J = 8.5 Hz, 3H), 7.80 (d, J = 8.7 Hz, 2H), 7.73 – 7.65 (m, 3H), 7.49 (d, J = 8.5 Hz, 4H), 7.36 (d, J = 8.5 Hz, 2H), 7.20 – 7.13 (m, 2H), 6.76 (dd, J = 15.6, 10.4 Hz, 1H), 6.63 (d, J = 15.6 Hz, 1H), 6.23 (dd, J = 15.1, 10.4 Hz, 1H), 5.79 (dd, J = 15.1, 6.2 Hz, 1H), 5.11 (dt, J = 13.4, 6.9 Hz, 2H).
^{13}C NMR (126 MHz, $CDCl_3$) δ 139.1, 134.4, 134.4, 133.7, 133.3, 133.2, 131.8, 130.1, 129.2, 129.0, 128.9, 128.5, 128.2, 128.2, 127.8, 127.7, 127.2, 126.9, 126.9, 126.6, 126.2, 123.4, 59.9.
HRMS (ESI): *m/z* calculated for $C_{27}H_{22}ClNO_2S$ $[M-H]^-$: 458.0987, found: 458.0992.

$[\alpha]_D^{23}$ -21.0 (*c* 1.0, $CHCl_3$, 84% *ee* sample).

Enantiomeric excess was determined to be 84% *ee* by chiral HPLC analysis (CHIRALPAK IA (*n*-hexane/^{i}PrOH = 4/1, flow rate 1.0 mL/min, detection at 321.1 nm, t_R = 13.9 min (minor), and 17.5 min (major).

5.2. Unsuccessful Substrates Attempts

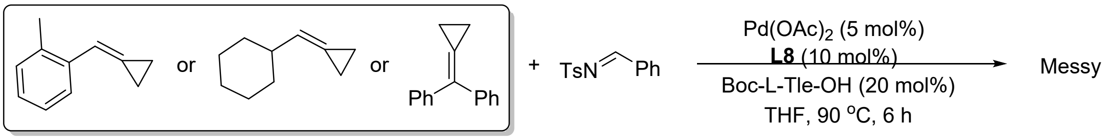

To further expand the substrate scope, some differently substituted ACPs were tested under the optimal conditions. Unfortunately, complex reaction profiles were generally observed as outlined above.

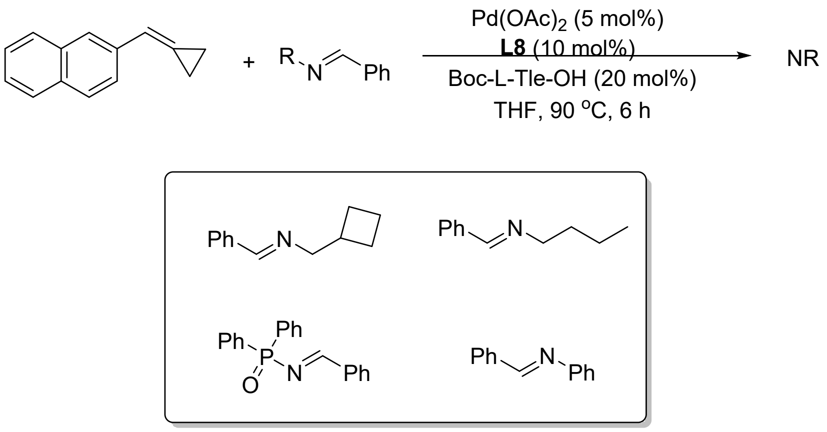

Meanwhile, the above outlined imines were inert to the reaction under the optimized conditions.

6 X-ray Crystal Structure Determination of the Products

To grow the crystals used to collect the X-ray data for **3ba**, the following method was used: the sample was dissolved with 3 mL ^{i}PrOH and 1 mL *n*-hexane in a small vial, which was kept aside at room temperature to obtain crystals.

Single crystals of $C_{24}H_{23}NO_2S$ [04-0817] were. A suitable crystal was selected and on a XtaLAB AFC12 (RINC): Kappa single diffractometer. The crystal was kept at 100.00(10) K during data collection. Using Olex2 [1], the structure was solved with the SHELXS [2] structure solution program using Direct Methods and refined with the SHELXL [3] refinement package using Least Squares minimisation.

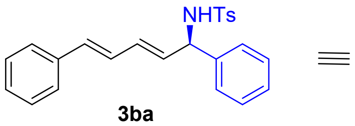

3ba

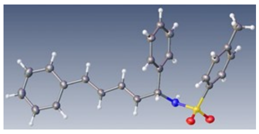

X-ray of **3ba**

Figure S3. The X-ray Diffraction Configuration of **3ba**. Thermal ellipsoid plots (50% probability)

Table S8. Crystallographic data for compounds **3ba**

Identification code	**3ba**
Empirical formula	$C_{24}H_{23}NO_2S$
Formula weight	389.49
Temperature/K	100.00(10)
Crystal system	orthorhombic

Space group	$P2_12_12_1$
a/Å	8.3434(2)
b/Å	9.5869(3)
c/Å	25.2570(6)
α/°	90
β/°	90
γ/°	90
Volume/Å^3	2020.24(9)
Z	4
ρ_{calc}g/cm^3	1.281
μ/mm^{-1}	1.569
F(000)	824.0
Crystal size/mm^3	0.14 × 0.12 × 0.11
Radiation	Cu Kα (λ = 1.54184)
2Θ range for data collection/°	7 to 143.974
Index ranges	-9 ≤ h ≤ 10, -11 ≤ k ≤ 11, -30 ≤ l ≤ 31
Reflections collected	6222
Independent reflections	3382 [R_{int} = 0.0246, R_{sigma} = 0.0375]
Data/restraints/parameters	3382/0/258
Goodness-of-fit on F^2	1.071
Final R indexes [I>=2σ (I)]	R_1 = 0.0376, wR_2 = 0.0931
Final R indexes [all data]	R_1 = 0.0387, wR_2 = 0.0937
Largest diff. peak/hole / e Å^{-3}	0.34/-0.45

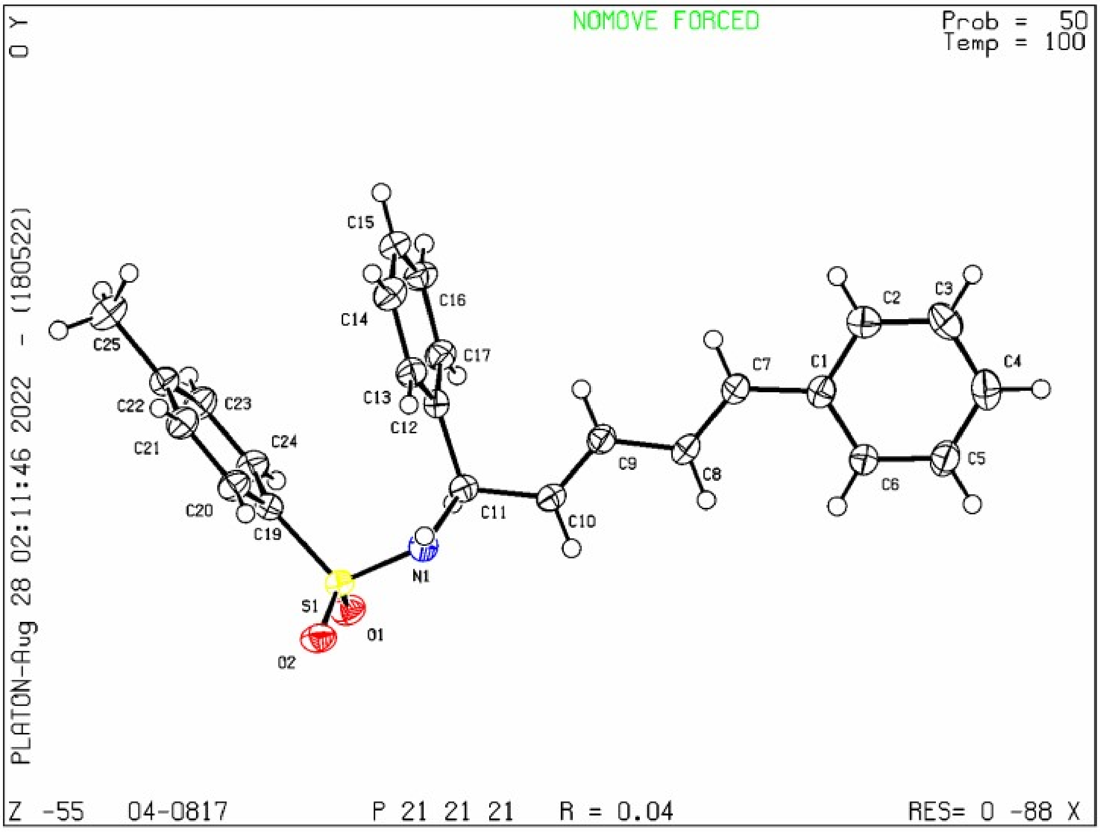

7. References

1. Han, J.; Zhou, W.; Zhang, P.-C.; Wang, H.; Zhang, H. Design and Synthesis of WJ-Phos, and Application in Cu-Catalyzed Enantioselective Boroacylation of 1,1-Disubstituted Allenes. *ACS Catal*. **2019**, *9*, 6890-6895.

2. Medina, J. M.; Kang, T. K. T.; Keary M. Engle Cu-Catalyzed Hydroboration of Benzylidenecyclopropanes: Reaction Optimization, (Hetero)Aryl Scope, and Origins of Pathway Selectivity. *ACS Catal*. **2019**, *9*, 11130-11136.

3. Zhu, Z.-Z.; Chen, K.; Yu, L.-Z.; Tang, X.-Y.; Shi, M. Copper(I)-Catalyzed Intramolecular Trifluoromethylation of Methylenecyclopropanes. *Org. Lett*. **2015**, *17*, 5994-5997.

4. Fürstner, A.; Aïssa, C. PtCl2-Catalyzed Rearrangement of Methylenecyclopropanes. *J. Am. Chem. Soc.* **2006**, *128*, 6306-6307.

5. Morales, S. Guijarro, F. G. García Ruano, J. L. Cid, M. B. A General Aminocatalytic Method for the Synthesis of Aldimines. *J. Am. Chem. Soc*. **2014**, *136*, 1082–1089.

6. Xiao, B.-X., Jiang, B., Yan, R-J., Zhu, J.-X., Chen, Y.-C. A Palladium Complex as an Asymmetric π-Lewis Base Catalyst for Activating 1,3-Dienes. *J. Am. Chem. Soc.* **2021**, *143*, 4809−4816.

8. Copies of NMR Spectra for Compounds

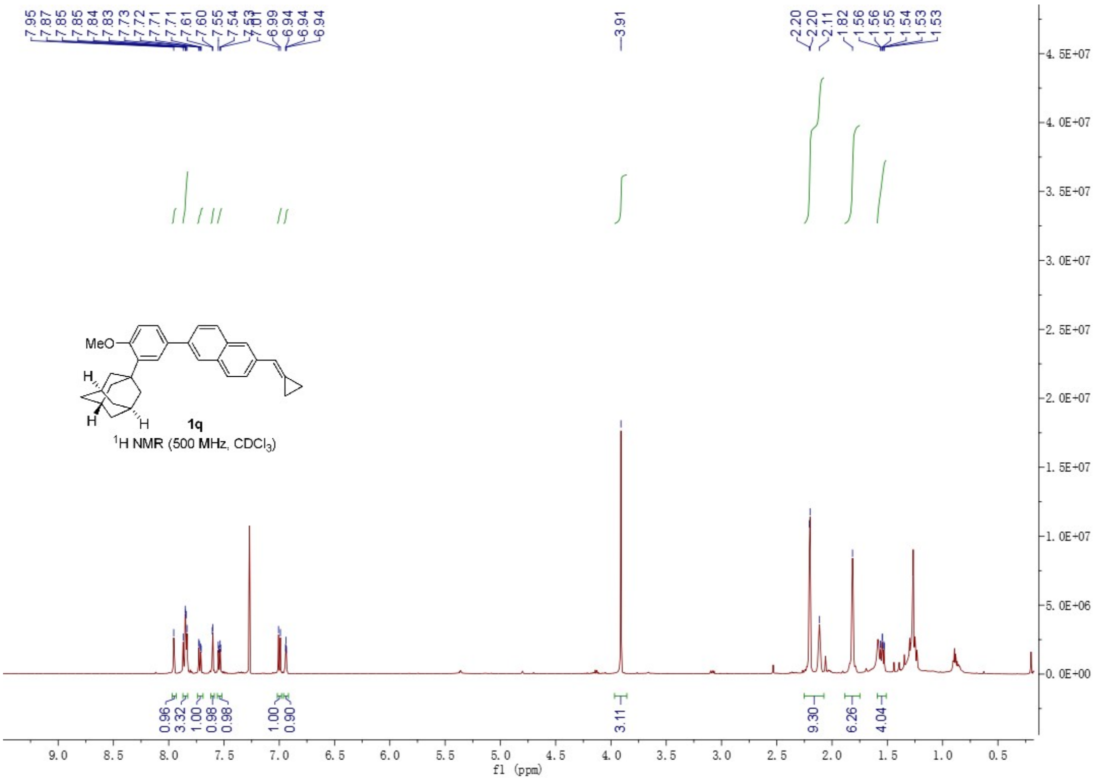

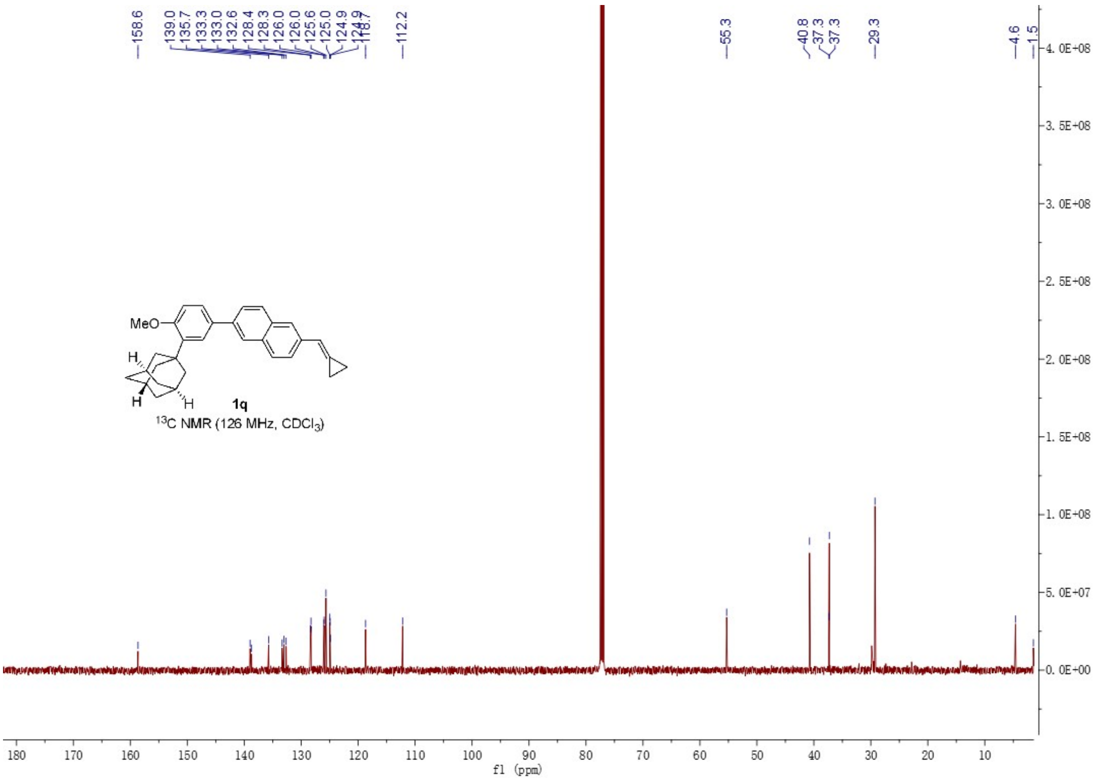

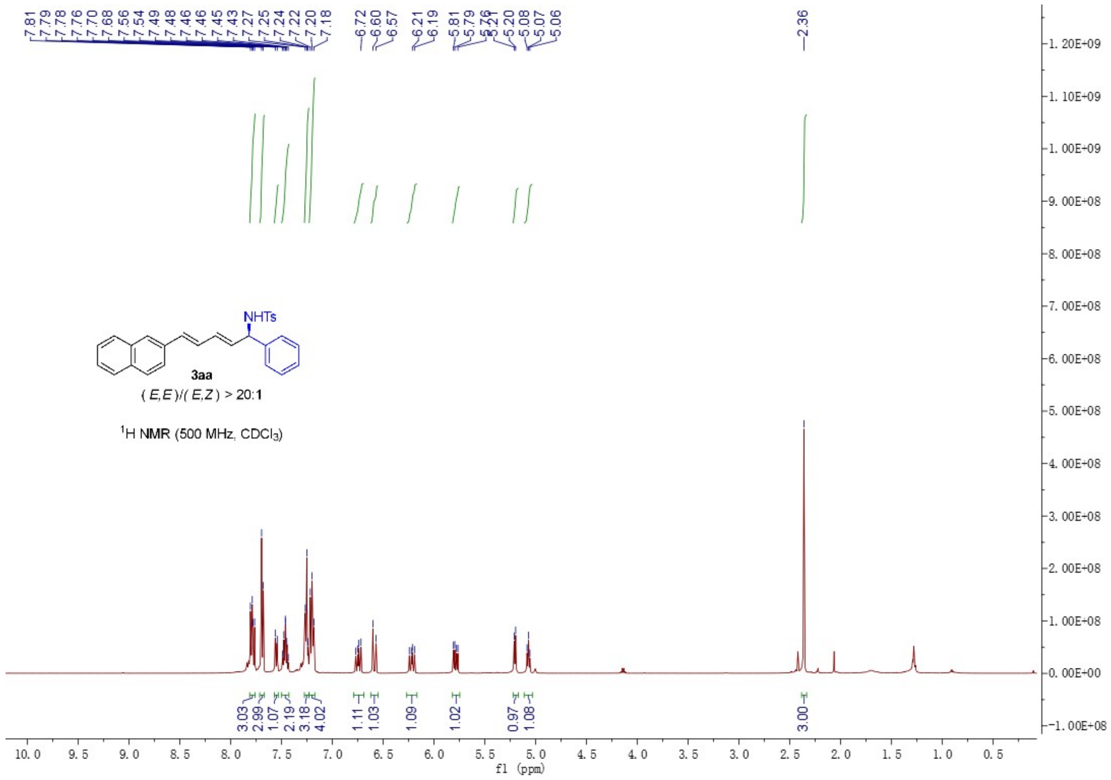
NHTs
3aa
(E,E)/(E,Z) > 20:1
1H NMR (500 MHz, CDCl3)
f1 (ppm)

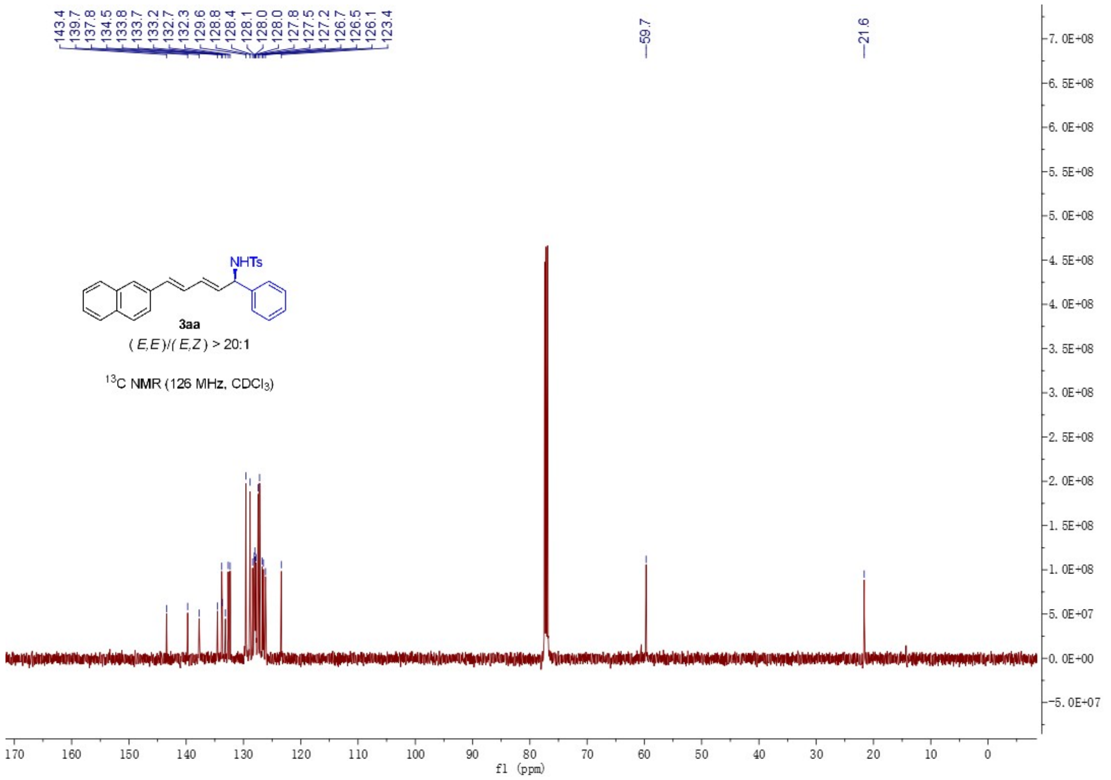
NHTs
3aa
(E,E)/(E,Z) > 20:1
13C NMR (126 MHz, CDCl3)
f1 (ppm)

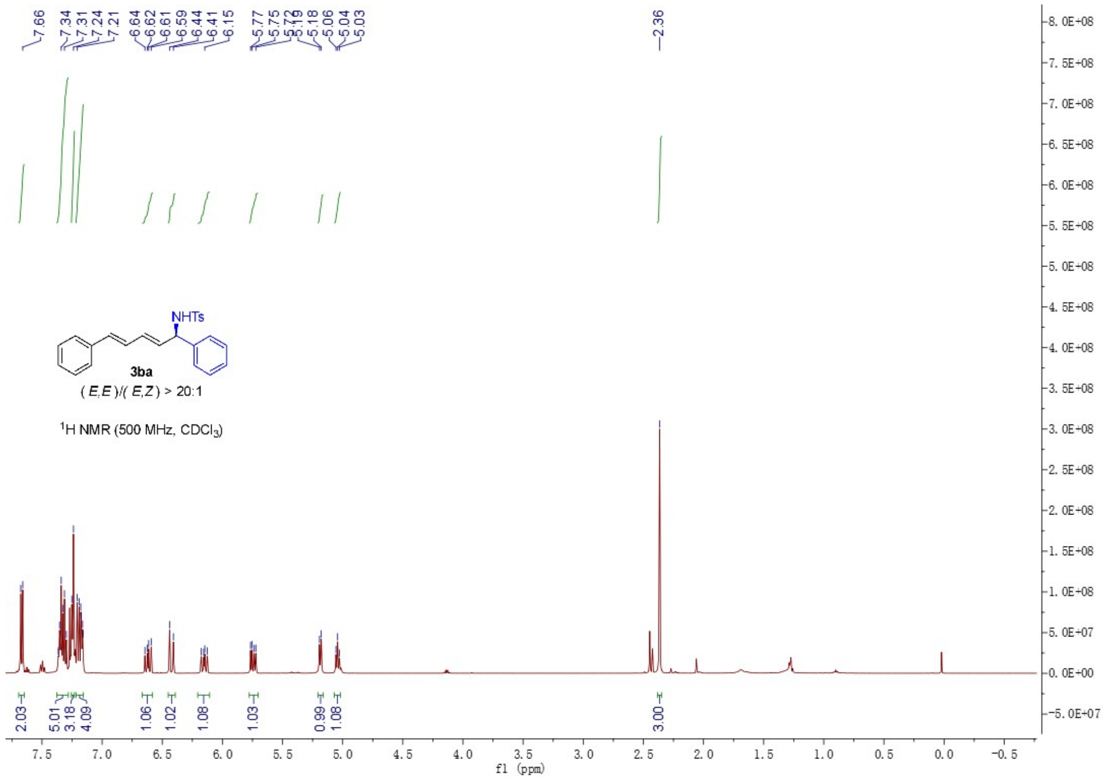
NHTs
3ba
(E,E)/(E,Z) > 20:1
^{1}H NMR (500 MHz, CDCl$_3$)
fl (ppm)

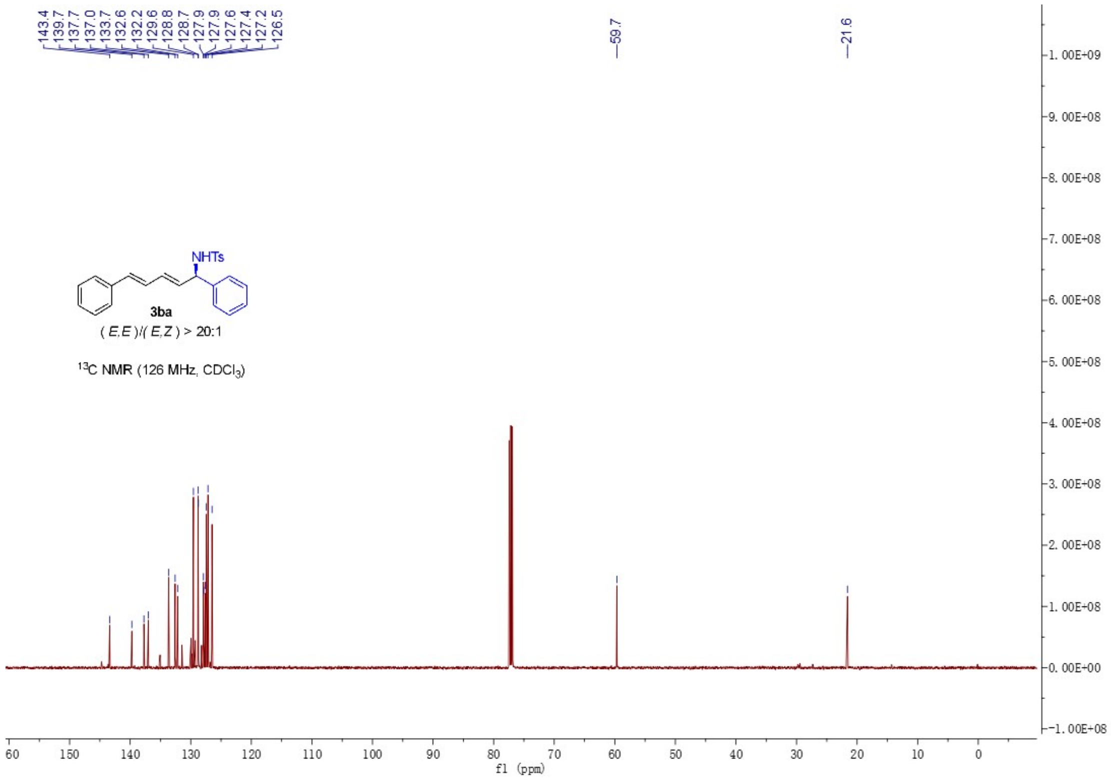
NHTs
3ba
(E,E)/(E,Z) > 20:1
^{13}C NMR (126 MHz, CDCl$_3$)
fl (ppm)

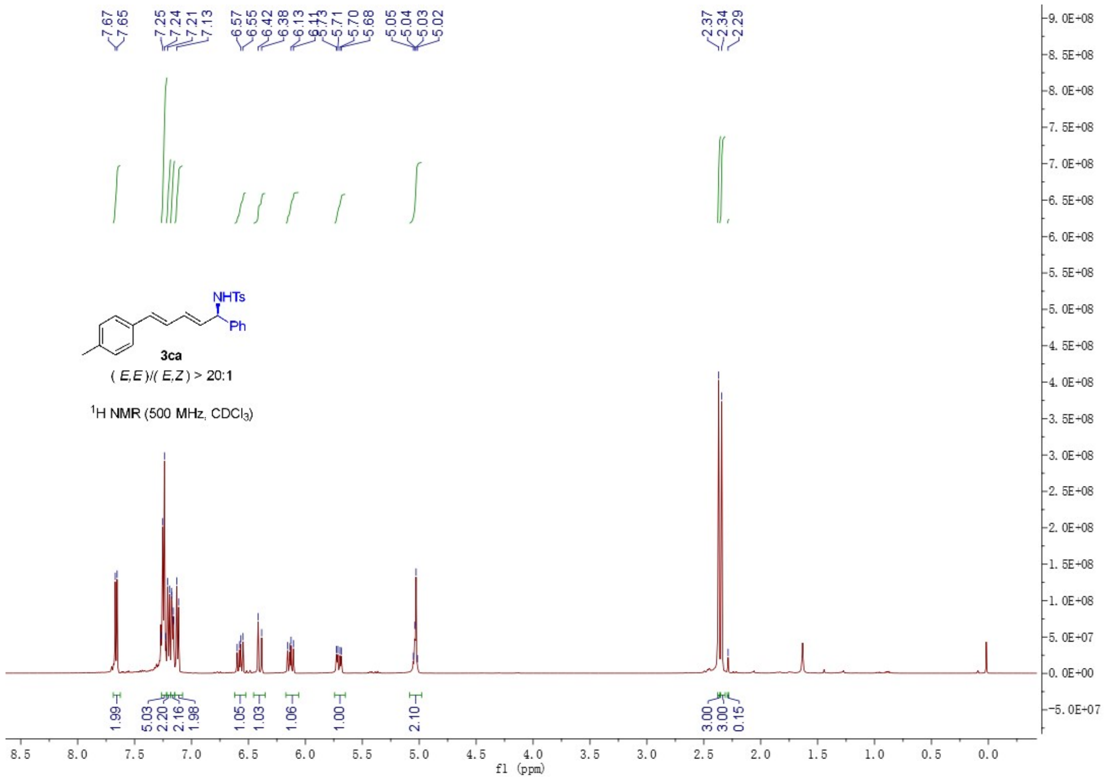
NHTs
Ph
3ca
(E,E)/(E,Z) > 20:1
1H NMR (500 MHz, CDCl3)
f1 (ppm)

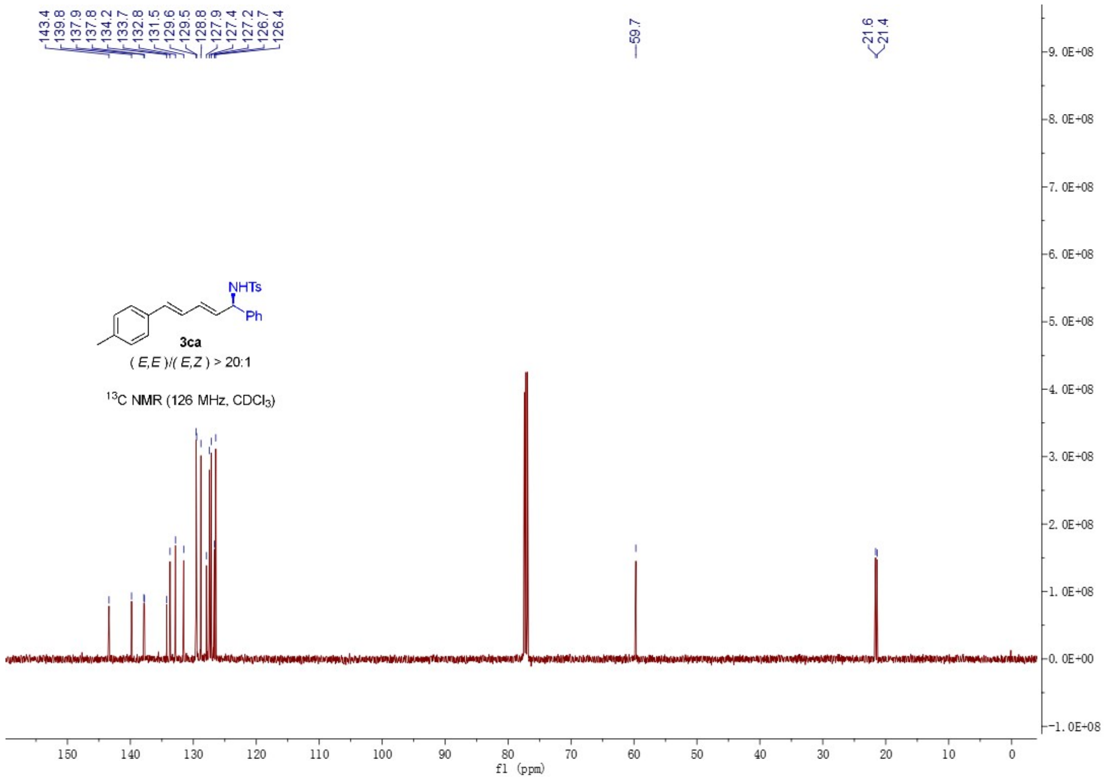
NHTs
Ph
3ca
(E,E)/(E,Z) > 20:1
13C NMR (126 MHz, CDCl3)
f1 (ppm)

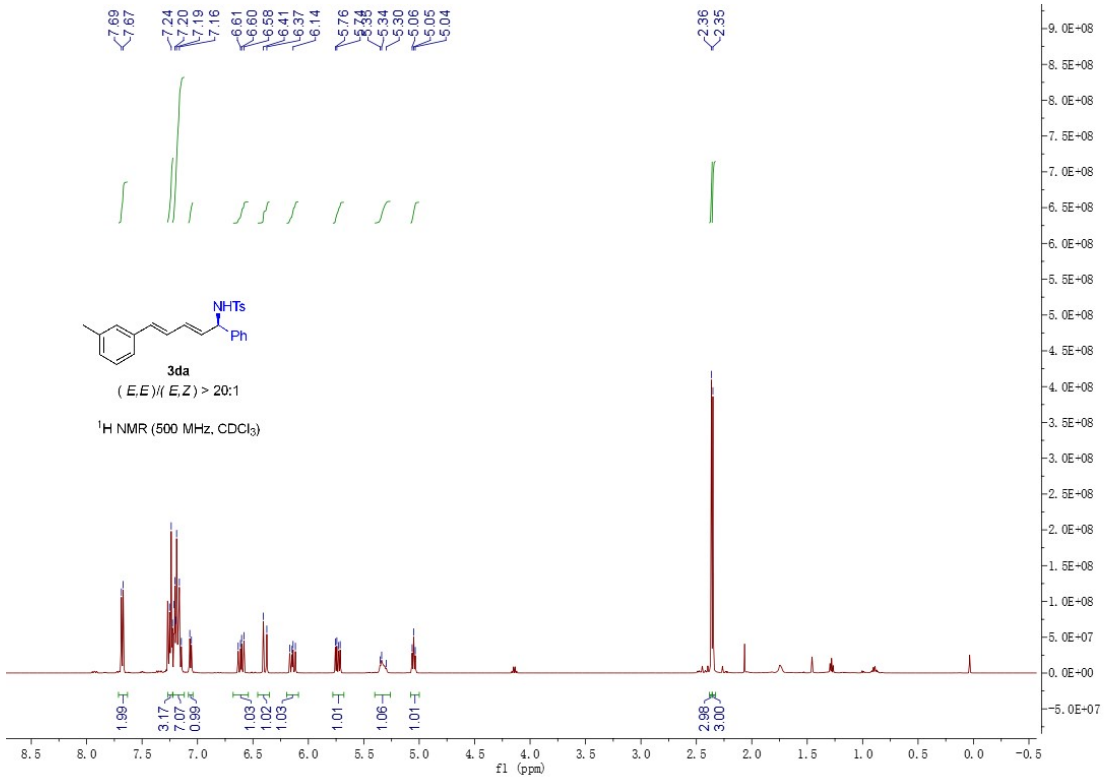
NHTs
Ph
3da
(E,E)/(E,Z) > 20:1
^{1}H NMR (500 MHz, CDCl3)
fl (ppm)

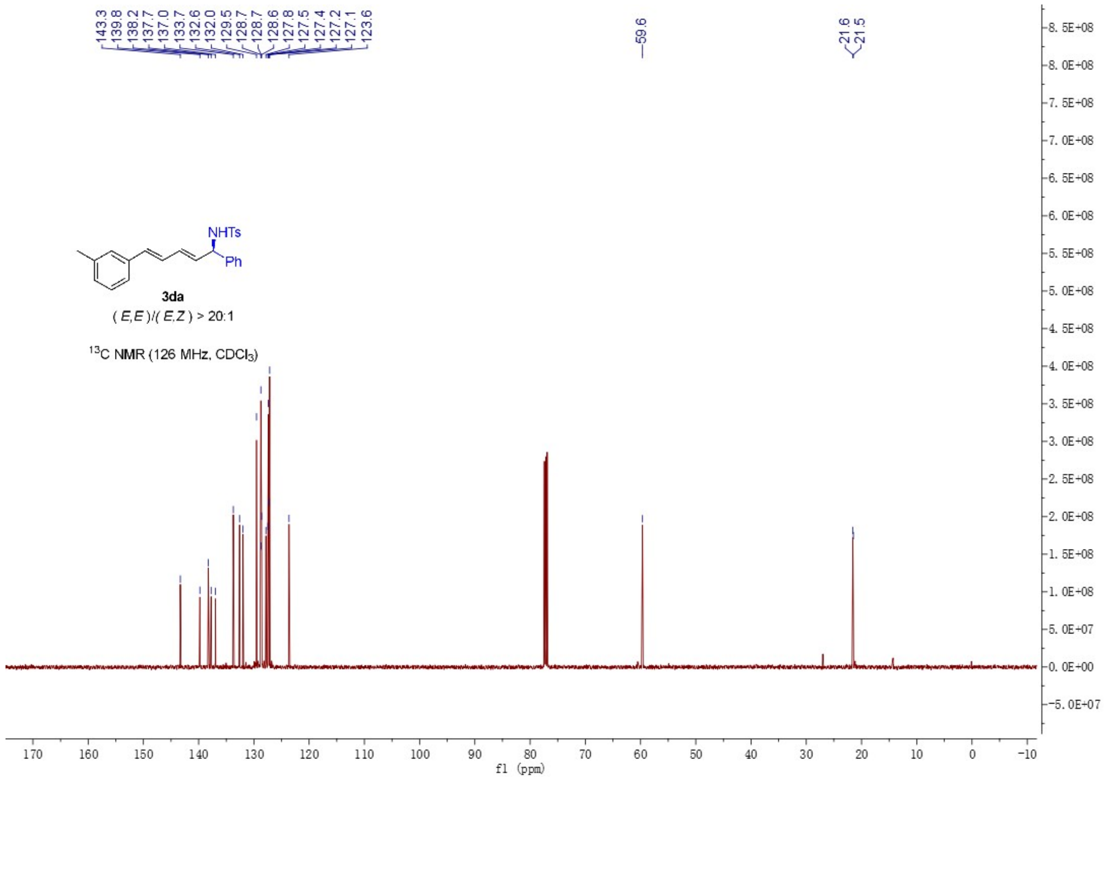
NHTs
Ph
3da
(E,E)/(E,Z) > 20:1
^{13}C NMR (126 MHz, CDCl3)
fl (ppm)

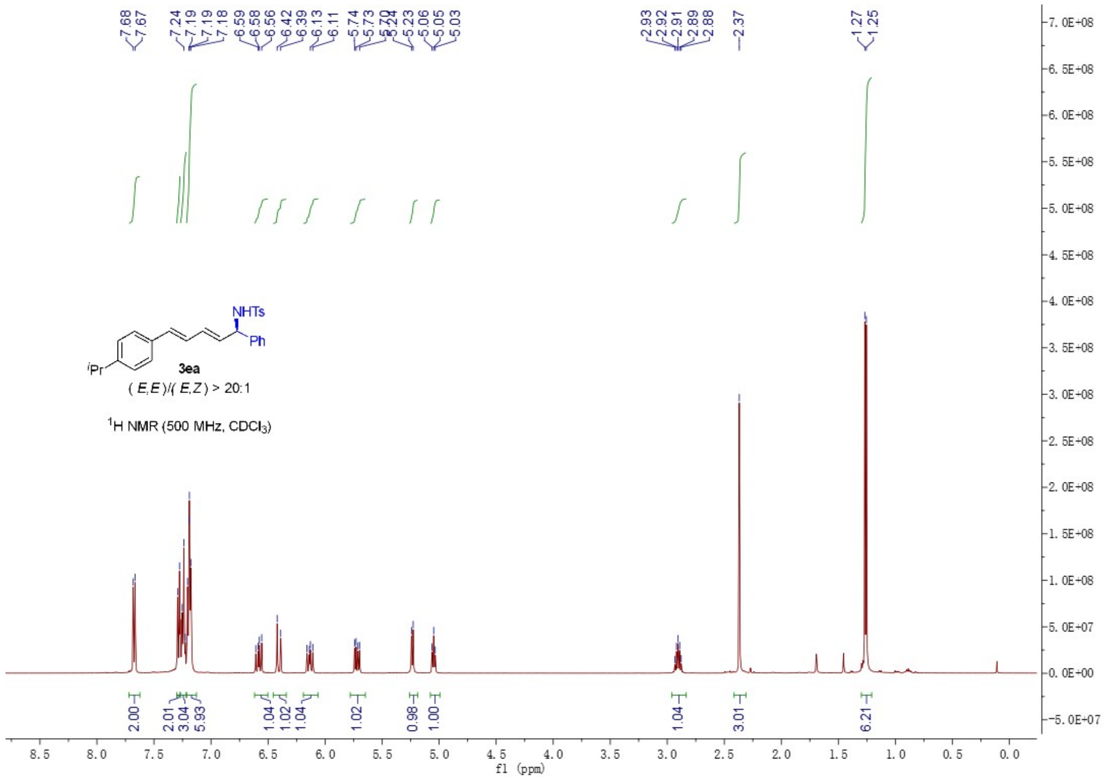
7.68
7.67
7.24
7.19
7.19
7.18
6.59
6.58
6.56
6.42
6.39
6.13
6.11
5.74
5.73
5.24
5.23
5.06
5.05
5.03
2.93
2.92
2.91
2.89
2.88
2.37
1.27
1.25
NHTs
Ph
^{i}Pr
3ea
(E,E)/(E,Z) > 20:1
^{1}H NMR (500 MHz, $CDCl_3$)
2.00
2.01
3.04
5.93
1.04
1.02
1.04
1.02
0.98
1.00
1.04
3.01
6.21
f1 (ppm)

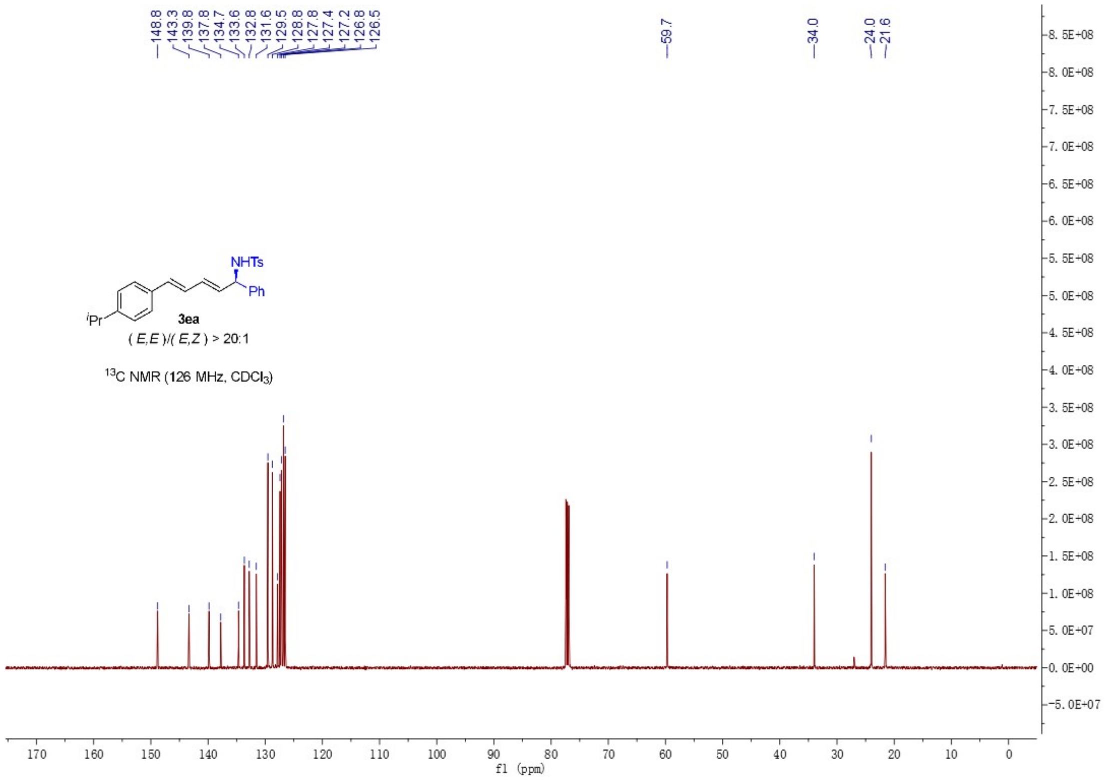
148.8
143.3
139.8
137.8
134.7
133.6
132.8
131.6
129.5
128.8
127.8
127.4
127.2
126.8
126.5
59.7
34.0
24.0
21.6
NHTs
Ph
^{i}Pr
3ea
(E,E)/(E,Z) > 20:1
^{13}C NMR (126 MHz, $CDCl_3$)
f1 (ppm)

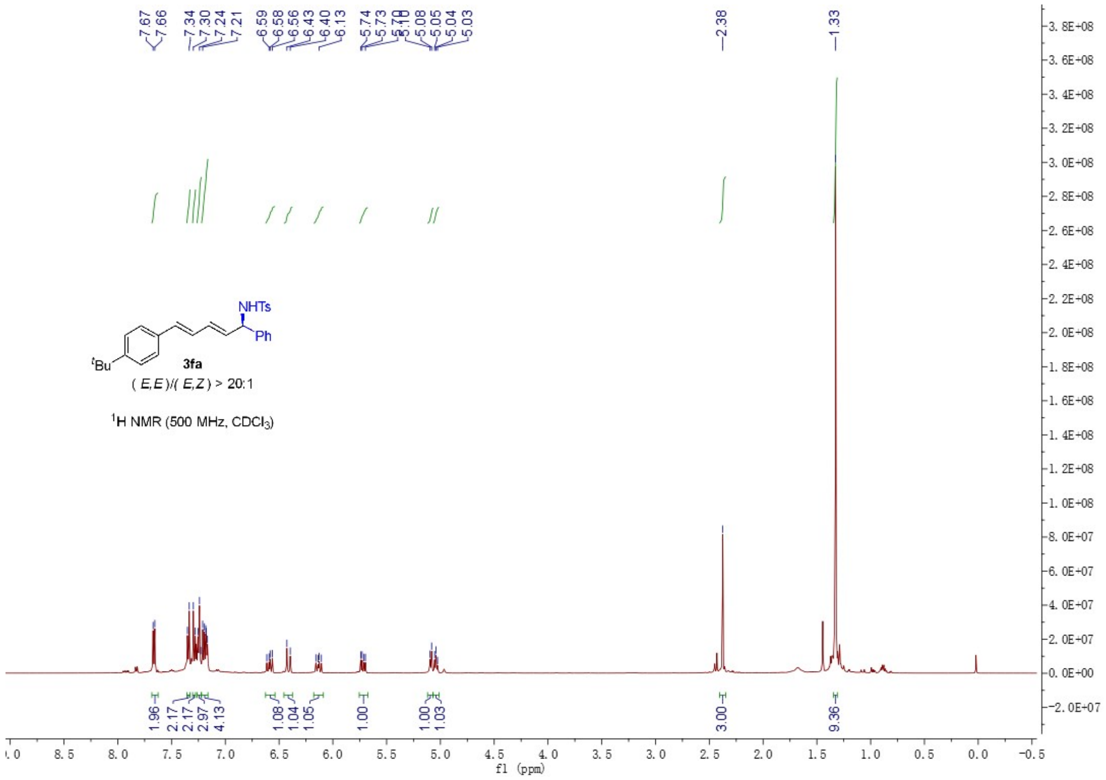
7.67
7.66
7.34
7.30
7.24
7.21
6.59
6.58
6.56
6.43
6.40
6.13
5.74
5.73
5.70
5.10
5.08
5.05
5.04
5.03
2.38
1.33
NHTs
Ph
tBu
3fa
(E,E)/(E,Z) > 20:1
1H NMR (500 MHz, CDCl3)
1.96
2.17
2.17
2.97
4.13
1.08
1.04
1.05
1.00
1.00
1.03
3.00
9.36
f1 (ppm)

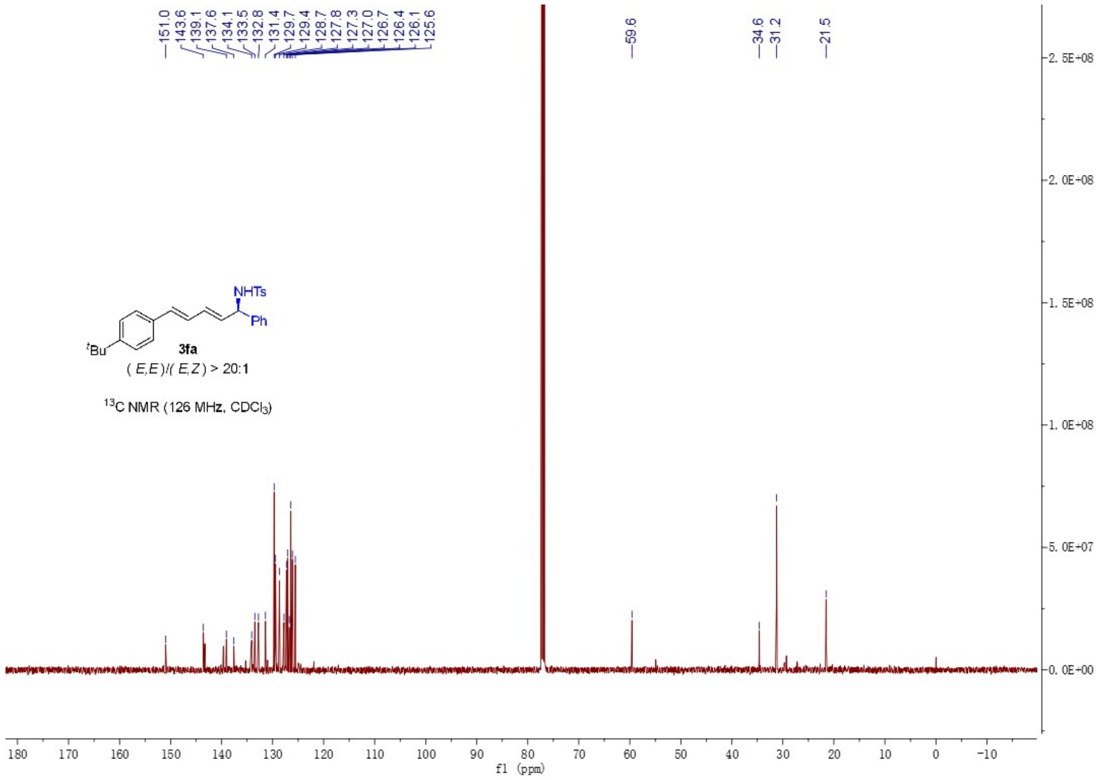
151.0
143.6
139.1
137.6
134.1
133.5
132.8
131.4
129.7
129.4
128.7
127.8
127.3
127.0
126.7
126.4
126.1
125.6
59.6
34.6
31.2
21.5
NHTs
Ph
tBu
3fa
(E,E)/(E,Z) > 20:1
13C NMR (126 MHz, CDCl3)
f1 (ppm)

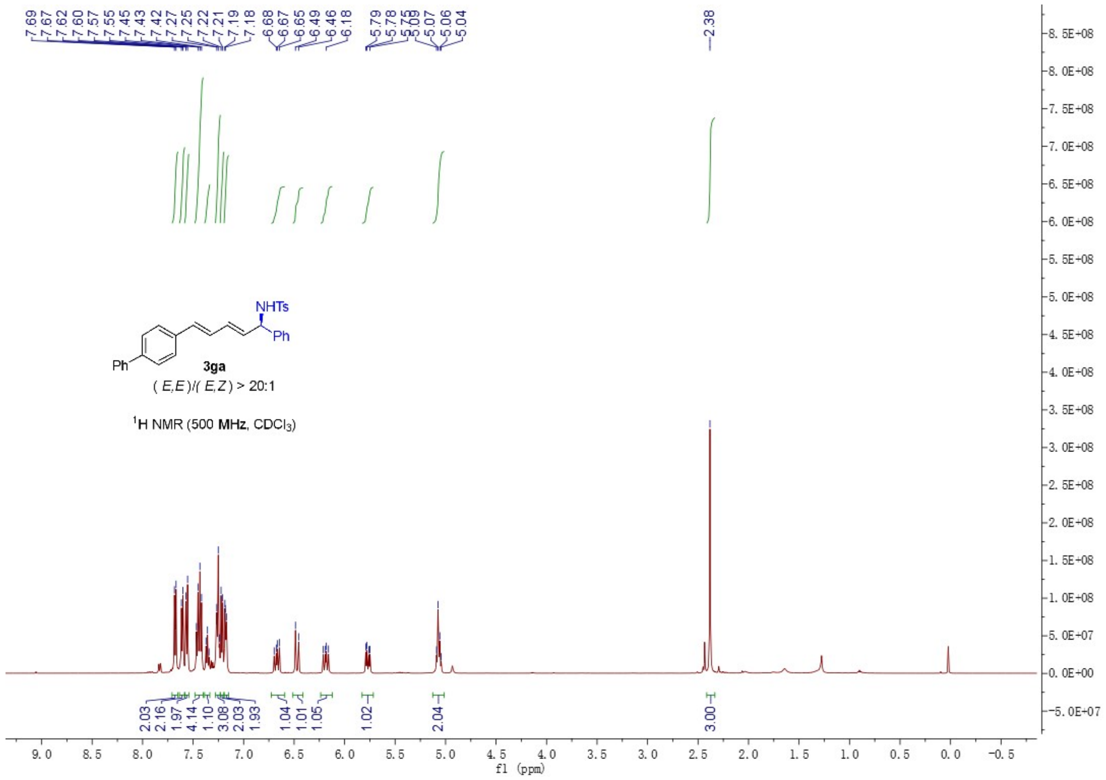
NHTs
Ph
Ph
3ga
(E,E)/(E,Z) > 20:1
1H NMR (500 MHz, CDCl3)
f1 (ppm)

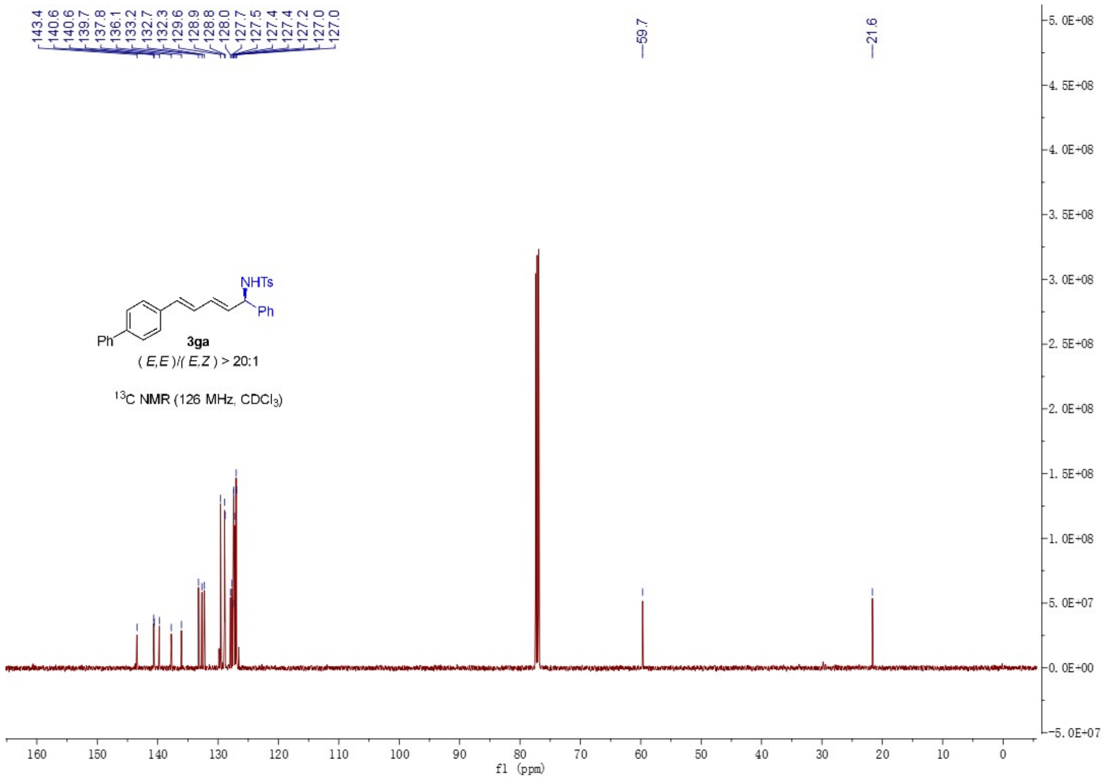
NHTs
Ph
Ph
3ga
(E,E)/(E,Z) > 20:1
13C NMR (126 MHz, CDCl3)
f1 (ppm)

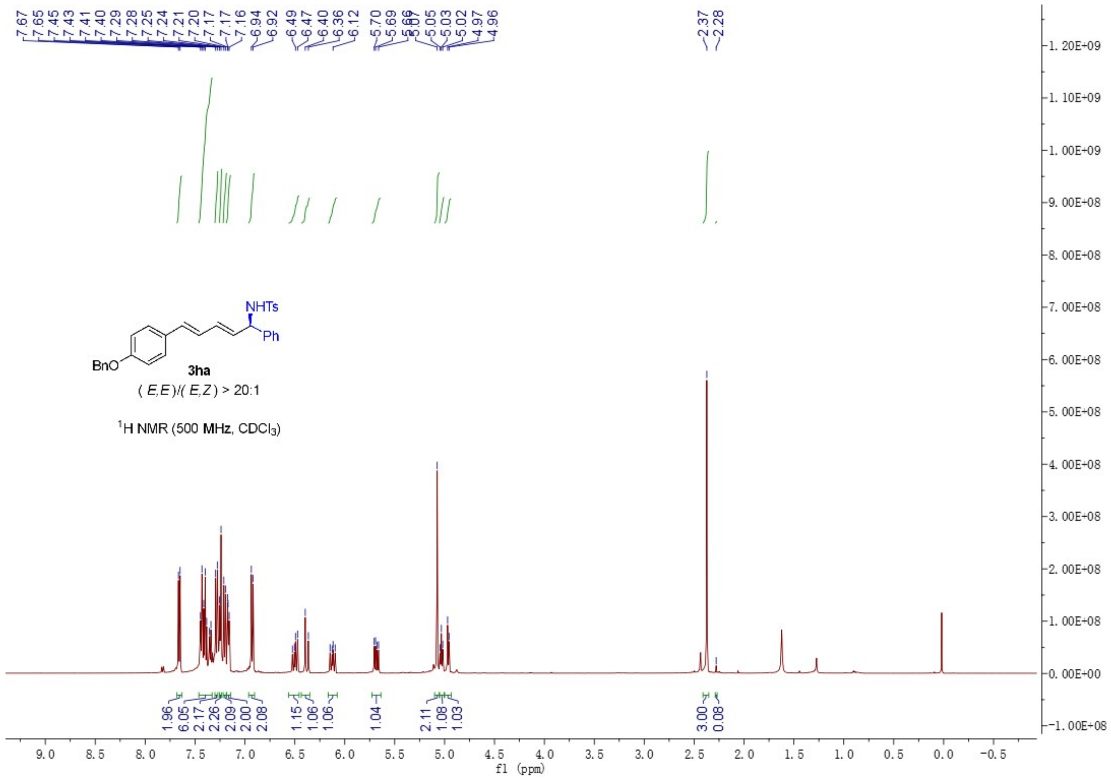
7.67
7.65
7.45
7.43
7.41
7.40
7.29
7.28
7.25
7.24
7.21
7.20
7.17
7.17
7.16
6.94
6.92
6.49
6.47
6.40
6.36
6.12
5.70
5.69
5.05
5.03
5.02
4.97
4.96
2.37
2.28
NHTs
Ph
BnO
3ha
(E,E)/(E,Z) > 20:1
1H NMR (500 MHz, CDCl3)
1.96
6.05
2.17
2.26
2.09
2.00
2.08
1.15
1.06
1.06
1.04
2.11
1.08
1.03
3.00
0.08
fl (ppm)

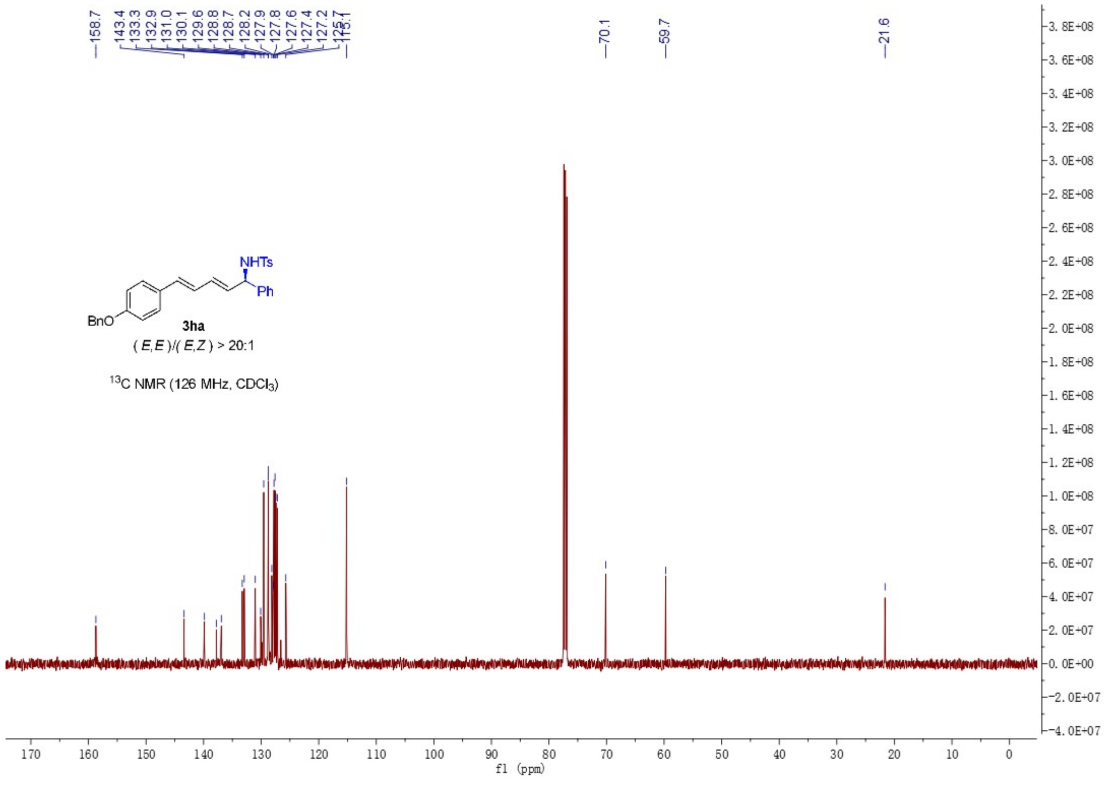
158.7
143.4
133.3
132.9
131.0
130.1
129.6
128.8
128.7
128.2
127.9
127.8
127.6
127.4
127.2
125.7
115.1
70.1
59.7
21.6
NHTs
Ph
BnO
3ha
(E,E)/(E,Z) > 20:1
13C NMR (126 MHz, CDCl3)
fl (ppm)

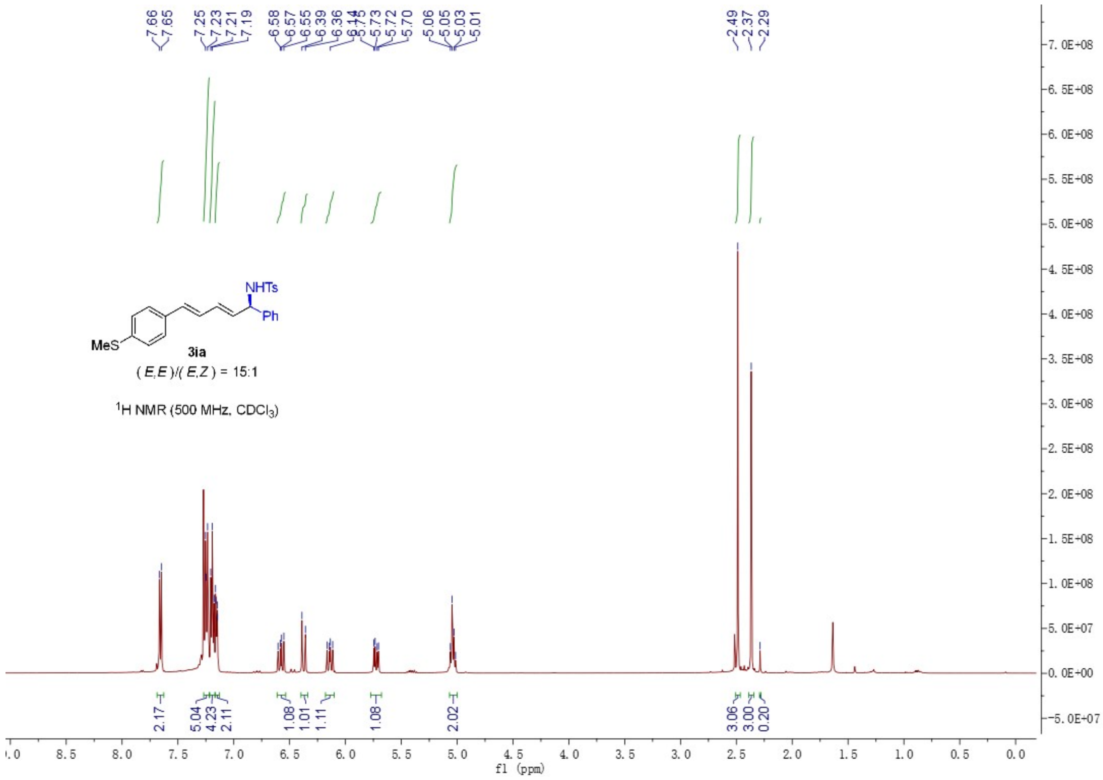

7.66
7.65
7.25
7.23
7.21
7.19
6.58
6.57
6.55
6.39
6.36
6.14
5.75
5.73
5.72
5.70
5.06
5.05
5.03
5.01
2.49
2.37
2.29
NHTs
Ph
MeS
3ia
(E,E)/(E,Z) = 15:1
¹H NMR (500 MHz, CDCl₃)
2.17
5.04
4.23
2.11
1.08
1.01
1.11
1.08
2.02
3.06
3.00
0.20
f1 (ppm)

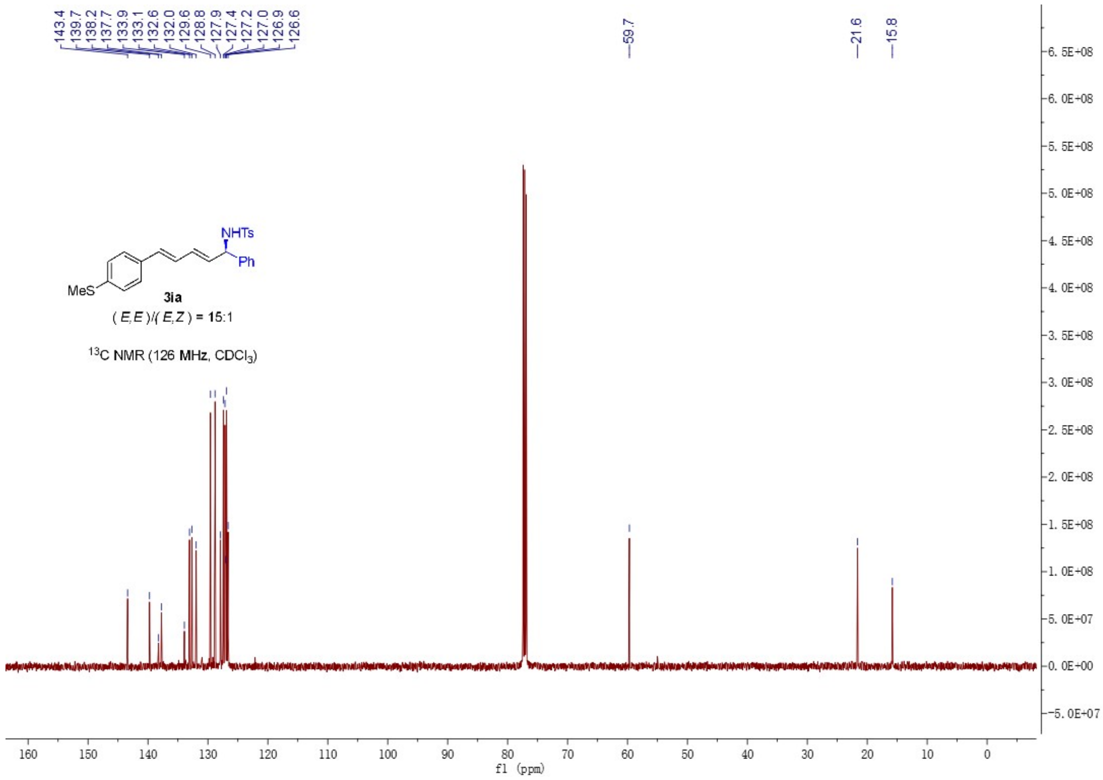

143.4
139.7
138.2
137.7
133.9
133.1
132.6
132.0
129.6
128.8
127.9
127.4
127.2
127.0
126.9
126.6
59.7
21.6
15.8
NHTs
Ph
MeS
3ia
(E,E)/(E,Z) = 15:1
¹³C NMR (126 MHz, CDCl₃)
f1 (ppm)

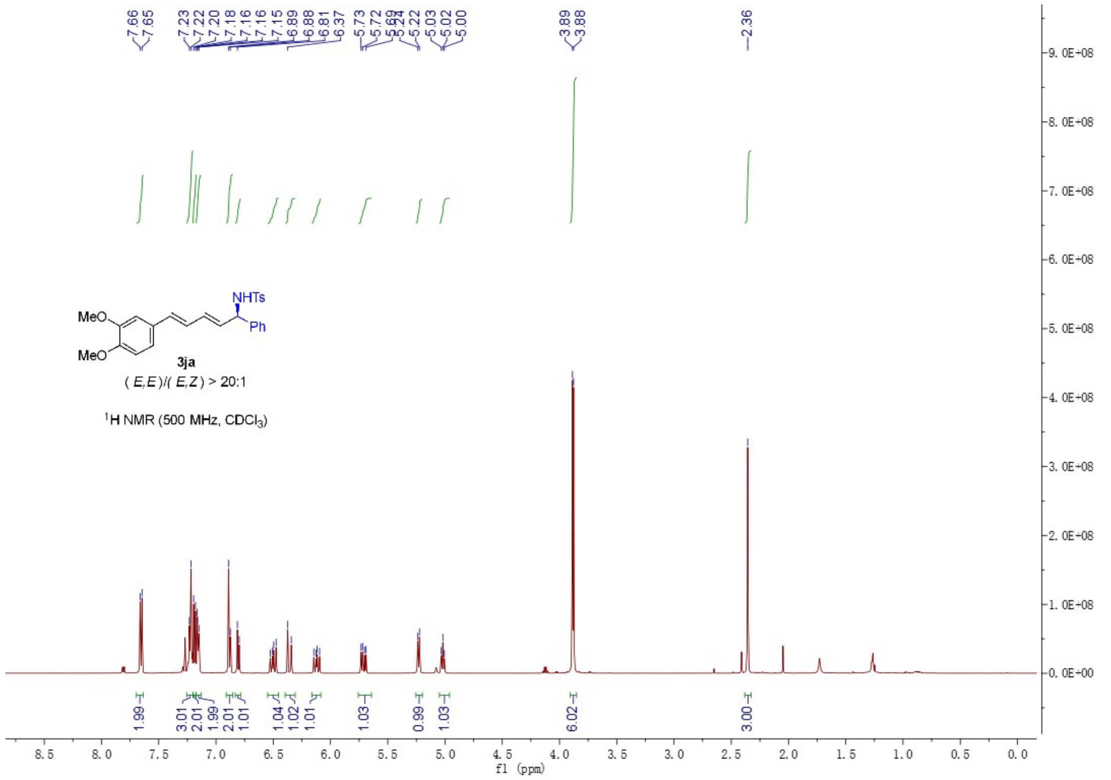
NHTs
MeO
MeO
Ph
3ja
(E,E)/(E,Z) > 20:1
1H NMR (500 MHz, CDCl3)
f1 (ppm)

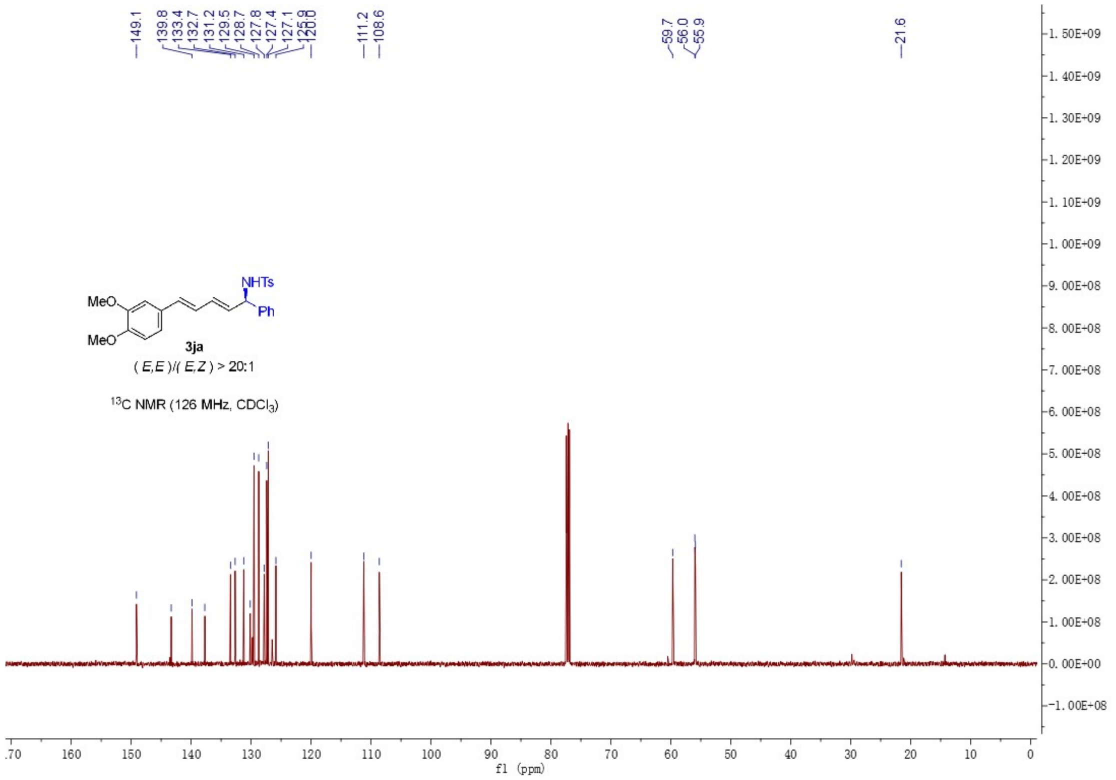
NHTs
MeO
MeO
Ph
3ja
(E,E)/(E,Z) > 20:1
13C NMR (126 MHz, CDCl3)
f1 (ppm)

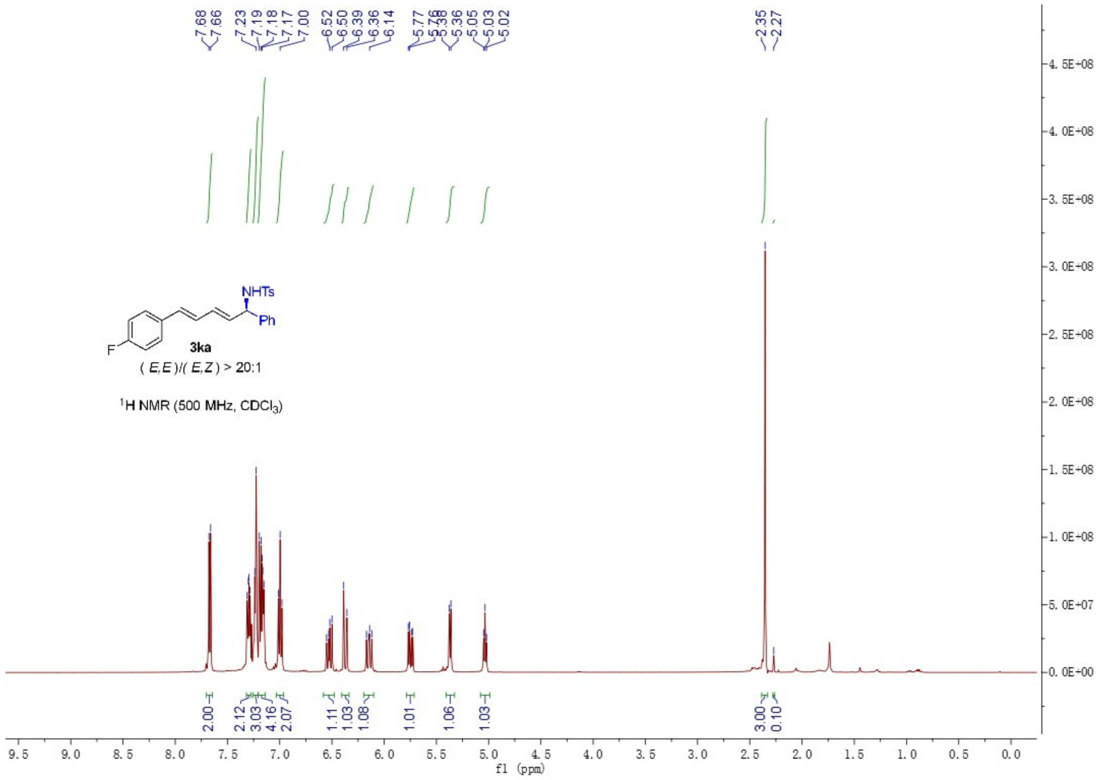
NHTs
Ph
F
3ka
(E,E)/(E,Z) > 20:1
1H NMR (500 MHz, CDCl3)
fl (ppm)

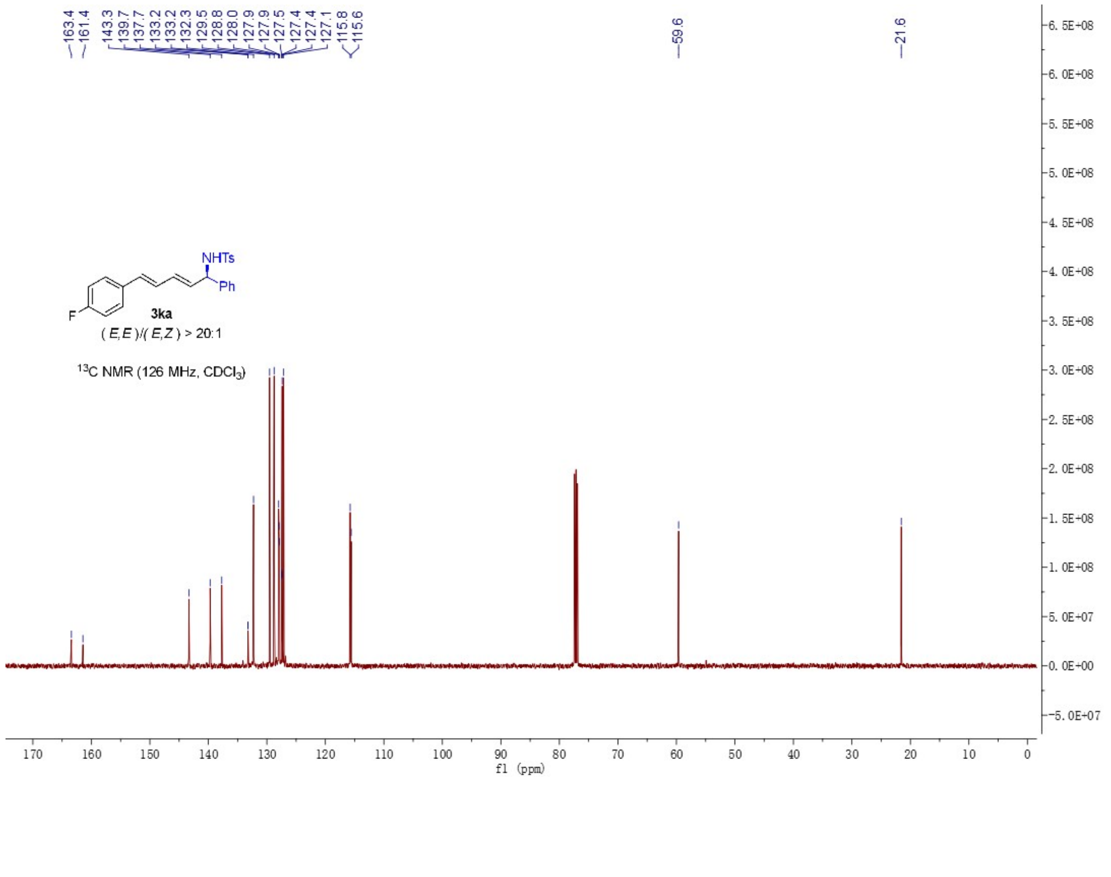
NHTs
Ph
F
3ka
(E,E)/(E,Z) > 20:1
13C NMR (126 MHz, CDCl3)
fl (ppm)

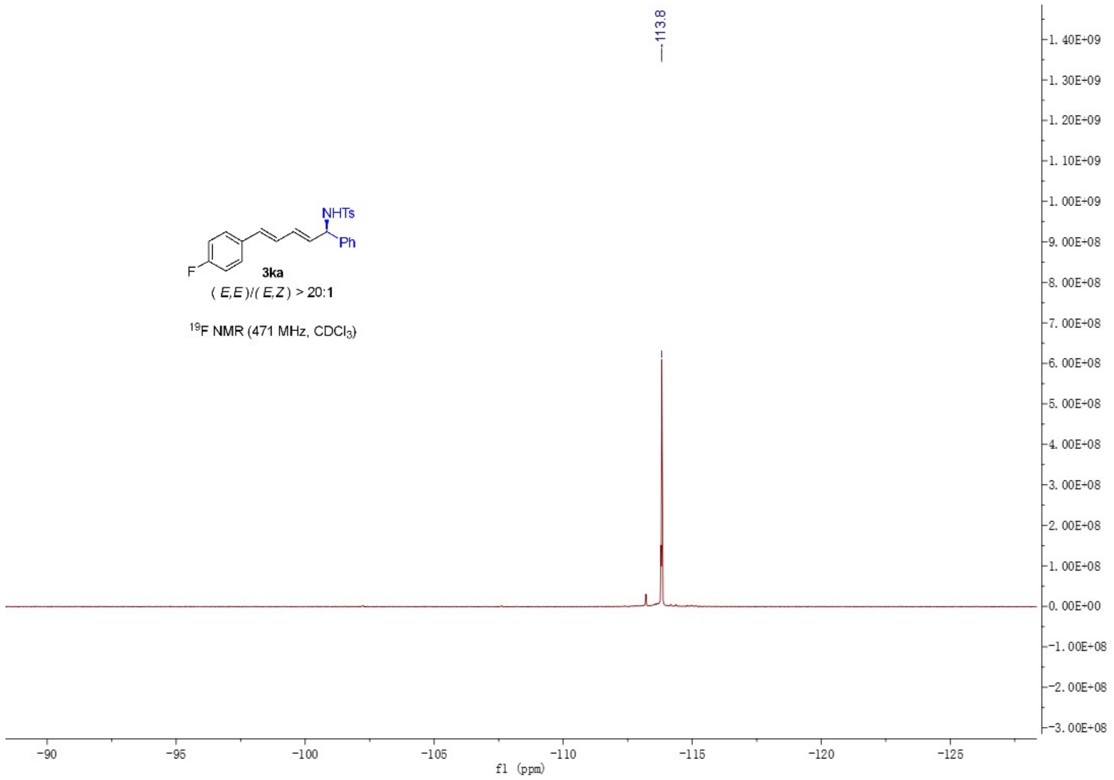

113.8
NHTs
Ph
F
3ka
(E,E)/(E,Z) > 20:1
19F NMR (471 MHz, CDCl3)
f1 (ppm)

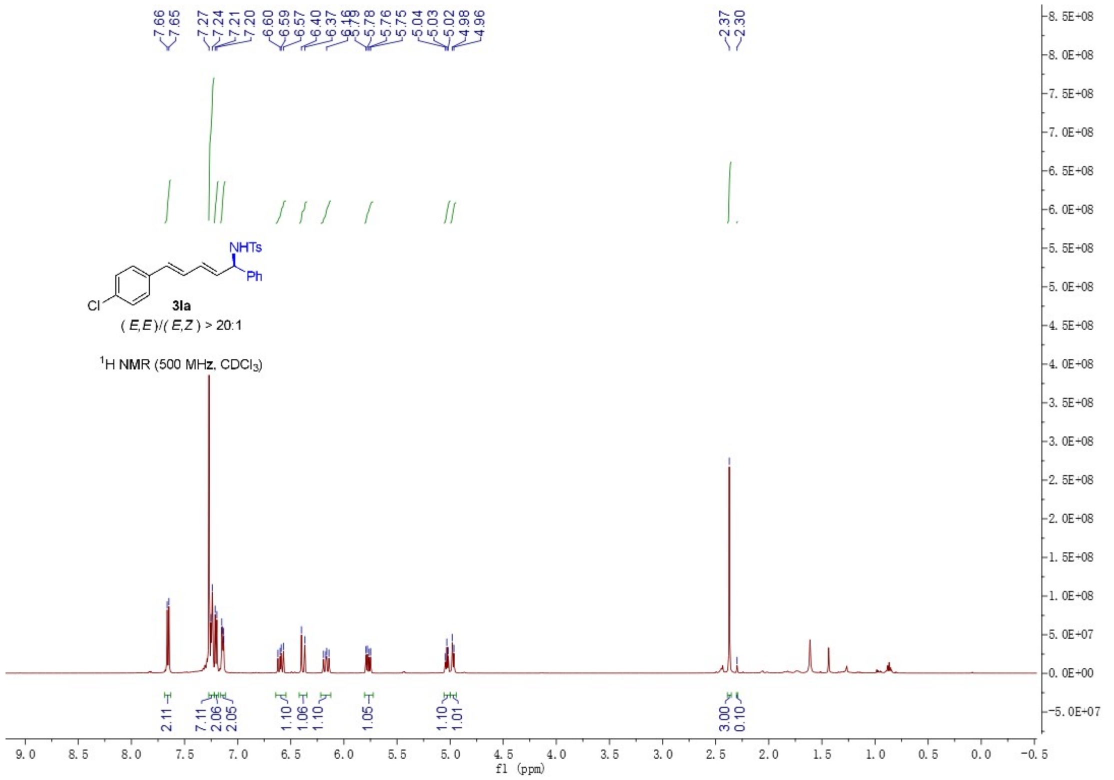

NHTs
Ph
Cl
3la
(E,E)/(E,Z) > 20:1
1H NMR (500 MHz, CDCl3)
f1 (ppm)

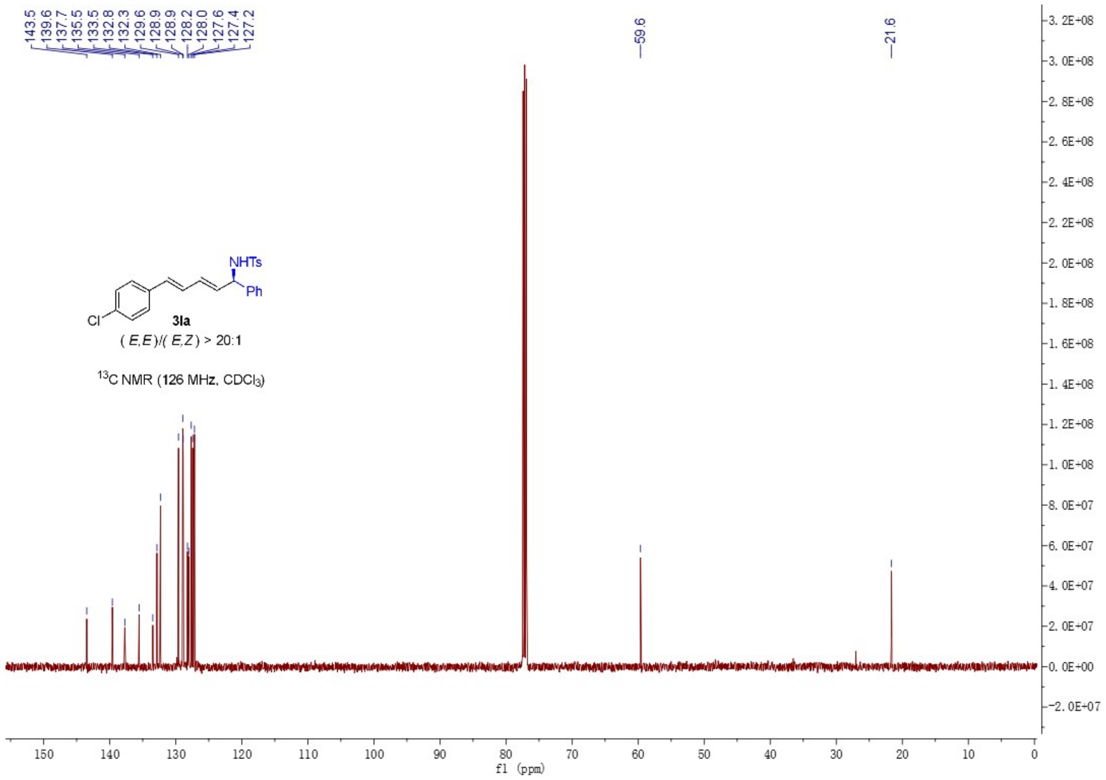
143.5
139.6
137.7
135.5
133.5
132.8
132.3
129.6
128.9
128.9
128.2
128.0
127.6
127.4
127.2
59.6
21.6
NHTs
Ph
Cl
3la
(E,E)/(E,Z) > 20:1
13C NMR (126 MHz, CDCl3)
f1 (ppm)

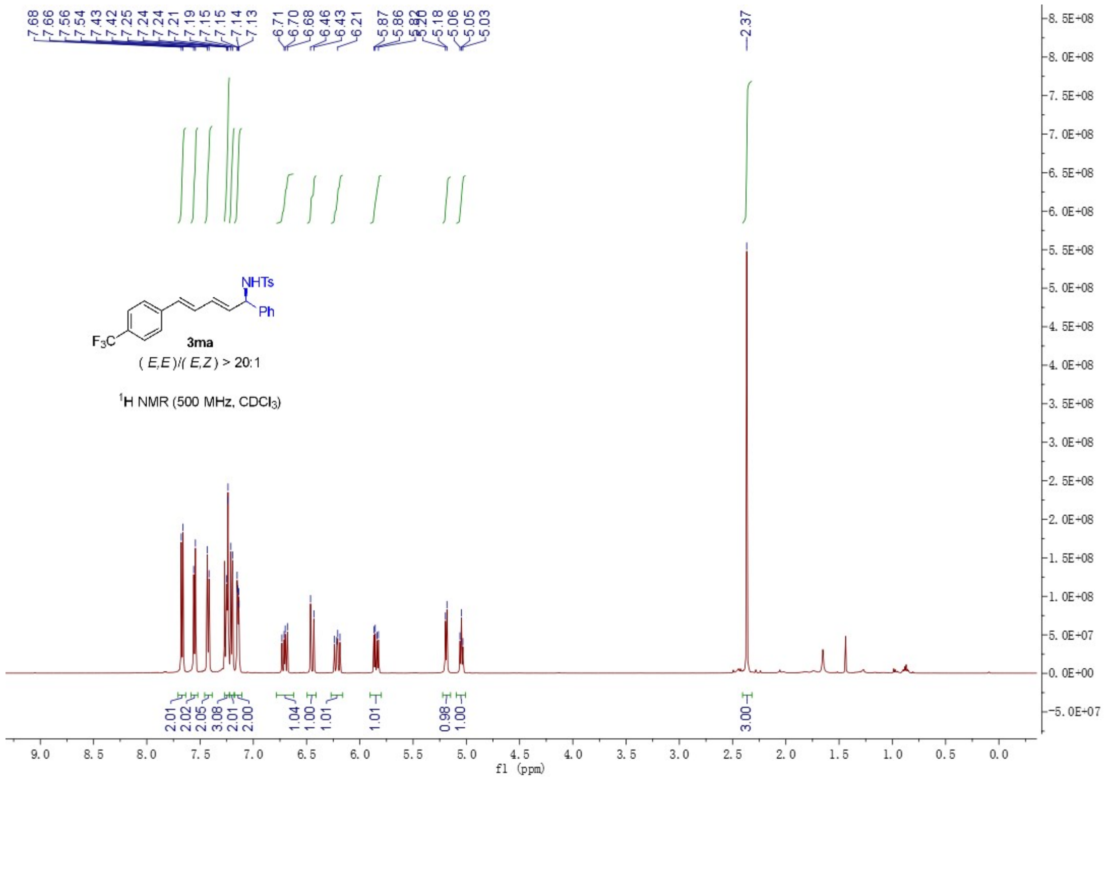
7.68
7.66
7.54
7.43
7.42
7.25
7.24
7.21
7.19
7.15
7.14
7.13
6.71
6.70
6.68
6.46
6.43
6.21
5.87
5.86
5.82
5.20
5.18
5.06
5.05
5.03
2.37
NHTs
Ph
F3C
3ma
(E,E)/(E,Z) > 20:1
1H NMR (500 MHz, CDCl3)
2.01
2.02
2.05
3.08
2.01
2.00
1.04
1.00
1.01
1.01
0.98
1.00
3.00
f1 (ppm)

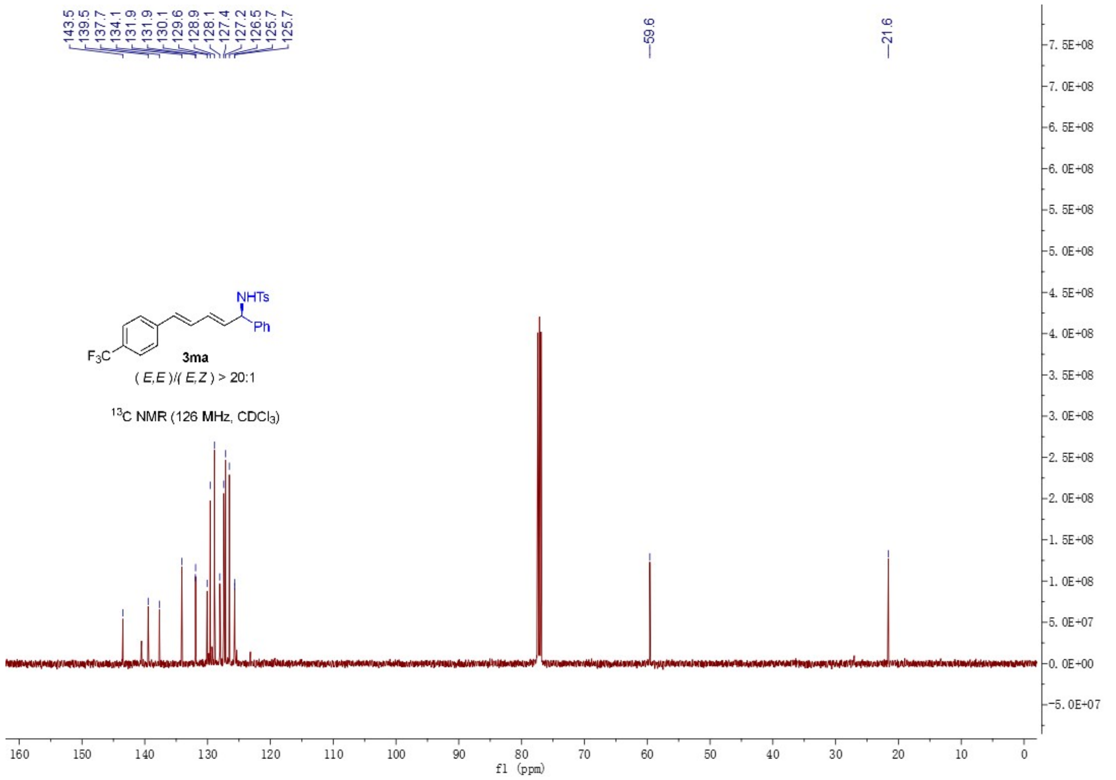
143.5
139.5
137.7
134.1
131.9
131.9
130.1
129.6
128.9
128.1
127.4
127.2
126.5
125.7
125.7
59.6
21.6
NHTs
Ph
F3C
3ma
(E,E)/(E,Z) > 20:1
13C NMR (126 MHz, CDCl3)
f1 (ppm)

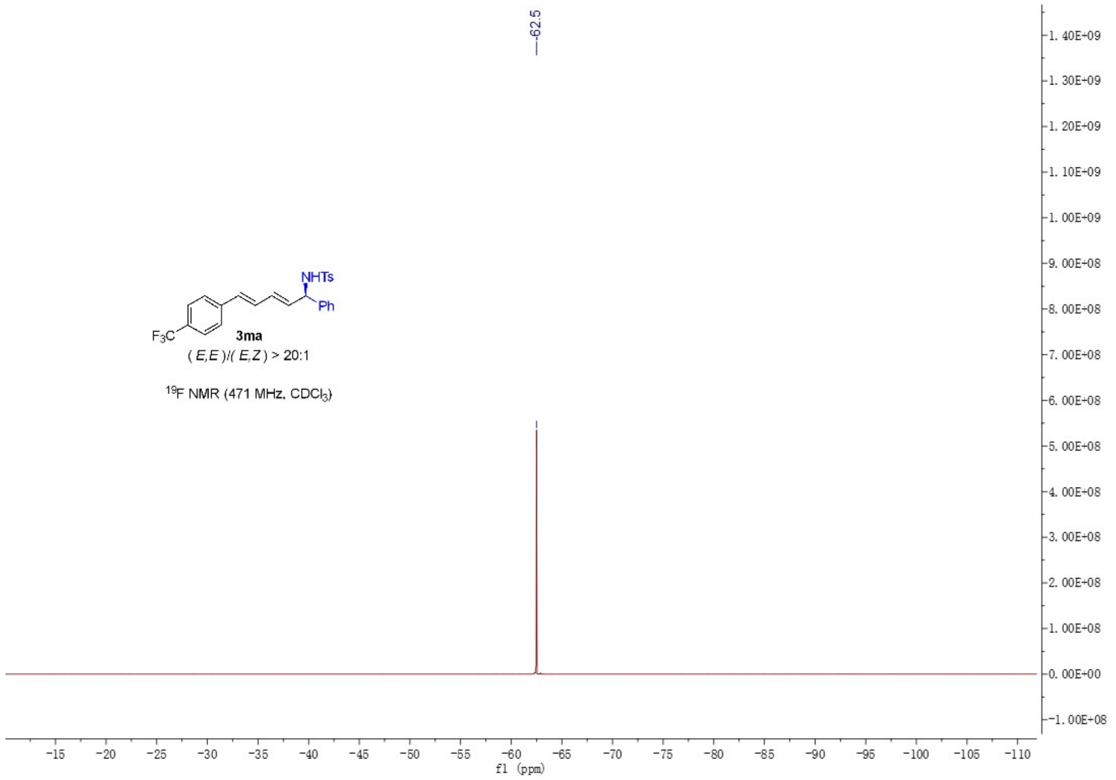
-62.5
NHTs
Ph
F3C
3ma
(E,E)/(E,Z) > 20:1
19F NMR (471 MHz, CDCl3)
f1 (ppm)

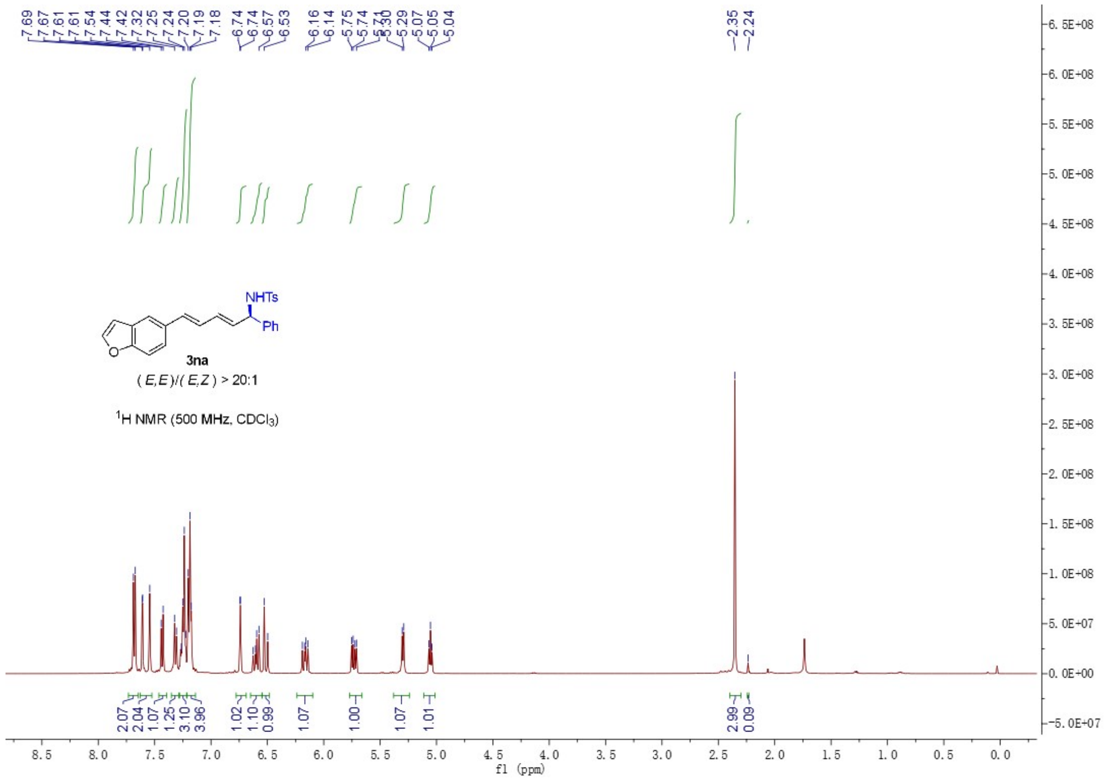
7.69 7.67 7.61 7.61 7.54 7.44 7.42 7.32 7.25 7.24 7.20 7.19 7.18 6.74 6.74 6.57 6.53 6.16 6.14 5.75 5.74 5.71 5.30 5.29 5.07 5.05 5.04 2.35 2.24
NHTs
Ph
3na
(E,E)/(E,Z) > 20:1
1H NMR (500 MHz, CDCl3)
2.07 2.04 1.07 1.25 3.10 3.96 1.02 1.10 0.99 1.07 1.00 1.07 1.01 2.99 0.09
f1 (ppm)

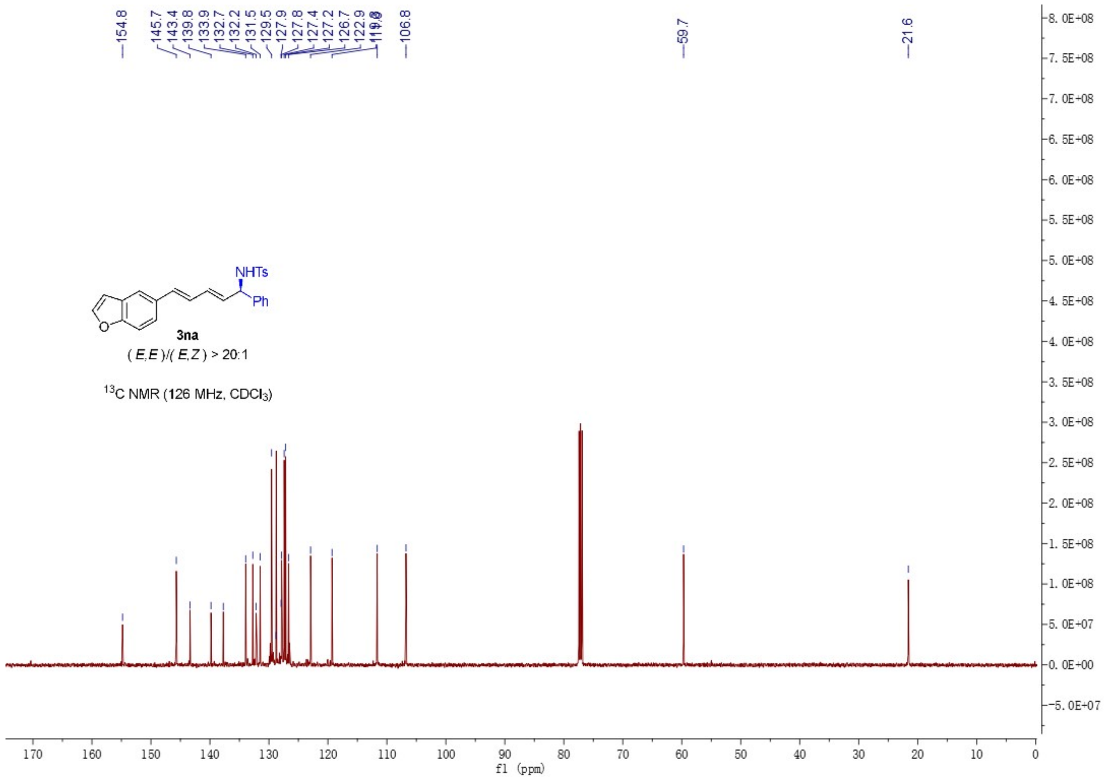
154.8 145.7 143.4 139.8 133.9 132.7 132.2 131.5 129.5 127.9 127.8 127.4 127.2 126.7 122.9 [illegible] 106.8 59.7 21.6
NHTs
Ph
3na
(E,E)/(E,Z) > 20:1
13C NMR (126 MHz, CDCl3)
f1 (ppm)

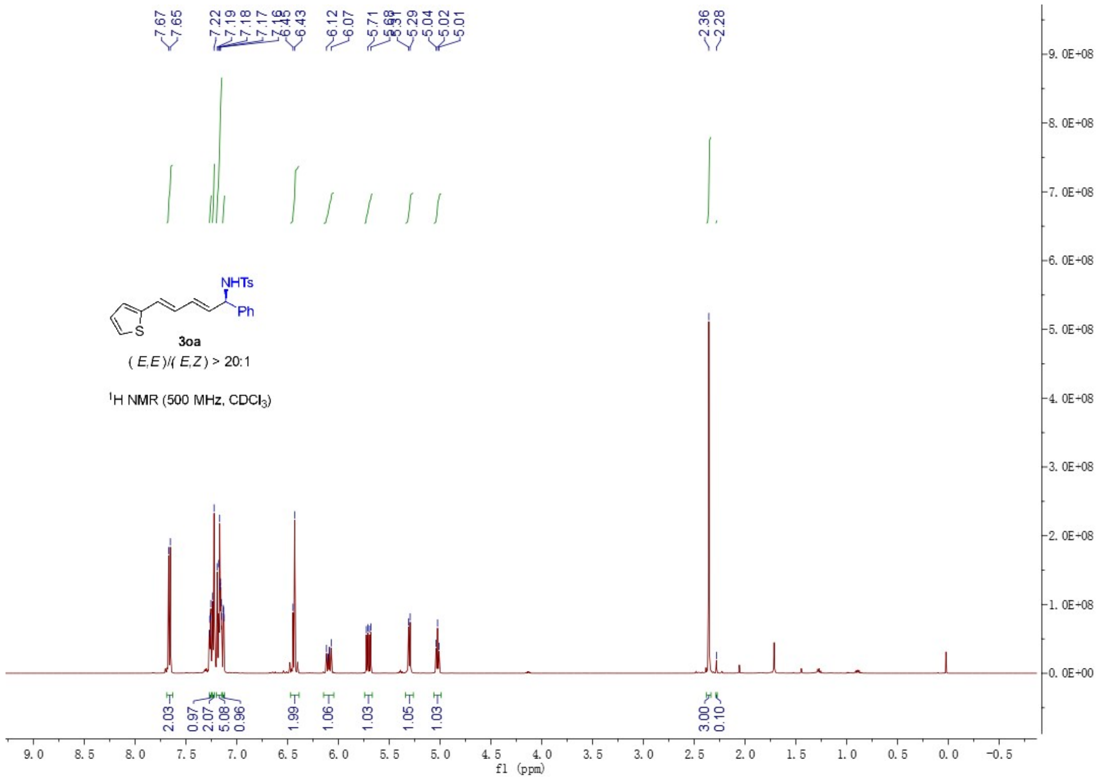
7.67
7.65
7.22
7.19
7.18
7.17
6.43
6.12
6.07
5.71
5.29
5.04
5.02
5.01
2.36
2.28
NHTs
Ph
S
3oa
(E,E)/(E,Z) > 20:1
1H NMR (500 MHz, CDCl3)
2.03
0.97
2.07
5.08
0.96
1.99
1.06
1.03
1.05
1.03
3.00
0.10
f1 (ppm)

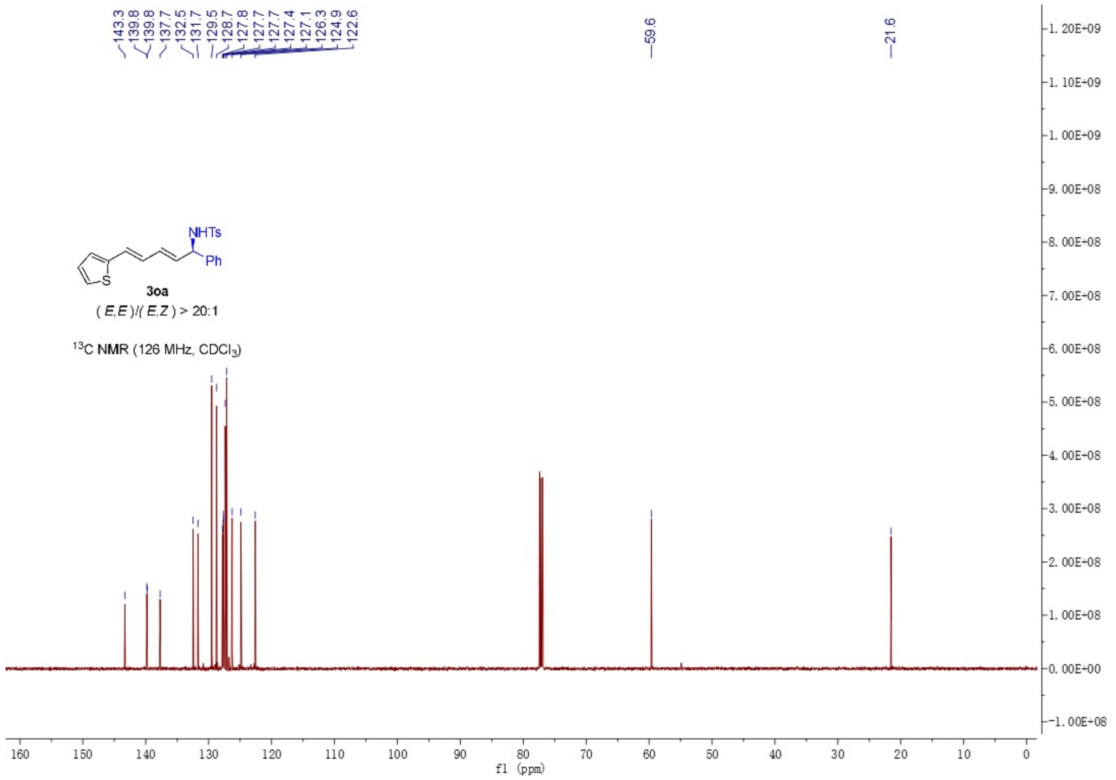
143.3
139.8
139.8
137.7
132.5
131.7
129.5
128.7
127.8
127.7
127.7
127.4
127.1
126.3
124.9
122.6
59.6
21.6
NHTs
Ph
S
3oa
(E,E)/(E,Z) > 20:1
13C NMR (126 MHz, CDCl3)
f1 (ppm)

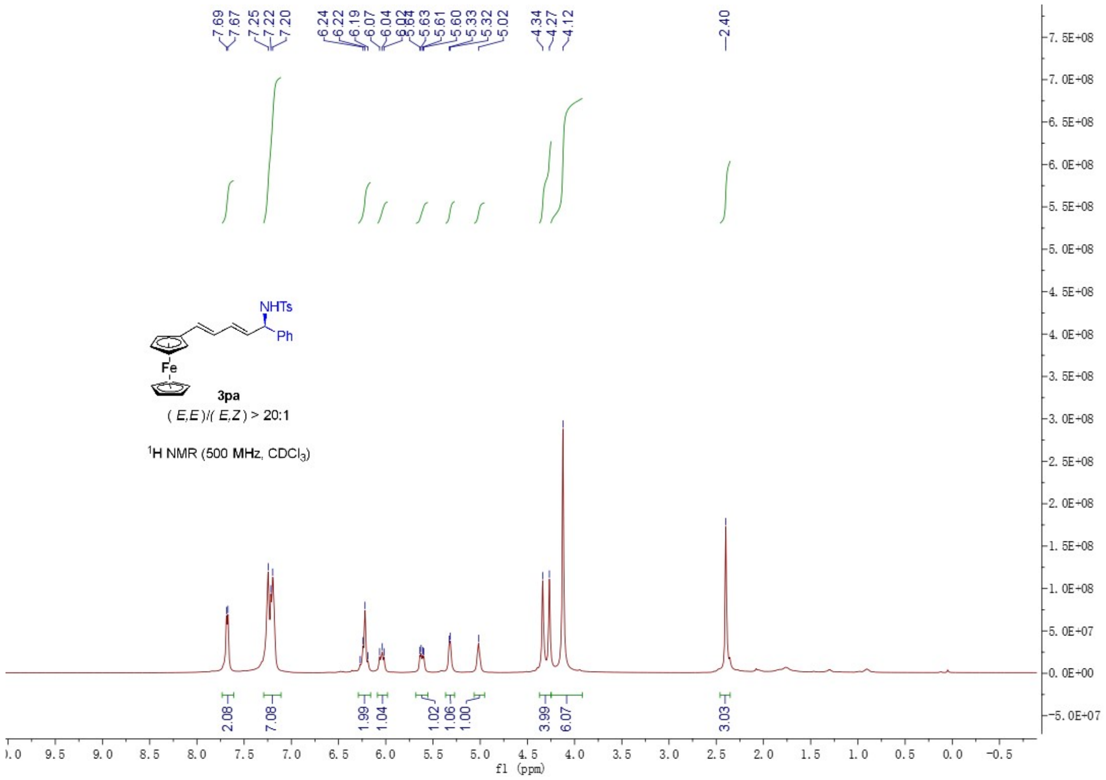
7.69
7.67
7.25
7.22
7.20
6.24
6.22
6.19
6.07
6.04
5.63
5.61
5.60
5.33
5.32
5.02
4.34
4.27
4.12
2.40
NHTs
Ph
Fe
3pa
(E,E)/(E,Z) > 20:1
1H NMR (500 MHz, CDCl3)
2.08
7.08
1.99
1.04
1.02
1.06
1.00
3.99
6.07
3.03
fl (ppm)

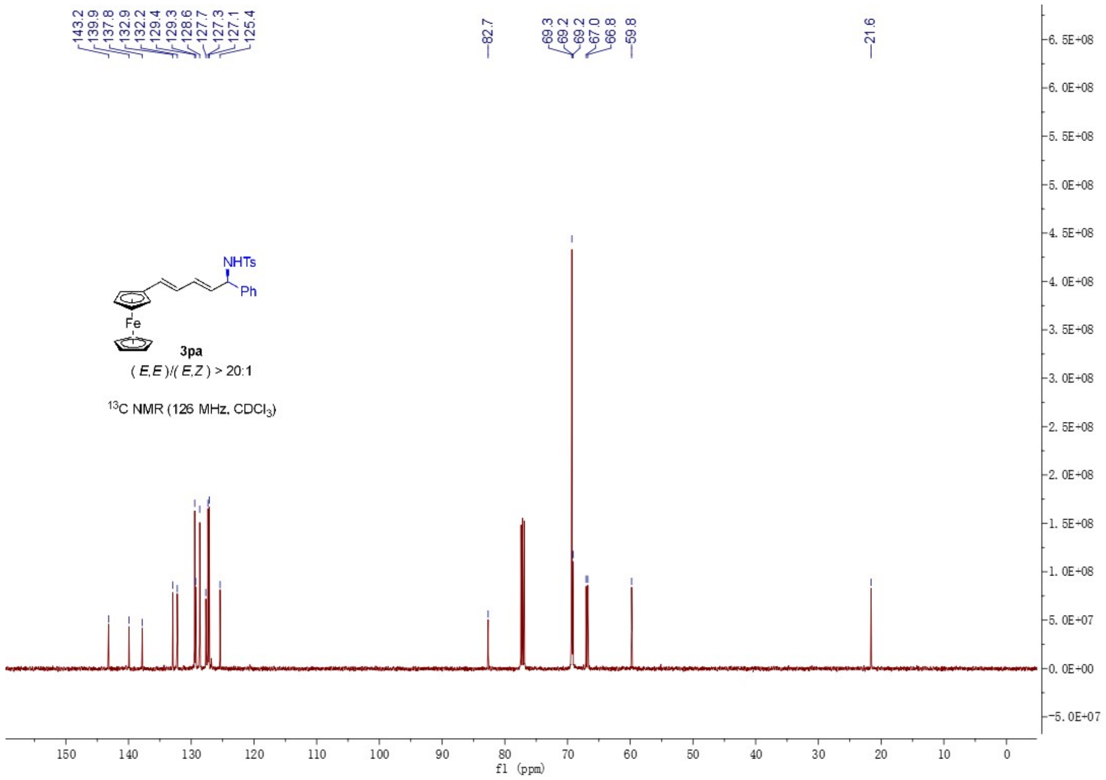
143.2
139.9
137.8
132.9
132.2
129.4
129.3
128.6
127.7
127.3
127.1
125.4
82.7
69.3
69.2
69.2
67.0
66.8
59.8
21.6
NHTs
Ph
Fe
3pa
(E,E)/(E,Z) > 20:1
13C NMR (126 MHz, CDCl3)
fl (ppm)

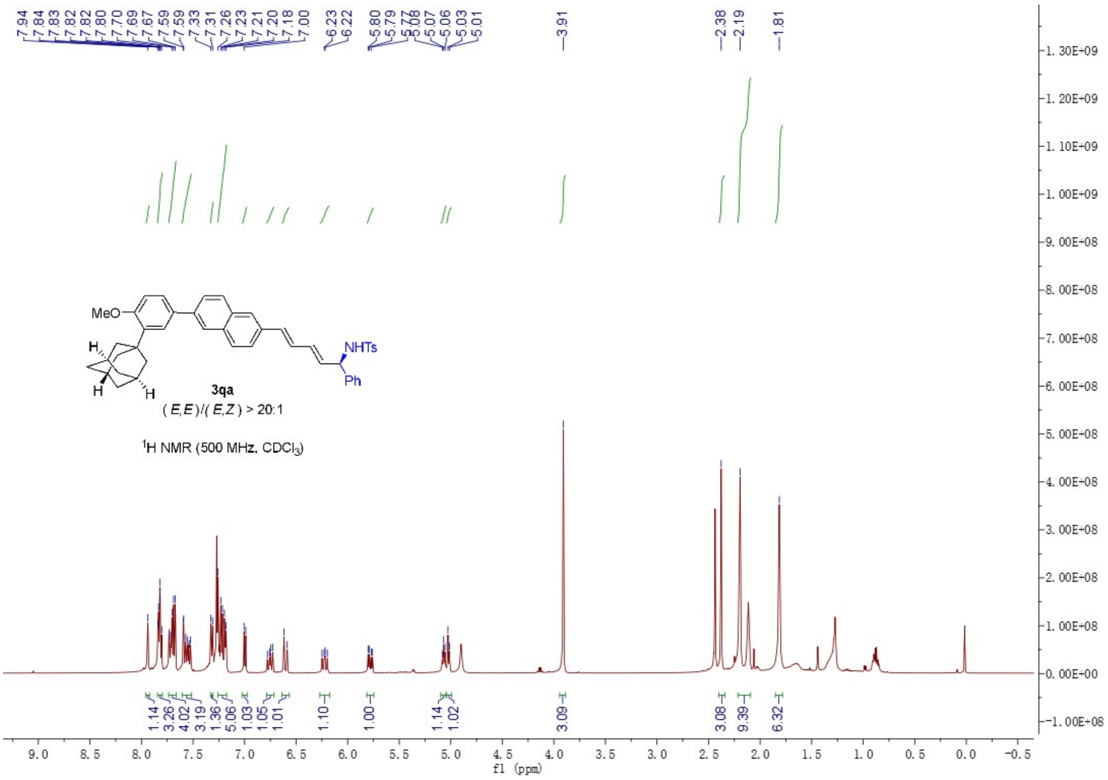
7.94 7.84 7.83 7.82 7.82 7.80 7.70 7.69 7.67 7.59 7.59 7.33 7.31 7.26 7.23 7.21 7.20 7.18 7.00 6.23 6.22 5.80 5.79 5.77 5.08 5.07 5.06 5.03 5.01
3.91
2.38 2.19 1.81
MeO
NHTs
Ph
3qa
(E,E)/(E,Z) > 20:1
1H NMR (500 MHz, CDCl3)
1.14 3.26 4.02 3.19 1.36 5.06 1.03 1.05 1.01 1.10 1.00 1.14 1.02 3.09 3.08 9.39 6.32
f1 (ppm)

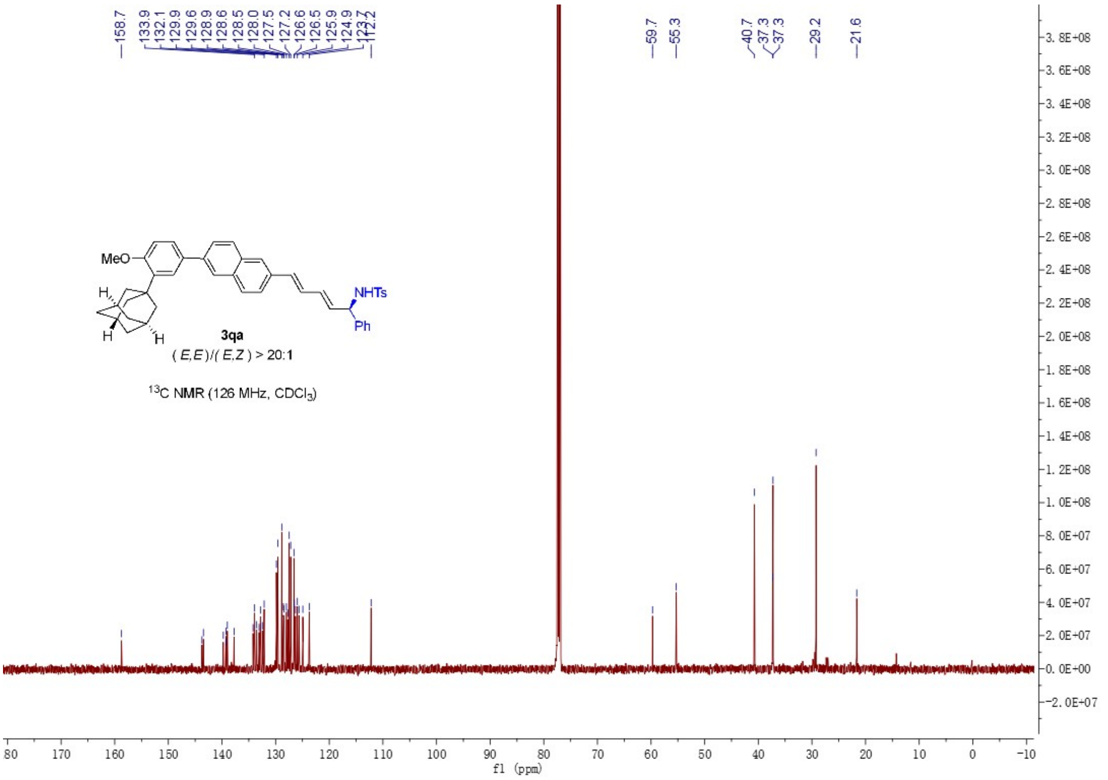
158.7 133.9 132.1 129.9 129.6 128.9 128.6 128.5 128.0 127.5 127.2 126.6 126.5 125.9 124.9 123.7 112.2
59.7 55.3 40.7 37.3 37.3 29.2 21.6
MeO
NHTs
Ph
3qa
(E,E)/(E,Z) > 20:1
13C NMR (126 MHz, CDCl3)
f1 (ppm)

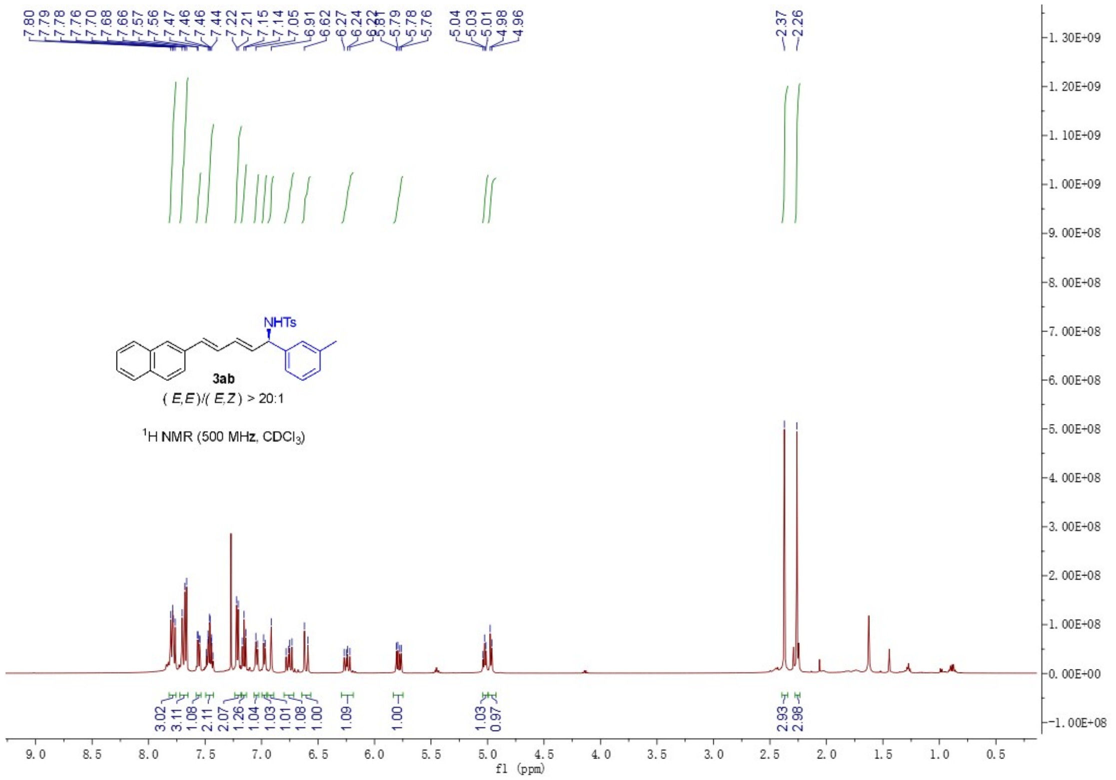
NHTs
3ab
(E,E)/(E,Z) > 20:1
1H NMR (500 MHz, CDCl3)
7.80 7.79 7.78 7.76 7.70 7.68 7.66 7.57 7.56 7.47 7.46 7.46 7.44 7.22 7.21 7.15 7.14 7.05 6.91 6.62 6.27 6.24 5.81 5.79 5.78 5.76 5.04 5.03 5.01 4.98 4.96
2.37 2.26
3.02 3.11 1.08 2.11 2.07 1.26 1.04 1.03 1.01 1.08 1.00 1.09 1.00 1.03 0.97 2.93 2.98
f1 (ppm)

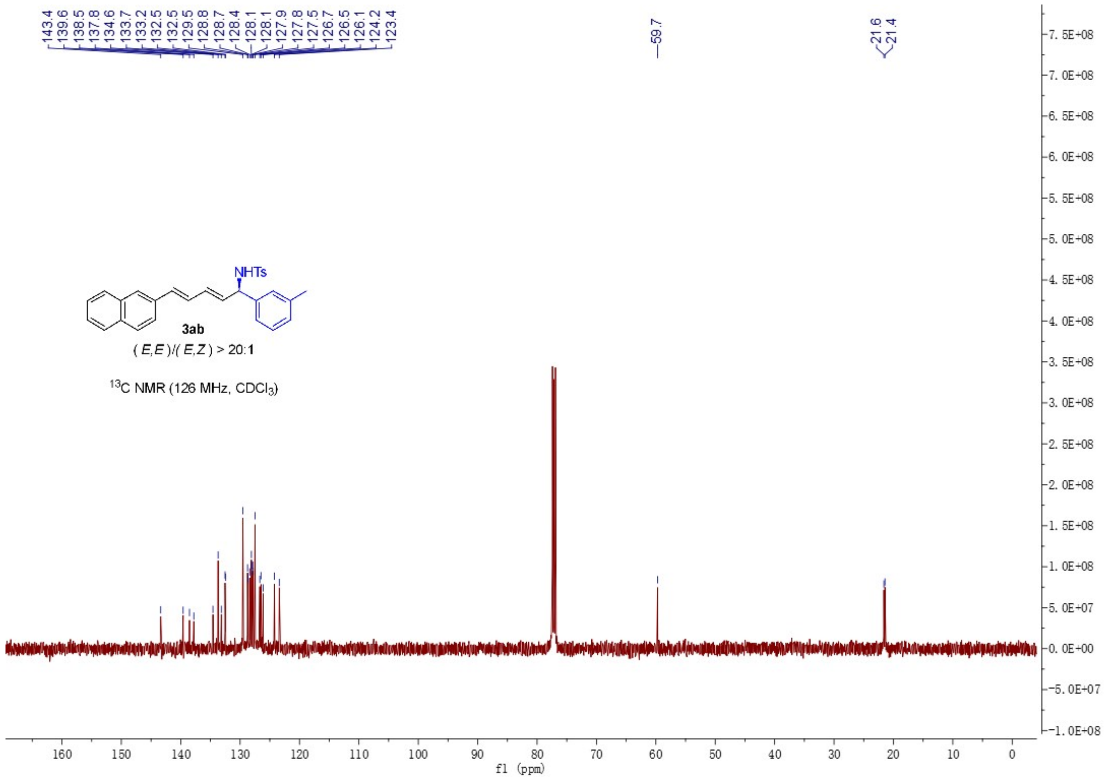
143.4 139.6 138.5 137.8 134.6 133.7 133.2 132.5 129.5 128.8 128.7 128.4 128.1 128.1 127.9 127.8 127.5 126.7 126.5 126.1 124.2 123.4
59.7
21.6 21.4
NHTs
3ab
(E,E)/(E,Z) > 20:1
13C NMR (126 MHz, CDCl3)
f1 (ppm)

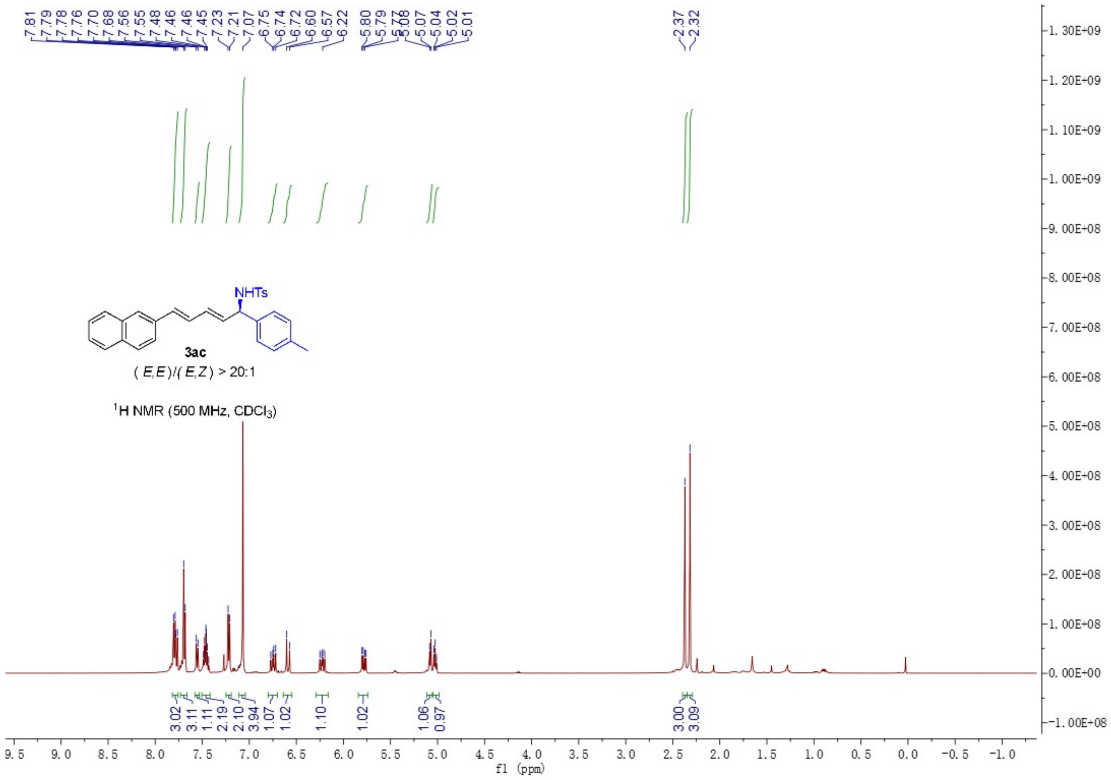

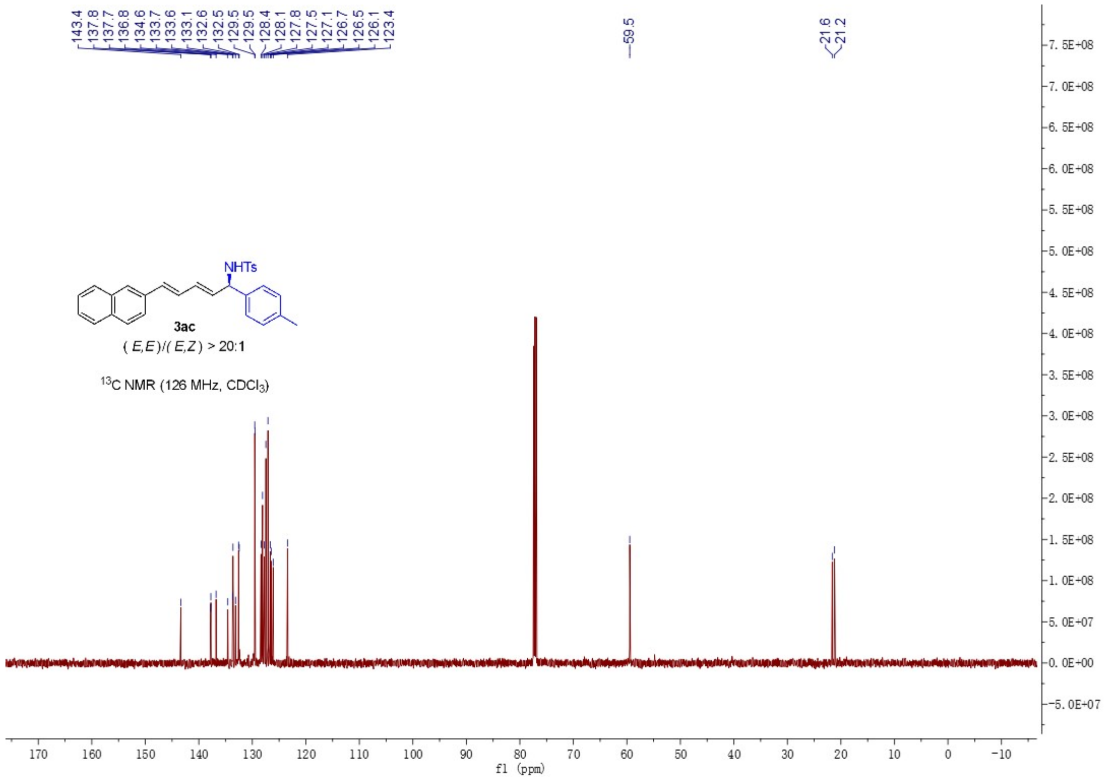

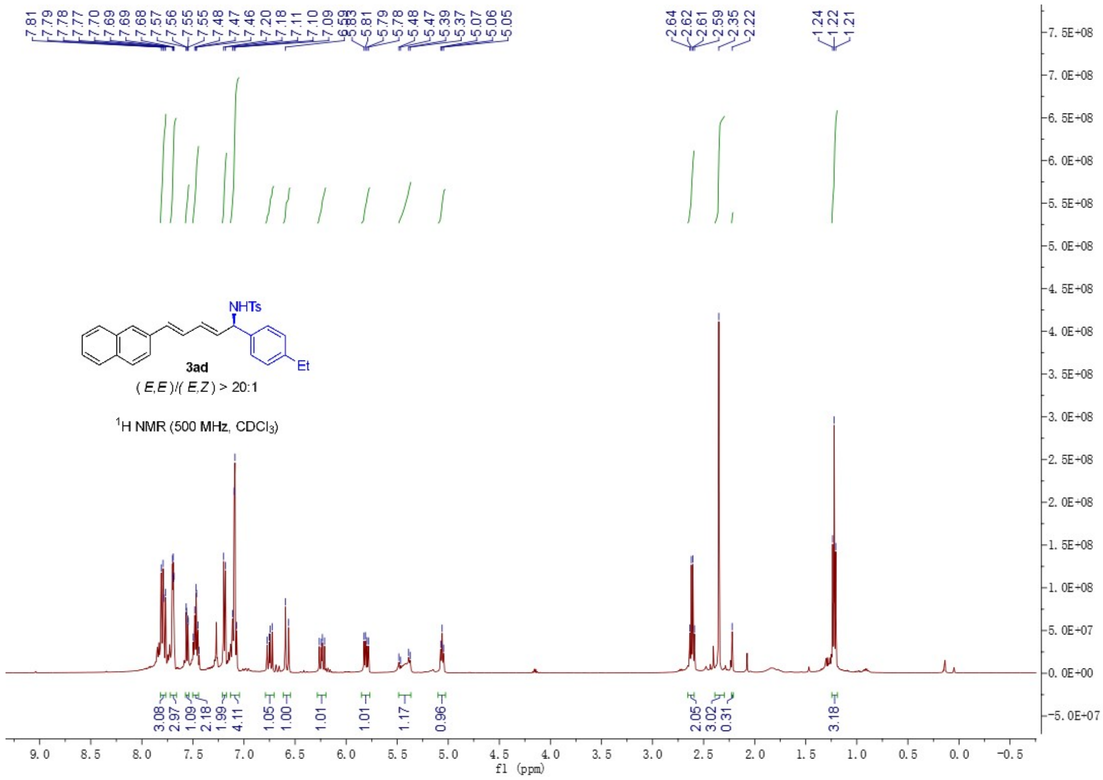
NHTs
Et
3ad
(E,E)/(E,Z) > 20:1
1H NMR (500 MHz, CDCl3)
7.81 7.79 7.78 7.77 7.70 7.69 7.69 7.68 7.57 7.56 7.55 7.55 7.48 7.47 7.46 7.20 7.18 7.11 7.10 7.09 5.81 5.79 5.78 5.48 5.47 5.39 5.37 5.07 5.06 5.05
2.64 2.62 2.61 2.59 2.35 2.22
1.24 1.22 1.21
3.08 2.97 1.09 2.18 1.99 4.11 1.05 1.00 1.01 1.01 1.17 0.96 2.05 3.02 0.31 3.18
f1 (ppm)

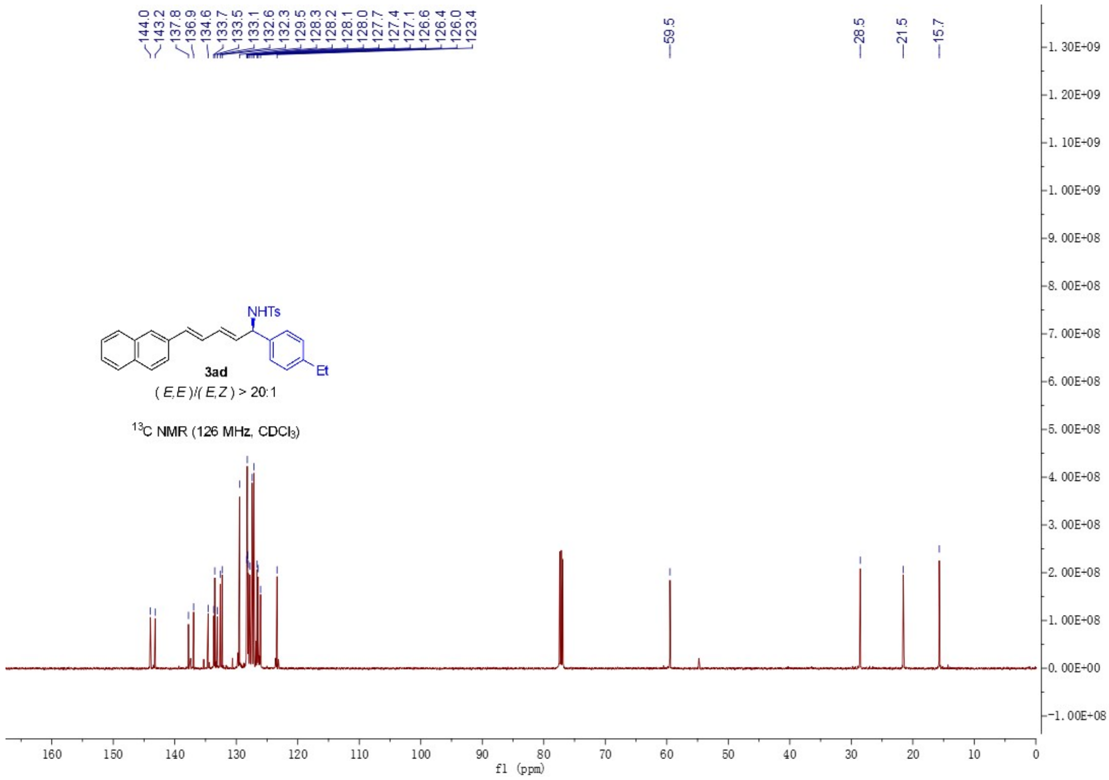
NHTs
Et
3ad
(E,E)/(E,Z) > 20:1
13C NMR (126 MHz, CDCl3)
144.0 143.2 137.8 136.9 134.6 133.7 133.5 133.1 132.6 132.3 129.5 128.3 128.2 128.1 128.0 127.7 127.4 127.1 126.6 126.4 126.0 123.4
59.5
28.5 21.5 15.7
f1 (ppm)

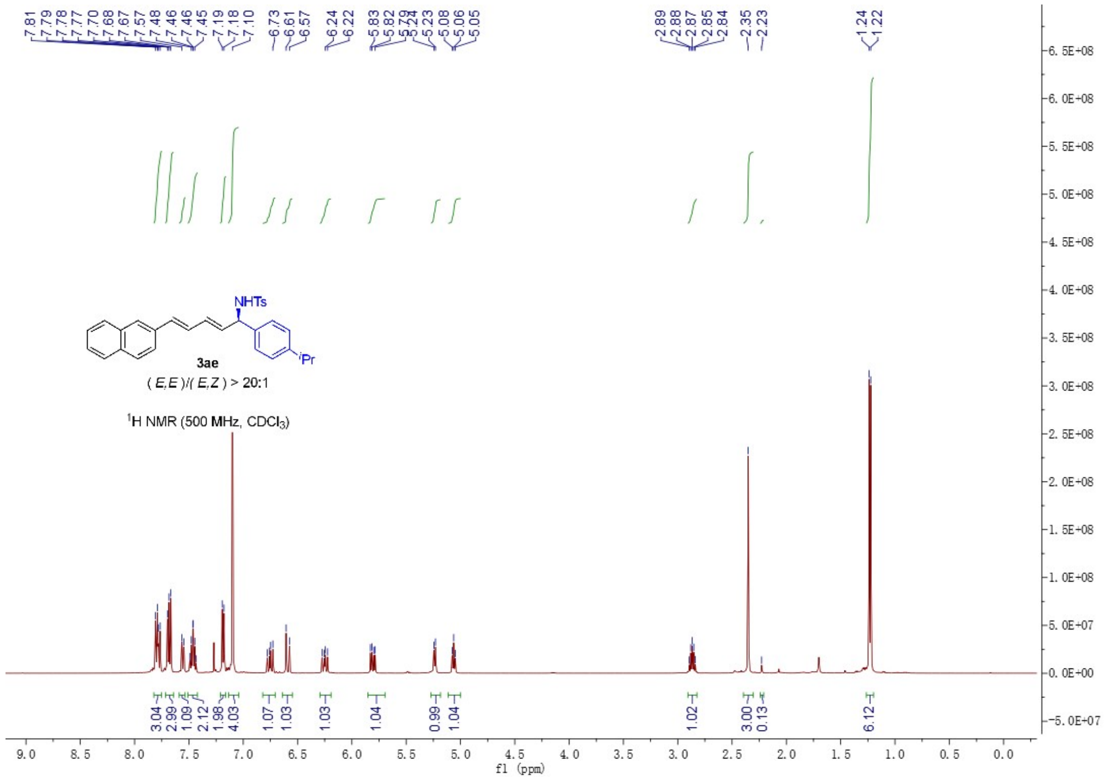
3ae
(E,E)/(E,Z) > 20:1
^{1}H NMR (500 MHz, CDCl$_3$)
f1 (ppm)

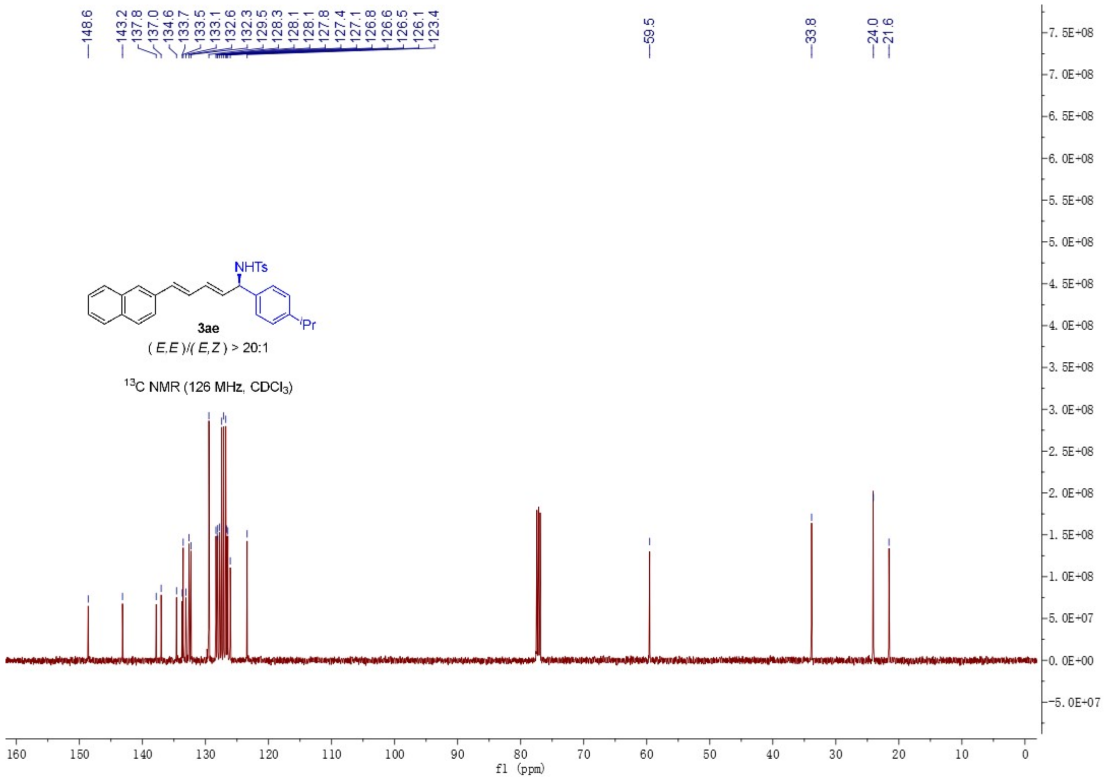
3ae
(E,E)/(E,Z) > 20:1
^{13}C NMR (126 MHz, CDCl$_3$)
f1 (ppm)

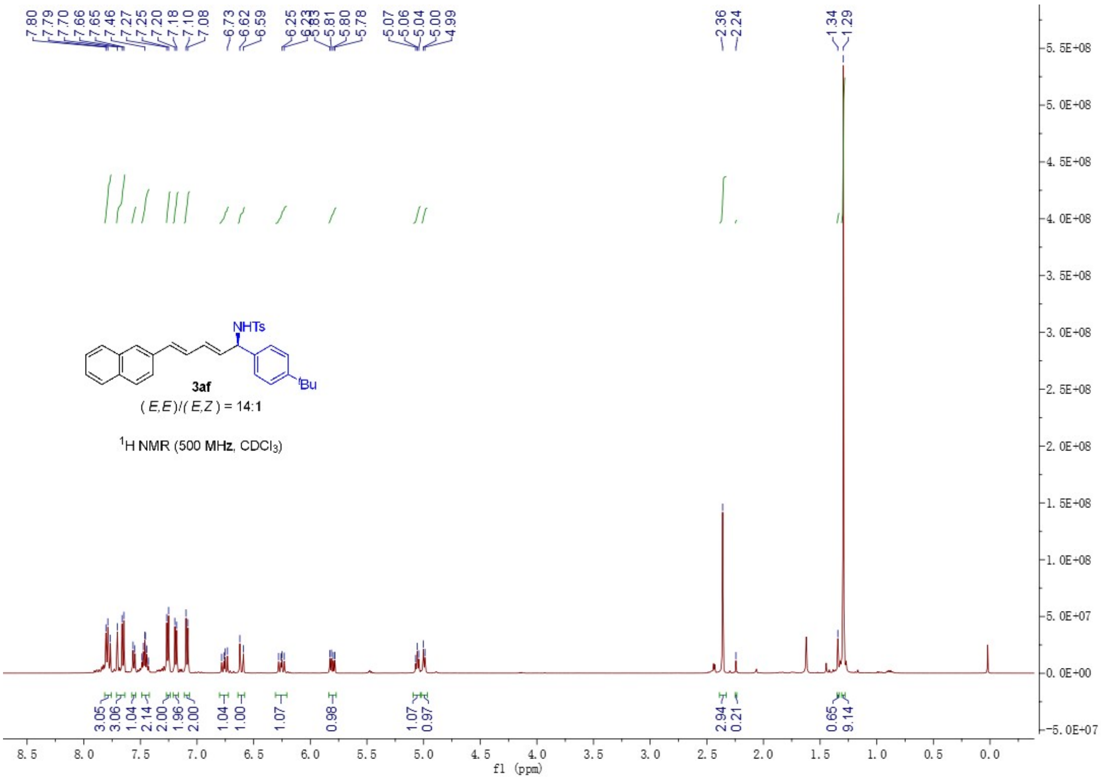
7.80 7.79 7.70 7.66 7.65 7.46 7.27 7.25 7.20 7.18 7.10 7.08 6.73 6.62 6.59 6.25 6.23 5.83 5.81 5.80 5.78 5.07 5.06 5.04 5.00 4.99
2.36 2.24
1.34 1.29
NHTs
tBu
3af
(E,E)/(E,Z) = 14:1
1H NMR (500 MHz, CDCl3)
3.05 3.06 1.04 2.14 2.00 1.96 2.00 1.04 1.00 1.07 0.98 1.07 0.97 2.94 0.21 0.65 9.14
fl (ppm)

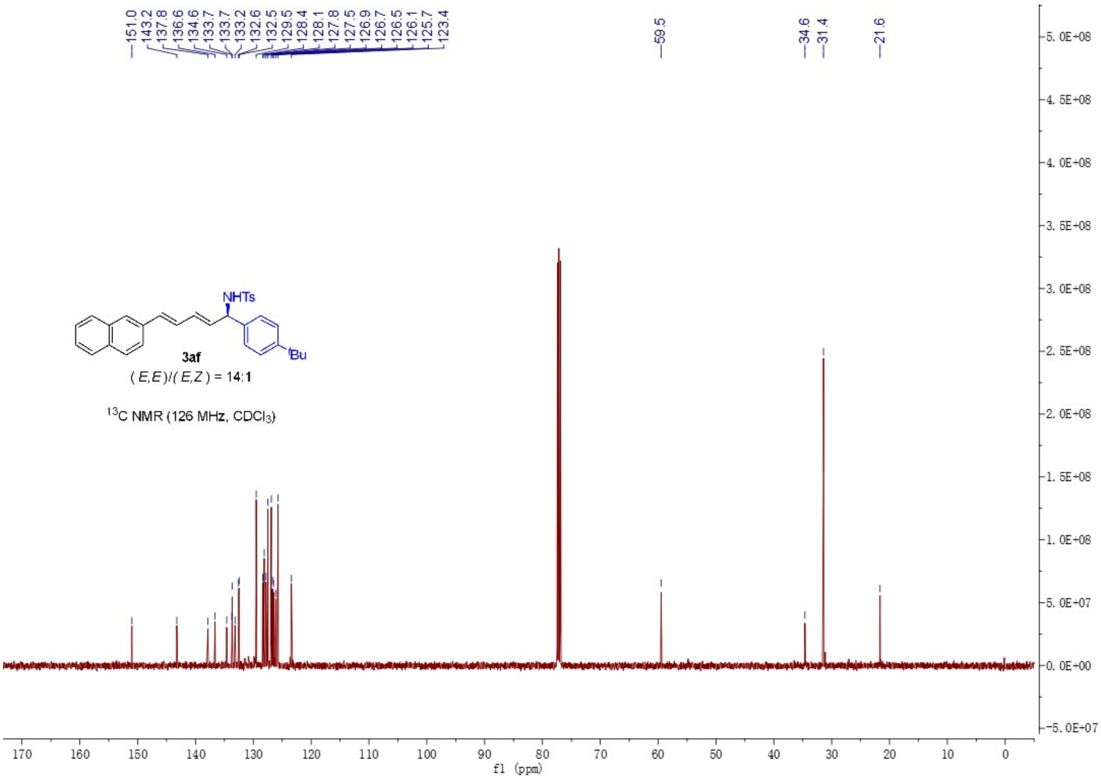
151.0 143.2 137.8 136.6 134.6 133.7 133.2 132.6 132.5 129.5 128.4 128.1 127.8 127.5 126.9 126.7 126.5 126.1 125.7 123.4
59.5
34.6 31.4
21.6
NHTs
tBu
3af
(E,E)/(E,Z) = 14:1
13C NMR (126 MHz, CDCl3)
fl (ppm)

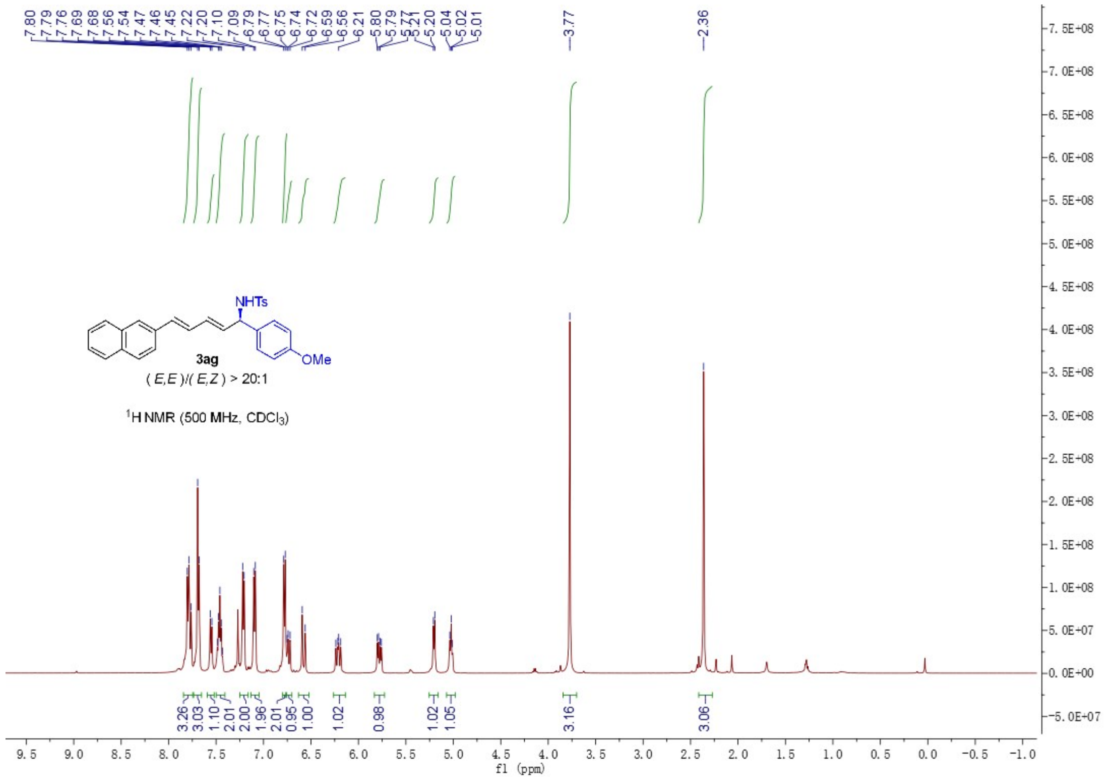
NHTs
OMe
3ag
(E,E)/(E,Z) > 20:1
1H NMR (500 MHz, CDCl3)
fl (ppm)

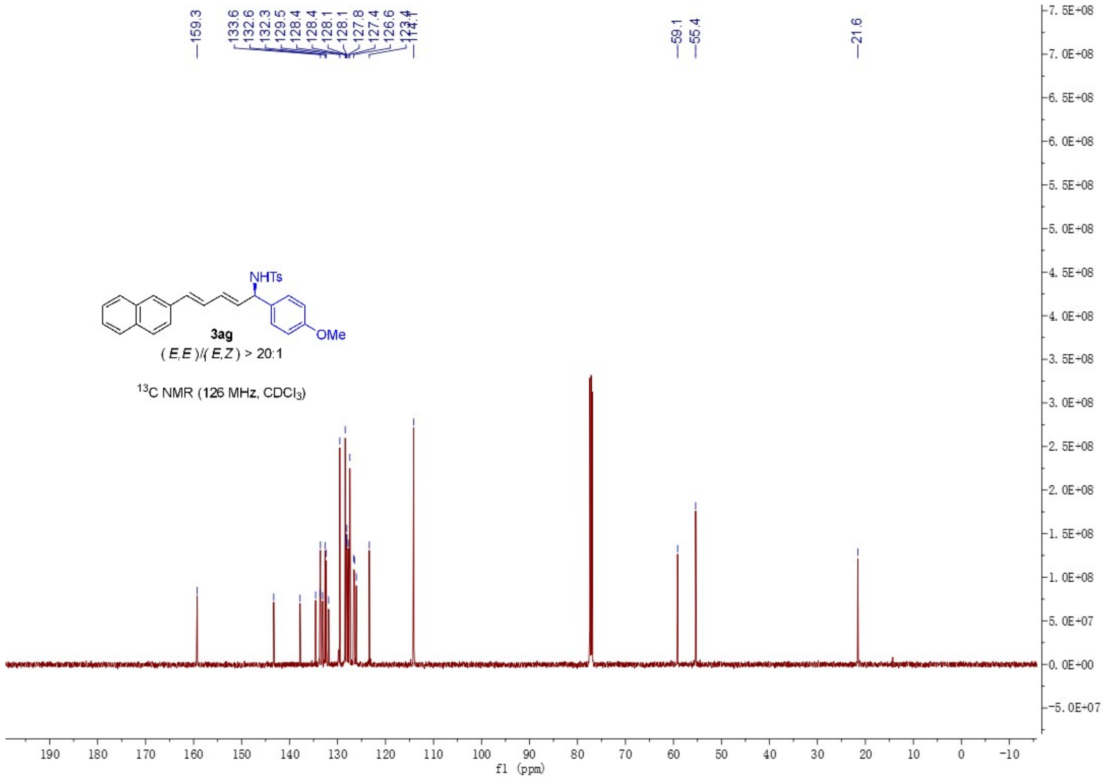
NHTs
OMe
3ag
(E,E)/(E,Z) > 20:1
13C NMR (126 MHz, CDCl3)
fl (ppm)

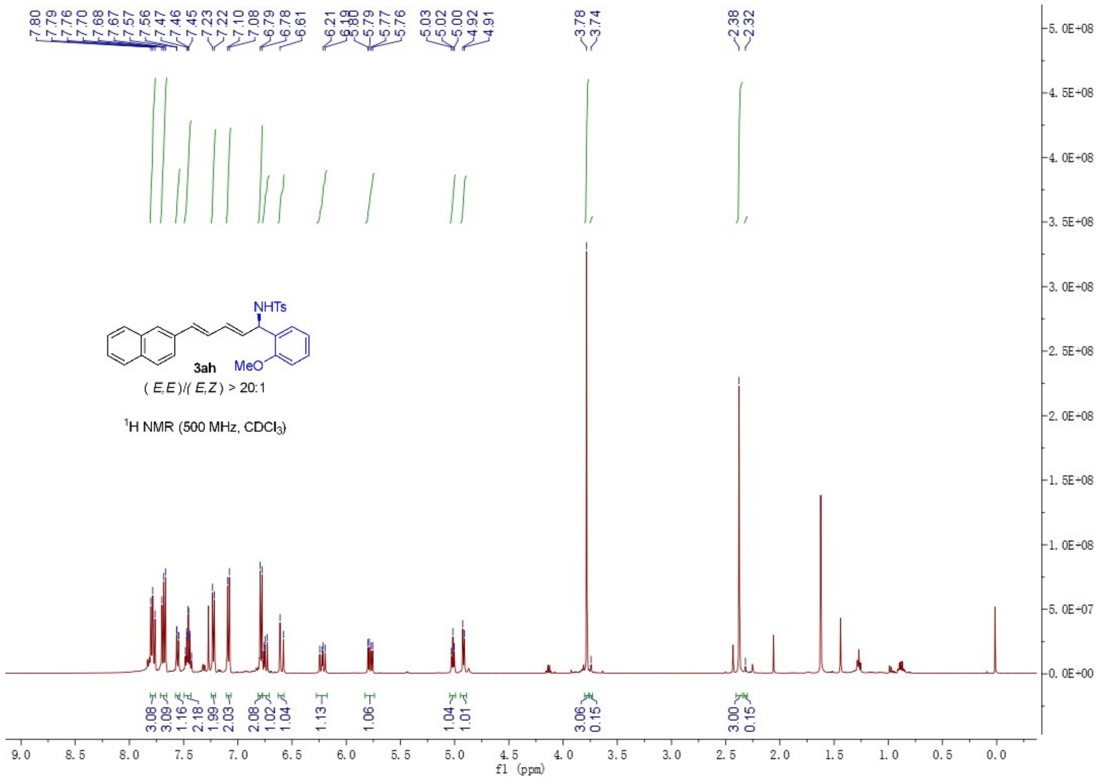
3ah
(E,E)/(E,Z) > 20:1
1H NMR (500 MHz, CDCl3)
f1 (ppm)

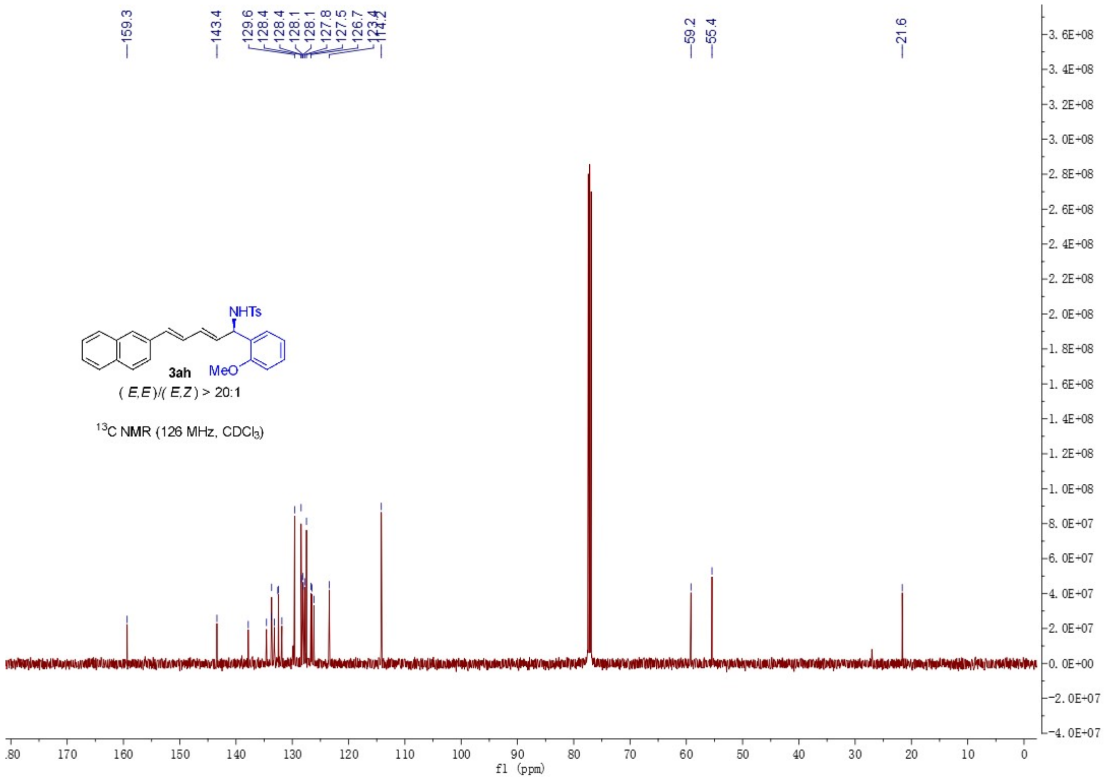
3ah
(E,E)/(E,Z) > 20:1
13C NMR (126 MHz, CDCl3)
f1 (ppm)

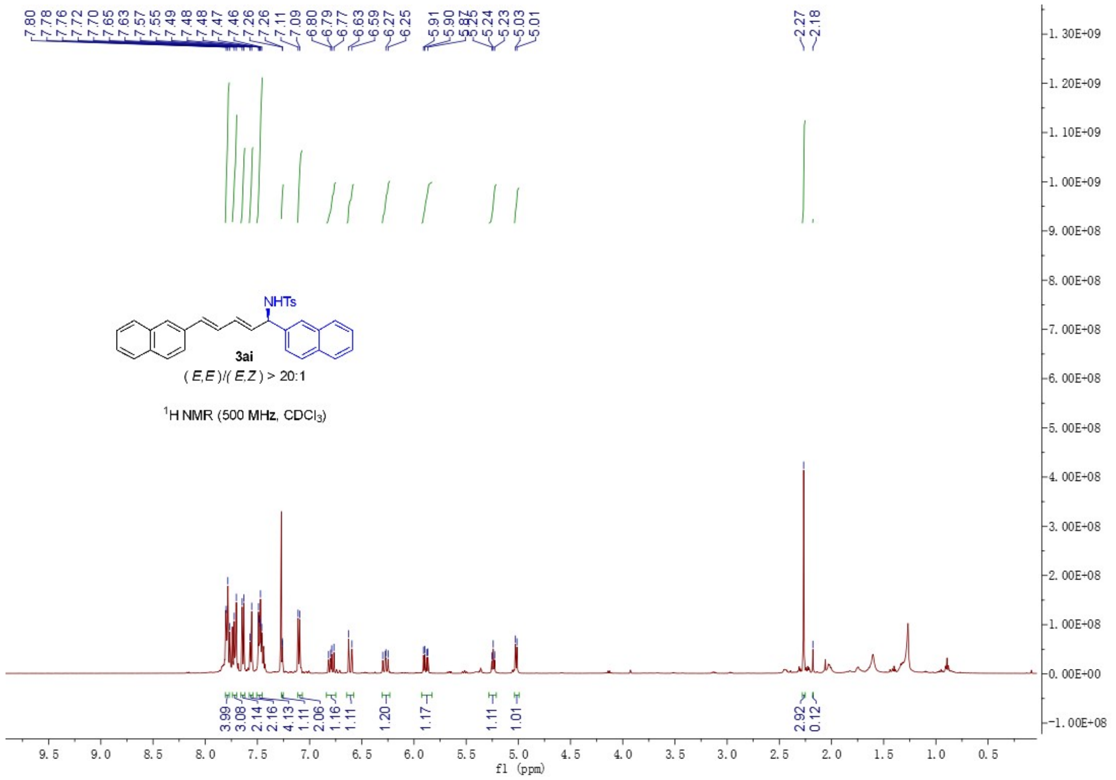

7.80
7.78
7.76
7.72
7.70
7.65
7.63
7.57
7.55
7.49
7.48
7.47
7.46
7.26
7.26
7.11
7.09
6.80
6.79
6.77
6.63
6.59
6.27
6.25
5.91
5.90
5.87
5.25
5.24
5.23
5.03
5.01
2.27
2.18
NHTs
3ai
(E,E)/(E,Z) > 20:1
1H NMR (500 MHz, CDCl3)
3.99
3.08
2.14
2.16
4.13
1.11
2.06
1.16
1.11
1.20
1.17
1.11
1.01
2.92
0.12
f1 (ppm)

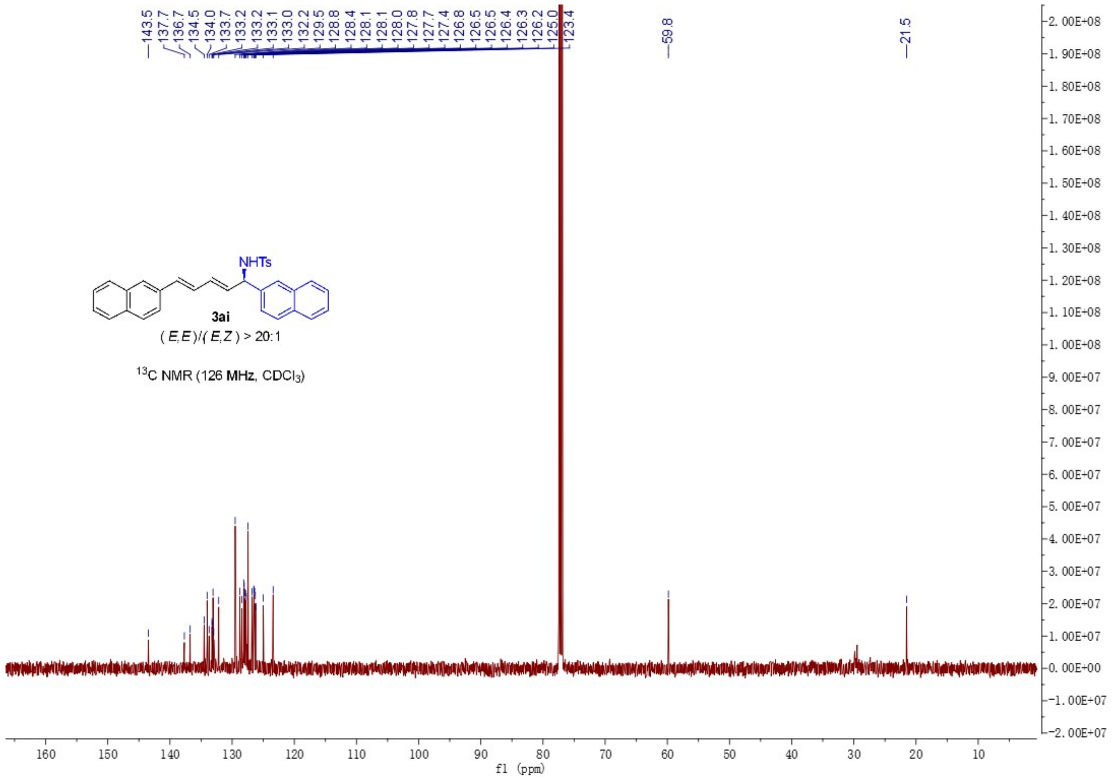

143.5
137.7
136.7
134.5
134.0
133.7
133.2
133.1
133.0
132.2
129.5
128.8
128.4
128.1
128.0
127.8
127.7
127.4
126.8
126.5
126.4
126.3
126.2
125.0
123.4
59.8
21.5
NHTs
3ai
(E,E)/(E,Z) > 20:1
13C NMR (126 MHz, CDCl3)
f1 (ppm)

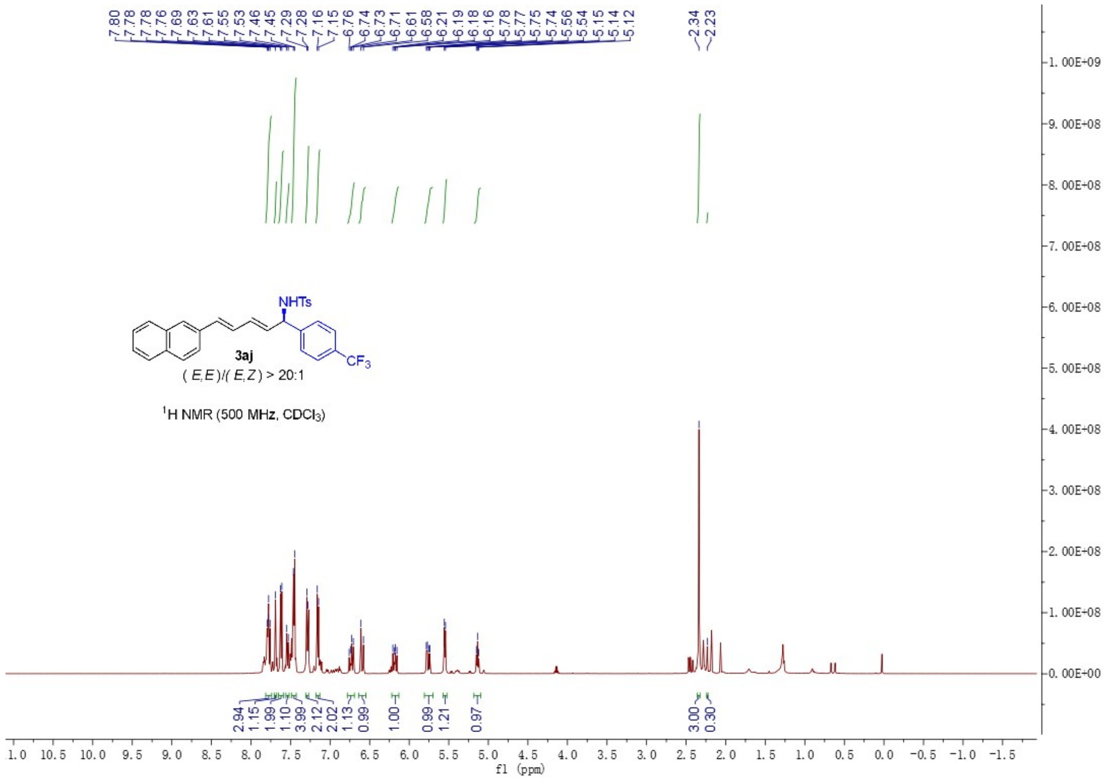
7.80 7.78 7.76 7.69 7.63 7.61 7.55 7.53 7.46 7.45 7.29 7.28 7.16 7.15 6.76 6.74 6.73 6.71 6.61 6.58 6.21 6.19 6.18 6.16 5.78 5.77 5.75 5.74 5.56 5.54 5.15 5.14 5.12
2.34 2.23
NHTs
3aj
(E,E)/(E,Z) > 20:1
CF3
1H NMR (500 MHz, CDCl3)
2.94 1.15 1.99 1.10 3.99 2.12 2.02 1.13 0.99 1.00 0.99 1.21 0.97
3.00 0.30
f1 (ppm)

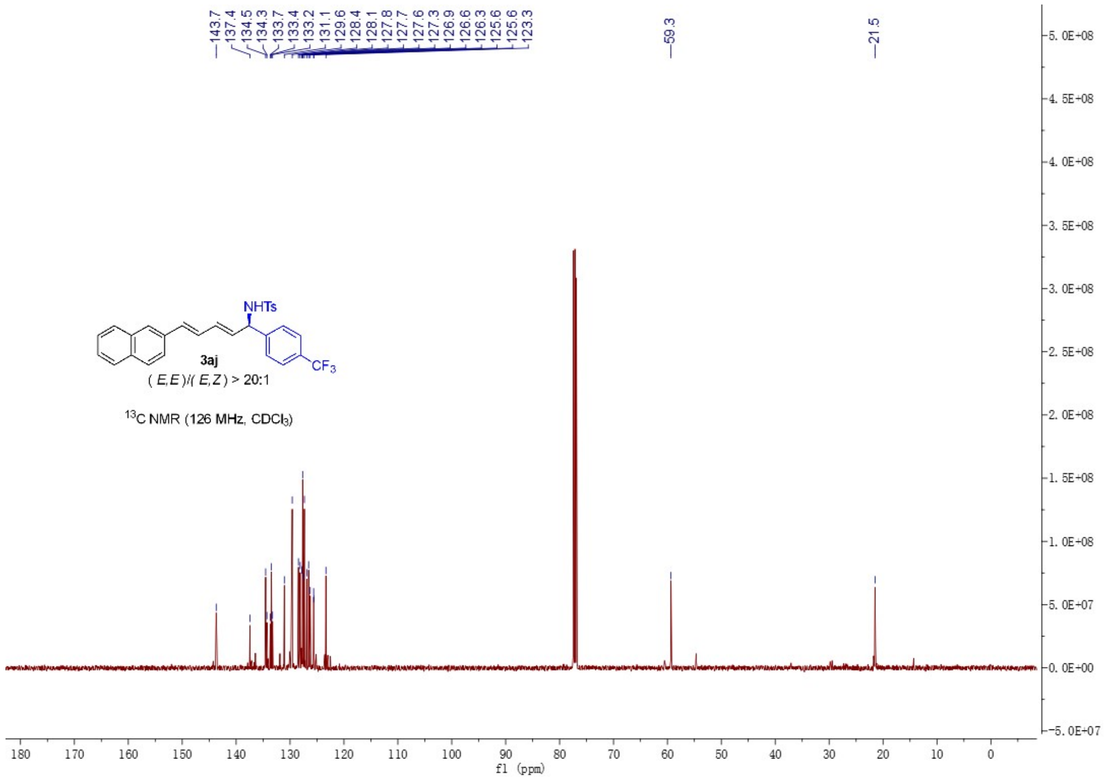
143.7 137.4 134.5 134.3 133.7 133.4 133.2 131.1 129.6 128.4 128.1 127.8 127.7 127.6 127.3 126.9 126.6 126.3 125.6 123.3
59.3
21.5
NHTs
3aj
(E,E)/(E,Z) > 20:1
CF3
13C NMR (126 MHz, CDCl3)
f1 (ppm)

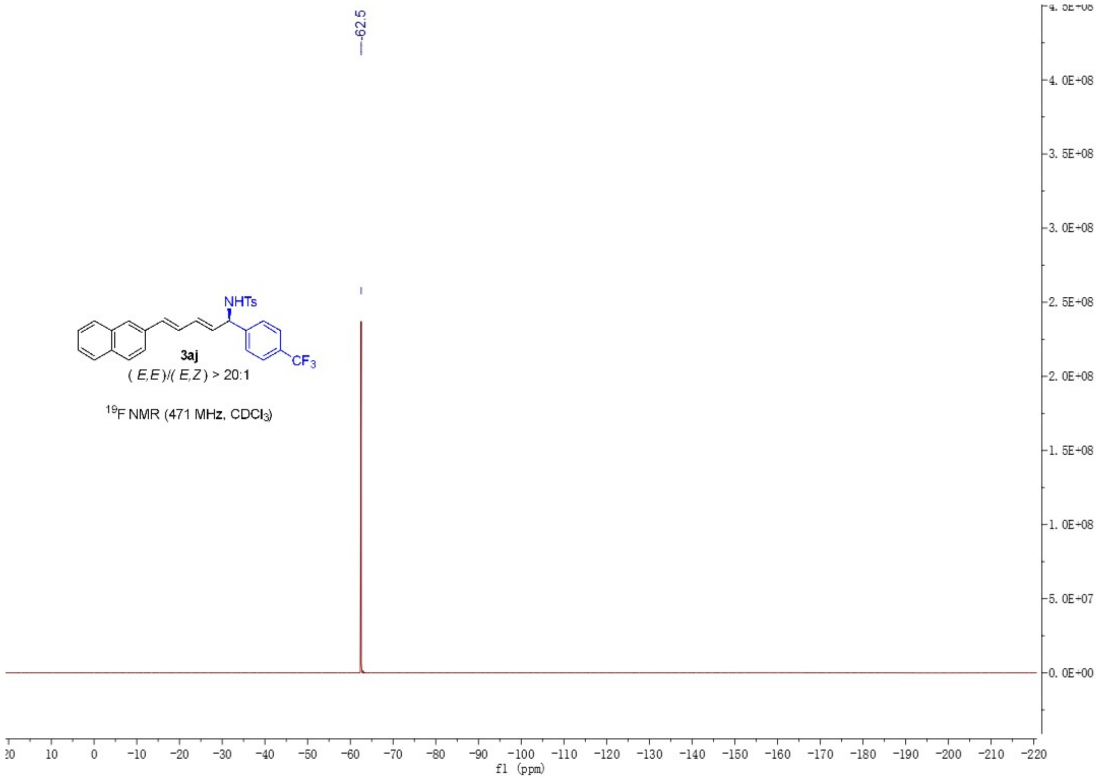
-62.5
NHTs
CF3
3aj
(E,E)/(E,Z) > 20:1
19F NMR (471 MHz, CDCl3)
f1 (ppm)

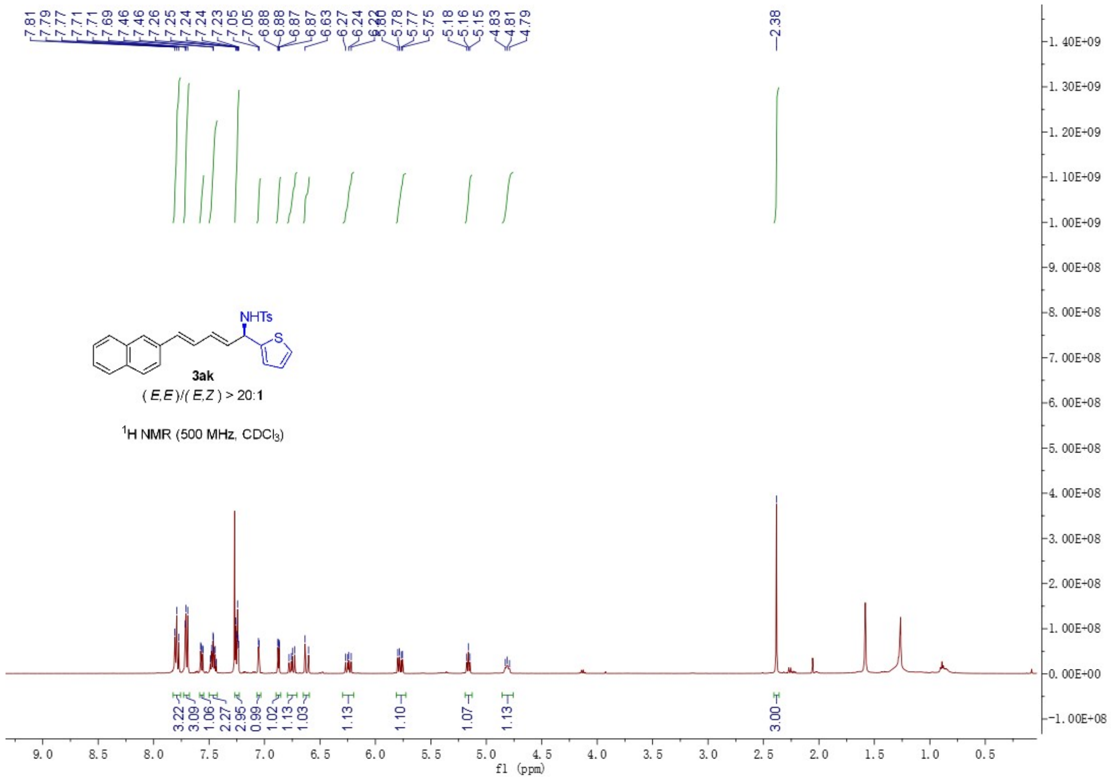
7.81 7.79 7.77 7.71 7.71 7.69 7.46 7.46 7.26 7.25 7.24 7.24 7.23 7.05 7.05 6.88 6.88 6.87 6.87 6.63 6.27 6.24 6.22 5.80 5.78 5.77 5.75 5.18 5.16 5.15 4.83 4.81 4.79
2.38
NHTs
S
3ak
(E,E)/(E,Z) > 20:1
1H NMR (500 MHz, CDCl3)
3.22 3.09 1.06 2.27 2.95 0.99 1.02 1.13 1.03 1.13 1.10 1.07 1.13 3.00
f1 (ppm)

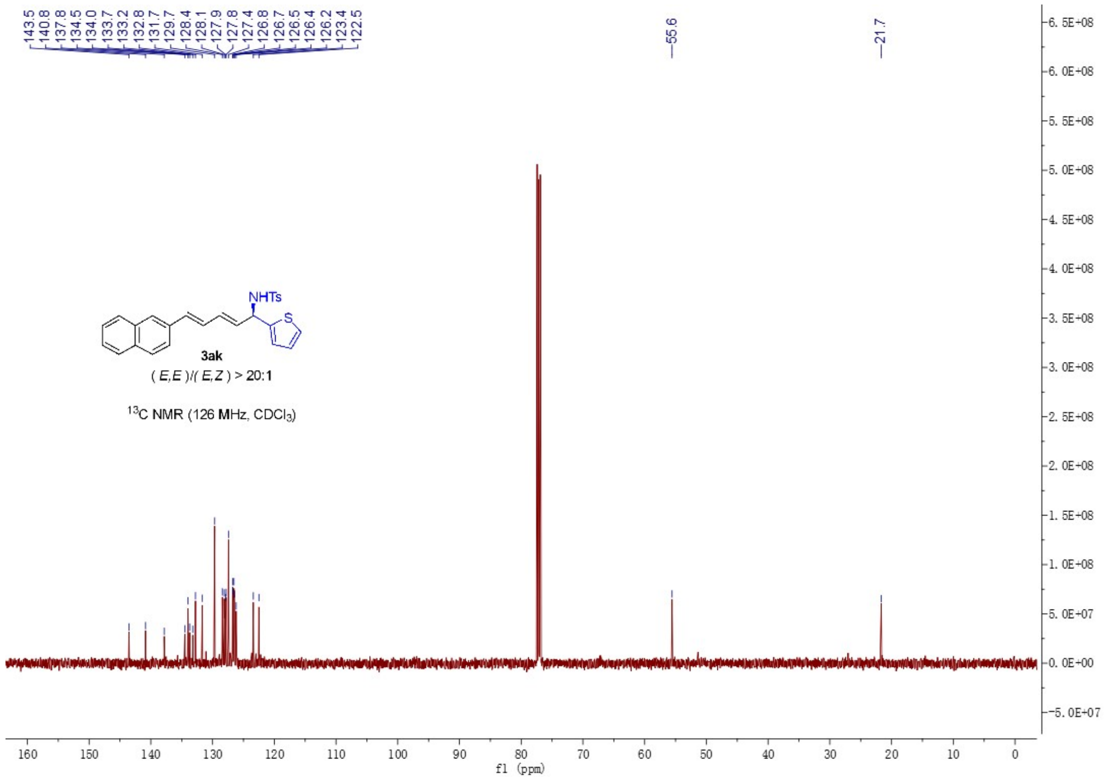
NHTs
3ak
(E,E)/(E,Z) > 20:1
13C NMR (126 MHz, CDCl3)
fl (ppm)

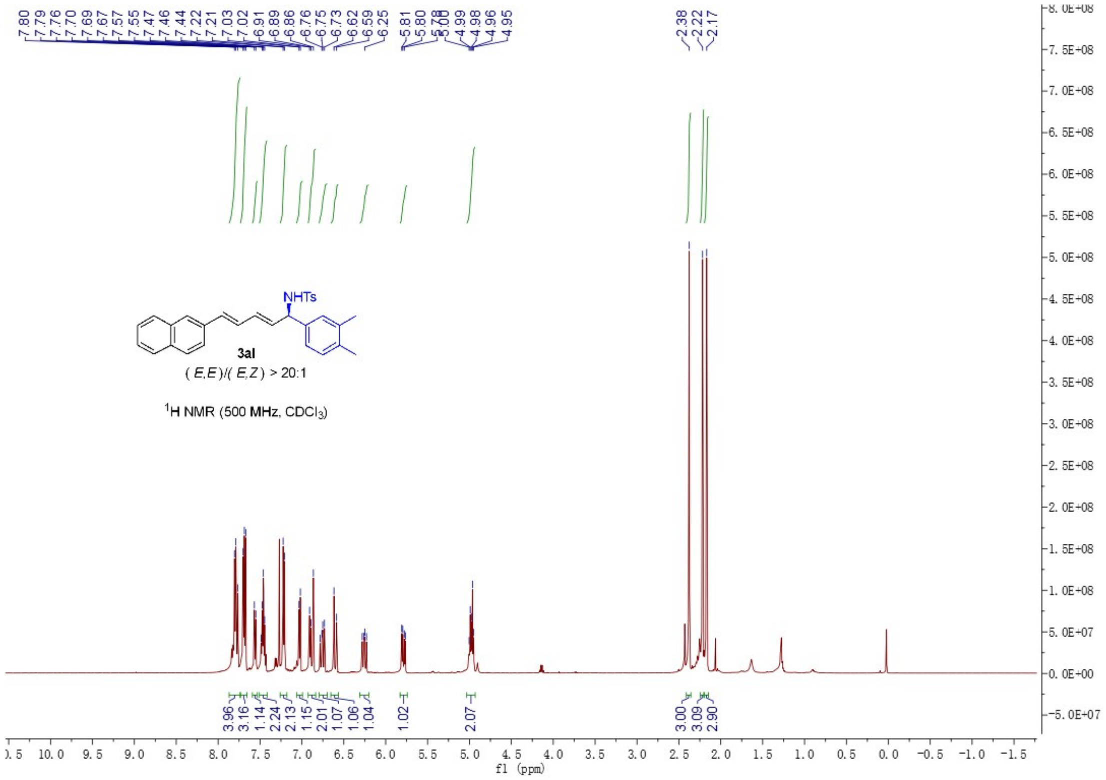
NHTs
3al
(E,E)/(E,Z) > 20:1
1H NMR (500 MHz, CDCl3)
fl (ppm)

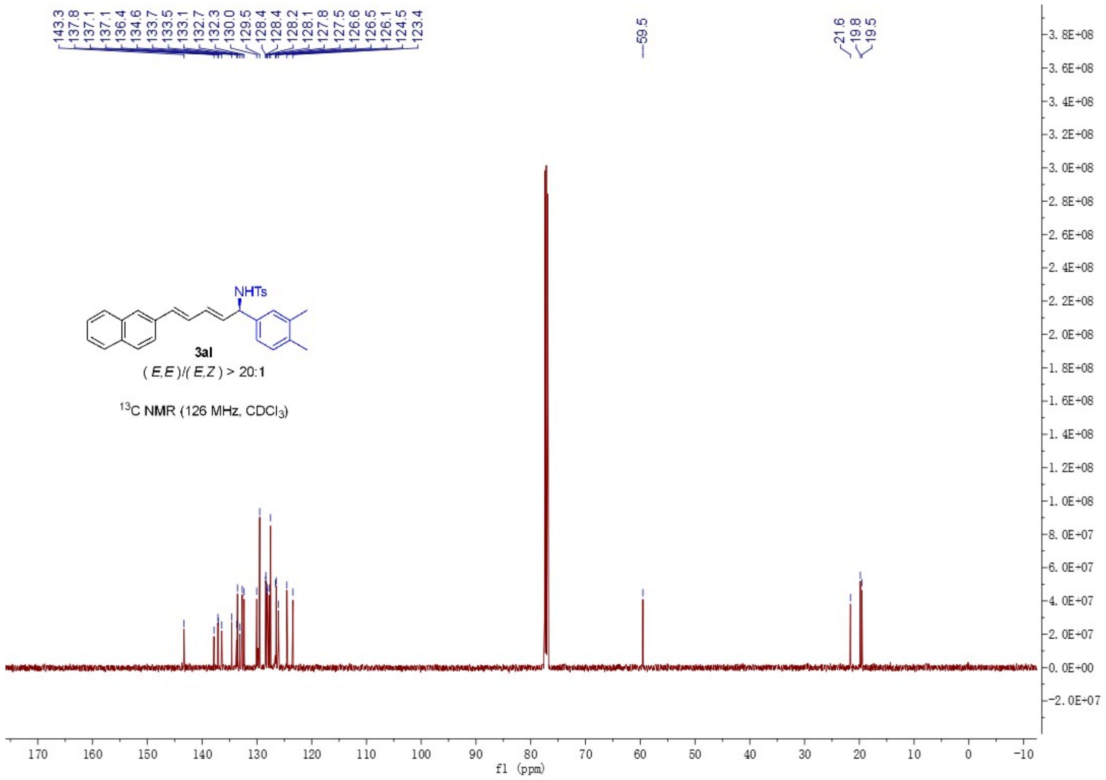
143.3
137.8
137.1
136.4
134.6
133.7
133.5
133.1
132.7
132.3
130.0
129.5
128.4
128.4
128.2
128.1
127.8
127.5
126.6
126.5
126.1
124.5
123.4
59.5
21.6
19.8
19.5
NHTs
3al
(E,E)/(E,Z) > 20:1
^{13}C NMR (126 MHz, CDCl$_3$)
f1 (ppm)

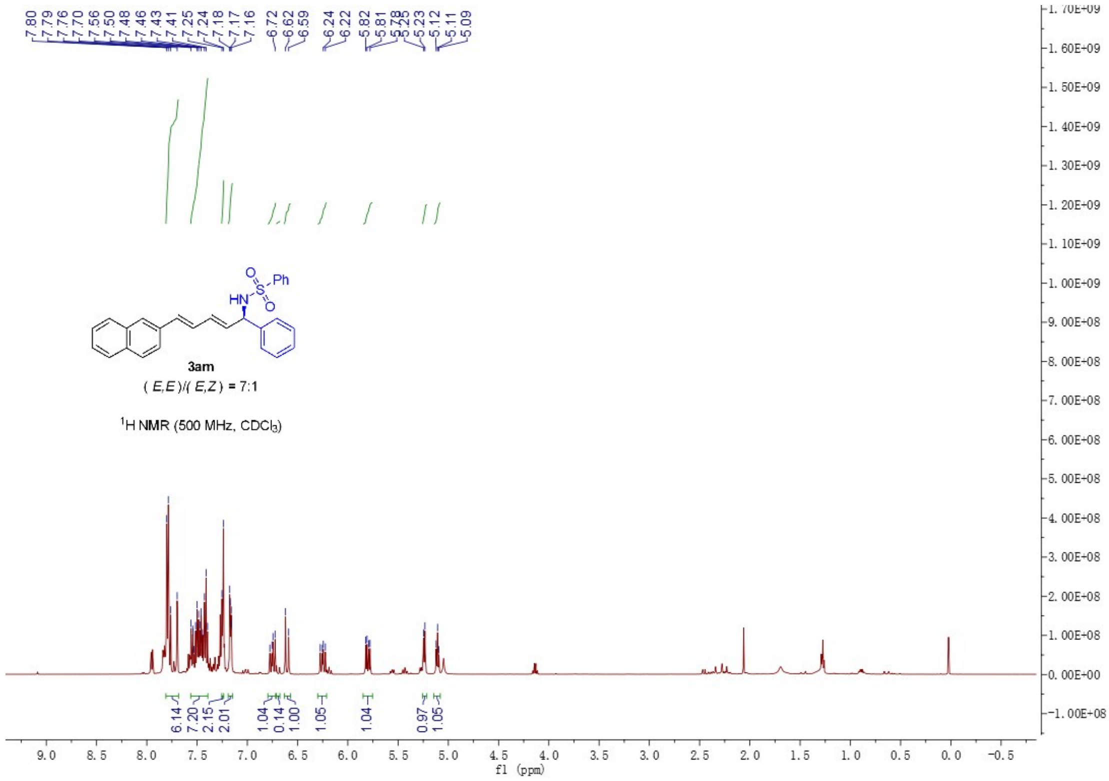
7.80
7.79
7.76
7.70
7.56
7.50
7.48
7.46
7.43
7.41
7.25
7.24
7.18
7.16
6.72
6.62
6.59
6.24
6.22
5.82
5.81
5.78
5.25
5.23
5.12
5.11
5.09
HN
S
O
O
Ph
3am
(E,E)/(E,Z) = 7:1
^{1}H NMR (500 MHz, CDCl$_3$)
6.14
7.20
2.15
2.01
1.04
0.14
1.00
1.05
1.04
0.97
1.05
f1 (ppm)

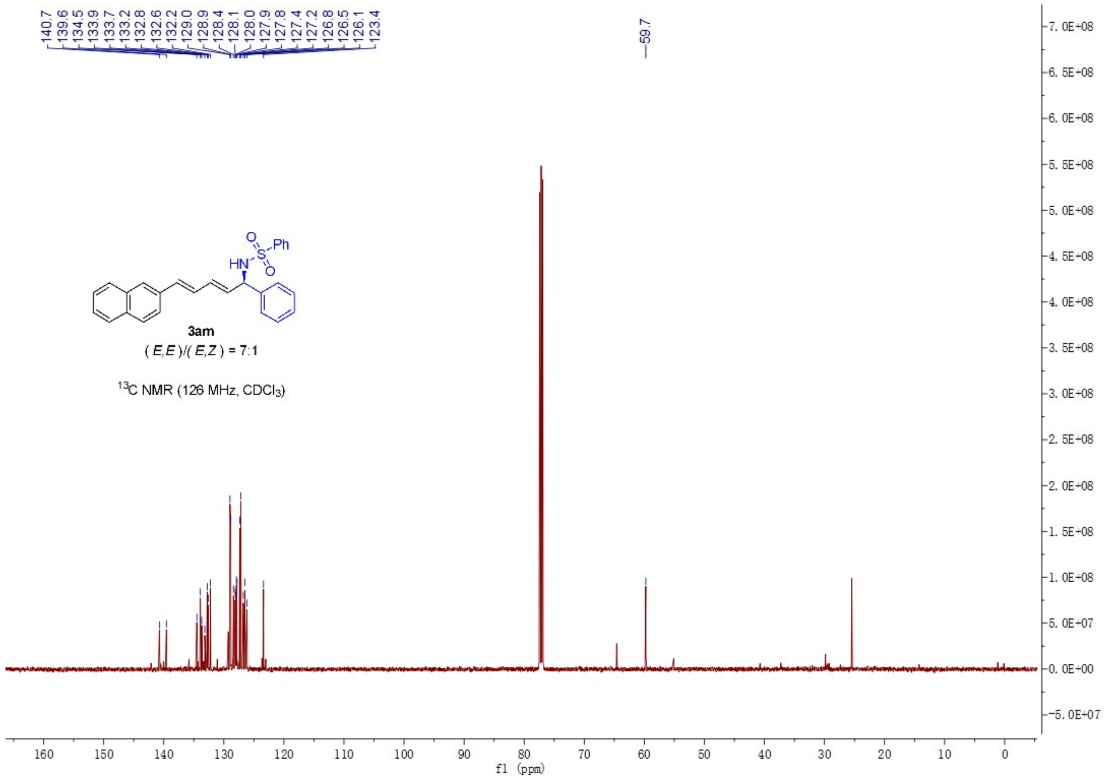

140.7
139.6
134.5
133.9
133.7
133.2
132.8
132.6
132.2
129.0
128.9
128.4
128.1
128.0
127.9
127.8
127.4
127.2
126.8
126.5
126.1
123.4
59.7
HN
S
O
O
Ph
3am
(E,E)/(E,Z) = 7:1
^{13}C NMR (126 MHz, CDCl$_3$)
fl (ppm)

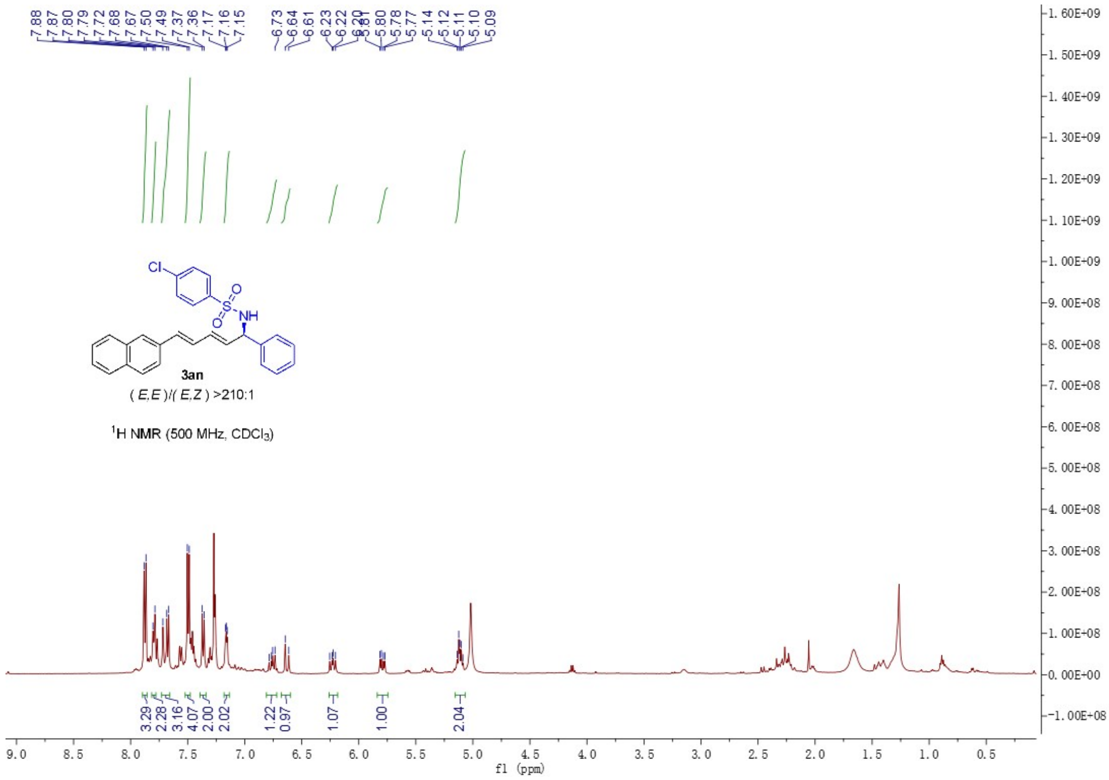

7.88
7.87
7.80
7.79
7.72
7.68
7.67
7.50
7.49
7.37
7.36
7.17
7.16
7.15
6.73
6.64
6.61
6.23
6.22
5.80
5.78
5.77
5.14
5.12
5.11
5.10
5.09
Cl
S
O
O
NH
3an
(E,E)/(E,Z) >210:1
^{1}H NMR (500 MHz, CDCl$_3$)
3.29
2.28
3.16
4.07
2.00
2.02
1.22
0.97
1.07
1.00
2.04
fl (ppm)

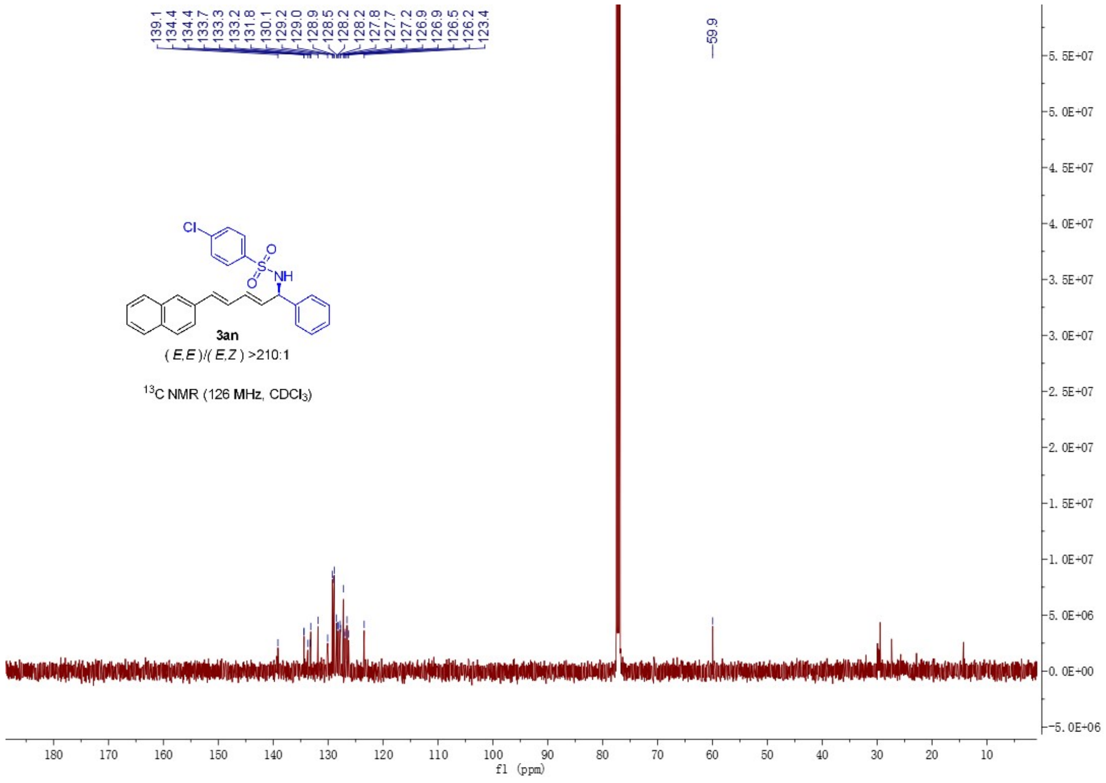

SAMPLE INFORMATION

Sample Name:	LXL-9027-4 IA20VS80	Acquired By:	Breeze
Sample Type:		Date Acquired:	2022/4/5 21:00:29 CST
Vial:	1:A,4	Acq. Method:	iPr vs Hex 20vs80
Injection #:	1	Date Processed:	2022/9/2 9:00:52 CST
Injection Volume:	5.00 ul	Channel Name:	290.0 nm
Run Time:	20.00 Minutes	Channel Desc.:	2998 (210-400) nm
Column Type:		Sample Set Name:	LXL

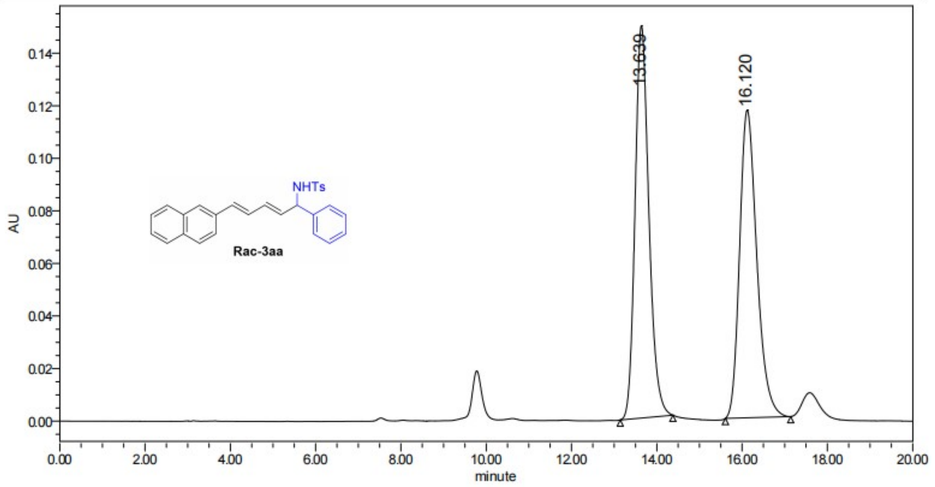

	RT (min)	Area (*sec)	% Area	Height	% Height
1	13.639	3357199	50.61	149302	56.05
2	16.120	3276732	49.39	117062	43.95

SAMPLE INFORMATION

Sample Name:	LXL-9046-5 IA20VS80	Acquired By:	Breeze
Sample Type:		Date Acquired:	2022/5/10 12:16:53 CST
Vial:	1:A,3	Acq. Method:	iPr vs Hex 20vs80
Injection #:	1	Date Processed:	2022/9/2 8:56:50 CST
Injection Volume:	10.00 ul	Channel Name:	290.0 nm
Run Time:	20.00 Minutes	Channel Desc.:	2998 (210-400) nm
Column Type:		Sample Set Name	LXL

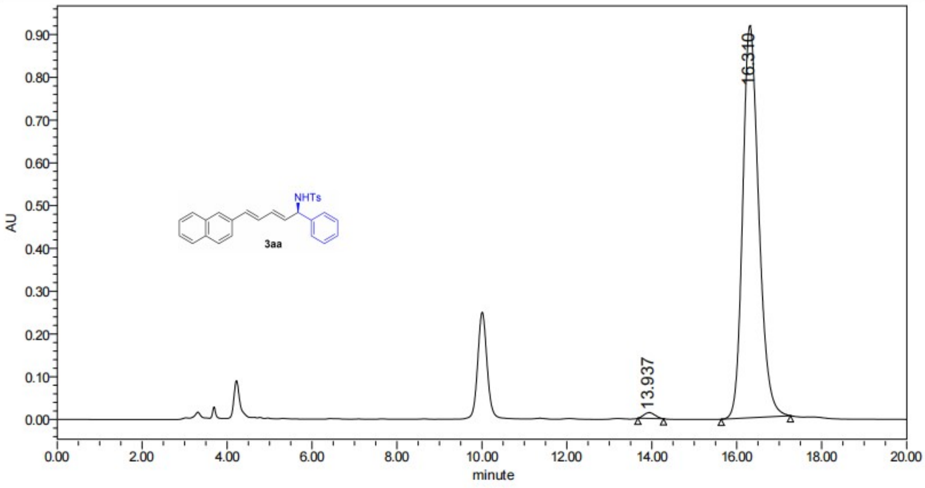

	RT (min)	Area (*sec)	% Area	Height	% Height
1	13.937	248367	1.02	13631	1.46
2	16.310	24177203	98.98	916933	98.54

SAMPLE INFORMATION

Sample Name:	LXL-9098-6 IA5VS95	Acquired By:	Breeze
Sample Type:		Date Acquired:	2022/8/12 12:46:42 CST
Vial:	1:A,1	Acq. Method:	iPr vs Hex 5vs95
Injection #:	1	Date Processed:	2022/8/12 14:18:51 CST
Injection Volume:	5.00 ul	Channel Name:	290.0 nm
Run Time:	46.00 Minutes	Channel Desc.:	2998 (210-400) nm
Column Type:		Sample Set Name	LXL

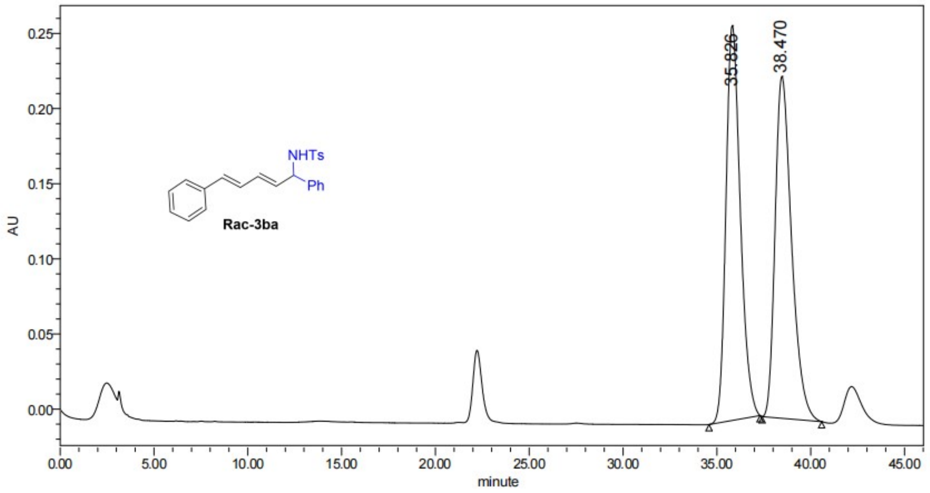

	RT (min)	Area (*sec)	% Area	Height	% Height
1	35.826	13969094	50.13	262698	53.60
2	38.470	13896835	49.87	227395	46.40

SAMPLE INFORMATION

Sample Name:	LXL-9098-4 IA5VS95	Acquired By:	Breeze
Sample Type:		Date Acquired:	2022/8/12 13:33:03 CST
Vial:	1:A,2	Acq. Method:	iPr vs Hex 5vs95
Injection #:	1	Date Processed:	2022/8/12 14:19:52 CST
Injection Volume:	5.00 ul	Channel Name:	290.0 nm
Run Time:	46.00 Minutes	Channel Desc.:	2998 (210-400) nm
Column Type:		Sample Set Name	LXL

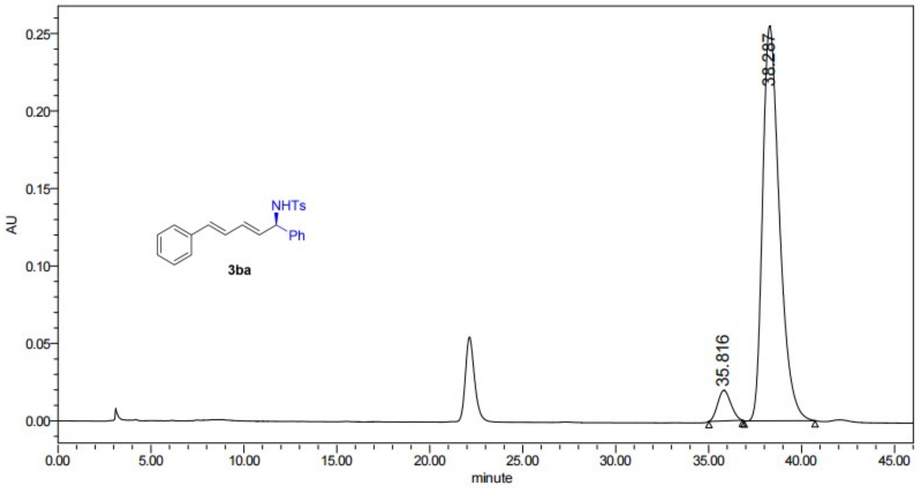

	RT (min)	Area (*sec)	% Area	Height	% Height
1	35.816	970563	5.68	19735	7.19
2	38.287	16126629	94.32	254924	92.81

SAMPLE INFORMATION

Sample Name:	LXL-9029-4 IA20VS80	Acquired By:	Breeze
Sample Type:		Date Acquired:	2022/4/11 12:56:30 CST
Vial:	1:A,2	Acq. Method:	iPr vs Hex 20vs80
Injection #:	1	Date Processed:	2022/6/1 20:44:26 CST
Injection Volume:	5.00 ul	Channel Name:	272.8 nm
Run Time:	20.00 Minutes	Channel Desc.:	2998 (210-400) nm
Column Type:		Sample Set Name	LXL

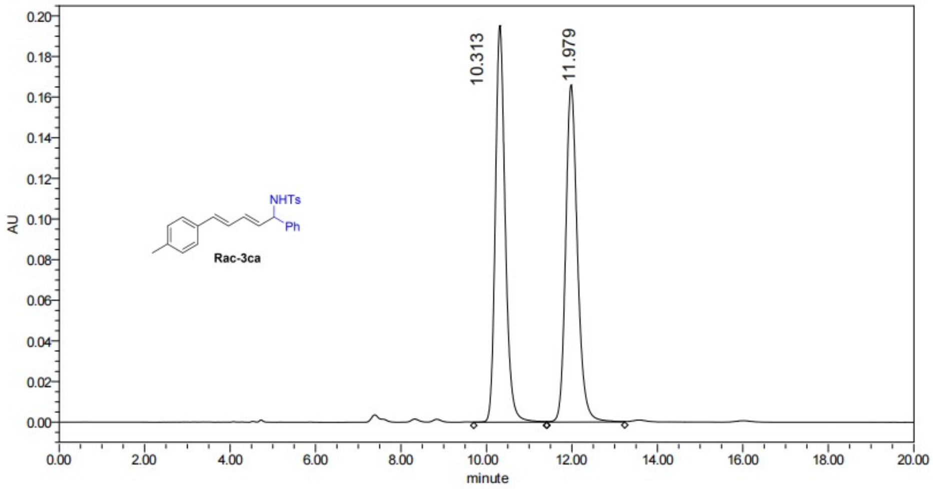

	RT (min)	Area (*sec)	% Area	Height	% Height
1	10.313	3142598	49.74	195231	54.07
2	11.979	3175830	50.26	165873	45.93

SAMPLE INFORMATION

Sample Name:	LXL-9049-5 IA20VS80	Acquired By:	Breeze
Sample Type:		Date Acquired:	2022/5/28 11:08:33 CST
Vial:	1:A,5	Acq. Method:	iPr vs Hex 20vs80
Injection #:	1	Date Processed:	2022/6/1 20:43:11 CST
Injection Volume:	10.00 ul	Channel Name:	272.8 nm
Run Time:	15.00 Minutes	Channel Desc.:	2998 (210-400) nm
Column Type:		Sample Set Name	LXL

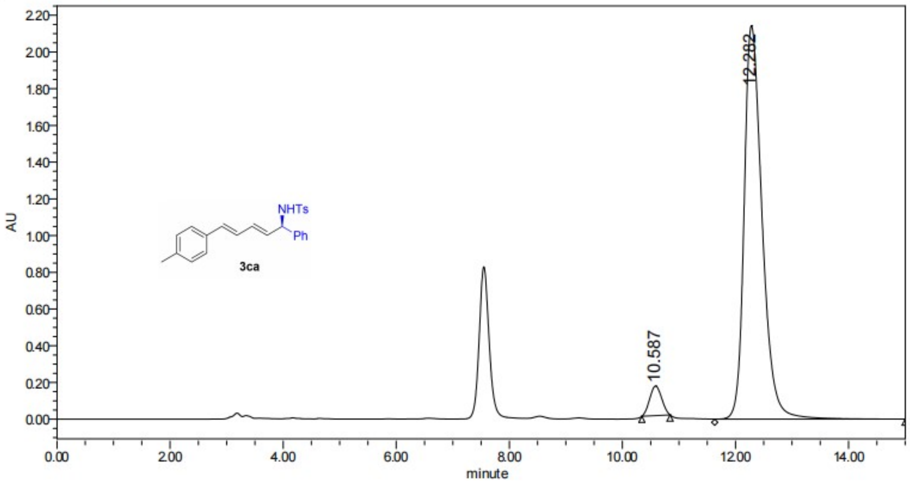

	RT (min)	Area (*sec)	% Area	Height	% Height
1	10.587	2356215	4.74	160930	6.98
2	12.282	47318254	95.26	2143136	93.02

SAMPLE INFORMATION

Sample Name:	lxl-m-Me-1 IA 5vs95	Acquired By:	Breeze
Sample Type:		Date Acquired:	2023/1/3 10:20:42 CST
Vial:	1:A,1	Acq. Method:	iPr vs Hex 5vs95
Injection #:	1	Date Processed:	2023/1/6 20:03:28 CST
Injection Volume:	5.00 ul	Channel Name:	254.0 nm
Run Time:	80.00 Minutes	Channel Desc.:	2998 (210-400) nm
Column Type:		Sample Set Name	1561

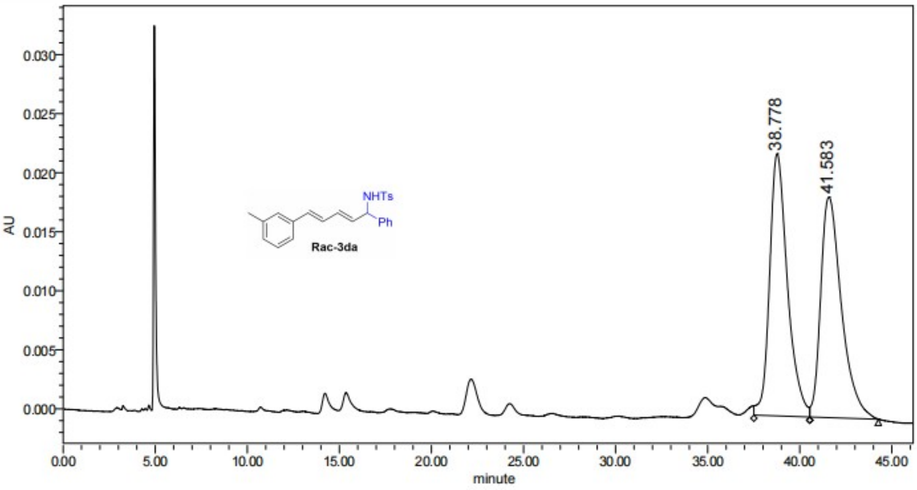

	RT (min)	Area (*sec)	% Area	Height	% Height
1	38.778	1500422	50.57	22220	54.32
2	41.583	1466575	49.43	18686	45.68

SAMPLE INFORMATION

Sample Name:	lxl-m-Me IA 5vs95	Acquired By:	Breeze
Sample Type:		Date Acquired:	2023/1/3 11:11:34 CST
Vial:	1:A,1	Acq. Method:	iPr vs Hex 5vs95
Injection #:	1 Injection	Date Processed:	2023/1/6 20:03:58 CST
Volume: 5.00 ul		Channel Name:	254.0 nm
Run Time:	80.00 Minutes	Channel Desc.:	2998 (210-400) nm
Column Type:		Sample Set Name	154616

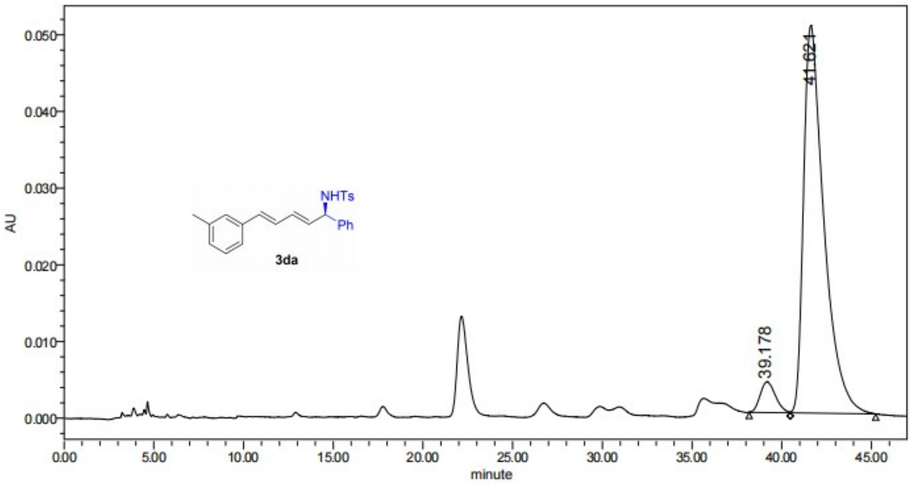

	RT (min)	Area (*sec)	% Area	Height	% Height
1	39.178	237750	5.62	4004	7.34
2	41.621	3993875	94.38	50551	92.66

SAMPLE INFORMATION

Sample Name:	LXL-9029-1 IA20VS80	Acquired By:	Breeze
Sample Type:		Date Acquired:	2022/4/11 12:36:07 CST
Vial:	1:A,1	Acq. Method:	iPr vs Hex 20vs80
Injection #:	1	Date Processed:	2022/6/1 20:35:13 CST
Injection Volume:	5.00 ul	Channel Name:	254.0 nm
Run Time:	20.00 Minutes	Channel Desc.:	2998 (210-400) nm
Column Type:		Sample Set Name:	LXL

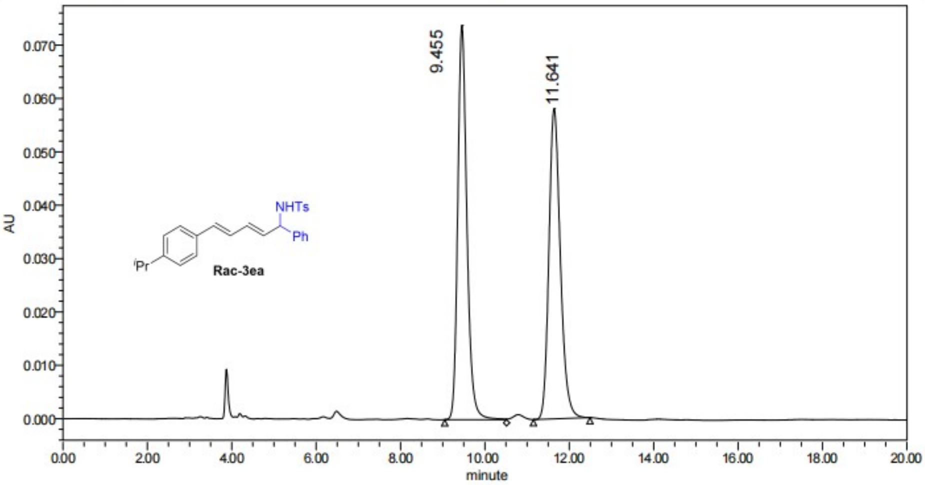

	RT (min)	Area (*sec)	% Area	Height	% Height
1	9.455	1116554	50.48	73858	55.96
2	11.641	1095233	49.52	58137	44.04

SAMPLE INFORMATION

Sample Name:	LXL-9049-1 IA20VS80	Acquired By:	Breeze
Sample Type:		Date Acquired:	2022/5/12 11:05:53 CST
Vial:	1:A,1	Acq. Method:	iPr vs Hex 20vs80
Injection #:	1	Date Processed:	2022/6/1 20:37:57 CST
Injection Volume:	10.00 ul	Channel Name:	254.0 nm
Run Time:	15.00 Minutes	Channel Desc.:	2998 (210-400) nm
Column Type:		Sample Set Name	LXL

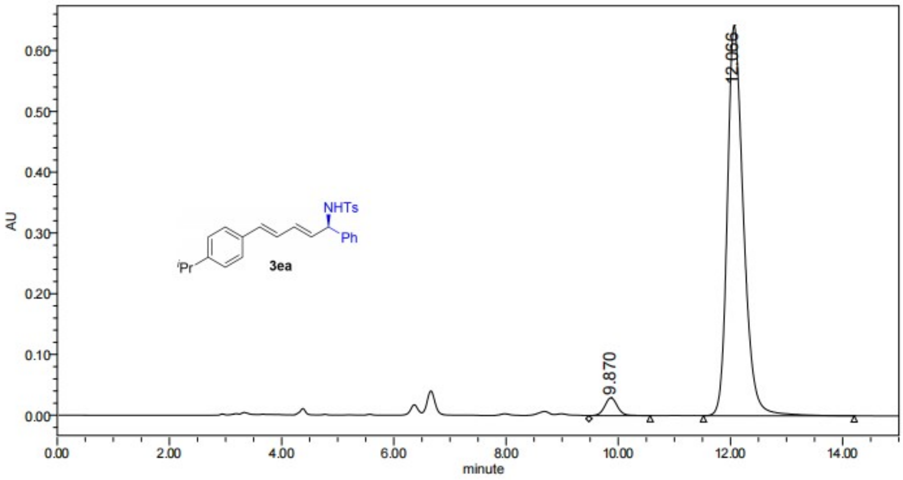

	RT (min)	Area (*sec)	% Area	Height	% Height
1	9.870	458648	3.45	29318	4.36
2	12.066	12841421	96.55	642346	95.64

SAMPLE INFORMATION

Sample Name:	lxl-tBu-2 ia 20vs80	Acquired By:	Breeze
Sample Type:		Date Acquired:	2023/1/2 9:52:23 CST
Vial:	1:A,1	Acq. Method:	iPr vs Hex 20vs80
Injection #:	1 Injection	Date Processed:	2023/1/6 20:01:24 CST
Volume: 5.00 ul		Channel Name:	321.1 nm
Run Time:	30.00 Minutes	Channel Desc.:	2998 (210-400) nm
Column Type:		Sample Set Name	1161

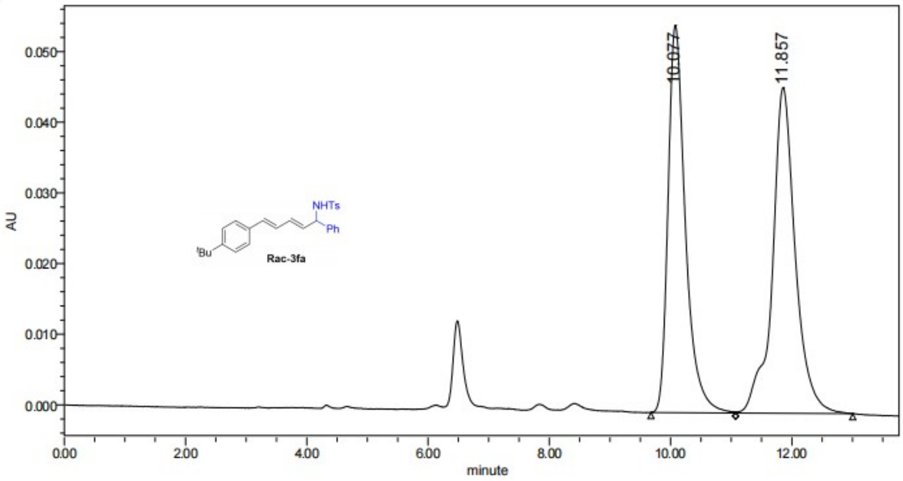

	RT (min)	Area (*sec)	% Area	Height	% Height
1	10.077	1089117	47.31	54832	54.32
2	11.857	1213209	52.69	46107	45.68

SAMPLE INFORMATION

Sample Name:	lxl-tBu IA 20VS80	Acquired By:	Breeze
Sample Type:		Date Acquired:	2022/12/29 19:59:02 CST
Vial:	1:A,1 Injection	Acq. Method:	iPr vs Hex 20vs80
#:	1 Injection Volume:	Date Processed:	2022/12/29 20:37:31 CST
5.00 ul		Channel Name:	321.1 nm
Run Time:	30.00 Minutes	Channel Desc.:	2998 (210-400) nm
Column Type:		Sample Set Name	5413

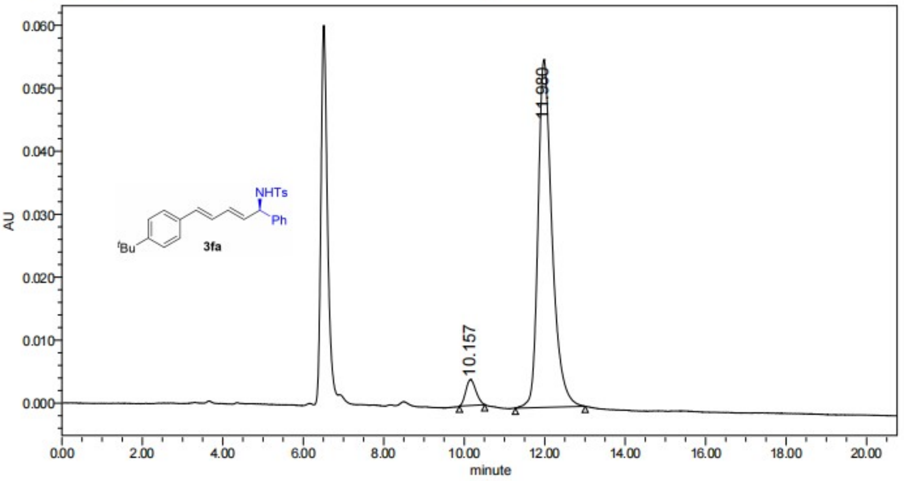

	RT (min)	Area (*sec)	% Area	Height	% Height
1	10.157	71462	5.07	4132	6.96
2	11.980	1338381	94.93	55231	93.04

SAMPLE INFORMATION

Sample Name:	LXL-9029-5 IA20VS80	Acquired By:	Breeze
Sample Type:		Date Acquired:	2022/5/21 0:59:10 CST
Vial:	1:A,5	Acq. Method:	iPr vs Hex 20vs80
Injection #:	1	Date Processed:	2022/6/1 20:53:42 CST
Injection Volume:	5.00 ul	Channel Name:	278.5 nm
Run Time:	20.00 Minutes	Channel Desc.:	2998 (210-400) nm
Column Type:		Sample Set Name:	LXL

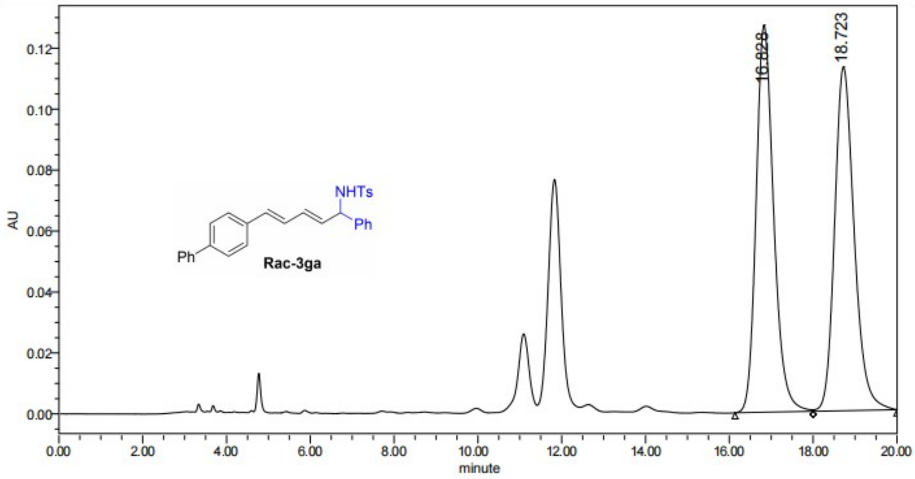

	RT (min)	Area (*sec)	% Area	Height	% Height
1	16.828	3694343	50.28	127023	52.96
2	18.723	3653783	49.72	112812	47.04

SAMPLE INFORMATION

Sample Name:	LXL-9049-2 IA20VS80	Acquired By:	Breeze
Sample Type:		Date Acquired:	2022/5/12 11:21:17 CST
Vial:	1:A,2	Acq. Method:	iPr vs Hex 20vs80
Injection #:	1	Date Processed:	2022/7/14 14:48:56 CST
Injection Volume:	10.00 ul	Channel Name:	278.5 nm
Run Time:	20.00 Minutes	Channel Desc.:	2998 (210-400) nm
Column Type:		Sample Set Name	LXL

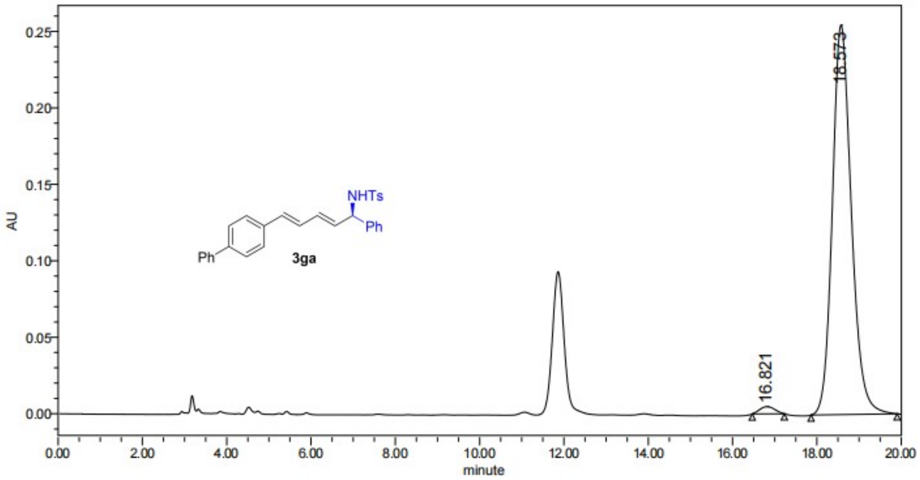

	RT (min)	Area (*sec)	% Area	Height	% Height
1	16.821	118218	1.46	4760	1.83
2	18.573	7965567	98.54	254746	98.17

SAMPLE INFORMATION

Sample Name:	LXL-9029-18 IA20VS80	Acquired By:	Breeze
Sample Type:		Date Acquired:	2022/5/11 11:59:27 CST
Vial:	1:A,5	Acq. Method:	iPr vs Hex 20vs80
Injection #:	1	Date Processed:	2022/8/3 14:51:36 CST
Injection Volume:	10.00 ul	Channel Name:	306.5 nm @2
Run Time:	20.00 Minutes	Channel Desc.:	2998 (210-400) nm
Column Type:		Sample Set Name	LXL

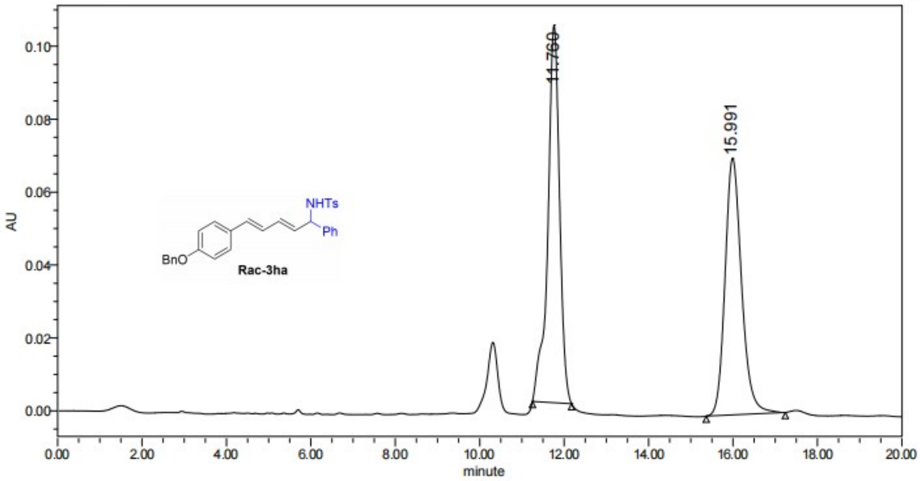

	RT (min)	Area (*sec)	% Area	Height	% Height
1	11.760	2004492	51.20	103549	59.52
2	15.991	1910844	48.80	70418	40.48

SAMPLE INFORMATION

Sample Name:	LXL-9049-12 IA20VS80	Acquired By:	Breeze
Sample Type:		Date Acquired:	2022/5/14 12:13:18 CST
Vial:	1:A,5	Acq. Method:	iPr vs Hex 20vs80
Injection #:	1	Date Processed:	2022/8/3 14:50:22 CST
Injection Volume:	10.00 ul	Channel Name:	306.5 nm
Run Time:	19.00 Minutes	Channel Desc.:	2998 (210-400) nm
Column Type:		Sample Set Name	LXL

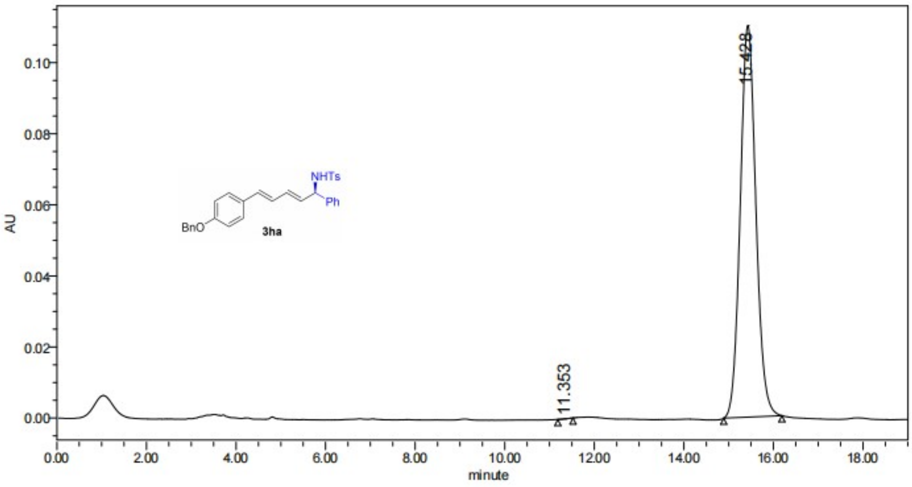

	RT (min)	Area (*sec)	% Area	Height	% Height
1	11.353	226	0.01	-33	0.03
2	15.428	2668806	99.99	110164	99.97

SAMPLE INFORMATION

Sample Name:	LXL-9029-13 IA20VS80	Acquired By:	Breeze
Sample Type:		Date Acquired:	2022/6/24 7:54:00 CST
Vial:	1:A,1	Acq. Method:	iPr vs Hex 20vs80
Injection #:	1	Date Processed:	2022/6/24 9:21:55 CST
Injection Volume:	5.00 ul	Channel Name:	307.7 nm
Run Time:	24.00 Minutes	Channel Desc.:	2998 (210-400) nm
Column Type:		Sample Set Name	LXL

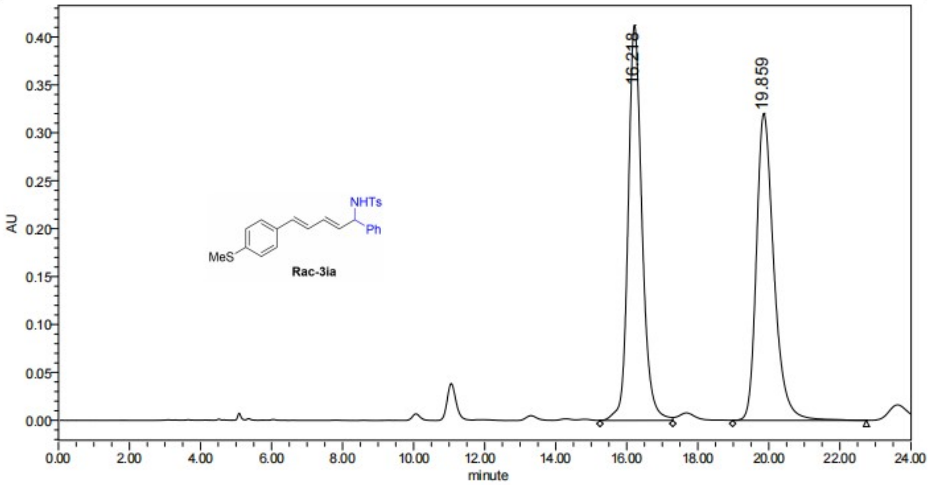

	RT (min)	Area (*sec)	% Area	Height	% Height
1	16.218	11185347	50.49	412354	56.28
2	19.859	10967010	49.51	320338	43.72

SAMPLE INFORMATION

Sample Name:	LXL-9069-3 IA20VS80	Acquired By:	Breeze
Sample Type:		Date Acquired:	2022/6/24 8:18:22 CST
Vial:	1:A,2	Acq. Method:	iPr vs Hex 20vs80
Injection #:	1	Date Processed:	2022/7/14 15:08:56 CST
Injection Volume:	5.00 ul	Channel Name:	307.7 nm
Run Time:	24.00 Minutes	Channel Desc.:	2998 (210-400) nm
Column Type:		Sample Set Name	LXL

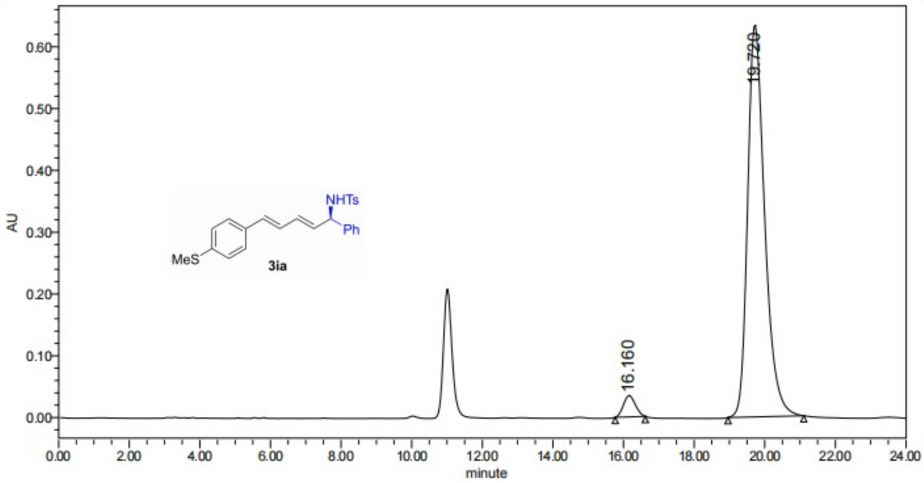

	RT (min)	Area (*sec)	% Area	Height	% Height
1	16.160	808803	3.67	34430	5.16
2	19.720	21249453	96.33	633396	94.84

SAMPLE INFORMATION

Sample Name:	LXL-9029-21 IA20VS80	Acquired By:	Breeze
Sample Type:		Date Acquired:	2022/6/15 17:21:42 CST
Vial:	1:A,2	Acq. Method:	iPr vs Hex 20vs80
Injection #:	1	Date Processed:	2022/8/3 14:56:12 CST
Injection Volume:	5.00 ul	Channel Name:	306.5 nm @3
Run Time:	33.00 Minutes	Channel Desc.:	2998 (210-400) nm
Column Type:		Sample Set Name	LXL

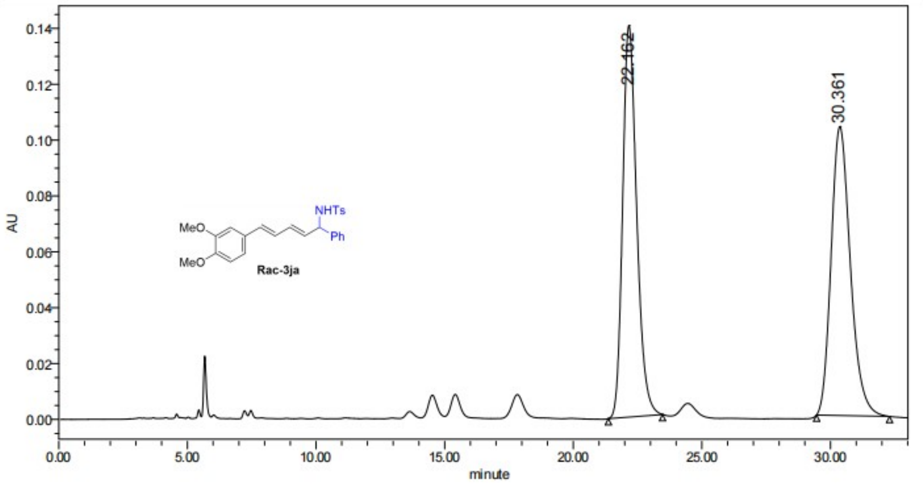

	RT (min)	Area (*sec)	% Area	Height	% Height
1	22.162	5303634	49.65	140199	57.54
2	30.361	5378280	50.35	103436	42.46

SAMPLE INFORMATION

Sample Name:	LXL-9049-16 IA20VS80	Acquired By:	Breeze
Sample Type:		Date Acquired:	2022/6/14 15:52:14 CST
Vial:	1:A,1	Acq. Method:	iPr vs Hex 20vs80
Injection #:	1	Date Processed:	2022/8/3 14:55:12 CST
Injection Volume:	5.00 ul	Channel Name:	306.5 nm
Run Time:	33.00 Minutes	Channel Desc.:	2998 (210-400) nm
Column Type:		Sample Set Name:	LXL

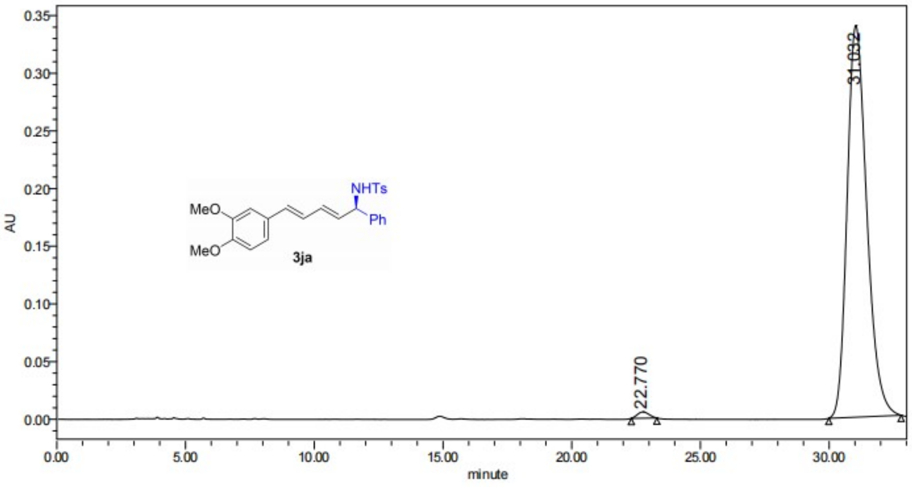

	RT (min)	Area (*sec)	% Area	Height	% Height
1	22.770	170527	0.94	5482	1.59
2	31.032	18011485	99.06	339494	98.41

SAMPLE INFORMATION

Sample Name:	LXL-9029-14 IA10VS90	Acquired By:	Breeze
Sample Type:		Date Acquired:	2022/6/24 16:07:21 CST
Vial:	1:A,2	Acq. Method:	iPr vs Hex 10vs90
Injection #:	1	Date Processed:	2022/8/3 14:58:11 CST
Injection Volume:	5.00 ul	Channel Name:	283.0 nm
Run Time:	30.00 Minutes	Channel Desc.:	2998 (210-400) nm
Column Type:		Sample Set Name	LXL

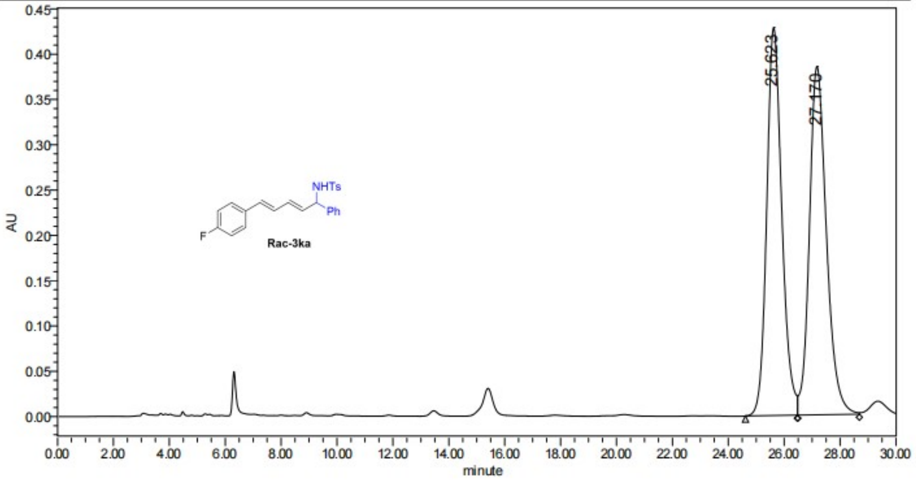

	RT (min)	Area (*sec)	% Area	Height	% Height
1	25.623	16037980	49.63	428239	52.69
2	27.170	16275747	50.37	384495	47.31

SAMPLE INFORMATION

Sample Name:	LXL-9078-8 IA10VS90	Acquired By:	Breeze
Sample Type:		Date Acquired:	2022/6/24 15:25:29 CST
Vial:	1:A,2	Acq. Method:	iPr vs Hex 10vs90
Injection #:	1	Date Processed:	2022/8/3 14:59:48 CST
Injection Volume:	5.00 ul	Channel Name:	283.0 nm
Run Time:	30.00 Minutes	Channel Desc.:	2998 (210-400) nm
Column Type:		Sample Set Name	LXL

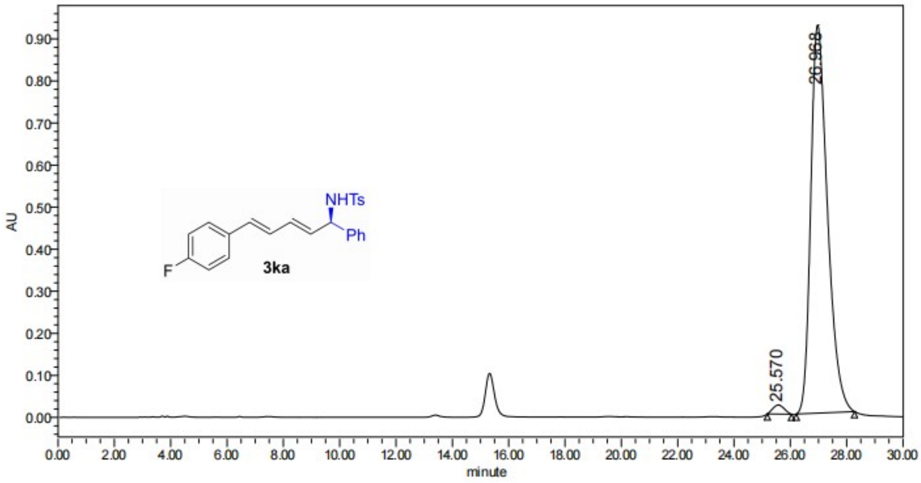

	RT (min)	Area (*sec)	% Area	Height	% Height
1	25.570	604105	1.56	21491	2.28
2	26.968	38038116	98.44	922593	97.72

SAMPLE INFORMATION

Sample Name:	LXL-9029-16 IA20VS80	Acquired By:	Breeze
Sample Type:		Date Acquired:	2022/5/21 3:41:20 CST
Vial:	1:B,3	Acq. Method:	iPr vs Hex 20vs80
Injection #:	1	Date Processed:	2022/7/14 15:19:19 CST
Injection Volume:	5.00 ul	Channel Name:	278.0 nm @1
Run Time:	22.00 Minutes	Channel Desc.:	2998 (210-400) nm
Column Type:		Sample Set Name	LHF

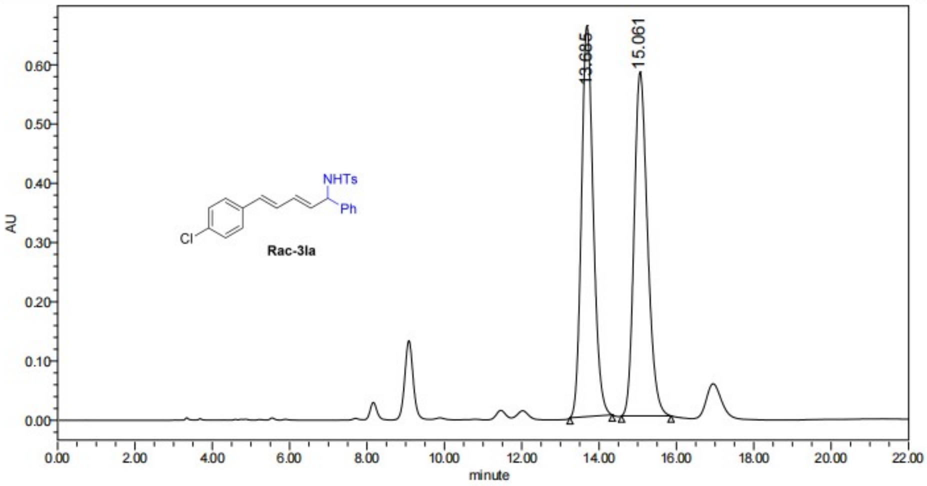

	RT (min)	Area (*sec)	% Area	Height	% Height
1	13.685	13967196	49.95	659755	53.21
2	15.061	13994455	50.05	580229	46.79

SAMPLE INFORMATION

Sample Name:	LXL-9049-11 IA20VS80	Acquired By:	Breeze
Sample Type:		Date Acquired:	2022/5/21 4:03:43 CST
Vial:	1:B,4	Acq. Method:	iPr vs Hex 20vs80
Injection #:	1	Date Processed:	2022/7/14 15:18:19 CST
Injection Volume:	5.00 ul	Channel Name:	278.0 nm
Run Time:	22.00 Minutes	Channel Desc.:	2998 (210-400) nm
Column Type:		Sample Set Name:	LXL

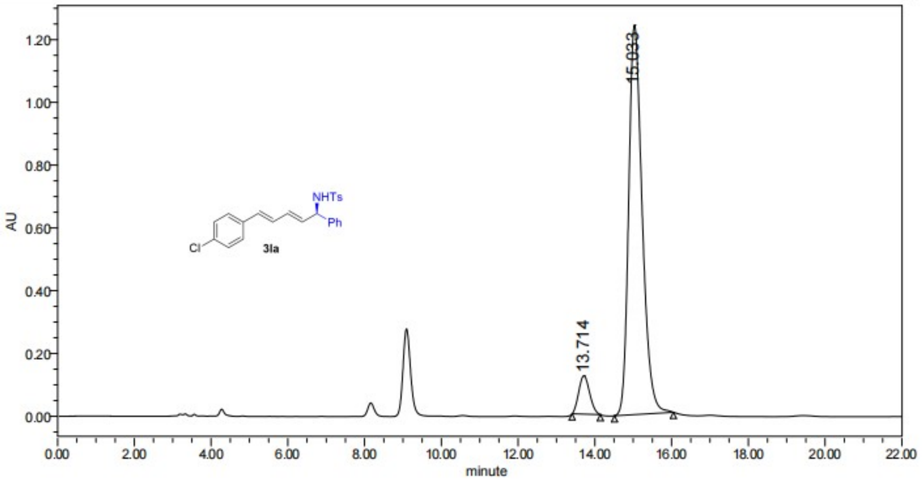

	RT (min)	Area (*sec)	% Area	Height	% Height
1	13.714	2411242	7.38	122407	8.98
2	15.033	30257629	92.62	1240785	91.02

SAMPLE INFORMATION

Sample Name:	LXL-9029-12 IA20VS80	Acquired By:	Breeze
Sample Type:		Date Acquired:	2022/5/11 10:05:40 CST
Vial:	1:A,3	Acq. Method:	iPr vs Hex 20vs80
Injection #:	1	Date Processed:	2022/6/14 15:28:16 CST
Injection Volume:	10.00 ul	Channel Name:	295.5 nm
Run Time:	20.00 Minutes	Channel Desc.:	2998 (210-400) nm
Column Type:		Sample Set Name	LXL

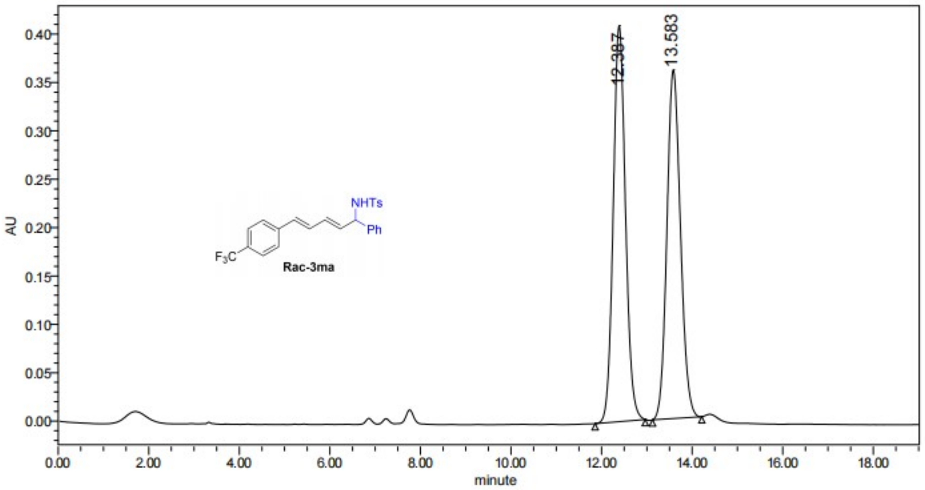

	RT (min)	Area (*sec)	% Area	Height	% Height
1	12.387	7665293	50.65	409262	53.18
2	13.583	7467153	49.35	360292	46.82

SAMPLE INFORMATION

Sample Name:	LXL-9069-2 IA20VS80	Acquired By:	Breeze
Sample Type:		Date Acquired:	2022/6/14 14:58:44 CST
Vial:	1:A,2	Acq. Method:	iPr vs Hex 20vs80
Injection #:	1	Date Processed:	2022/6/14 15:27:12 CST
Injection Volume:	5.00 ul	Channel Name:	295.5 nm
Run Time:	16.00 Minutes	Channel Desc.:	2998 (210-400) nm
Column Type:		Sample Set Name	LXL

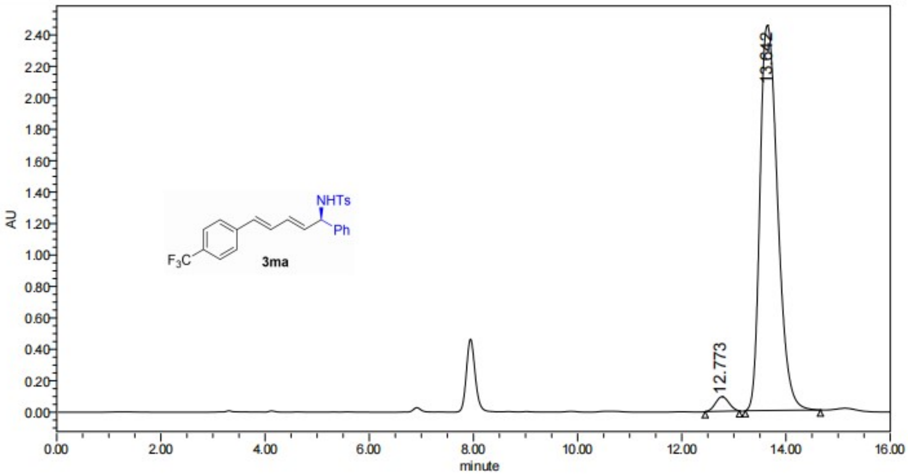

	RT (min)	Area (*sec)	% Area	Height	% Height
1	12.773	1653622	2.78	92892	3.65
2	13.642	57864060	97.22	2450399	96.35

SAMPLE INFORMATION

Sample Name:	LXL-9029-11 IA20VS80	Acquired By:	Breeze
Sample Type:		Date Acquired:	2022/5/21 1:39:52 CST
Vial:	1:A,7	Acq. Method:	iPr vs Hex 20vs80
Injection #:	1	Date Processed:	2022/6/1 21:18:45 CST
Injection Volume:	5.00 ul	Channel Name:	329.1 nm
Run Time:	30.00 Minutes	Channel Desc.:	2998 (210-400) nm
Column Type:		Sample Set Name:	LXL

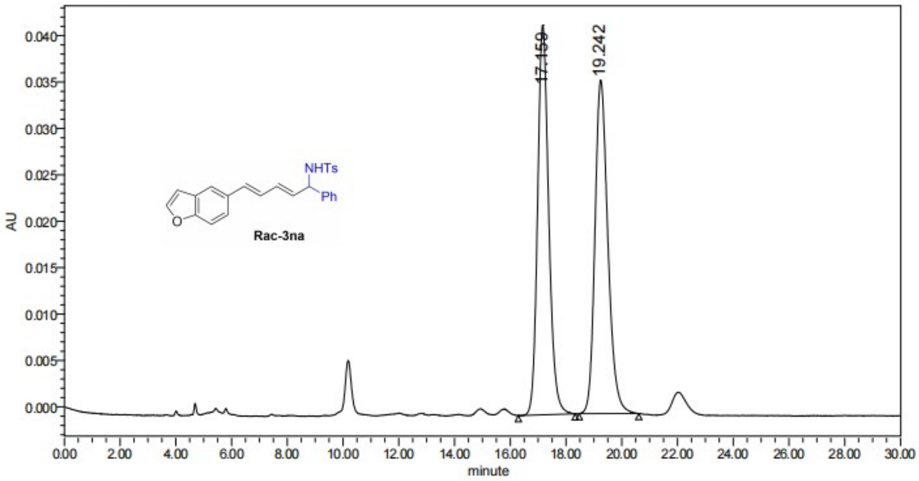

	RT (min)	Area (*sec)	% Area	Height	% Height
1	17.159	1160004	49.99	41973	53.87
2	19.242	1160647	50.01	35948	46.13

SAMPLE INFORMATION

Sample Name:	LXL-9049-7 IA20VS80	Acquired By:	Breeze
Sample Type:		Date Acquired:	2022/5/21 2:10:14 CST
Vial:	1:A,8	Acq. Method:	iPr vs Hex 20vs80
Injection #:	1	Date Processed:	2022/6/1 21:20:22 CST
Injection Volume:	5.00 ul	Channel Name:	329.1 nm @1
Run Time:	30.00 Minutes	Channel Desc.:	2998 (210-400) nm
Column Type:		Sample Set Name:	LXL

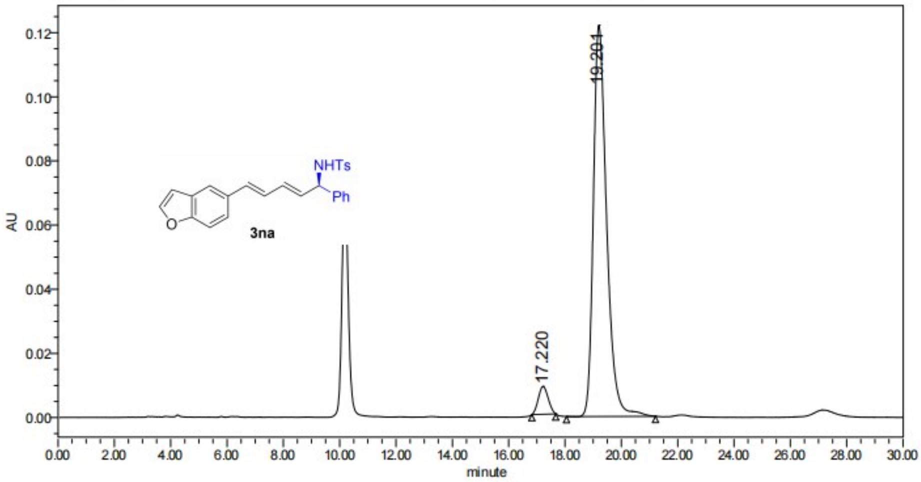

	RT (min)	Area (*sec)	% Area	Height	% Height
1	17.220	210660	5.04	8694	6.65
2	19.201	3972487	94.96	121968	93.35

SAMPLE INFORMATION

Sample Name:	LXL-9029-15 IA20VS80	Acquired By:	Breeze
Sample Type:		Date Acquired:	2022/5/11 11:18:42 CST
Vial:	1:A,3	Acq. Method:	iPr vs Hex 20vs80
Injection #:	1	Date Processed:	2022/6/1 21:15:52 CST
Injection Volume:	10.00 ul	Channel Name:	254.0 nm @11
Run Time:	20.00 Minutes	Channel Desc.:	2998 (210-400) nm
Column Type:		Sample Set Name	LXL

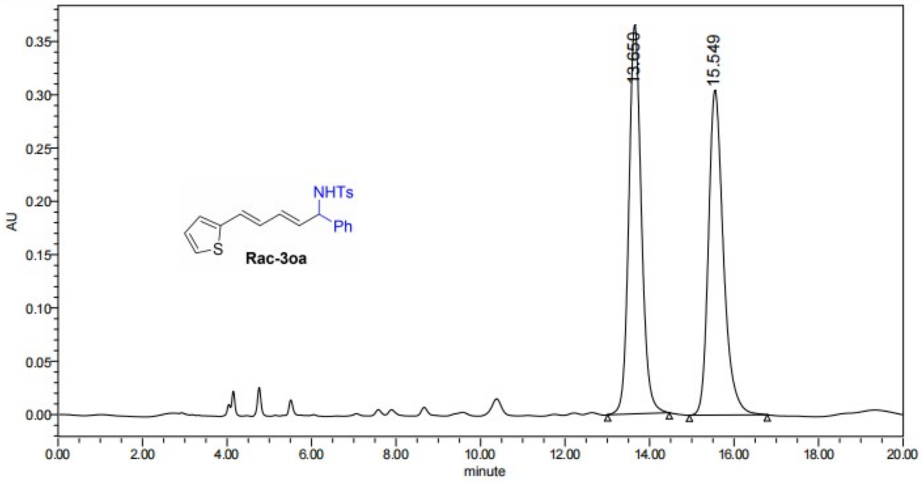

	RT (min)	Area (*sec)	% Area	Height	% Height
1	13.650	7512689	49.99	364625	54.47
2	15.549	7515281	50.01	304741	45.53

SAMPLE INFORMATION

Sample Name:	LXL-9049-10 IA20VS80	Acquired By:	Breeze
Sample Type:		Date Acquired:	2022/5/14 11:36:31 CST
Vial:	1:A,3	Acq. Method:	iPr vs Hex 20vs80
Injection #:	1	Date Processed:	2022/6/1 21:14:22 CST
Injection Volume:	10.00 ul	Channel Name:	254.0 nm @10
Run Time:	18.00 Minutes	Channel Desc.:	2998 (210-400) nm
Column Type:		Sample Set Name	LXL

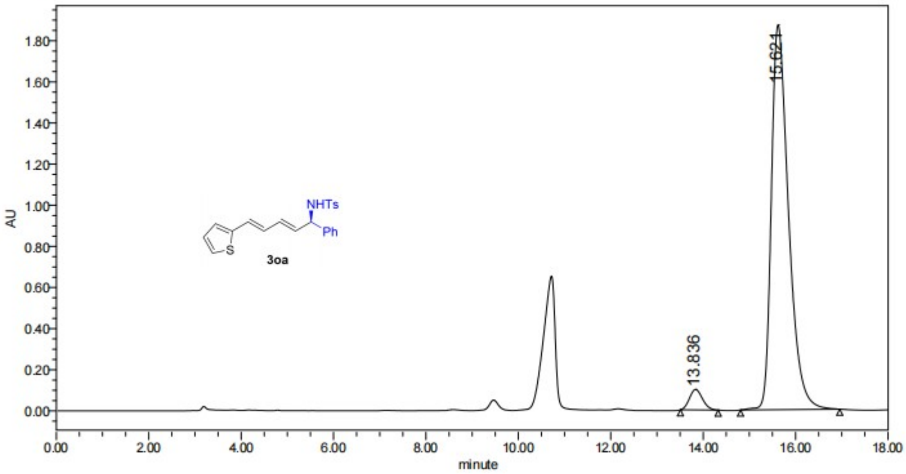

	RT (min)	Area (*sec)	% Area	Height	% Height
1	13.836	1954016	3.89	99777	5.06
2	15.621	48298923	96.11	1871000	94.94

SAMPLE INFORMATION

Sample Name:	LXL-9029-8 IA20VS80	Acquired By:	Breeze
Sample Type:		Date Acquired:	2022/5/11 9:24:54 CST
Vial:	1:A,1	Acq. Method:	iPr vs Hex 20vs80
Injection #:	1	Date Processed:	2022/7/14 15:01:48 CST
Injection Volume:	10.00 ul	Channel Name:	290.0 nm
Run Time:	20.00 Minutes	Channel Desc.:	2998 (210-400) nm
Column Type:		Sample Set Name	LXL

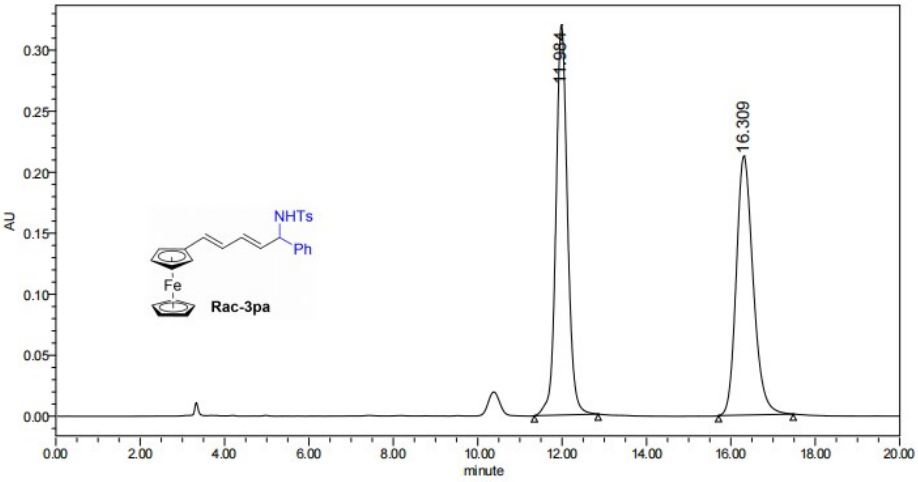

	RT (min)	Area (*sec)	% Area	Height	% Height
1	11.984	6095214	50.77	319700	60.10
2	16.309	5910155	49.23	212253	39.90

SAMPLE INFORMATION

Sample Name:	LXL-9049-6 IA20VS80	Acquired By:	Breeze
Sample Type:		Date Acquired:	2022/5/14 9:31:03 CST
Vial:	1:A,2	Acq. Method:	iPr vs Hex 20vs80
Injection #:	1	Date Processed:	2022/7/14 15:00:01 CST
Injection Volume:	10.00 ul	Channel Name:	290.0 nm @1
Run Time:	22.00 Minutes	Channel Desc.:	2998 (210-400) nm
Column Type:		Sample Set Name	LXL

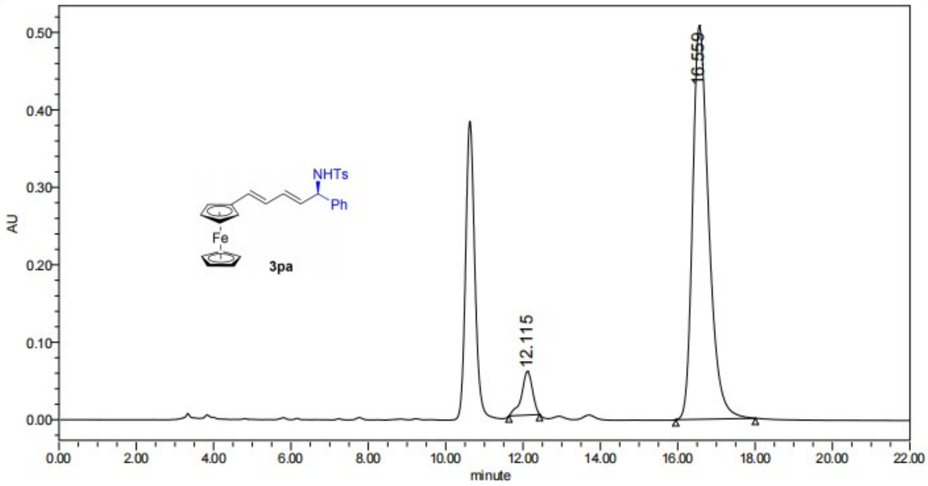

	RT (min)	Area (*sec)	% Area	Height	% Height
1	12.115	1102528	6.97	56404	9.99
2	16.559	14714592	93.03	508394	90.01

SAMPLE INFORMATION

Sample Name:	lxl-NP-1 IA 20VS80	Acquired By:	Breeze
Sample Type:		Date Acquired:	2023/1/6 19:58:33 CST
Vial:	1:A,1	Acq. Method:	iPr vs Hex 20vs80
Injection #:	1	Date Processed:	2023/1/7 14:01:17 CST
Injection Volume:	5.00 ul	Channel Name:	321.1 nm
Run Time:	120.00 Minutes	Channel Desc.:	2998 (210-400) nm
Column Type:		Sample Set Name	asdf

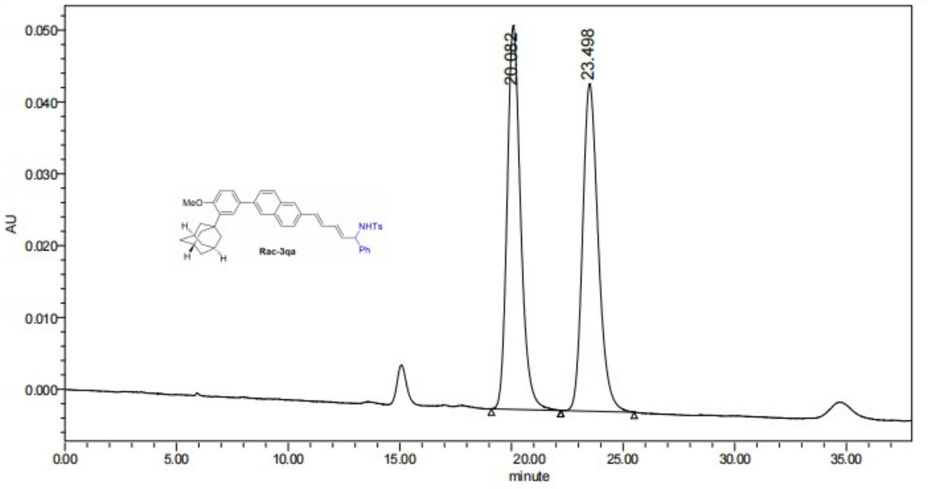

	RT (min)	Area (*sec)	% Area	Height	% Height
1	20.082	2243924	50.20	53421	53.97
2	23.498	2225666	49.80	45557	46.03

SAMPLE INFORMATION

Sample Name:	lxl-NP-2 IA 20VS80	Acquired By:	Breeze
Sample Type:		Date Acquired:	2023/1/7 12:00:13 CST
Vial:	1:A,1	Acq. Method:	iPr vs Hex 20vs80
Injection #:	1 Injection	Date Processed:	2023/1/7 14:02:04 CST
Volume:	5.00 ul	Channel Name:	321.1 nm
Run Time:	30.00 Minutes	Channel Desc.:	2998 (210-400) nm
Column Type:		Sample Set Name	1561

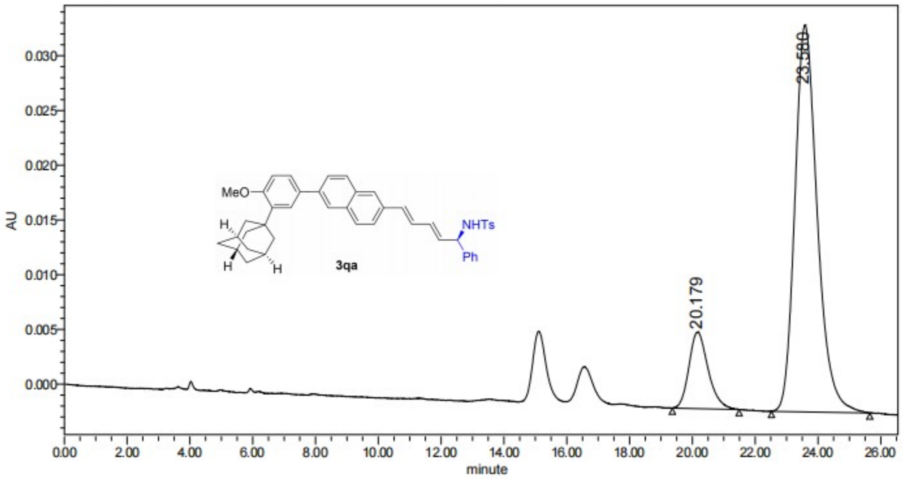

	RT (min)	Area (*sec)	% Area	Height	% Height
1	20.179	297937	14.58	7006	16.54
2	23.580	1746186	85.42	35342	83.46

SAMPLE INFORMATION

Sample Name:	LXL-9077-7-3 IA20VS80	Acquired By:	Breeze
Sample Type:		Date Acquired:	2022/6/27 9:25:59 CST
Vial:	1:A,3	Acq. Method:	iPr vs Hex 20vs80
Injection #:	1	Date Processed:	2022/7/2 10:24:38 CST
Injection Volume:	5.00 ul	Channel Name:	306.3 nm
Run Time:	16.00 Minutes	Channel Desc.:	2998 (210-400) nm
Column Type:		Sample Set Name	LXL

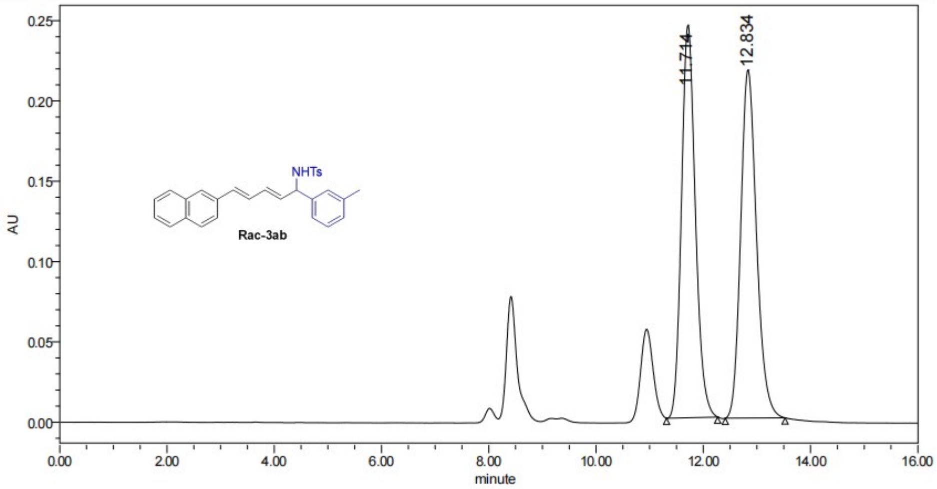

	RT (min)	Area (*sec)	% Area	Height	% Height
1	11.714	4405221	49.75	244350	53.00
2	12.834	4449658	50.25	216646	47.00

SAMPLE INFORMATION

Sample Name:	LXL-9078-7 IA20VS80	Acquired By:	Breeze
Sample Type:		Date Acquired:	2022/6/24 14:33:01 CST
Vial:	1:A,3	Acq. Method:	iPr vs Hex 20vs80
Injection #:	1	Date Processed:	2022/7/2 10:26:05 CST
Injection Volume:	5.00 ul	Channel Name:	306.3 nm
Run Time:	16.00 Minutes	Channel Desc.:	2998 (210-400) nm
Column Type:		Sample Set Name	LXL

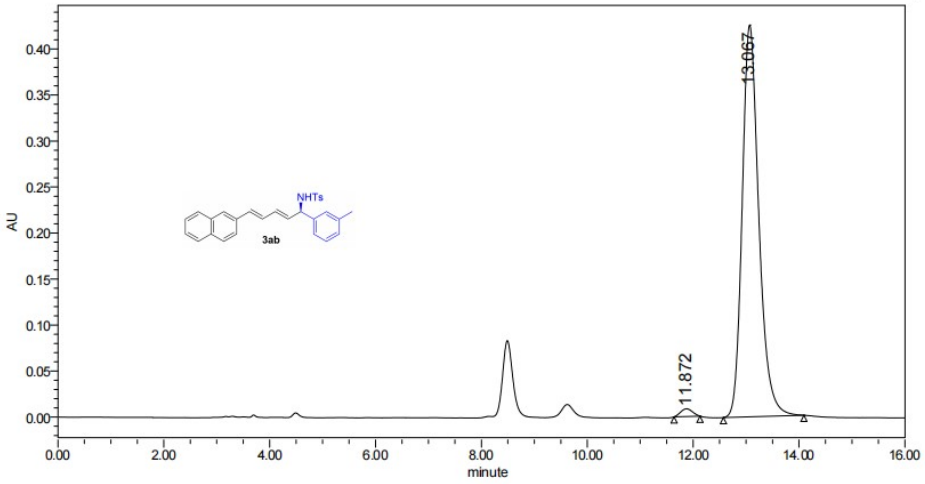

	RT (min)	Area (*sec)	% Area	Height	% Height
1	11.872	129337	1.37	8368	1.93
2	13.067	9283445	98.63	425729	98.07

SAMPLE INFORMATION

Sample Name:	lxl-p-Me-1 ia 20vs80	Acquired By:	Breeze
Sample Type:		Date Acquired:	2023/1/2 10:43:32 CST
Vial:	1:A,2	Acq. Method:	iPr vs Hex 20vs80
Injection #:	1	Date Processed:	2023/1/6 20:02:35 CST
Injection Volume:	5.00 ul	Channel Name:	321.1 nm
Run Time:	30.00 Minutes	Channel Desc.:	2998 (210-400) nm
Column Type:		Sample Set Name	15461061

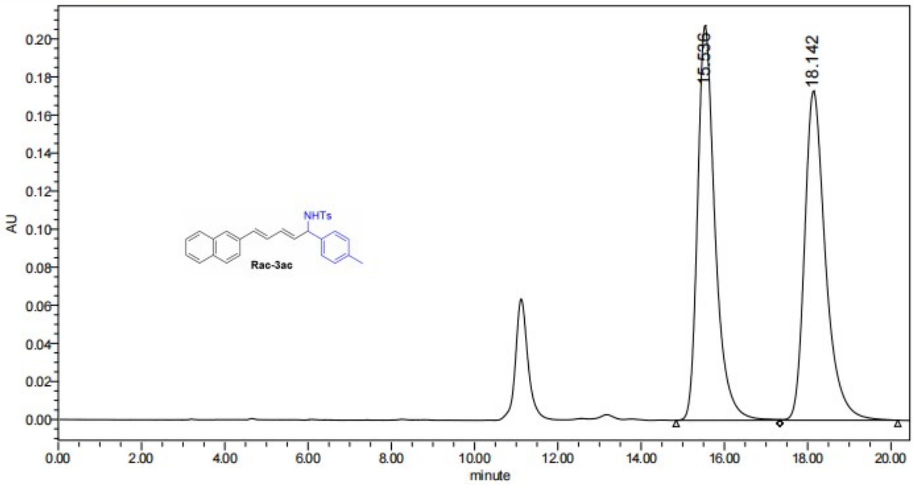

	RT (min)	Area (*sec)	% Area	Height	% Height
1	15.536	6053014	50.04	207399	54.52
2	18.142	6044189	49.96	173019	45.48

SAMPLE INFORMATION

Sample Name:	Ixl-p-Me IA 20VS 80	Acquired By:	Breeze
Sample Type:		Date Acquired:	2022/12/29 21:28:23 CST
Vial:	1:A,3	Acq. Method:	iPr vs Hex 20vs80
Injection #:	1 Injection	Date Processed:	2023/1/6 20:00:51 CST
Volume: 5.00 ul		Channel Name:	321.1 nm
Run Time:	30.00 Minutes	Channel Desc.:	2998 (210-400) nm
Column Type:		Sample Set Name	46416

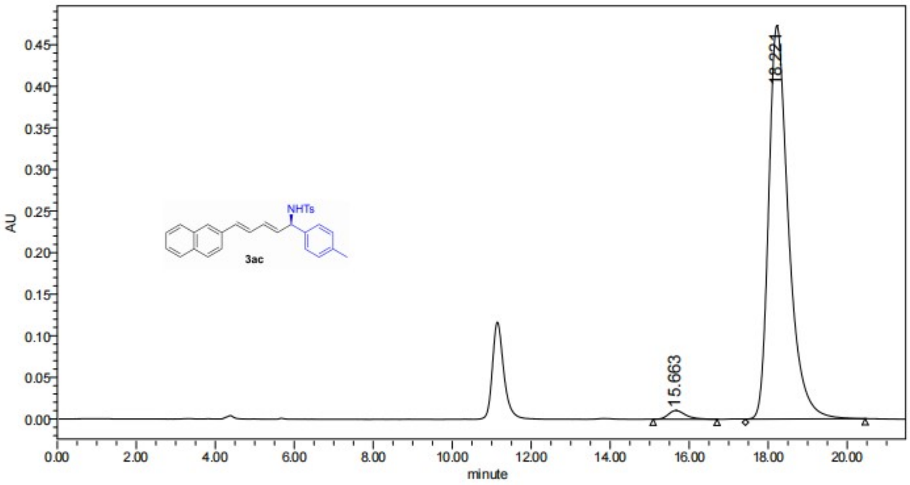

	RT (min)	Area (*sec)	% Area	Height	% Height
1	15.663	319102	1.89	10511	2.17
2	18.221	16548254	98.11	473182	97.83

SAMPLE INFORMATION

Sample Name:	LXL-90136-3 IA20VS80	Acquired By:	Breeze
Sample Type:		Date Acquired:	2022/10/14 14:19:15 CST
Vial:	1:A,3	Acq. Method:	iPr vs Hex 20vs80
Injection #:	1	Date Processed:	2022/10/14 19:27:30 CST
Injection Volume:	5.00 ul	Channel Name:	321.5 nm
Run Time:	20.00 Minutes	Channel Desc.:	2998 (210-400) nm
Column Type:		Sample Set Name	LXL

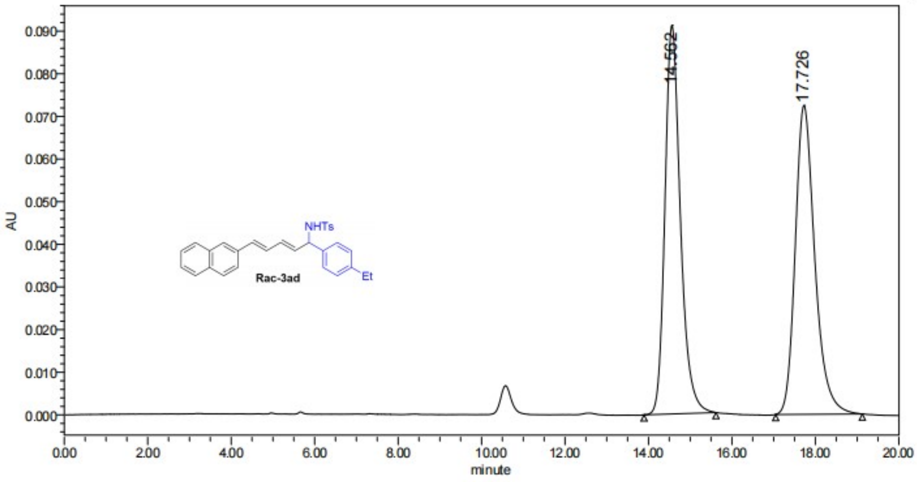

	RT (min)	Area (*sec)	% Area	Height	% Height
1	14.562	2343335	49.82	91155	55.71
2	17.726	2359886	50.18	72465	44.29

SAMPLE INFORMATION

Sample Name:	LXL-90136-11 IA20VS80	Acquired By:	Breeze
Sample Type:		Date Acquired:	2022/10/14 18:07:34 CST
Vial:	1:A,4	Acq. Method:	iPr vs Hex 20vs80
Injection #:	1	Date Processed:	2022/10/14 19:26:45 CST
Injection Volume:	5.00 ul	Channel Name:	321.5 nm
Run Time:	20.00 Minutes	Channel Desc.:	2998 (210-400) nm
Column Type:		Sample Set Name	LXL

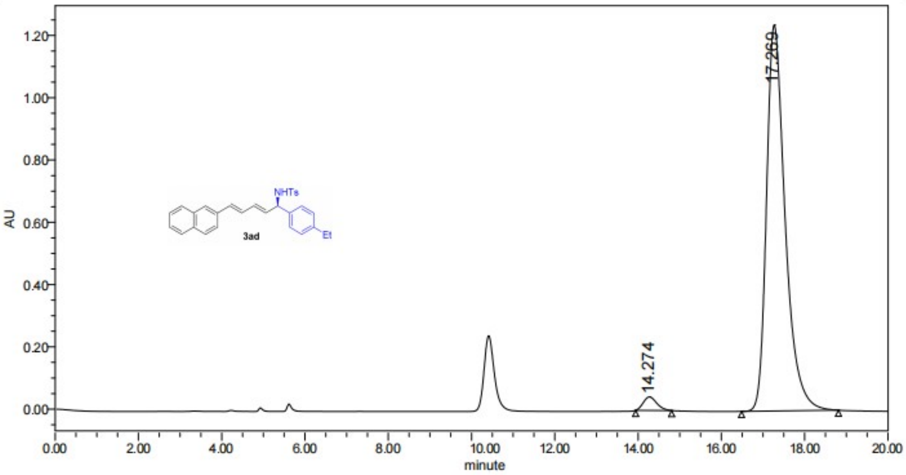

	RT (min)	Area (*sec)	% Area	Height	% Height
1	14.274	969611	2.46	43259	3.37
2	17.269	38489084	97.54	1239593	96.63

SAMPLE INFORMATION

Sample Name:	LXL-9032-3 IA20VS80	Acquired By:	Breeze
Sample Type:		Date Acquired:	2022/5/20 23:53:04 CST
Vial:	1:A,2	Acq. Method:	iPr vs Hex 20vs80
Injection #:	1	Date Processed:	2022/7/12 15:50:49 CST
Injection Volume:	5.00 ul	Channel Name:	254.0 nm @18
Run Time:	20.00 Minutes	Channel Desc.:	2998 (210-400) nm
Column Type:		Sample Set Name:	LXL

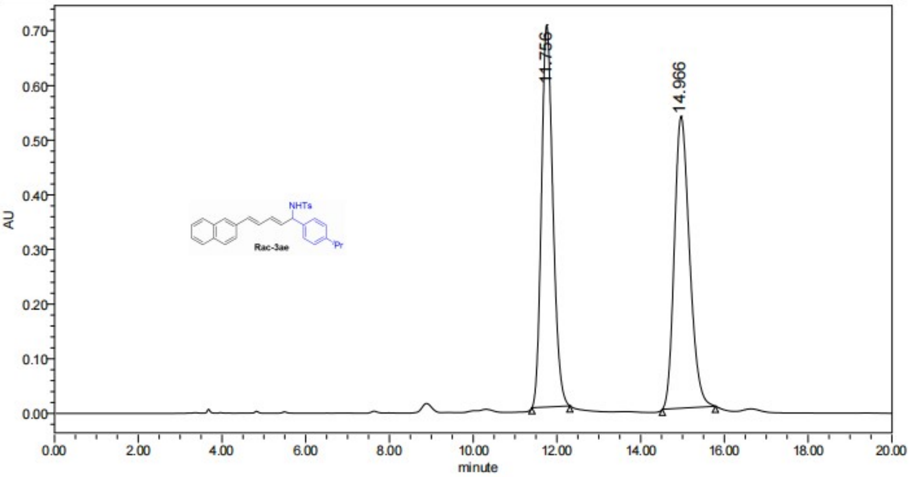

	RT (min)	Area (*sec)	% Area	Height	% Height
1	11.756	13296816	49.68	699168	56.68
2	14.966	13467379	50.32	534317	43.32

SAMPLE INFORMATION

Sample Name:	LXL-9066-3-2 IA20VS80	Acquired By:	Breeze
Sample Type:		Date Acquired:	2022/6/13 14:29:15 CST
Vial:	1:A,2	Acq. Method:	iPr vs Hex 20vs80
Injection #:	1	Date Processed:	2022/7/12 15:49:24 CST
Injection Volume:	5.00 ul	Channel Name:	254.0 nm
Run Time:	17.00 Minutes	Channel Desc.:	2998 (210-400) nm
Column Type:		Sample Set Name	LXL

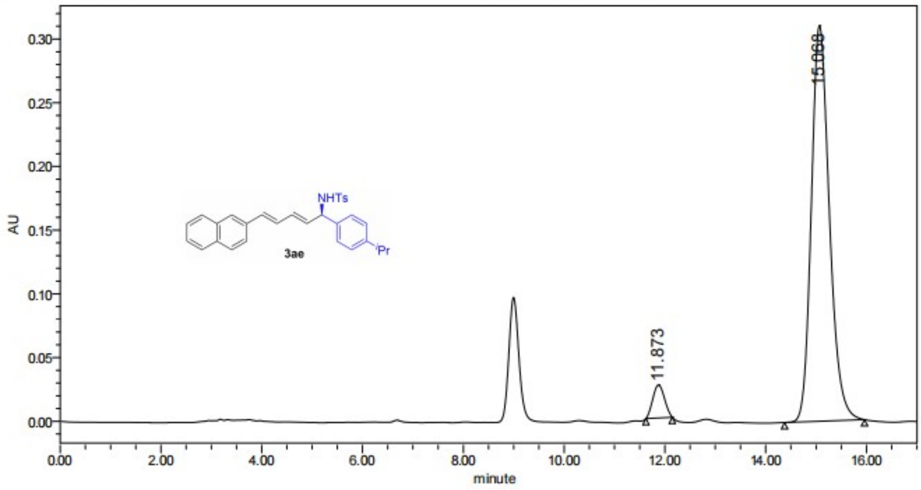

	RT (min)	Area (*sec)	% Area	Height	% Height
1	11.873	413903	5.09	25838	7.68
2	15.068	7712636	94.91	310450	92.32

SAMPLE INFORMATION

Sample Name:	LXL-9077-7-4 IA20VS80	Acquired By:	Breeze
Sample Type:		Date Acquired:	2022/6/27 9:05:38 CST
Vial:	1:A,2	Acq. Method:	iPr vs Hex 20vs80
Injection #:	1	Date Processed:	2022/7/12 16:44:33 CST
Injection Volume:	5.00 ul	Channel Name:	265.0 nm
Run Time:	20.00 Minutes	Channel Desc.:	2998 (210-400) nm
Column Type:		Sample Set Name	LXL

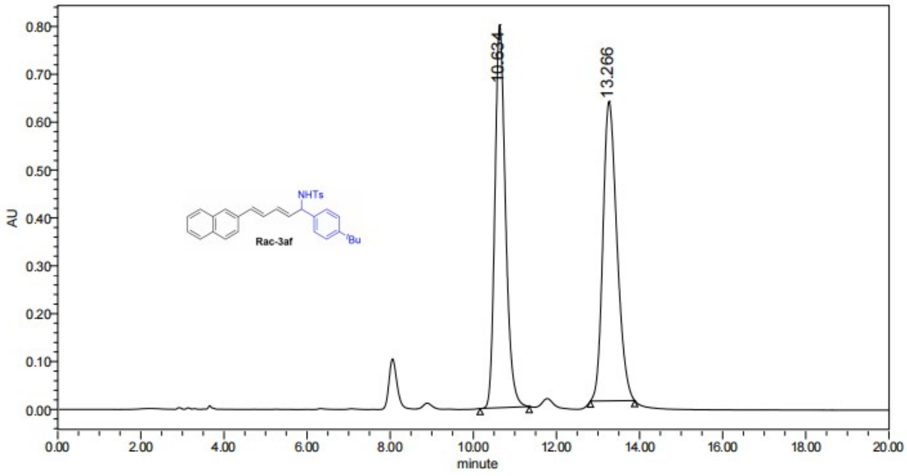

	RT (min)	Area (*sec)	% Area	Height	% Height
1	10.634	14178876	49.17	799760	56.12
2	13.266	14655677	50.83	625428	43.88

SAMPLE INFORMATION

Sample Name:	LXL-9078-6 IA20VS80	Acquired By:	Breeze
Sample Type:		Date Acquired:	2022/6/24 10:50:10 CST
Vial:	1:A,3	Acq. Method:	iPr vs Hex 20vs80
Injection #:	1	Date Processed:	2022/7/2 10:32:52 CST
Injection Volume:	5.00 ul	Channel Name:	265.0 nm
Run Time:	24.00 Minutes	Channel Desc.:	2998 (210-400) nm
Column Type:		Sample Set Name	LXL

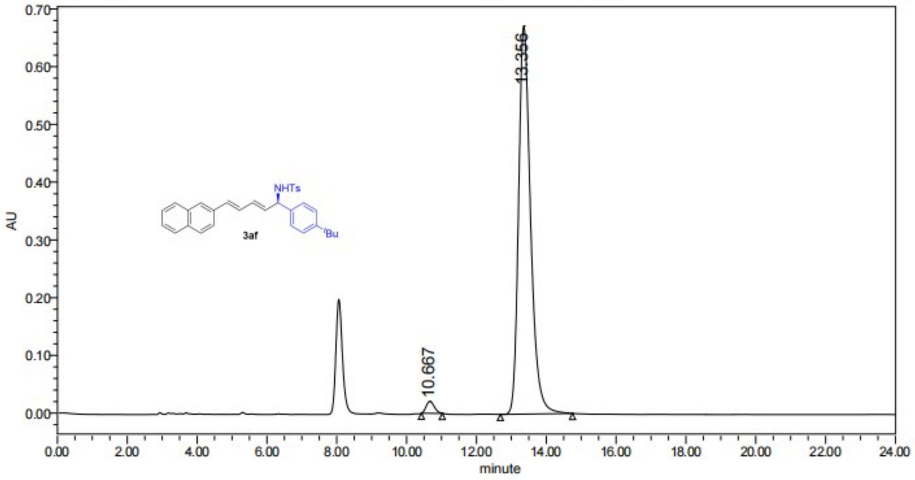

	RT (min)	Area (*sec)	% Area	Height	% Height
1	10.667	334404	2.04	20682	2.99
2	13.356	16023879	97.96	672091	97.01

SAMPLE INFORMATION

Sample Name:	LXL-9032-5 IA20VS80	Acquired By:	Breeze
Sample Type:		Date Acquired:	2022/5/30 13:10:44 CST
Vial:	1:A,7	Acq. Method:	iPr vs Hex 20vs80
Injection #:	1	Date Processed:	2022/6/24 10:47:52 CST
Injection Volume:	10.00 ul	Channel Name:	273.6 nm
Run Time:	28.00 Minutes	Channel Desc.:	2998 (210-400) nm
Column Type:		Sample Set Name	LXL

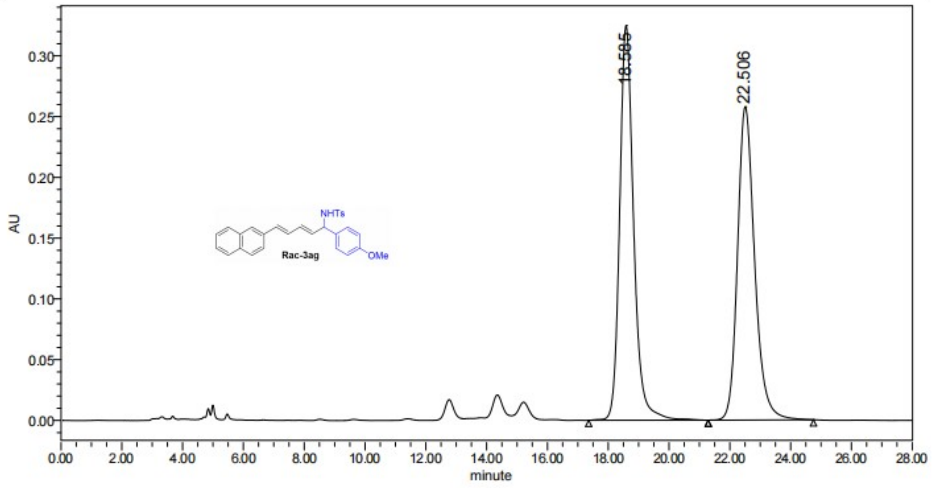

	RT (min)	Area (*sec)	% Area	Height	% Height
1	18.585	10357291	50.67	324811	55.75
2	22.506	10084821	49.33	257788	44.25

SAMPLE INFORMATION

Sample Name:	LXL-9078-4 IA20VS80	Acquired By:	Breeze
Sample Type:		Date Acquired:	2022/6/24 10:02:25 CST
Vial:	1:A,1	Acq. Method:	iPr vs Hex 20vs80
Injection #:	1	Date Processed:	2022/6/24 10:45:18 CST
Injection Volume:	5.00 ul	Channel Name:	273.6 nm
Run Time:	25.00 Minutes	Channel Desc.:	2998 (210-400) nm
Column Type:		Sample Set Name	LXL

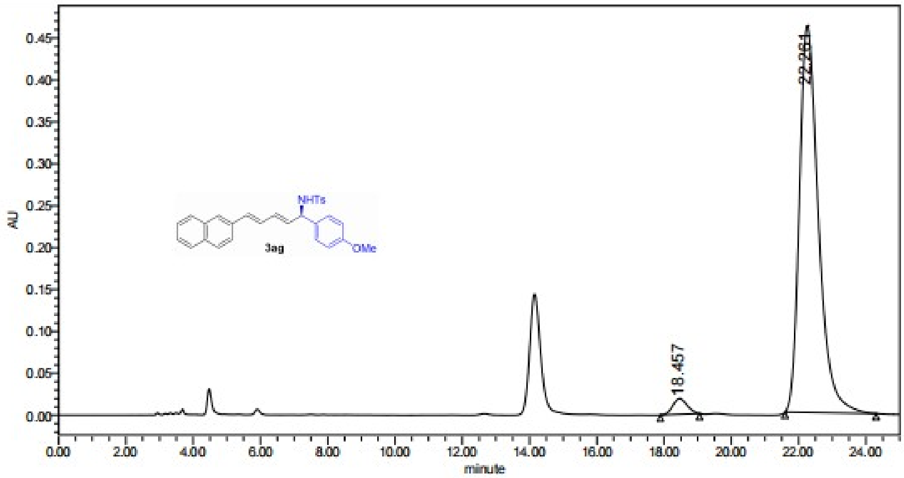

	RT (min)	Area (*sec)	% Area	Height	% Height
1	18.457	561451	3.01	18774	3.91
2	22.261	18068265	96.99	461921	96.09

SAMPLE INFORMATION

Sample Name:	LXL-9032-6 IA20VS80	Acquired By:	Breeze
Sample Type:		Date Acquired:	2022/6/23 20:22:28 CST
Vial:	1:A,1	Acq. Method:	iPr vs Hex 20vs80
Injection #:	1	Date Processed:	2022/7/2 10:42:20 CST
Injection Volume:	5.00 ul	Channel Name:	305.0 nm@1
Run Time:	20.00 Minutes	Channel Desc.:	2998 (210-400) nm
Column Type:		Sample Set Name	LXL

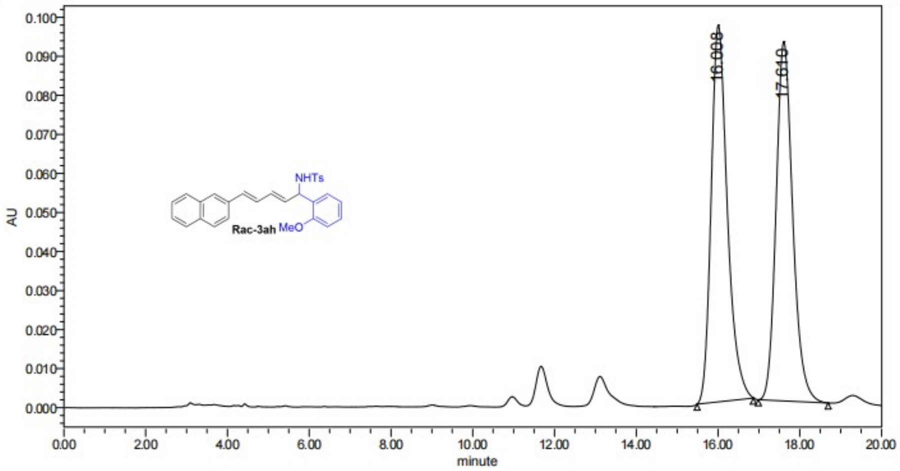

	RT (min)	Area (*sec)	% Area	Height	% Height
1	16.008	2650798	50.46	96485	51.18
2	17.610	2602848	49.54	92043	48.82

SAMPLE INFORMATION

Sample Name:	LXL-9078-5 IA20VS80	Acquired By:	Breeze
Sample Type:		Date Acquired:	2022/6/24 10:27:47 CST
Vial:	1:A,2	Acq. Method:	iPr vs Hex 20vs80
Injection #:	1	Date Processed:	2022/7/2 10:40:09 CST
Injection Volume:	5.00 ul	Channel Name:	305.0 nm
Run Time:	22.00 Minutes	Channel Desc.:	2998 (210-400) nm
Column Type:		Sample Set Name	LXL

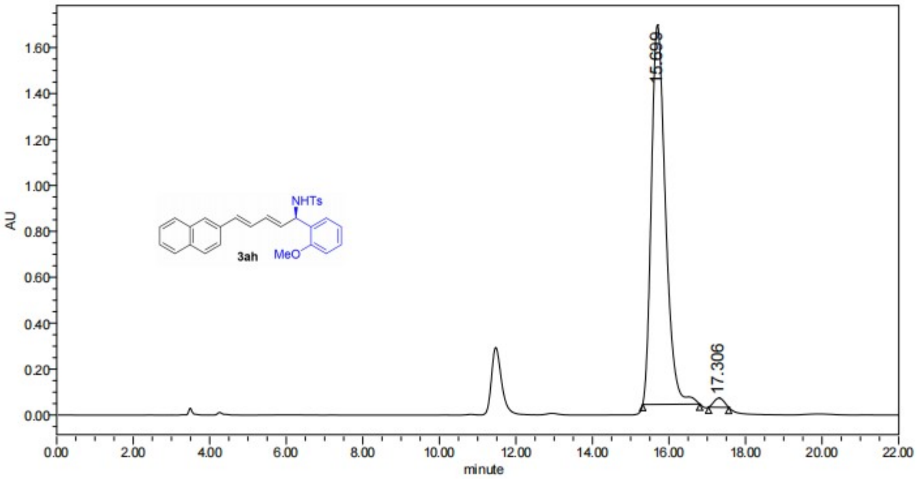

	RT (min)	Area (*sec)	% Area	Height	% Height
1	15.699	42431655	98.32	1651557	97.62
2	17.306	723664	1.68	40238	2.38

SAMPLE INFORMATION

Sample Name:	LXL-9032-20 IA10VS90	Acquired By:	Breeze
Sample Type:		Date Acquired:	2022/6/23 16:05:56 CST
Vial:	1:A,2	Acq. Method:	iPr vs Hex 10vs90
Injection #:	1	Date Processed:	2022/7/14 14:35:52 CST
Injection Volume:	5.00 ul	Channel Name:	304.0 nm
Run Time:	50.00 Minutes	Channel Desc.:	2998 (210-400) nm
Column Type:		Sample Set Name	LXL

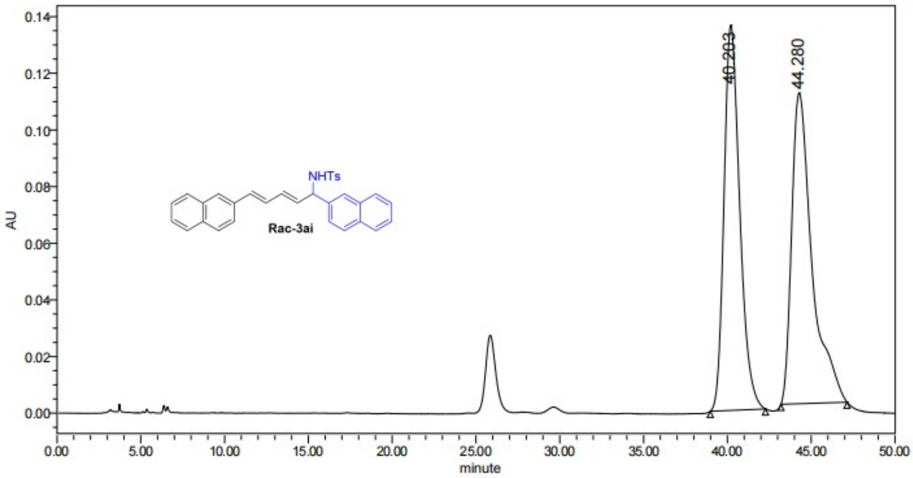

	RT (min)	Area (*sec)	% Area	Height	% Height
1	40.203	8874661	49.01	136004	55.36
2	44.280	9231858	50.99	109690	44.64

SAMPLE INFORMATION

Sample Name:	LXL-9078-2-10 10VS90	Acquired By:	Breeze
Sample Type:		Date Acquired:	2022/7/6 9:20:30 CST
Vial:	1:A,1	Acq. Method:	iPr vs Hex 10vs90
Injection #:	1	Date Processed:	2022/7/6 17:13:57 CST
Injection Volume:	5.00 ul	Channel Name:	304.1 nm
Run Time:	50.00 Minutes	Channel Desc.:	2998 (210-400) nm
Column Type:		Sample Set Name:	LXL

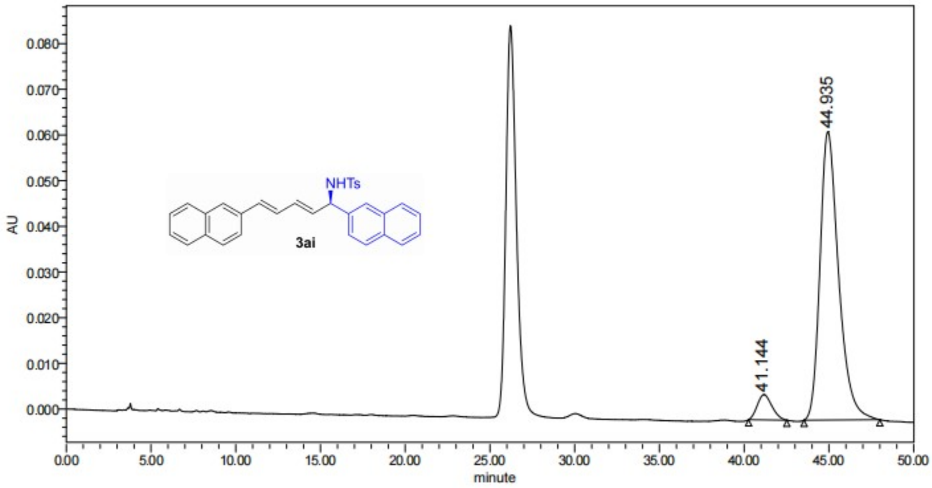

	RT (min)	Area (*sec)	% Area	Height	% Height
1	41.144	333505	6.48	5538	8.07
2	44.935	4816259	93.52	63119	91.93

SAMPLE INFORMATION

Sample Name:	lxl-CF3-1 ia 20vs80	Acquired By:	Breeze
Sample Type:		Date Acquired:	2023/1/2 10:11:41 CST
Vial:	1:A,1	Acq. Method:	iPr vs Hex 20vs80
Injection #:	1 Injection	Date Processed:	2023/1/6 20:01:59 CST
Volume: 5.00 ul		Channel Name:	321.1 nm
Run Time:	30.00 Minutes	Channel Desc.:	2998 (210-400) nm
Column Type:		Sample Set Name	15481

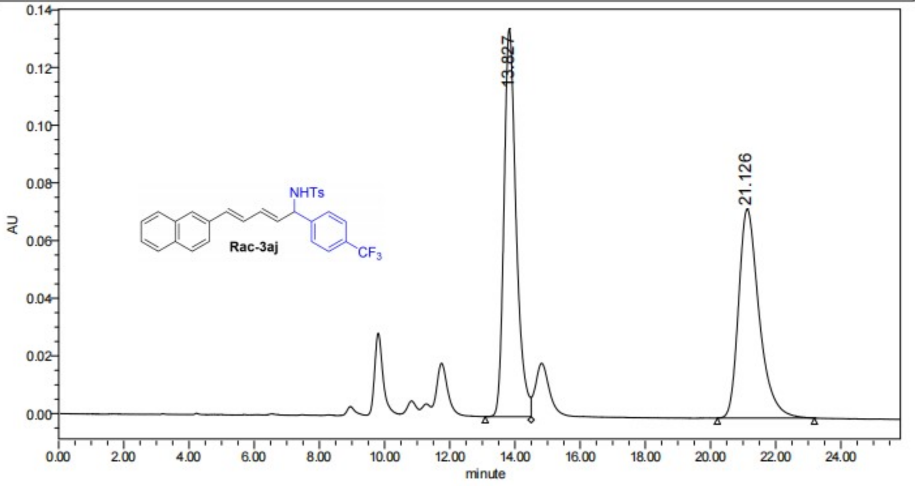

	RT (min)	Area (*sec)	% Area	Height	% Height
1	13.827	3489928	52.37	134549	64.97
2	21.126	3174554	47.63	72552	35.03

SAMPLE INFORMATION

Sample Name:	lxl-CF3-2 IA 20VS80	Acquired By:	Breeze
Sample Type:		Date Acquired:	2022/12/29 21:00:38 CST
Vial:	1:A,2	Acq. Method:	iPr vs Hex 20vs80
Injection #:	1 Injection	Date Processed:	2023/1/6 20:00:11 CST
Volume: 5.00 ul		Channel Name:	321.1 nm
Run Time:	30.00 Minutes	Channel Desc.:	2998 (210-400) nm
Column Type:		Sample Set Name	1531

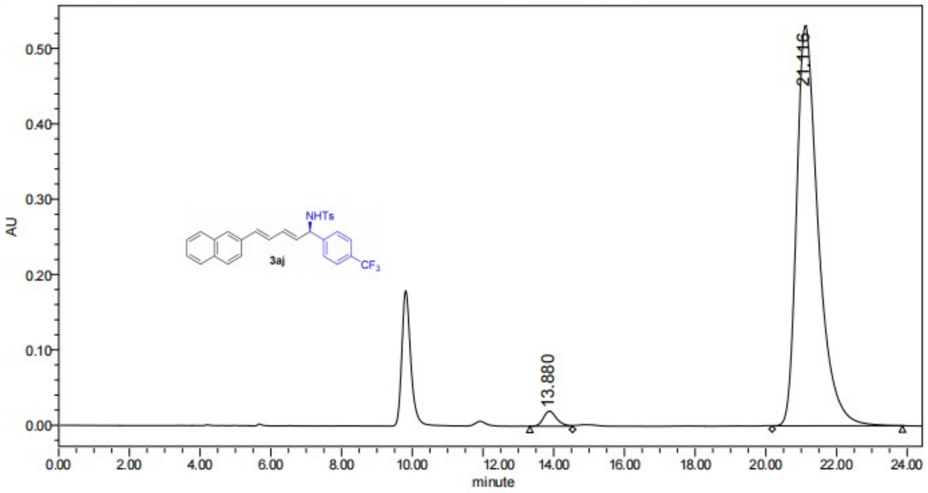

	RT (min)	Area (*sec)	% Area	Height	% Height
1	13.880	523558	2.27	19965	3.62
2	21.116	22577773	97.73	530942	96.38

SAMPLE INFORMATION

Sample Name:	LXL-9032-4 IA20VS80	Acquired By:	Breeze
Sample Type:		Date Acquired:	2022/5/20 23:22:42 CST
Vial:	1:A,1	Acq. Method:	iPr vs Hex 20vs80
Injection #:	1	Date Processed:	2022/7/12 16:36:58 CST
Injection Volume:	5.00 ul	Channel Name:	318.0 nm @3
Run Time:	30.00 Minutes	Channel Desc.:	2998 (210-400) nm
Column Type:		Sample Set Name:	LXL

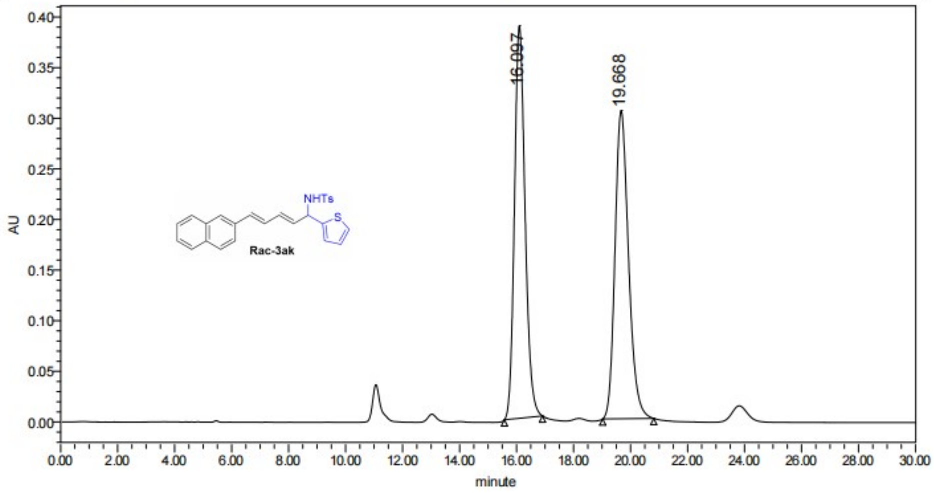

	RT (min)	Area (*sec)	% Area	Height	% Height
1	16.097	9998588	49.91	387679	56.01
2	19.668	10034958	50.09	304424	43.99

SAMPLE INFORMATION

Sample Name:	LXL-9078-2-11	Acquired By:	Breeze
Sample Type:		Date Acquired:	2022/7/6 8:40:55 CST
Vial:	1:A,1	Acq. Method:	iPr vs Hex 20vs80
Injection #:	1	Date Processed:	2022/7/6 9:10:02 CST
Injection Volume:	5.00 ul	Channel Name:	318.0 nm
Run Time:	25.00 Minutes	Channel Desc.:	2998 (210-400) nm
Column Type:		Sample Set Name	LXL

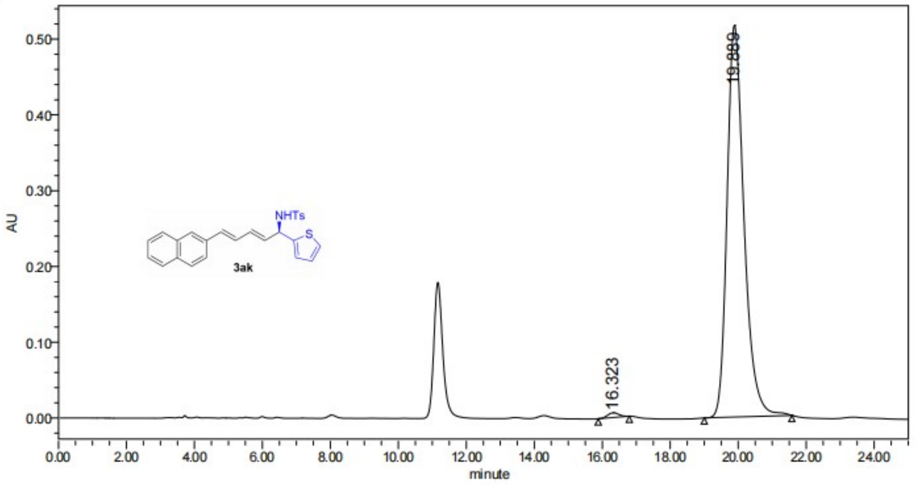

	RT (min)	Area (*sec)	% Area	Height	% Height
1	16.323	143309	0.79	6285	1.20
2	19.889	17894907	99.21	516874	98.80

SAMPLE INFORMATION

Sample Name:	LXL-90136-2 IA10VS90	Acquired By:	Breeze
Sample Type:		Date Acquired:	2022/10/15 11:41:36 CST
Vial:	1:A,1	Acq. Method:	iPr vs Hex 10vs90
Injection #:	1 Injection	Date Processed:	2022/10/15 14:17:34 CST
Volume: 5.00 ul		Channel Name:	280.0 nm
Run Time:	40.00 Minutes	Channel Desc.:	2998 (210-400) nm
Column Type:		Sample Set Name	1531

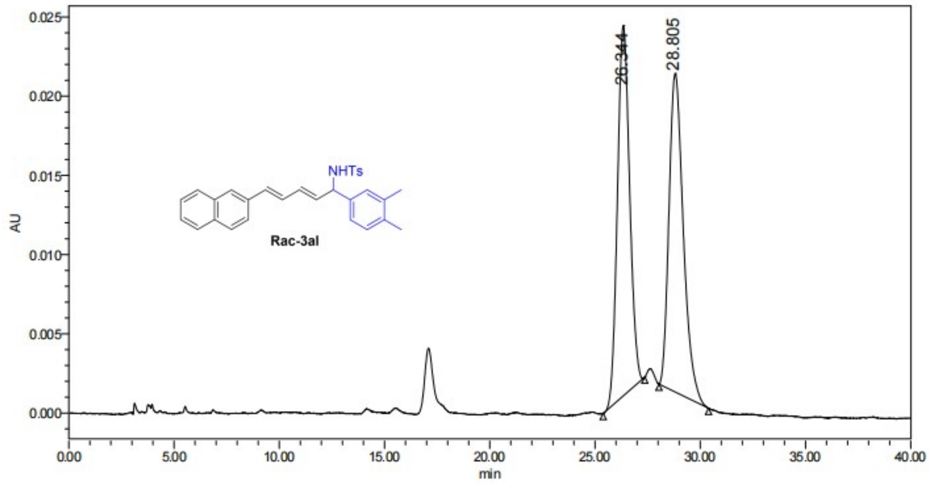

	RT (min)	Area (*sec)	% Area	Height	% Height
1	26.344	934555	49.57	23361	53.75
2	28.805	950865	50.43	20104	46.25

SAMPLE INFORMATION

Sample Name:	LXL-90136-10 IA10VS90	Acquired By:	Breeze
Sample Type:		Date Acquired:	2022/10/15 10:00:26 CST
Vial:	1:A,3	Acq. Method:	iPr vs Hex 10vs90
Injection #:	1 Injection	Date Processed:	2022/10/15 14:18:39 CST
Volume: 5.00 ul		Channel Name:	280.0 nm
Run Time:	40.00 Minutes	Channel Desc.:	2998 (210-400) nm
Column Type:		Sample Set Name	LXL

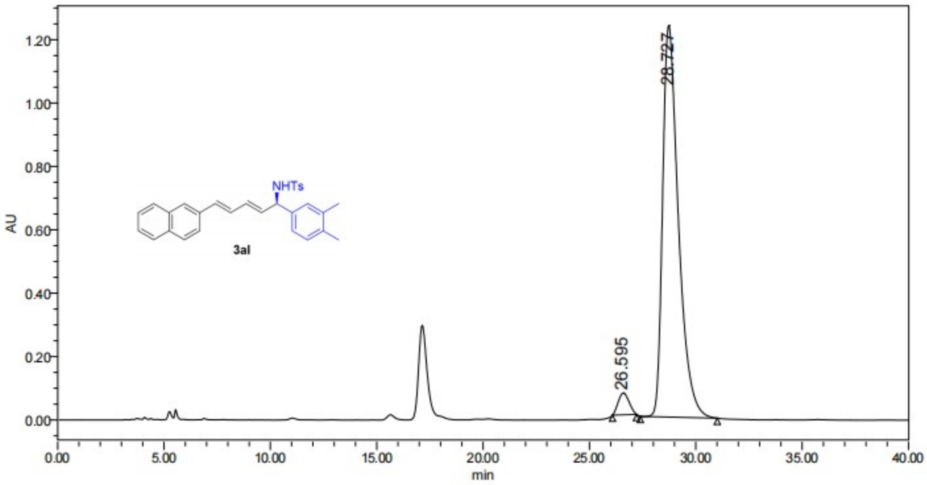

	RT (min)	Area (*sec)	% Area	Height	% Height
1	26.595	2418057	3.67	68550	5.26
2	28.727	63519950	96.33	1235263	94.74

SAMPLE INFORMATION

Sample Name:	lxl-Ph-1 IA 20VS80	Acquired By:	Breeze
Sample Type:		Date Acquired:	2023/1/3 15:39:27 CST
Vial:	1:A,1	Acq. Method:	iPr vs Hex 20vs80
Injection #:	1 Injection	Date Processed:	2023/1/6 20:04:37 CST
Volume: 5.00 ul		Channel Name:	321.1 nm
Run Time:	60.00 Minutes	Channel Desc.:	2998 (210-400) nm
Column Type:		Sample Set Name	2561

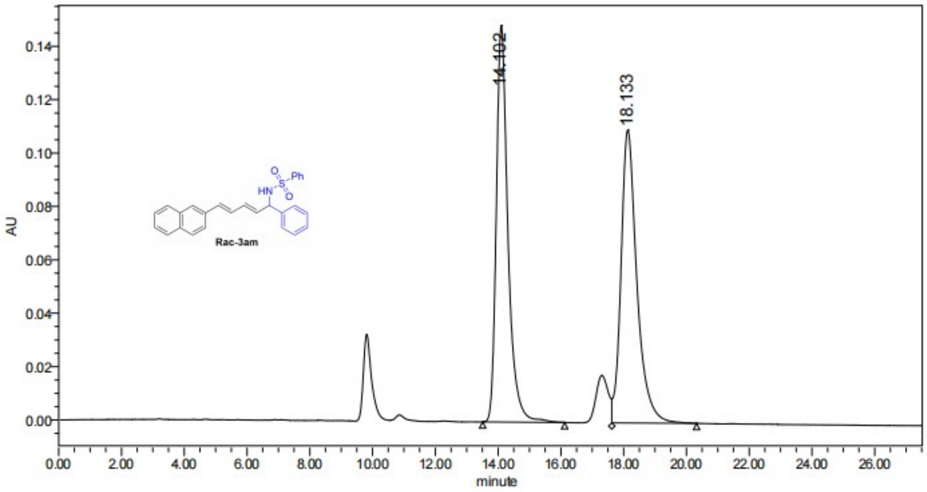

	RT (min)	Area (*sec)	% Area	Height	% Height
1	14.102	3819000	49.77	148503	57.51
2	18.133	3854262	50.23	109727	42.49

SAMPLE INFORMATION

Sample Name:	Ixl-Ph IA 20vs80	Acquired By:	Breeze
Sample Type:		Date Acquired:	2023/1/3 16:45:39 CST
Vial:	1:A,1	Acq. Method:	iPr vs Hex 20vs80
Injection #:	1 Injection	Date Processed:	2023/1/6 20:05:37 CST
Volume: 5.00 ul		Channel Name:	321.1 nm
Run Time:	60.00 Minutes	Channel Desc.:	2998 (210-400) nm
Column Type:		Sample Set Name	259

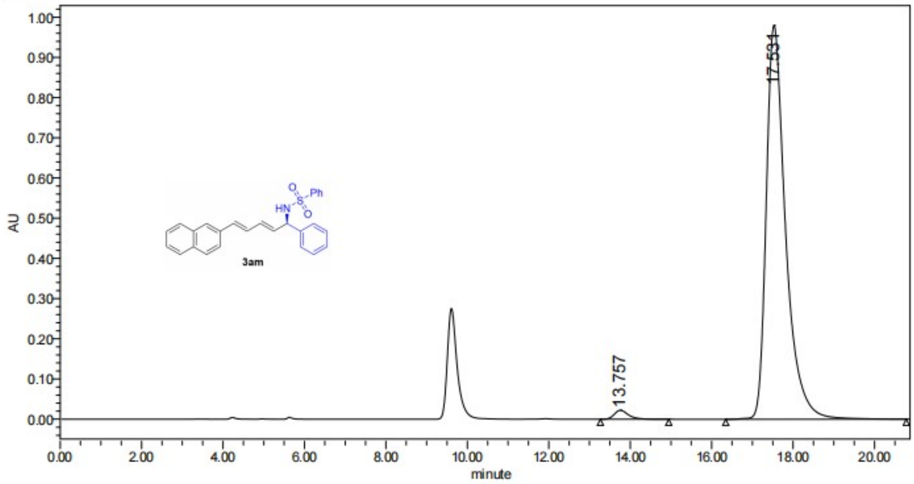

	RT (min)	Area (*sec)	% Area	Height	% Height
1	13.757	550437	1.67	22492	2.24
2	17.531	32338036	98.33	980251	97.76

SAMPLE INFORMATION

Sample Name:	Ixl-Cl-1 IA 20VS80	Acquired By:	Breeze
Sample Type:		Date Acquired:	2023/1/3 16:12:16 CST
Vial:	1:A,1 Injection	Acq. Method:	iPr vs Hex 20vs80
#:	1 Injection Volume:	Date Processed:	2023/1/6 20:05:08 CST
5.00 ul		Channel Name:	321.1 nm
Run Time:	60.00 Minutes	Channel Desc.:	2998 (210-400) nm
Column Type:		Sample Set Name	41561

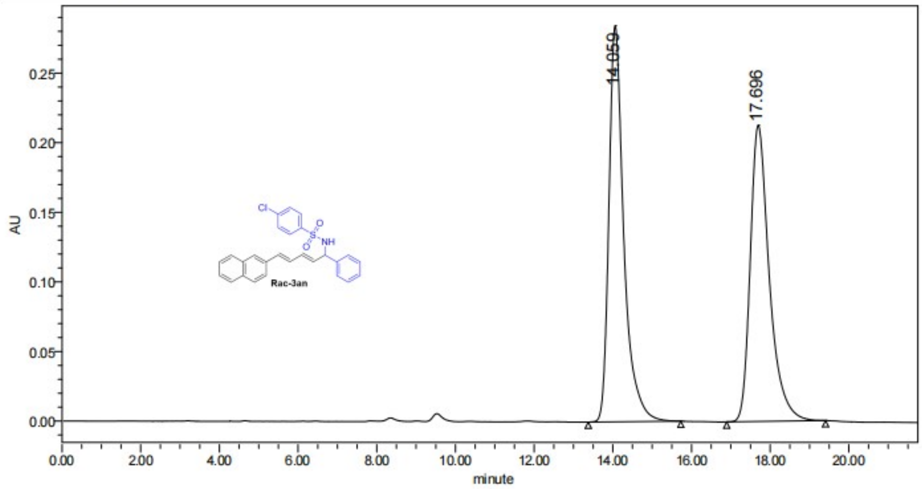

	RT (min)	Area (*sec)	% Area	Height	% Height
1	14.059	7470765	51.05	284586	57.21
2	17.696	7162326	48.95	212889	42.79

SAMPLE INFORMATION

Sample Name:	Ixl-Cl IA 20vs80	Acquired By:	Breeze
Sample Type:		Date Acquired:	2023/1/3 17:07:54 CST
Vial:	1:A,1	Acq. Method:	iPr vs Hex 20vs80
Injection #:	1	Date Processed:	2023/1/6 20:06:08 CST
Injection Volume:	5.00 ul	Channel Name:	321.1 nm
Run Time:	60.00 Minutes	Channel Desc.:	2998 (210-400) nm
Column Type:		Sample Set Name	1561

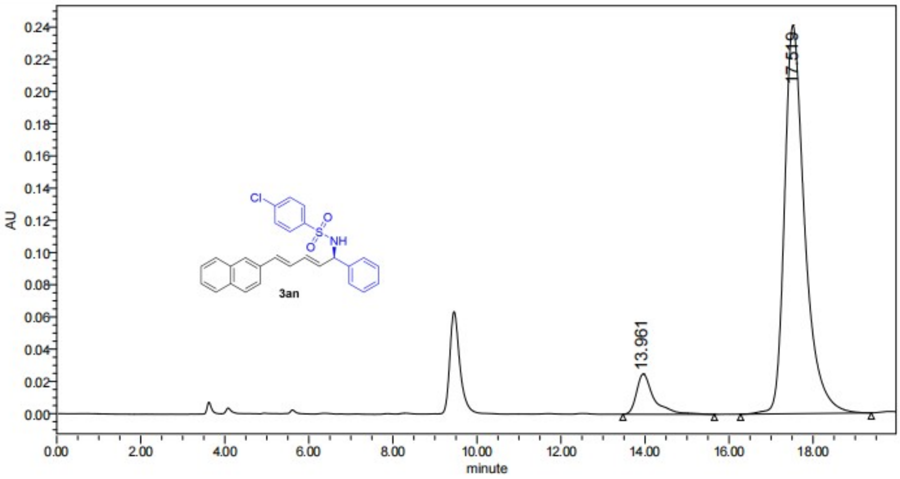

	RT (min)	Area (*sec)	% Area	Height	% Height
1	13.961	722891	8.21	25058	9.41
2	17.519	8081823	91.79	241269	90.59
